# Global mapping of antibiotic resistance rates among clinical isolates of *Stenotrophomonas maltophilia*: a systematic review and meta-analysis

**DOI:** 10.1186/s12941-024-00685-4

**Published:** 2024-03-19

**Authors:** Narjess Bostanghadiri, Mohammad Sholeh, Tahereh Navidifar, Leila Dadgar-Zankbar, Zahra Elahi, Alex van Belkum, Davood Darban-Sarokhalil

**Affiliations:** 1 Department of Microbiology, School of Medicine, Iran University of Medical Sciences, Tehran, Iran; 2 Microbial Biotechnology Research Center, Iran University of Medical Sciences, Tehran, Iran; 3 Department of Bacteriology, Pasteur Institute of Iran, Tehran, Iran; 4 Department of Basic Sciences, Shoushtar Faculty of Medical Sciences, Shoushtar, Iran; 5 Open Innovation & Partnerships, BaseClear, Leiden, Netherlands

**Keywords:** *Stenotrophomonas maltophilia*, Antibiotic resistance, Global prevalence, Systematic review, Meta-analysis

## Abstract

**Introduction:**

Infections caused by *Stenotrophomonas maltophilia* are clinically important due to its intrinsic resistance to a broad range of antibiotics. Therefore, selecting the most appropriate antibiotic to treat *S. maltophilia* infection is a major challenge.

**Aim:**

The current meta-analysis aimed to investigate the global prevalence of antibiotic resistance among *S. maltophilia* isolates to the develop more effective therapeutic strategies.

**Method:**

A systematic literature search was performed using the appropriate search syntax after searching Pubmed, Embase, Web of Science and Scopus databases (May 2023). Statistical analysis was performed using Pooled and the random effects model in R and the metafor package. A total of 11,438 articles were retrieved. After a thorough evaluation, 289 studies were finally eligible for inclusion in this systematic review and meta-analysis.

**Result:**

Present analysis indicated that the highest incidences of resistance were associated with doripenem (97%), cefoxitin (96%), imipenem and cefuroxime (95%), ampicillin (94%), ceftriaxone (92%), aztreonam (91%) and meropenem (90%) which resistance to Carbapenems is intrinsic. The lowest resistance rates were documented for minocycline (3%), cefiderocol (4%). The global resistance rate to TMP-SMX remained constant in two periods before and after 2010 (14.4% vs. 14.6%). A significant increase in resistance to tigecycline and ceftolozane/tazobactam was observed before and after 2010.

**Conclusions:**

Minocycline and cefiderocol can be considered the preferred treatment options due to low resistance rates, although regional differences in resistance rates to other antibiotics should be considered. The low global prevalence of resistance to TMP-SMX as a first-line treatment for *S. maltophilia* suggests that it remains an effective treatment option.

**Supplementary Information:**

The online version contains supplementary material available at 10.1186/s12941-024-00685-4.

## Introduction

*Stenotrophomonas maltophilia* (*S. maltophilia*) is commonly considered a microorganism with low virulence potential in humans. It is thus classified as an opportunistic pathogen, primarily affecting those with compromised immune systems. While *S. maltophilia* is infrequently detected within the oropharyngeal microbiota of healthy individuals, it is often found in the oropharynx of hospitalized individuals and those with cystic fibrosis [1]. In these two particular groups, it may cause persistent respiratory tract infection that may lead to inflammation, lung impairment, and sometimes even early death [2]. *Stenotrophomonas spp.* primarily cause hospital-acquired infections, with pneumonia being the most common manifestation. However, it can also lead to a variety of infections, including bloodstream, urinary, intra-abdominal, catheter and implanted device infections. In rare cases, it may cause heart-, bone-, soft tissue-, and nervous system infections [3]. *S. maltophilia* is a common pathogen in polymicrobial infections, and the rate of its isolation in the aforementioned infections ranges from 33 to 70%. In polymicrobial infections, the overall prognosis may be affected by interactions between different types of bacteria. For example, *P. aeruginosa* and *S. maltophilia* are able to form companion biofilms in the lungs, establishing an environment that is mutually beneficial to both of these bacterial species. This interaction has been associated with a higher mortality rate in pneumonia patients [4]. Effective management strategies for *S. maltophilia* infections are uncertain due to the limited number of treatment options available, supported by in vitro and clinical evidence. Furthermore, differentiating between colonization and invasive infections due to *S. maltophilia* can present a considerable challenge. Trimethoprim-sulfamethoxazole (TMP-SMX) is generally considered the preferred therapeutic option for the treatment of *S. maltophilia* infections based on promising in vitro activity and positive clinical outcomes [5]. Levofloxacin is generally considered an alternative antibiotic in case of resistance to TMP-SMX [6]. Other therapeutic alternatives, such as ceftazidime, ticarcillin-clavulanic acid, tigecycline, and colistin, have also been proposed. Infections caused by *Stenotrophomonas* spp. are clinically important due to their intrinsic resistance to a broad range of antibiotics, including most β-lactams [4, 5]. Antibiotic resistance is facilitated by different mechanisms involving plasmids, integrons, insertion sequence common region elements, antibiotic modifying enzymes, multidrug efflux pumps, and reduced outer membrane permeability to drugs [7]. Currently, beta-lactam antibiotics are not recommended for treating infections caused by *S. maltophilia* because two endogenous beta-lactamase genes are present intrinsically in all isolates of this bacterial species. The first one is a Class B zinc-dependent metallo-β-lactamase, identified as bla_*L1*_. This enzyme can hydrolyze all β-lactams, excluding aztreonam, and it is also unaffected by the β-lactamase inhibitors employed in clinical settings. The second is a class A serine-β-lactamase (bla_*L2*_) that is fortunately still susceptible to inhibition by presently available β-lactamase inhibitors but it can hydrolyze β-lactams, including cephalosporins and carbapenems [8, 9]. Efflux pumps of the Resistance Nodulation Division (RND) family, including SmeDEF and SmeYZ have an important role in conferring resistance to TMP-SMX and most antibiotics [10]. Moreover, animal strains significantly contribute to the genetic variation in the *S. maltophilia* complex, as they act as a source of mobile antibiotic resistance genes [11]. Hence, selecting the most appropriate antibiotic to treat *S. maltophilia* infection is a challenge. The main aim of the present study is to assess the global resistance rate of *S. maltophilia* to frequently prescribed antibiotics. Therefore, this meta-analysis of resistance rates may be useful in the development of innovative and robust therapeutic strategies.

## Methods

### Search strategy and study selection

Studies focused on *S. maltophilia* antimicrobial resistance were identified through a systematic search of online databases, including MEDLINE (PubMed), Web of Science, Embase, and Scopus (May 2023). The following search syntax was utilized for search in PubMed and other databases. The comprehensive search conducted using “*Stenotrophomonas maltophilia*”, “*s. maltophilia*” “antibiotic resistance” and all relevant keywords without any restriction during searching the databases. the search syntax is mentioned in supplementary file 1. We used Mesh Terms, Emtree, and the free text method to determine synonyms. This review was performed and documented in compliance with the guidelines of the Preferred Reporting Items for Systematic Reviews and Meta-Analyses (PRISMA) [12]. The records found through database searching were merged, and the duplicates were removed using EndNote 20 (Thomson Reuters, New York, NY, USA). To prevent bias, two reviewers independently screened the records by title/abstract and full text to exclude the irrelevant articles. The third author investigated any disparities.

### Selection criteria and data extraction

All qualified studies were extracted and sorted into an Excel spreadsheet (Microsoft, Redmond, WA): first author’s name, publication year, country, continent, sample collection date, the total number of *S. maltophilia* strains collected, diagnostic methods, antibiotic susceptibility test methodology (disk diffusion, dilution method, automated system), interpretative guidelines used (CLSI, EUCAST, Other) and the number/fraction of resistant isolates to each antibiotic (Supplementary Table S1). To mitigate the possibility of any inaccuracies in the extraction of data, two authors (NBGH and LD) extracted the necessary data independently and reached an agreement on any discrepant data. Eligibility criteria for incorporating articles in the meta-analysis were a report on the proportion of antibiotic resistance, determined sample size and availability of a full-text English-published format of the article. The following factors determined exclusion: (1) *S. maltophilia* was not detected; (2) *S. maltophilia* was isolated from animals or the environment; (3) *S. maltophilia* antibiotic resistance was not presented or only superficially reported as MIC50/90; (4) evaluation of the combined effects of antibiotics only; (5) when the guideline used was not specified; (6) when there was no clear reporting of resistance rates; (7) data were from conference abstracts, editorials, prior meta-analyses, systematic reviews, narrative reviews;(8) when an article was not available, in case of articles without full-text availability; (9) failure to access full articles even after repeated attempts to establish contact with the corresponding author via electronic mail.

### Quality assessment

Two blinded reviewers evaluated the research quality by utilizing a modified version of the assessment tool introduced by the Newcastle-Ottawa scale (NOS) adapted specifically for cross-sectional studies [13] (Supplementary Table S1). Each study was attributed scores of 0–4, 5–6, and 6–7, assigning low, moderate, and high quality, respectively. In instances where there was disagreement, a third reviewer was tasked with adjudication.

### Publication bias

Publication bias was statistically assessed using Egger’s and Begg’s tests, Funnel plot, Fail and safe and Trim and Fill.

### Definitions

Individuals were defined as being infected by *S. maltophilia* if they tested positive with appropriate phenotypical or molecular laboratory tests. The frequency of resistance was determined by a standard antimicrobial susceptibility test. Results from disk diffusion, dilution methods, and automated systems were accepted for the definition of resistance as well.

### Statistical analysis

The main target of the present study was to determine the global prevalence of antibiotic resistance among clinical isolates of *S. maltophilia* to different classes of antibiotics. The resistance rates for all antimicrobial agents are depicted through a forest plot diagram its pertinent 95% confidence interval (CI). Subgroup analysis was performed to investigate differences in prevalence between antibiotics, to compare the resistance rates based on countries and continents, antimicrobial susceptibility testing (AST) methods used (disk diffusion, dilution methods, automated systems), year of publication (1958–2010 versus 2011–2023), quality assessment scores, and AST guidelines applied (CLSI, EUCAST, Other) (Supplementary Table S2). Meta-regression analysis conducted by moderator analysis for publication years is shown in the supplementary Figure file. The examination was executed employing proportions as the resultant measures. A random-effects model was applied to all information gathered. The level of heterogeneity (i.e., τ^2^) was estimated using the DerSimonian-Laird estimator [14]. In addition to the estimate of τ^2^, the Q-test for heterogeneity and the I^2^ statistic are reported. Studies with a studentized residual larger than the 100 × (1-0.05/(2×k))th percentile of a standard normal distribution were considered potential outliers and were excluded from the analyses. The rank correlation test [15] and the regression test [16] used the standard error of the observed outcomes as a predictor to check for funnel plot asymmetry. The analysis was carried out using R (version 4.2.1) and the metafor package (version 3.8.1) [17, 18]. *P* < 0.05 was considered statistically significant.

## Results

### Search results

**Fig. 1:**
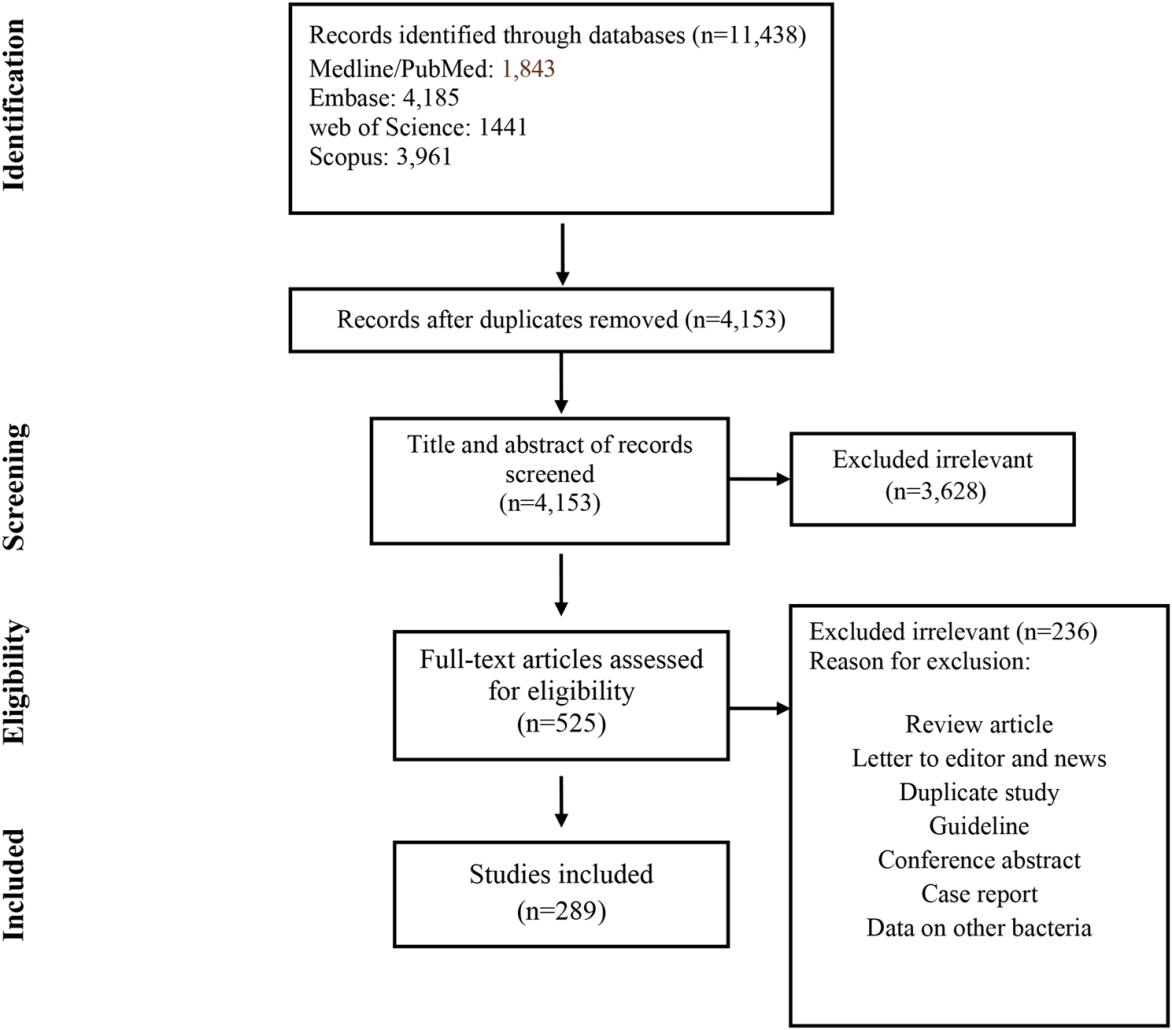
Flow chart of study selection for inclusion in the systematic review and meta-analysis

The process for the selection of articles is shown in Fig. 1. A total of 11,438 articles was identified by searching the four electronic databases mentioned above.

After removing duplicates (*n* = 7285), the titles and abstracts of 4664 articles were screened. Of these, 525 met the inclusion criteria and were retained for full-text review. Of the 525 studies, 236 were excluded because they were non-original studies, conference abstracts, reviews, articles without full text, studies with inappropriate data, or lacking susceptibility testing data or resistance data. A total of 289 studies were finally eligible for inclusion in the present systematic review and meta-analysis [19–308] (Supplementary Table S1).

### Characteristics of the included studies

Overall, the analysis encompassed a total of 289 studies conducted between the years 1958 and 2023. These articles exhibited an extensive geographical reach, with studies executed in various regions across the globe. Most of the studies were sourced from Asia (*n* = 125, 42.95%), followed by the European region (*n* = 86, 29.55%), North America (*n* = 43, 14.77%), South America (*n* = 10, 3.43%), Africa (*n* = 9, 3.09%), and, finally, Australia (*n* = 1, 0.3%). A total of seventeen studies (5.8%) were conducted simultaneously on different continents and therefore classified as multi-continental. Utilization of standards in interpreting susceptibility outcomes with the application of dissimilar breakpoints displayed variation. Amongst the guidelines utilized in the interpretation of antimicrobial susceptibilities, those from Clinical & Laboratory Standards Institute (CLSI) were the most widely employed. The NOS critical appraisal checklist was utilized to evaluate the reviewed studies’ characteristics. Out of the 289 studies that were analysed, 72 (24.7%) received high-quality scores, 190 (65.3%) received moderate-quality scores, and 29 (10%) received low-quality scores. Resistance to trimethoprim-sulfamethoxazole and ceftazidime was detected in most studies (*n* = 225, 77.31%) included in the meta-analysis.

### Meta-analysis results

The resistance rate to different antimicrobial agents and the subgroup analysis by continent, country, year of publication, method of susceptibility testing, quality score, and guideline were presented in supplementary Table S2 and Fig. 3-5. Furthermore, a more comprehensive examination has been conducted below regarding the dissemination of resistance towards certain crucial antibiotics highlighted in the CLSI, EUCAST, and FDA reports Fig. 6.

**Fig. 2:**
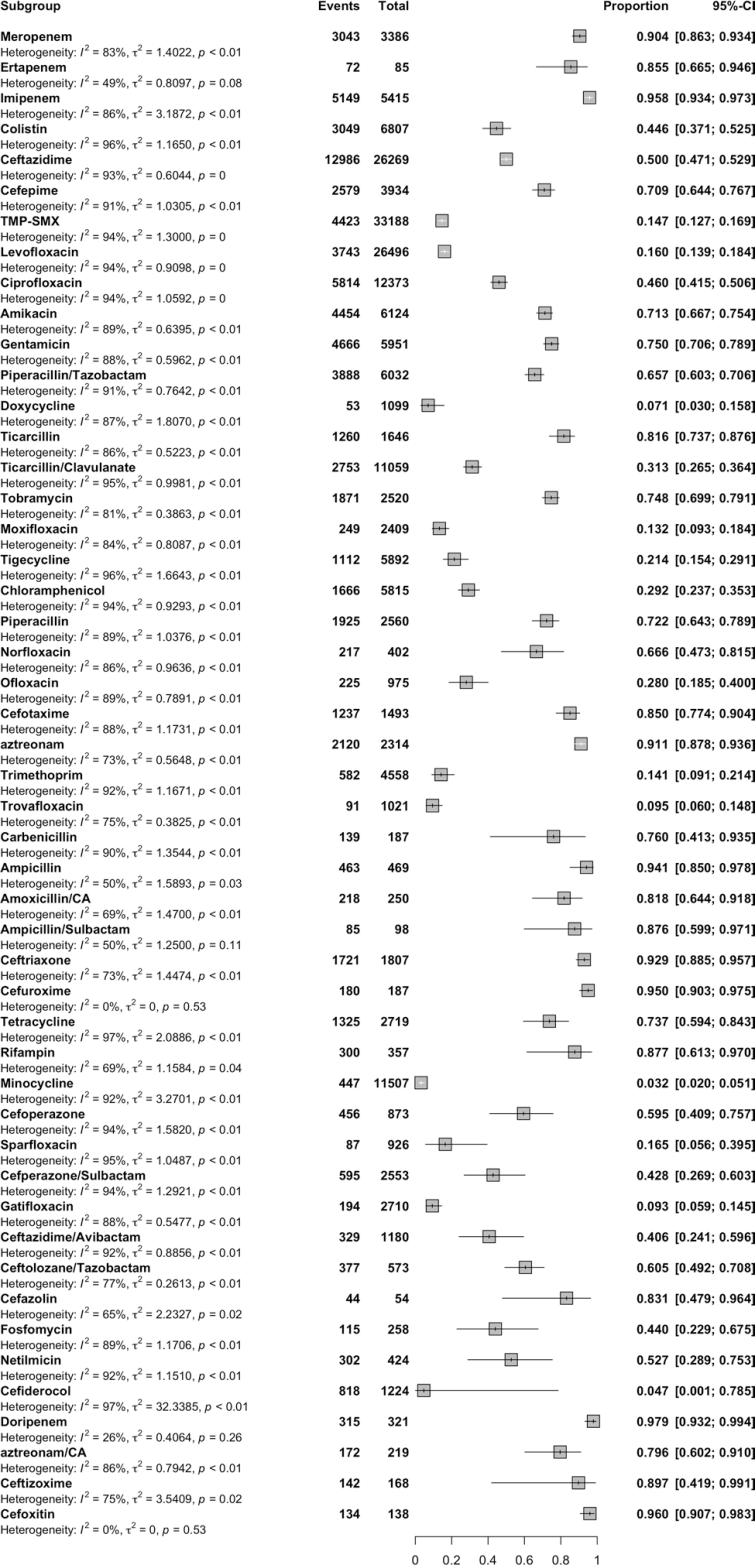
Forest plot of the global antibiotic resistance rates of *S. maltophilia* isolation from clinical samples

According to the results as shown in the forest plot diagram in Fig. 2, the highest resistance rate was documented for doripenem (97%), cefoxitin (96%), imipenem and cefuroxime (95%), ampicillin (94%), ceftriaxone (92%), aztreonam (91%) and meropenem (90%), respectively. The lowest resistance rates were found for minocycline (3%), cefiderocol (4%), doxycycline (7%) and gatifloxacin (9%).

**Fig. 3:**
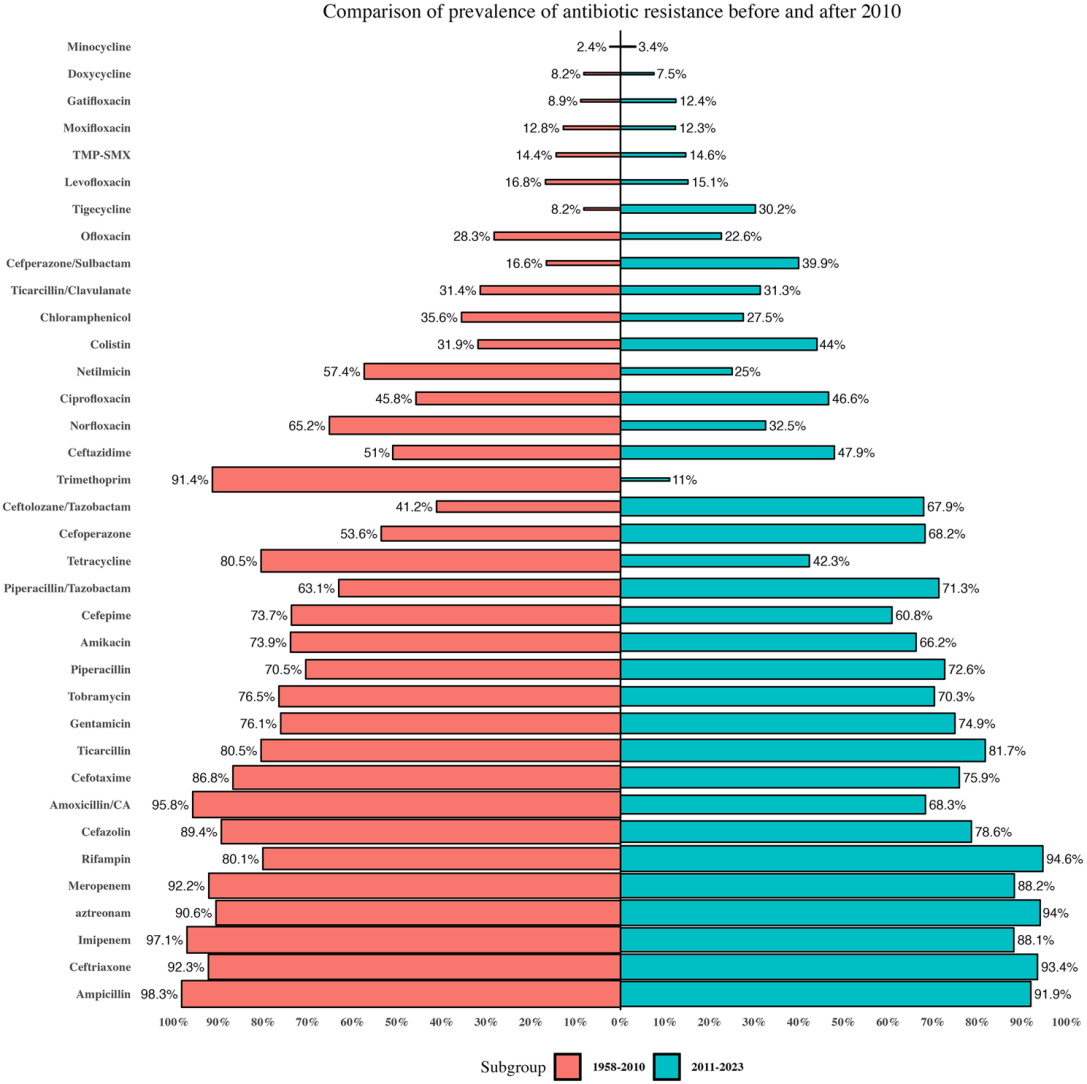
Prevalence of antibiotic resistance among *S. maltophilia* by continent

**Fig. 4:**
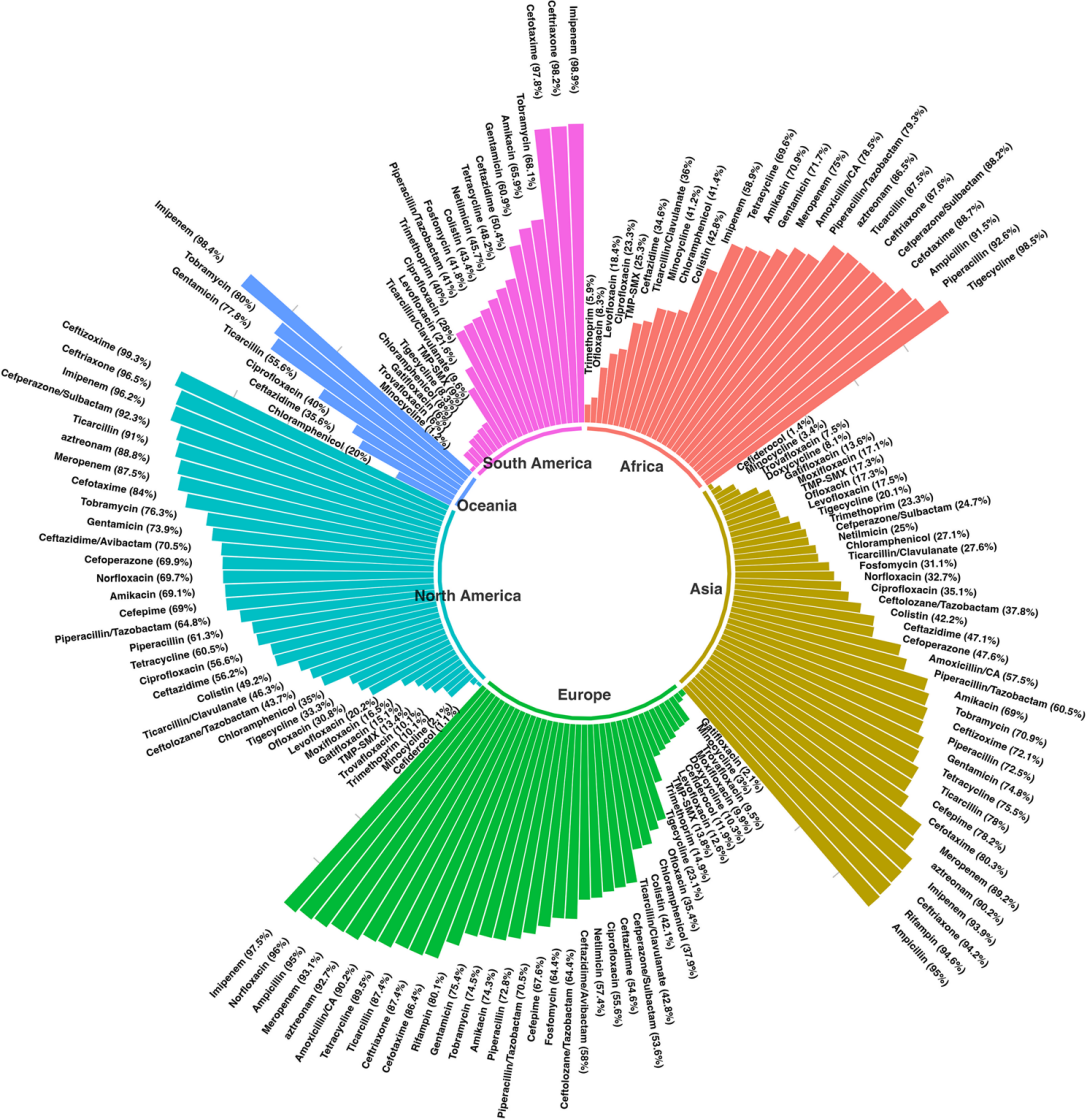
Comparison of antibiotic resistance among *S. maltophilia* strains stratified by continent of origin

Comparing the antibiotic resistance rates among *S. maltophilia* collections according to the continent of origin (Figs. 3 and 4) revealed that the highest resistance to imipenem was reported from South America (98%), Australia (98%), and Europe (97%). While the highest rate of resistance in other continents, such as North America, Africa, and Asia was found for ceftizoxime (99%), tigecycline (98%), and ampicillin (95%).

**Fig. 5:**
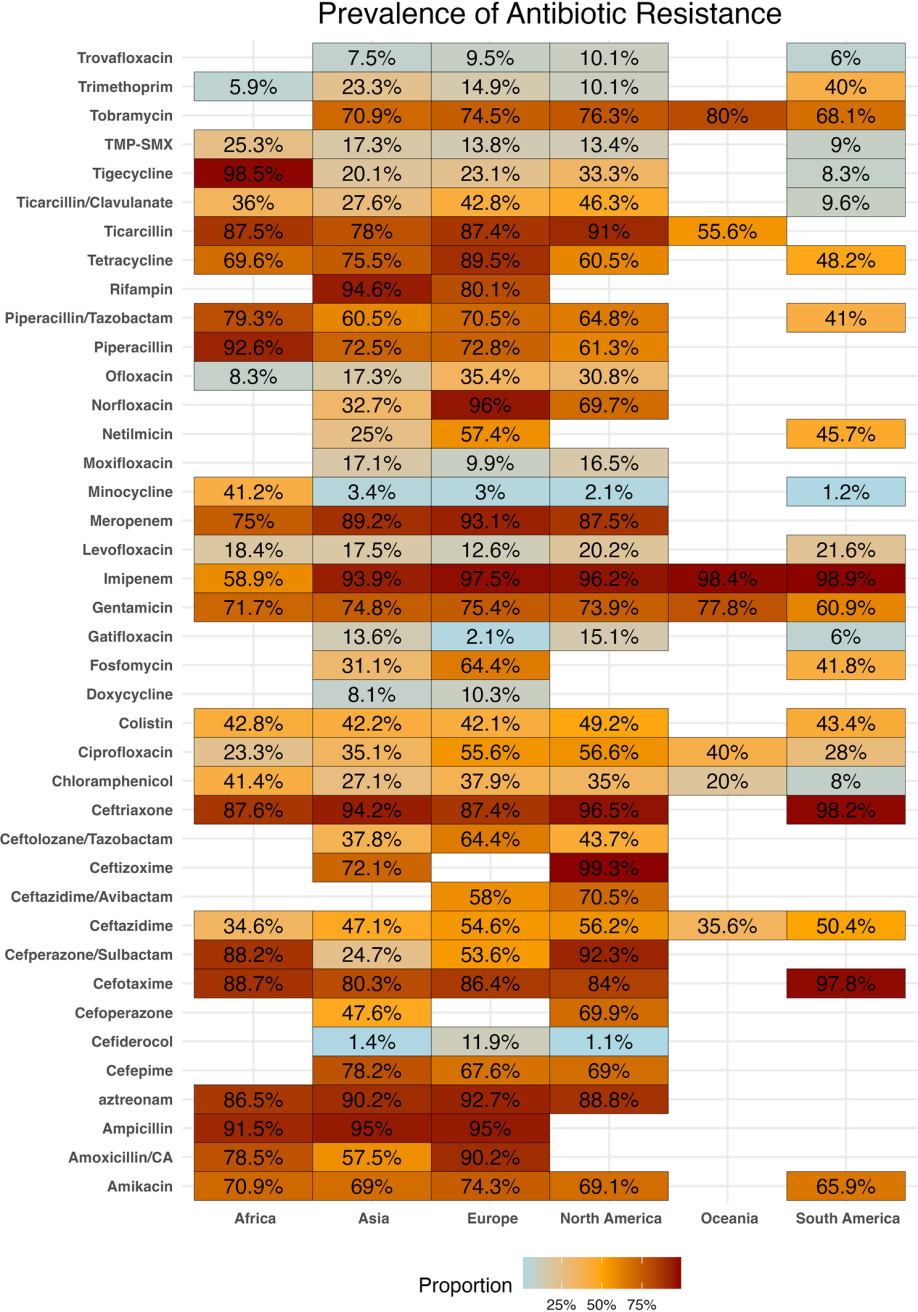
Global antibiotic resistance rates of *S. maltophilia* during 1958–2010 and 2011–2023

A subgroup meta-analysis examined antibiotic resistance rates before and after 2010. This revealed a considerable escalation in resistance toward certain antibiotics such as tigecycline, and ceftolozan/tazobactam in more recent times. In contrast, we found a decreased resistance rate against trimethoprim, tetracycline, imipenem, and amoxicillin/clavulanate (Fig. 5). Based on the results of Egger’s and Begg’s tests, there was a publication bias in the meta-analysis of imipenem, levofloxacin, minocycline. Due to this publication bias, the proportion of resistance to imipenem, levofloxacin, minocycline changed to 0.92, 0.20 and 0.06, respectively, after applying the fill and trim method (Table 1).

**Table 1:** Results of fill and trim, egger and begg test, and fail-safe

Different classes	Antibiotic	Egger test	Begg test	Fail and safe	Fill and Trim
Penicillins	Ampicillin	0.669	0.097	232	0.941 (0.850, 0.978)
	Carbenicillin	0.051	> 0.999	16	0.760 (0.413, 0.935)
	Ticarcillin	0.012	0.169	1290	0.766 (0.675, 0.837)
	Piperacillin	0.949	0.619	3728	0.671 (0.581, 0.750)
Phosphonic antibiotic	Fosfomycin	0.680	> 0.999	0	0.440 (0.229, 0.675)
Monobactams	Aztreonam	0.072	0.615	6624	0.905 (0.870, 0.932)
β-lactam/β-lactamase inhibitor	Amoxicillin/ clavulanate	0.248	0.731	126	0.784 (0.589, 0.901)
	Ampicillin/sulbactam	0.315	> 0.999	24	0.876 (0.599, 0.971)
	Aztreonam/ clavulanate	< 0.001	0.333	68	0.796 (0.602, 0.910)
	Cefperazone/sulbactam	0.166	0.163	463	0.253 (0.140, 0.415)
	Ceftazidime/avibactam	< 0.001	0.399	0	0.480 (0.290, 0.675)
	Piperacillin/tazobactam	0.771	0.765	8069	0.623 (0.568, 0.675)
	Ticarcillin/clavulanate	0.119	0.302	23,374	0.337 (0.285, 0.393)
Cephalosporins	Ceftazidime	0.016	0.432	0	0.456 (0.427, 0.486)
	Cefazolin	0.003	0.817	11	0.494 (0.157, 0.836)
	Cefepime	0.555	0.605	5321	0.673 (0.607, 0.733)
	Cefiderocol	0.013	0.239	46	0.101 (0.004, 0.758)
	Cefoperazone	0.102	0.675	0	0.509 (0.329, 0.687)
	Cefotaxime	0.086	0.741	2388	0.780 (0.688, 0.850)
	Cefoxitin	0.956	> 0.999	38	0.964 (0.920, 0.985)
	Ceftizoxime	0.598	> 0.999	23	0.897 (0.419, 0.991)
	Ceftriaxone	0.315	0.229	3851	0.884 (0.816, 0.930)
	Cefuroxime	0.848	0.817	99	0.950 (0.903, 0.975)
Carbapenems	Imipenem	< 0.001	< 0.001	20,049	0.921 (0.883, 0.947)
	Meropenem	0.277	0.306	11,163	0.852 (0.792, 0.897)
	Doripenem	0.100	> 0.999	81	0.963 (0.887, 0.989)
	Ertapenem	0.060	0.719	42	0.750 (0.527, 0.890)
Aminoglycosides	Amikacin	0.259	0.342	13,594	0.702 (0.655, 0.744)
	Gentamicin	0.061	0.055	16,886	0.722 (0.674, 0.766)
	Netilmicin	0.188	0.697	16	0.550 (0.318, 0.762)
	Tobramycin	0.302	0.594	4895	0.730 (0.679, 0.775)
fluoroquinolones	Gatifloxacin	0.097	0.542	2777	0.093 (0.059, 0.145)
	Ciprofloxacin	0.979	0.159	2405	0.482 (0.437, 0.528)
	Levofloxacin	< 0.001	0.015	247,151	0.207 (0.181, 0.235)
	Moxifloxacin	0.422	0.276	5131	0.164 (0.115, 0.228)
	Norfloxacin	< 0.001	0.358	1	0.450 (0.257, 0.659)
	Sparfloxacin	0.128	> 0.999	252	0.165 (0.056, 0.395)
	Ofloxacin	0.290	0.858	515	0.280 (0.185, 0.400)
	Trovafloxacin	0.160	0.358	1041	0.099 (0.063, 0.153)
Tetracyclines	Tetracycline	< 0.001	0.497	249	0.643 (0.494, 0.769)
	Doxycycline	0.019	0.165	763	0.097 (0.043, 0.207)
	Tigecycline	0.568	0.710	10,995	0.214 (0.154, 0.291)
	Minocycline	< 0.001	0.003	32,012	0.064 (0.042, 0.096)
Sulfonamides	Trimethoprim/ sulfamethoxazole	< 0.001	0.436	399,468	0.196 (0.171, 0.224)
	Trimethoprim	0.343	0.440	5604	0.141 (0.091, 0.214)
Polymyxins	Colistin	0.561	0.951	206	0.446 (0.371, 0.525)
Other	Chloramphenicol	0.023	0.888	8714	0.338 (0.276, 0.406)
Rifamycin	Rifampin	0.229	0.333	71	0.877 (0.613, 0.970)

**Fig. 6:**
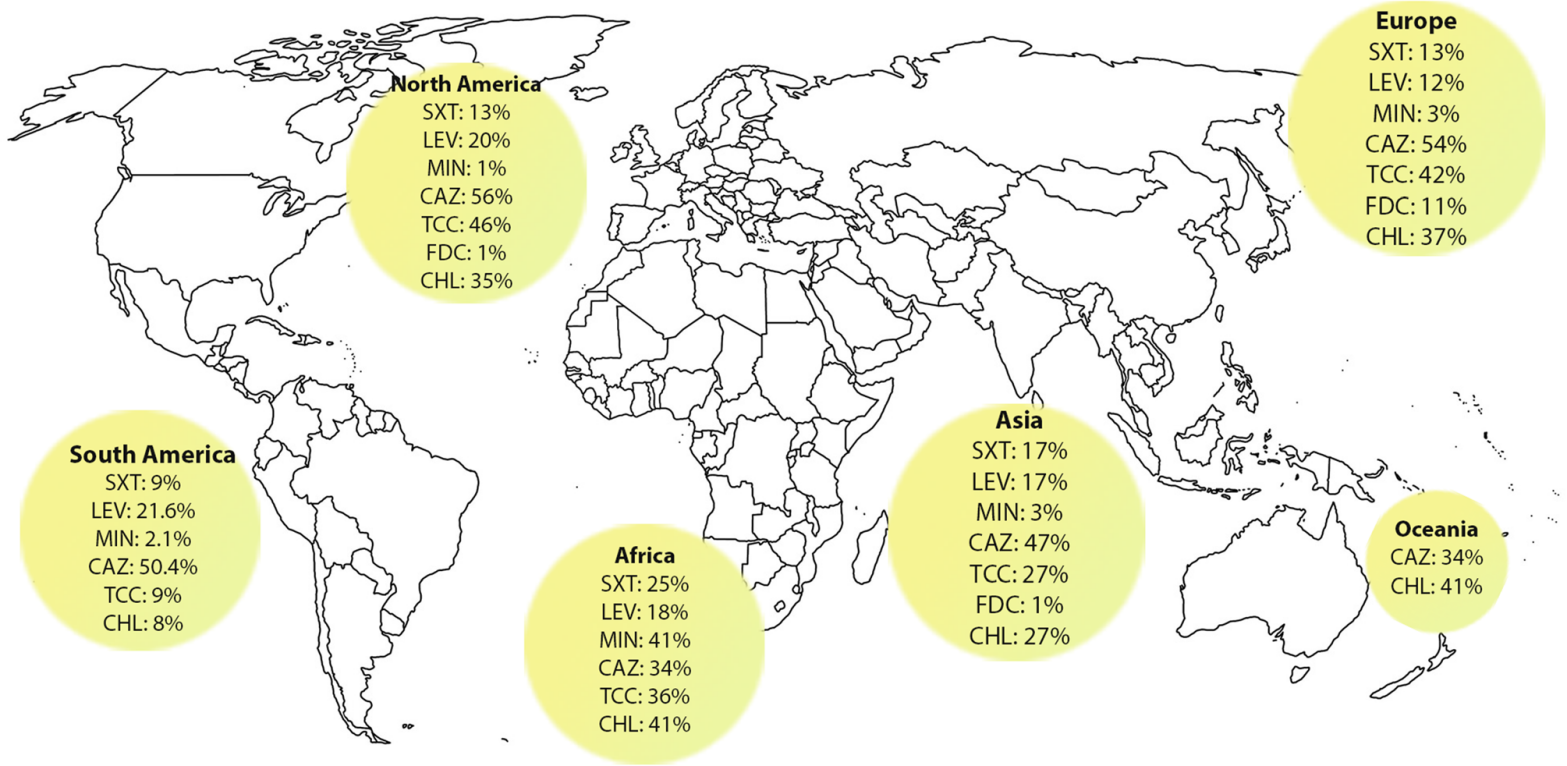
Comparison of recommended antibiotic resistance among *S. maltophilia* around the world. SXT: Cotrimoxazole; LEV: Levofloxacin; MIN: Minocycline; CZA: Ceftazidime; TCC: Ticarcilin-Clavunic acid; FDC: Cefiderocol; CHL: Chloramphenicol

### Prevalence of TMP-SMX resistance

A total of 33,188 isolates that were investigated in 225 studies were subjected to TMP-SMX resistance testing. The estimated average proportion through the employment of the random-effects model was *µ* = 0.147 (95%CI, 0.127, 0.169). The Q-test analysis presented evidence of heterogeneity among the actual outcomes Q (224) = 3955.963, I^2^ = 94.34%, *P* < 0.001. There was no significant difference in subgroup analysis (*P* > 0.05). According to the results of the meta-regression analysis, the prevalence of resistance was not correlated to the year of publication (*r* = 0.010, *P* = 0.394).

### Prevalence of ceftazidime resistance

A total of 26,269 isolates that were investigated in 225 studies were subjected to ceftazidime resistance testing. The estimated average proportion by employing the random-effects model was *µ* = 0.500 (95% CI, 0.471, 0.529). The Q-test analysis presented evidence of heterogeneity among the actual outcomes Q (224) = 3422.047, I^2^ = 93.45%, *P* < 0.001. Due to the subgroup analysis, the difference between countries and AST guidelines was significant (*P* < 0.001). Hungary exhibited the highest resistance level among the countries that provided data on the prevalence of resistance to ceftazidime. At the same time, Poland demonstrated the lowest resistance level compared to the other countries (0.98 and 0.14, respectively). Most of the literature consulted the CLSI guidelines to determine the resistance level (Supplementary Table S2). According to the results of the meta-regression analysis, the prevalence of resistance was not correlated to the year of publication (*r* = -0.004, *P =* 0.572).

### Prevalence of levofloxacin resistance

A total of 26,496 isolates that were investigated in 163 studies were subjected to levofloxacin resistance testing. The estimated average proportion by employing the random-effects model was *µ* = 0.160 (95%CI, 0.139, 0.184). The Q-test analysis presented evidence of heterogeneity among the actual outcomes Q (162) = 2580.510, I^2^ = 93.72%, *P* < 0.001. As a consequence of the subgroup analysis, a significant difference was found between continents and countries (*P* < 0.001). South America displayed the highest prevalence of resistance (0.21). Compared to the other countries that reported the prevalence of resistance, Malawi exhibited the highest resistance level (0.84). The automated system detected the highest number of resistant isolates. (Supplementary Table S2). The rank correlation and the regression test demonstrated potential funnel plot asymmetry (0.015 and < 0.001, respectively). Due to this publication bias, the implementation of the fill and trim method changed the proportion to 0.207 (95%CI, 0.181, 0.235). According to the results of the meta-regression analysis, the prevalence of resistance was not correlated to the year of publication (*r* = -0.023, *P =* 0.119).

### Prevalence of chloramphenicol resistance

A total of 5815 isolates that were investigated in 56 studies were subjected to chloramphenicol resistance testing. The estimated average proportion through the employment of the random-effects model was *µ* = 0.292 (95%CI, 0.237, 0.353). The Q-test analysis presented evidence of heterogeneity among the actual outcomes Q (55) = 882.229, I^2^ = 93.77%, *P* < 0.001. As a consequence of the subgroup analysis, a significant difference was found between the AST method (*P* = 0.01). The automated system yielded the greatest quantity of resistant isolates. (0.51). According to the results of the meta-regression analysis, the prevalence of resistance was not correlated to the year of publication (*r* = 0.011, *P* = 0.629).

### Prevalence of ticarcillin/clavulanic acid resistance

A total of 11,059 isolates that were investigated in 85 studies were subjected to ticarcillin/clavulanate resistance testing. The estimated average proportion through the employment of the random-effects model was *µ* = 0.313 (95% CI, 0.265, 0.364). The Q-test analysis presented evidence of heterogeneity among the actual outcomes (84) = 1649.878, I^2^ = 94.91%, *P* < 0.001. As a consequence of the subgroup analysis, the difference between countries and continents was significant (*P* < 0.001). Concerning the continents, North America and Europe displayed the highest prevalence of ticarcillin/clavulanic resistance (0.46 and 0.42, respectively). Furthermore, among the countries that reported the prevalence, Switzerland and Canada showed the most significant resistance level compared to other countries (0.87 and 0.73, respectively). (Supplementary Table S2). According to the results of the meta-regression analysis, the prevalence of resistance was not correlated to the year of publication (*r* = -0.001, *P =* 0.930).

### Prevalence of tigecycline resistance

A total of 5892 isolates that were investigated in 45 studies were subjected to tigecycline resistance testing. The estimated average proportion through the employment of the random-effects model was *µ* = 0.214 (95%CI, 0.154, 0.291). The Q-test analysis presented evidence of heterogeneity among the actual outcomes Q (44) = 1017.756, I^2^ = 95.68%, *P* < 0.001. Due to the subgroup analysis, the difference between countries, continents, and the AST method was significant (*P* < 0.001). Concerning the continents, Africa displayed the highest prevalence of resistance (0.98). Furthermore, among the countries that reported the rate of resistance, Egypt showed the most significant resistance level compared to other countries (0.98). Most of the literature consulted the dilution method to determine the resistance (Supplementary Table S2). According to the results of the meta-regression analysis, the prevalence of resistance was weakly correlated to the year of publication (*r* = 0.212, *P* < 0.001).

### Prevalence of minocycline resistance

A total of 11,507 isolates that were investigated in 71 studies were subjected to minocycline resistance testing. The estimated average proportion through the employment of the random-effects model was *µ* = 0.032 (95%CI, 0.020, 0.051). The Q-test analysis presented evidence of heterogeneity among the actual outcomes Q (70) = 919.005, I^2^ = 92.38%, P = < 0.001. Due to the subgroup analysis, the difference between the continent and AST methods was significant (*P* < 0.001). Concerning the continents, Africa displayed the highest prevalence of resistance (0.41). The dilution method was the predominant technique utilized in the literature to determine resistance. The majority of the literature consulted the CLSI guidelines to determine the resistance level (Supplementary Table S2). The rank correlation and the regression test indicated potential funnel plot asymmetry (0.003 and < 0.001, respectively). Due to this publication bias, the implementation of the fill and trim method resulted in a proportion change of 0.064 (95%CI, 0.042, 0.096). According to the results of the meta-regression analysis, the prevalence of resistance was not correlated to the year of publication (*r* = 0.050, *P =* 0.270).

### Prevalence of colistin resistance

A total of 6807 isolates that were investigated in 57 studies were subjected to colistin resistance testing. The estimated average proportion through the employment of the random-effects model was *µ* = 0.446 (95%CI, 0.371, 0.525). The Q-test analysis presented evidence of heterogeneity among the actual outcomes Q (56) = 1364.348, I^2^ = 95.90%, *P* < 0.001. As a consequence of the subgroup analysis, a significant difference was found between countries and the AST method (*P* < 0.001). Somalia displayed the highest prevalence of resistance compared to the other countries (0.97). The most resistant isolates were detected by dilution methods (0.53). According to the results of the meta-regression analysis, the prevalence of resistance was not correlated to the year of publication (*r* = 0.017, *P* = 0.660).

### Prevalence of cefiderocol resistance

A total of 1224 isolates that were investigated in 7 studies were subjected to cefiderocol resistance testing. The estimated average proportion through the employment of the random-effects model was *µ* = 0.047 (95% CI, 0.001, 0.785). The Q-test analysis presented evidence of heterogeneity among the actual outcomes Q (6) = 175.191, I^2^ = 96.58%, *P* < 0.001. There was no significant difference in subgroup analysis (*P* > 0.05). According to the results of the meta-regression analysis, the prevalence of resistance was not correlated to the year of publication (*r* = 2.312, *P =* 0.294).

## Discussion

Several antimicrobial agents have been recommended for treating *S. maltophilia* infections and TMP-SMX, minocycline, tigecycline, levofloxacin, and cefiderocol are among these [8]. TMP-SMX and minocycline are recommended by the Infectious Diseases Society of America (IDSA) panel as the preferred drugs for monotherapy of mild infections and as part of combination therapy for moderate to severe infections. A second agent such as minocycline (preferred), tigecycline, levofloxacin, or cefiderocol can be added in case of a slow response to monotherapy [5]. The accurate performance of AST for *S. maltophilia* remains challenging due to the limited clinical data available regarding the relevance of in vitro assays. According to CLSI reviewed *S. maltophilia* breakpoints in 2019 because of this limitation, the CLSI *S. maltophilia* breakpoints have not been updated [309]. The lack of uniform breakpoints can be attributed to several unknown factors, including microbiological, clinical and pharmacokinetic/pharmacodynamic PK/PD data typically used to establish such breakpoints [310, 311]. The inadequate information available may have a considerable impact on the treatment of patients. Still, CLSI has established MIC interpretation criteria for seven antibiotics, including TMP-SMX, ticarcillin-clavulanate, ceftazidime, cefiderocol, levofloxacin, minocycline, and chloramphenicol. In contrast, The European Committee on Antimicrobial Susceptibility Testing (EUCAST) has defined MIC and disc breakpoints for only TMP-SMX. However, ticarcillin-clavulanate is no longer in production, and the utilization of chloramphenicol is infrequent in the United States due to its considerable toxicity. This leaves only five agents with interpretable antibiotic MIC data available to clinicians and for clinically relevant application in AST [4, 5]. Our comprehensive meta-analysis found that most studies were performed in the Asian continent (*n* = 125) and the highest and lowest global resistant rates were for doripenem and minocycline, respectively (Fig. 2). The general prevalence of resistance to TMP-SMX stands at a rather low 14.7%. Notably, South America has recorded the lowest rate of resistance. In investigating the global antibiotic resistance of *S. maltophilia*, two meta-analysis studies were conducted by Banar et al. [311] and Dadashi et al. [312]. The main advantage of our study compared to those is that we analyse antibiotic resistance rates over a wider time period (1958- May 2023) and accomplish a comprehensive investigation of resistance rates to further antibiotics. The aforementioned studies found that the resistance rate to TMP-SMX was lower than the rate observed in our study. These discrepancies can be explained due to their fewer studies than ours and the inclusion of studies that only used the CLSI guidelines to interpret antibiotic susceptibility data by Dadashi et al. [312]. Furthermore, the prevalence of resistance to this drug has not exhibited significant change (*p* > 0.01) during two distinct periods (14.4% from 1958 to 2010 vs. 14.6% from 2011 to 2023), thereby indicating the constant efficacy of this drug in managing *S. maltophilia* infections. Epidemiological studies have consistently demonstrated the efficacy of TMP-SMX, with a likelihood of activity exceeding 90% against *S. maltophilia* [5]. This antibiotic has nearly identical breakpoints for EUCAST (> 4 mg/L) and CLSI (≥ 4 mg/L). Therefore, a difference in resistance rates according to the breakpoint used was also observed in our study, so the prevalence was 12.8% (95% CI, 8.5–18.7%) using EUCAST, but 16.2% (95% CI, 13.4–19.3%) using CLSI.

In the tetracycline group, tetracycline exhibited the highest level of antibiotic resistance (73.7%), but resistance has decreased since 2010. However, there have been few reports on determining tetracycline susceptibility in this period. Resistance to minocycline and especially tigecycline has increased compared to previous decades and there are more reports of AST. Minocycline and tigecycline are used as second-line drugs to treat *S. maltophilia* infections. These drugs exhibit extensive penetration into lung tissue and have low MICs in surveillance studies against *S. maltophilia*, with activity against approximately 70–90% of isolates [5]. The guideline issued by the IDSA suggests using an elevated dosage regimen of minocycline as the primary monotherapy agent for mild infections. In cases of TMP-SMX and levofloxacin resistance, minocycline is also often used., as resistance to them is associated with multidrug efflux pumps but does not appear to impact minocycline susceptibility [313]. This antibiotic has minimal potential for interactions with other drugs and exhibits a relatively favorable tolerability profile [4]. Our meta-analysis found the lowest resistance rate for minocycline (3.2%). Similar to ours, Dadashi et al. [312] and Banar et al. also found minocycline as the best antibiotic against *S. maltophilia* isolates. On the other hand, tigecycline for treating community-acquired bacterial pneumonia was approved by the US Food and Drug Administration (FDA) in 2005 [9]. In our meta-analysis, the resistance rate to tigecycline was higher than minocycline (21.4). In our meta-analysis, the resistance rate to tigecycline was higher than minocycline (21.4). According to our meta-analysis data, a four-fold increase in resistance to tigecycline was seen during the two periods (8.2% in 1958–2010 and 30.2% in 2011–2023). Benar et al. also confirmed a 4-fold increase in resistance to this antibiotic during the years before 2010 compared to after 2010 [311]. In the past, *S. maltophilia* was effectively treated with β-lactam drugs. However, high rates of resistance are reported for almost all of them. In the penicillin group, all of them exhibited a high level of resistance. Of these, antibiotic susceptibility testing for ticarcillin and piperacillin has been lower from 2011 to 2023. Therefore, the therapeutic guidelines do not recommend using the penicillin group for managing *S. maltophilia*.

A noteworthy increase in antibiotic resistance has been observed in some members of the cephalosporin group. Specifically, ceftriaxone and cefazolin have exhibited consistent levels of resistance over two distinct periods. The resistance rate to cefotaxim has also decreased during the two periods (86.5% in 1958–2010 and 75.9% in 2011–2023)., which may be attributed to a limited number of reports during this interval. Contrarily, despite numerous reports of susceptibility to cefoperazone, a low level of susceptibility was noted during the same period. Carbapenem resistance poses a major obstacle for healthcare providers, with levels found to be at 90% or more. Among them, antibiotic susceptibility testing to meropenem and imipenem has been reported in most studies. Meropenem resistance has been consistently high two times, with rates of 92.2% from 1958 to 2010 and 88.2% from 2011 to 2023. Our meta-analysis found the lowest resistance rate for imipenem (95.8%), although due to publication bias, according to Trim and Fill analysis, the prevalence of resistance to this antibiotic is 92.1%. Nevertheless, it is noteworthy that resistance to imipenem has declined during the aforementioned chronological intervals. In the combination of β-lactam and inhibitor group, most studies have reported the antibiotic susceptibility testing to ticarcillin/clavulanate and piperacillin/tazobactam. The overall resistance rate to piperacillin/tazobactam was 2-fold compared to ticarcillin/clavulanate. However, it is noteworthy that the resistance rate has remained constant for ticarcillin/clavulanate and a slight increase for piperacillin/tazobactam throughout the period spanning from 1958 to 2010 and from 2011 to 2023. In addition, the number of reports evaluating the susceptibility of these two antibiotics was lower after 2010 rather than before 2010.

Only ticarcillin/clavulanate, ceftazidime, and cefiderocol have MIC interpretive criteria based on the CLSI guidelines among the β-lactam agents. Previously, ticarcillin/clavulanate and ceftazidime showed favorable efficacy in treating *S. maltophilia*. Susceptibility to these two antibiotics has decreased in recent studies [3]. Our study suggests that the resistance rate to ceftazidime was 50%, and the resistance rate remained constant during two periods. Benar et al. [311]. also reported the same rate of resistance to ceftazidime as ours. The IDSA panel does not recommend the prescription of ceftazidime for managing *S. maltophilia* infections due to its ineffectiveness against *S. maltophilia* isolates, even in cases where these isolates are susceptible in vitro [5]. Furthermore, an additional concern about inactivating β-lactamases is the potential for inaccuracy and non-reproducibility of ceftazidime [303, 314]. Furthermore, an additional issue that could be associated with the existence of inactivating β-lactamases is that the MIC of ceftazidime against *S. maltophilia* may not be precise and reproducible when utilizing AST that is typically utilized by clinical microbiology laboratories. Currently, the CLSI provides the breakpoint for ceftazidime in clinical settings; however, a few older reports (7 studies) have interpreted the breakpoint for ceftazidime based on previous versions of the EUCAST. Ticarcillin/clavulanic acid exhibited a resistance rate of 31.3%, which is similar to the results conducted by Banar et al. [311]. Like ceftazidime, the resistance rates to ticarcillin/clavulanic acid did not change during two periods (*P* > 0.01). Previous investigations have demonstrated that the susceptibility rates of *S. maltophilia* to the aforementioned antibiotic during 1997–1998 ranged from 71 to 90% but dropped to 27–46.1% during 2003–2008 [4]. In this meta-analysis, the antibiotic effectivity of cefiderocol as a novel siderophore cephalosporin was determined in a few studies (7 reports) with a low prevalence but a high heterogenicity (4.7%; 95% CI: 1-78.5%). All of them were performed after 2010 with the disk diffusion method. The majority of reports were in European countries. Cefiderocol exhibits favorable activity in vitro against *S. maltophilia* because of its stability against both serine and metallo-β-lactamases, as well as demonstrated MIC90 values that were as low as 0.12–0.5 mg/L, even if the isolates displayed resistance to TMP-SMX and/or levofloxacin [279, 315]. Despite the restricted accessibility of clinical data, it has been indicated through in vitro data and animal models that there is substantial potential for the utilization of cefiderocol in treating infections caused by *S. maltophilia*. The IDSA panel suggests that, although cefiderocol monotherapy may be effective for mild infections, combining cefiderocol with another agent should be used to treat moderate and severe *S. maltophilia* infections [5]. Using fluoroquinolones, particularly levofloxacin, as a second-line therapy is often implemented when resistance to TMP-SMX or where the administration is impossible due to a life-threatening allergy or other clinical factors [8]. There have been few reports of susceptibility testing for the fluoroquinolone group except for levofloxacin and ciprofloxacin. Amongst the class of quinolone and fluoroquinolone agents, ciprofloxacin had the highest resistance rate (46%), with a constant resistance trend over two periods. Although, few studies reported antibiotic susceptibility of ciprofloxacin in recent decades. Our study shows that the resistance rate to levofloxacin was relatively low (16%), although due to publication bias, the prevalence of resistance to this antibiotic is 20.7% according to trim and fill analysis. Banar et al. revealed a global resistance rate to levofloxacin exceeding our findings at 19.7% in contrast to our 17.7%. They showed a significant difference in the prevalence of resistance between the different regions [311]. On the other hand, Dadashi et al. highlighted a global prevalence of resistance to levofloxacin lower than ours (14.4% vs. 17.7%) due to the fewer included studies. The prevalence of resistance to this drug has decreased in two recent decades. The restricted application of this antibiotic in recent times can be attributed to the likelihood of resistance development during therapy [312]. This is particularly relevant for patients with cystic fibrosis or cirrhosis, who commonly experience frequent or chronic quinolone exposure [8]. Within the aminoglycoside group, every compound exhibited significant degrees of resistance. However, their use in antibiotic susceptibility testing declined during the 2011 to 2023 period due to their inefficiency. The overall resistance rate to chloramphenicol was relatively low (29.2). The frequency of resistance to this medication has experienced a decrease in recent years. The utilization of chloramphenicol in clinical settings is restricted due to its possible adverse effects, such as bone marrow suppression or induction of aplastic anemia [8].

In the present study, the overall resistance rate to colistin was relatively high (46.6%). Colistin treatment provides a rescue therapy for various multidrug-resistant (MDR) Gram-negative infections. However, its application is restricted by its notable nephrotoxicity and the emergence of more advanced, efficacious, and less toxic antimicrobial agents [8]. An increased incidence of colistin-resistant isolates has also been observed in recent years. (31.9% from 1958 to 2010 vs. 44% from 2011 to 2023). Similar to these results, Rodríguez et al. [316]. showed that colistin resistance elevated from 8% in 1996 to 45% in 2013 due to the significant increase (11.4-fold) of colistin usage during the study period. Drug susceptibility testing for *Stenotrophomonas* spp is obstructed by its diverse mechanisms of drug resistance [8]. Notably, no established CLSI susceptibility criteria exist for any of the polymyxins.

An increasingly common clinical challenge associated with this pathogen is heterogeneous resistance to colistin, whereby distinct subpopulations within a single isolate display different susceptibilities to the antibiotic [316]. There are also challenges with the accuracy and repeatability of polymyxin MICs. Therefore, the IDSA panel recommends avoiding polymyxins for *S. maltophilia* infections [5]. There are some limitations to our study. First, several studies did not use specific guidelines or report the exact resistance rate. Therefore, the rate of antibiotic resistance may have been affected by these studies not being included in the meta-analysis. Second, the full text of several published studies was not available despite communicating with the corresponding authors by sending several e-mails, and only a few of them responded. Third, certain studies assessed susceptibility rates solely based on MIC50/90 without reporting prevalence. Therefore, these studies, which may have influenced the pooled prevalence of antimicrobial resistance, were excluded from the meta-analysis.

## Conclusion

According to our meta-analysis, due to the low rates of resistance to minocycline and cefiderocol, these two antibiotics can be suggested as the preferred therapeutic options for treating most if not all infections caused by *S. maltophilia*. TMP-SMX, as a first-choice drug of *S. maltophilia*, indicated the low rates of resistance worldwide. Hence, it seems that this drug is still an effective therapeutic option. Also, due to the high-frequency resistance to β-lactams (except cefiderocol), especially carbapenems and aminoglycosides, in the last two decades, these antibiotic groups should not be recommended in therapeutic guidelines, especially as monotherapy. On the other hand, the prevalence rates of antimicrobial resistance in *S. maltophilia* in the African continent are limited by the few numbers of studies. Hence, a regular monitoring and surveillance program should be carried out to determine the antibiotic sensitivity of this bacterium across this continent.

### Electronic supplementary material

Below is the link to the electronic supplementary material.

Supplementary Material 1: The supplementary material included the Supplementary File 1, Supplementary Figure’s file, Supplementary Table S1 and S2

Supplementary Material 2

Supplementary Material 3

Supplementary Material 4

## Data Availability

All data generated or analysed during this study are included in this published article [and its supplementary information files].

## Abbreviations

TMP-SMX: Trimethoprim-sulfamethoxazole
RND: Resistance Nodulation Division
PRISMA: Preferred Reporting Items for Systematic Reviews and Meta-Analyses
NOS: Newcastle-Ottawa scale
CI: confidence interval
AST: antimicrobial susceptibility testing
CLSI: Clinical & Laboratory Standards Institute
EUCAST: The European Committee on Antimicrobial Susceptibility Testing
FDA: US Food and Drug Administration
MDR: multidrug-resistant
IDSA: Infectious Diseases Society of America
