# Supplementary material for: Global mapping of antibiotic resistance rates among clinical isolates of Stenotrophomonas maltophilia: a systematic review and meta-analysis
Source: Ann Clin Microbiol Antimicrob. 2024 Mar 19;23:26. doi: 10.1186/s12941-024-00685-4 (PMC10953290; doi:10.1186/s12941-024-00685-4)
Supplement: Supplementary file 1 — Supplementary Material 1: The supplementary material included the Supplementary File 1, Supplementary Figure’s file, Supplementary Table S1 and S2 [file 12941_2024_685_MOESM1_ESM.docx]

(“*Stenotrophomonas maltophilia*” OR “*S. maltophilia*” ) AND (“antimicrobial-drug resistance” OR “drug resistance” OR “antibiotic resistance” OR “antimicrobial activity” OR “Trimethoprim-Sulfamethoxazole” OR “TMP-SMX” OR “co-trimoxazole” OR “sulfamethoxazole” OR “Fluoroquinolones” OR “levofloxacin” OR “Minocycline” OR “Beta-Lactam” OR “ceftazidime” OR “Cefiderocol” OR “cefepime” OR “Colistin” OR “Polymyxin” OR “relebactam-imipenem” OR “ceftazidime-avibactam” OR “Ceftolozane-tazobactam” OR “Avibactam-Aztreonam” OR “aminoglycosides” OR “Plazomicin” OR “Eravacycline” OR “Tigecycline” OR “tetracycline” OR “ciprofloxacin” OR “doxycycline” OR “macrolides” OR “Chloramphenicol” OR “Ticarcillin-clavulanate”)

PUBMED:

May 31, 2023

Result: 1,843 results

((Stenotrophomonas maltophilia) OR (S. maltophilia)) AND (((((((((((((((((((((((((((((antimicrobial-drug resistance OR drug resistance OR antibiotic resistance OR antimicrobial activity) ) OR (Trimethoprim-Sulfamethoxazole)) OR ((TMP-SMX))) OR (co-trimoxazole)) OR (sulfamethoxazole)) OR (Fluoroquinolones)) OR (levofloxacin)) OR (Minocycline)) OR (Beta-Lactam)) OR (ceftazidime)) OR (Cefiderocol)) OR (cefepime)) OR (Colistin)) OR (Polymyxin)) OR (relebactam-imipenem)) OR (ceftazidime-avibactam)) OR (Ceftolozane-tazobactam)) OR (Avibactam-Aztreonam)) OR (aminoglycosides)) OR (Plazomicin)) OR (Eravacycline)) OR (Tigecycline)) OR (tetracycline)) OR (ciprofloxacin)) OR (doxycycline)) OR (macrolides)) OR (Chloramphenicol)) OR (Ticarcillin-clavulanate))

Embase

May 31, 2023

Result: 4,185

‘Stenotrophomonas maltophilia’ OR ‘S. maltophilia’ AND ‘antimicrobial-drug resistance’ OR ‘drug resistance’ OR ‘antibiotic resistance’ OR ‘antimicrobial activity’ OR ‘Trimethoprim-Sulfamethoxazole’ OR ‘TMP-SMX’ OR ‘co-trimoxazole’ OR ‘sulfamethoxazole’ OR ‘Fluoroquinolones’ OR ‘levofloxacin’ OR ‘Minocycline’ OR ‘Beta-Lactam’ OR ‘ceftazidime’ OR ‘Cefiderocol’ OR ‘cefepime’ OR ‘Colistin’ OR ‘Polymyxin’ OR ‘relebactam-imipenem’ OR ‘ceftazidime-avibactam’ OR ‘Ceftolozane-tazobactam’ OR ‘Avibactam-Aztreonam’ OR ‘aminoglycosides’ OR ‘Plazomicin’ OR ‘Eravacycline’ OR ‘Tigecycline’ OR ‘tetracycline’ OR ‘ciprofloxacin’ OR ‘doxycycline’ OR ‘macrolides’ OR ‘Chloramphenicol’ OR ‘Ticarcillin-clavulanate’

WOS

May 31, 2023

Result: 1441

“Stenotrophomonas maltophilia” OR “S. maltophilia” AND “antimicrobial-drug resistance” OR “drug resistance” OR “antibiotic resistance” OR “antimicrobial activity” OR “Trimethoprim-Sulfamethoxazole” OR “TMP-SMX” OR “co-trimoxazole” OR “sulfamethoxazole” OR “Fluoroquinolones” OR “levofloxacin” OR “Minocycline” OR “Beta-Lactam” OR “ceftazidime” OR “Cefiderocol” OR “cefepime” OR “Colistin” OR “Polymyxin” OR “relebactam-imipenem” OR “ceftazidime-avibactam” OR “Ceftolozane-tazobactam” OR “Avibactam-Aztreonam” OR “aminoglycosides” OR “Plazomicin” OR “Eravacycline” OR “Tigecycline” OR “tetracycline” OR “ciprofloxacin” OR “doxycycline” OR “macrolides” OR “Chloramphenicol” OR “Ticarcillin-clavulanate”

**Scopus**

May 31, 2023

**result: 3,961**

TITLE-ABS-KEY(“Stenotrophomonas maltophilia” OR “S. maltophilia”)

TITLE-ABS-KEY(“antimicrobial-drug resistance” OR “drug resistance” OR “antibiotic resistance” OR “antimicrobial activity” OR “Trimethoprim-Sulfamethoxazole” OR “TMP-SMX” OR “co-trimoxazole” OR “sulfamethoxazole” OR “Fluoroquinolones” OR “levofloxacin” OR “Minocycline” OR “Beta-Lactam” OR “ceftazidime” OR “Cefiderocol” OR “cefepime” OR “Colistin” OR “Polymyxin” OR “relebactam-imipenem” OR “ceftazidime-avibactam” OR “Ceftolozane-tazobactam” OR “Avibactam-Aztreonam” OR “aminoglycosides” OR “Plazomicin” OR “Eravacycline” OR “Tigecycline” OR “tetracycline” OR “ciprofloxacin” OR “doxycycline” OR “macrolides” OR “Chloramphenicol” OR “Ticarcillin-clavulanate”)
