# Supplementary material for: Global mapping of antibiotic resistance rates among clinical isolates of Stenotrophomonas maltophilia: a systematic review and meta-analysis
Source: Ann Clin Microbiol Antimicrob. 2024 Mar 19;23:26. doi: 10.1186/s12941-024-00685-4 (PMC10953290; doi:10.1186/s12941-024-00685-4)
Supplement: Supplementary file 2 — Supplementary Material 2 [file 12941_2024_685_MOESM2_ESM.docx]

**List of Contents**

The funnel plots and meta-regression of the Penicillins group

The funnel plot and meta-regression of the Monobactam

The funnel plot and meta-regression of the Fosfomycin

The funnel plots and meta-regression of the β-lactam combination agent

The funnel plots and meta-regression of the Carbapenems

The funnel plots and meta-regression of the Cephalosporins

The funnel plots and meta-regression of the Aminoglycosides

The funnel plots and meta-regression of the Tetracyclines

The funnel plots and meta-regression of the Fluoroquinolones

The funnel plots and meta-regression of the Sulfonamides

The funnel plot and meta-regression of the Colistin

The funnel plot meta-regression of the Chloramphenicol

The funnel plot and meta-regression of the Rifampin


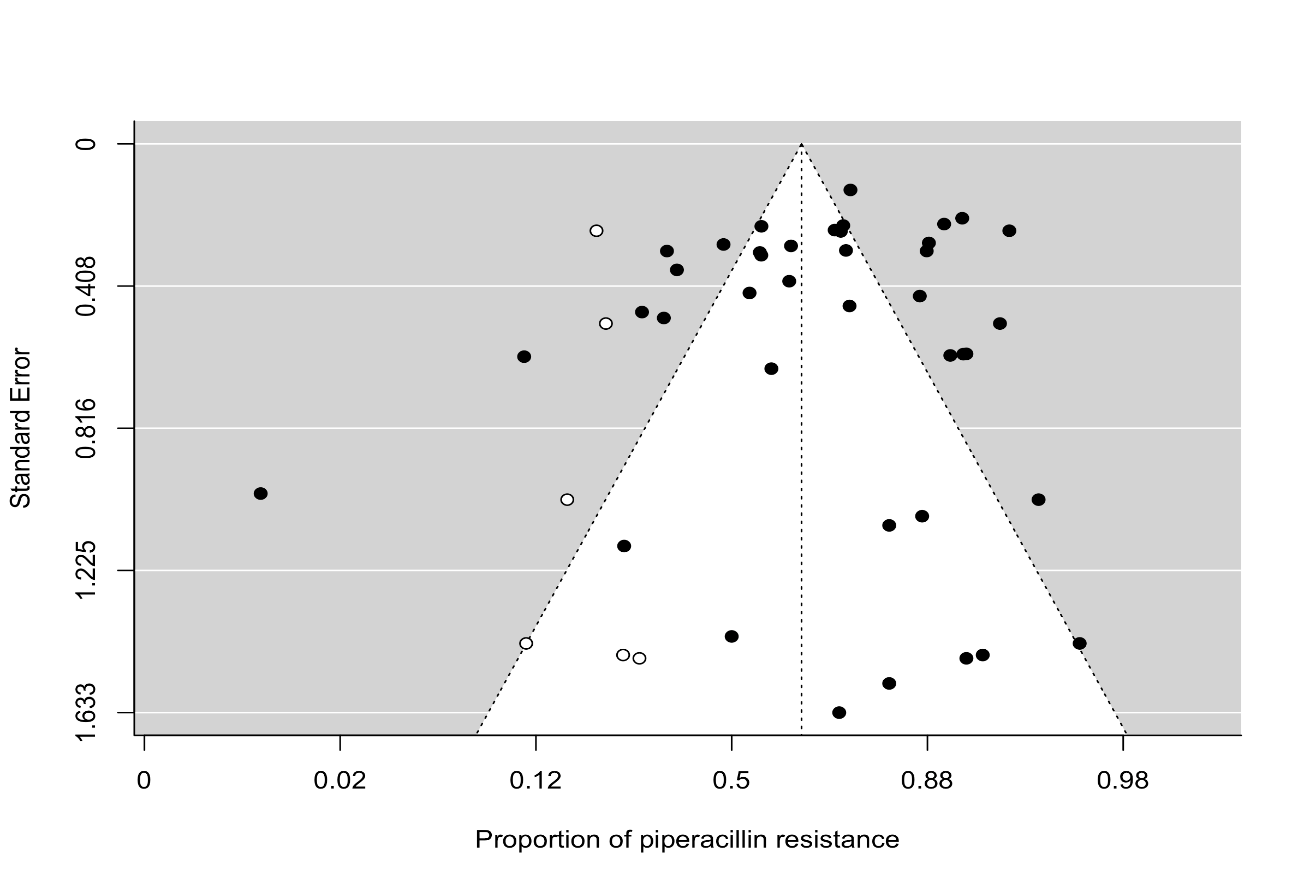
Figure 1. The funnel plot of meta-analysis of publication studies. Each black dot represents a study. The white dots represent missing studies. The black line in the middle represents the average effect size.

**Penicillin group**


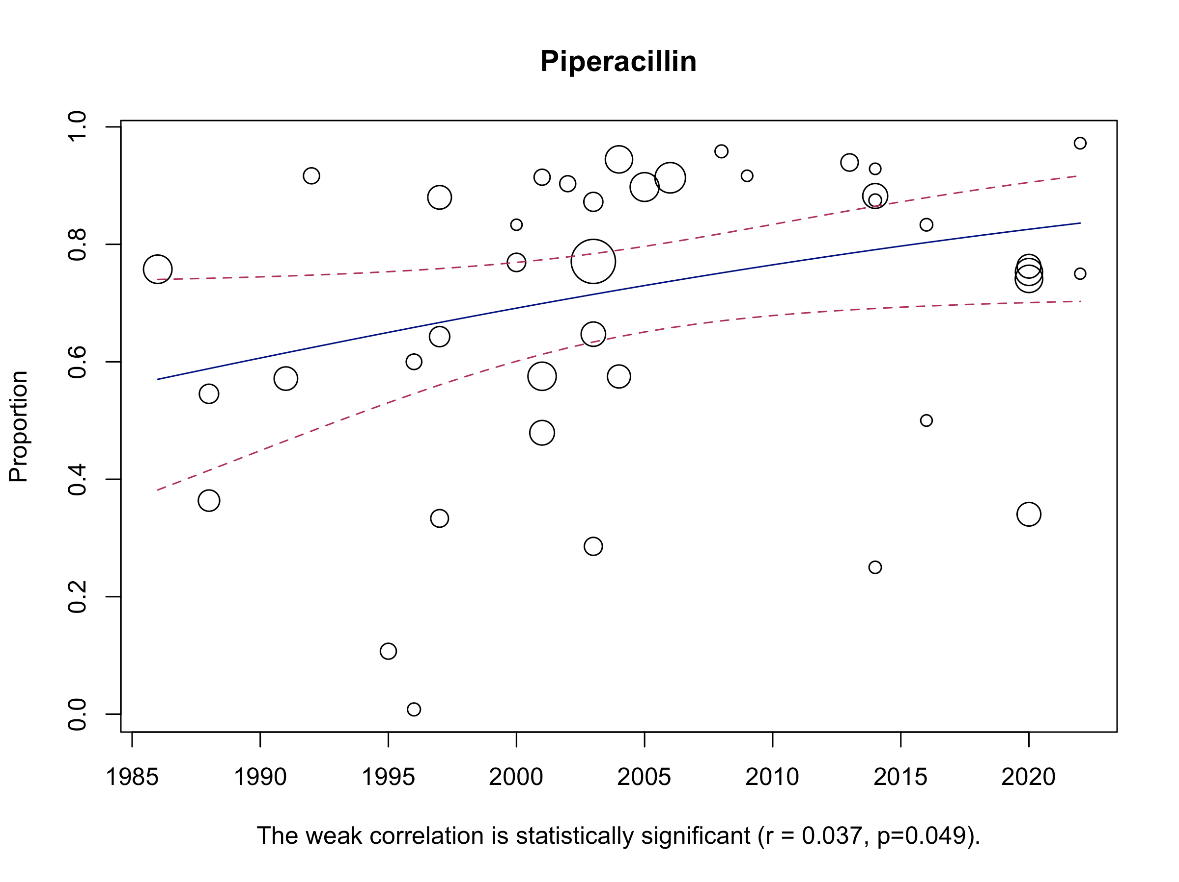


Figure 2. Bubble plot with fitted meta-regression for the year of publication.


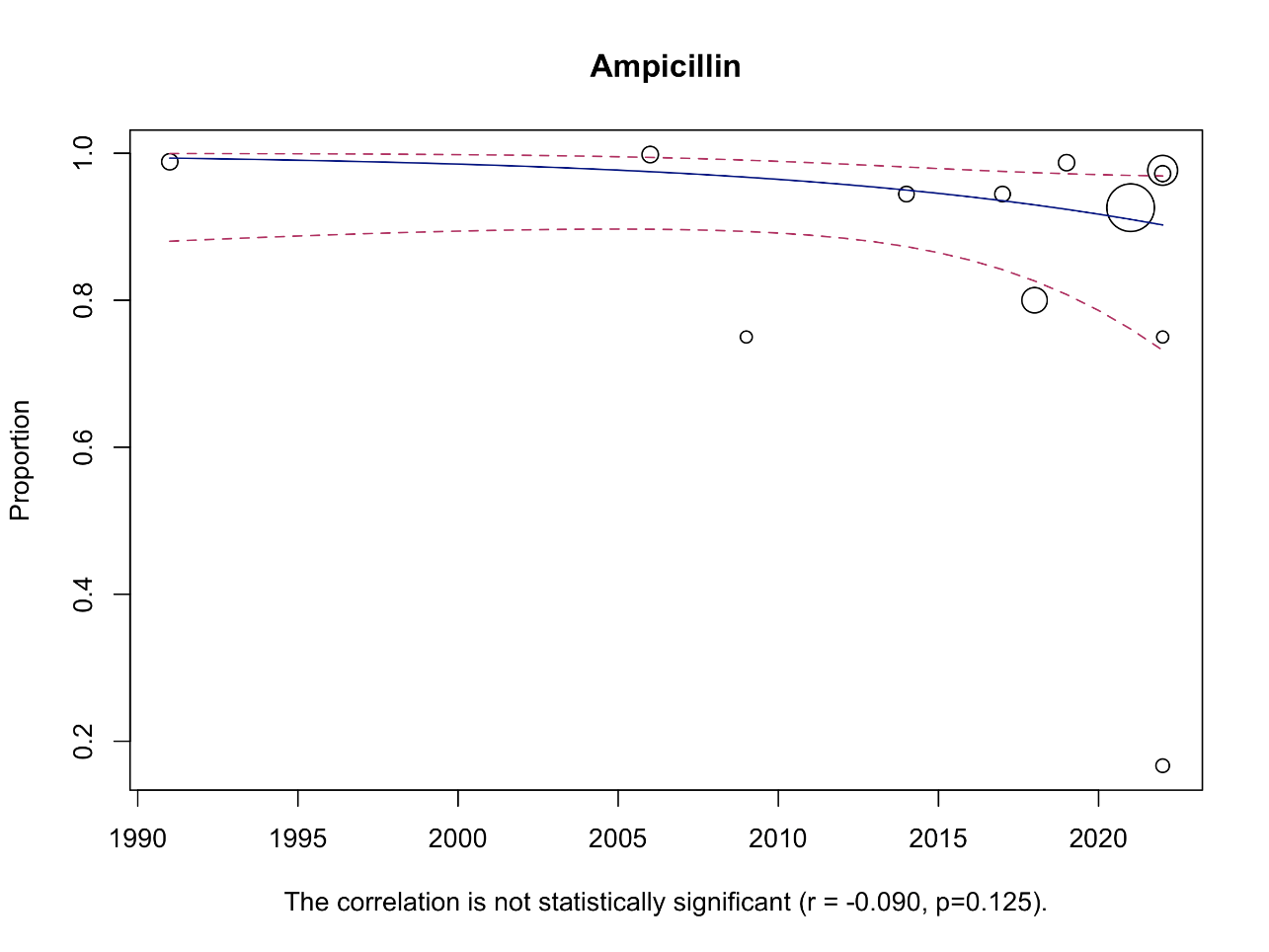

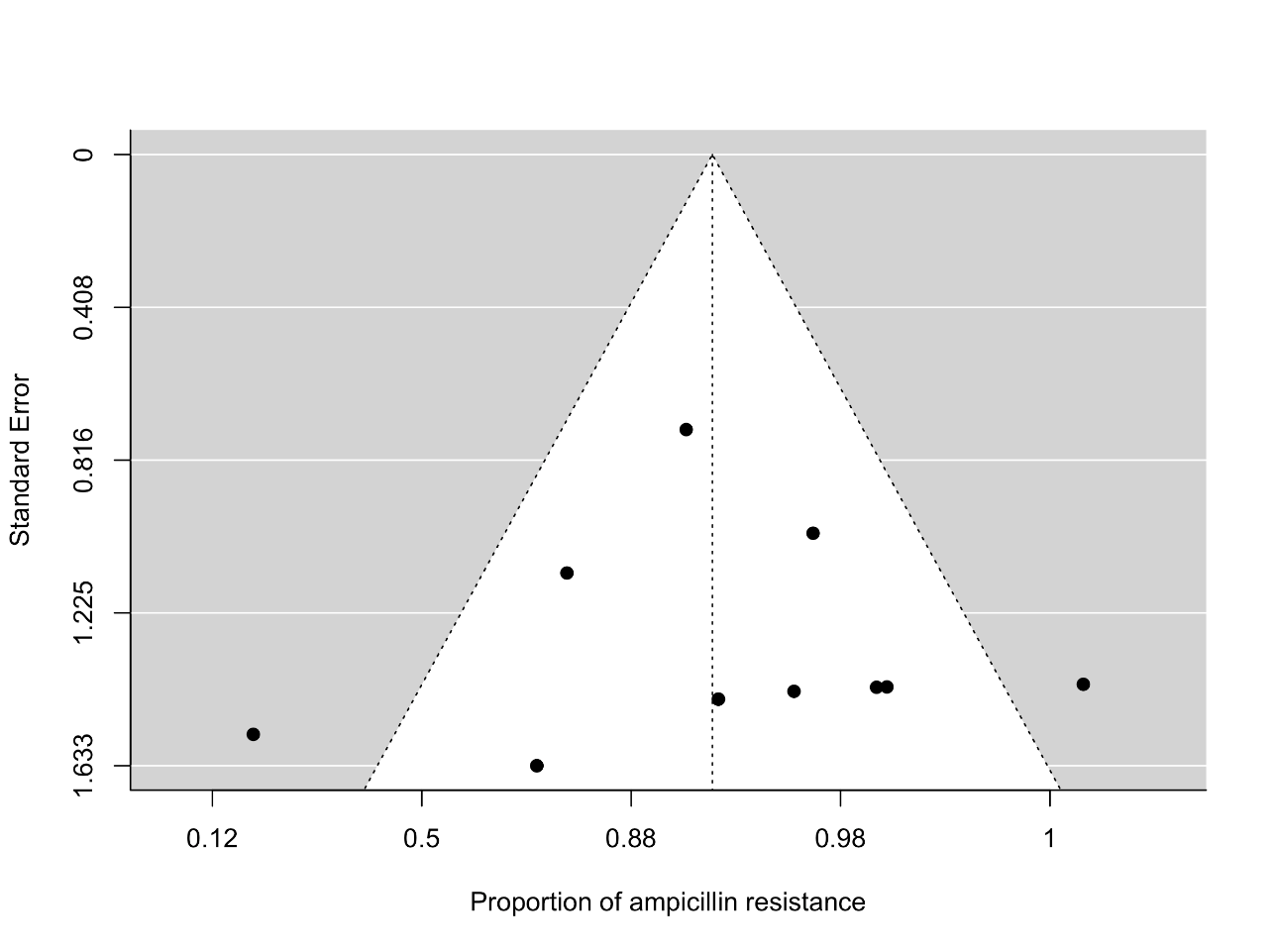
Figure 3. The funnel plot of meta-analysis of publication studies. Each black dot represents a study. The black line in the middle represents the average effect size.

Figure 4. Bubble plot with fitted meta-regression for the year of publication.


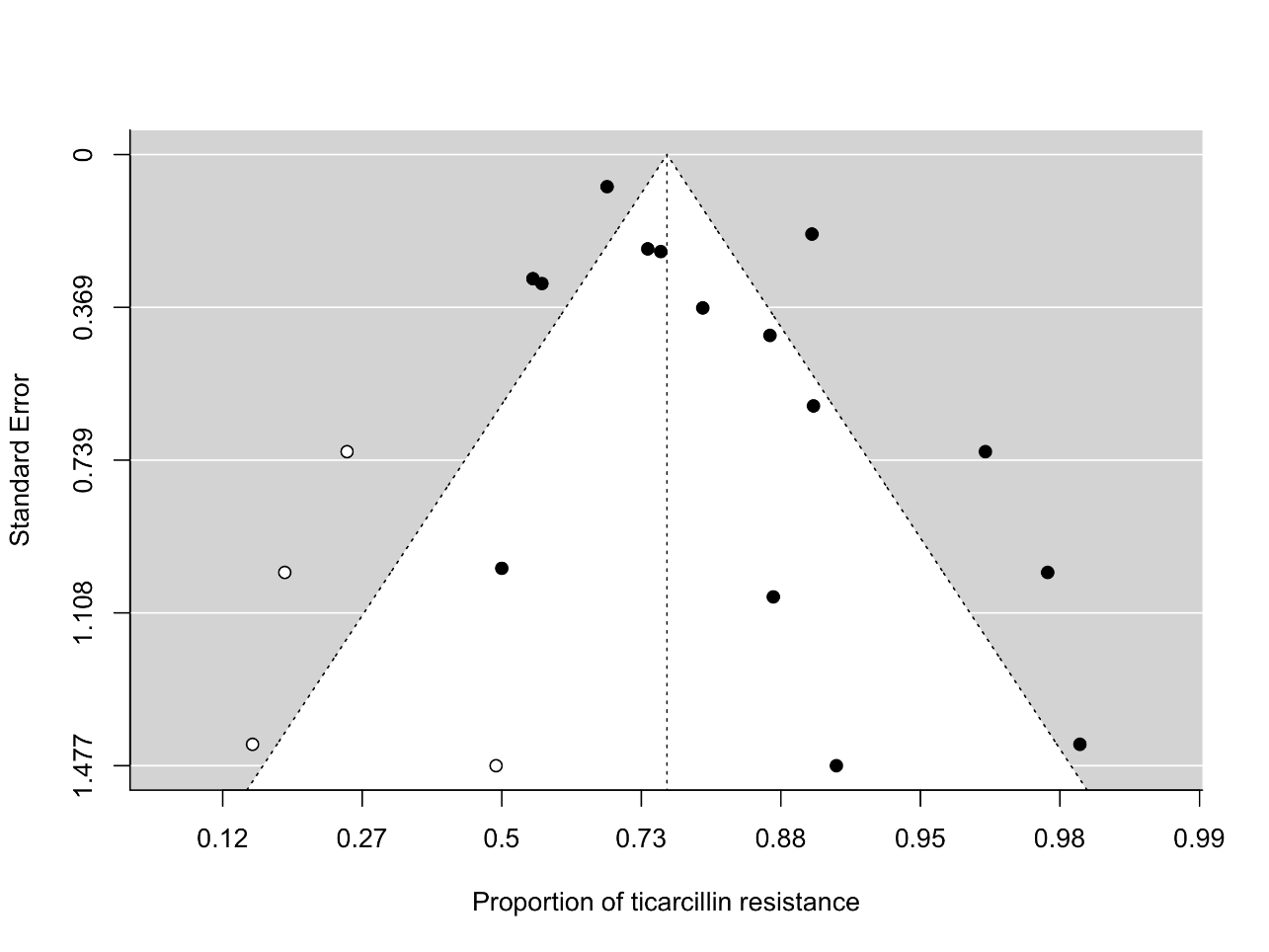


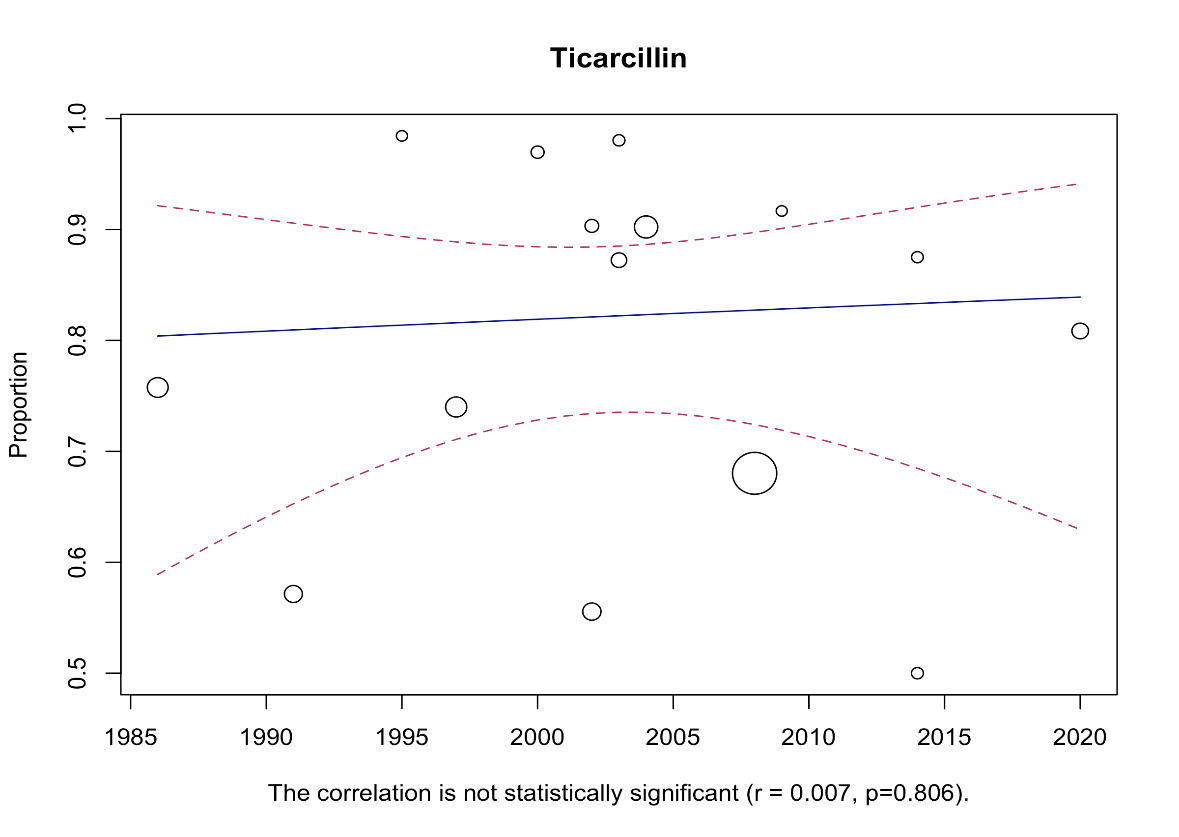
Figure 5. Bubble plot with fitted meta-regression for the year of publication.

Figure 6. The funnel plot of meta-analysis of publication studies. Each black dot represents a study. The white dots represent missing studies. The black line in the middle represents the average effect size.


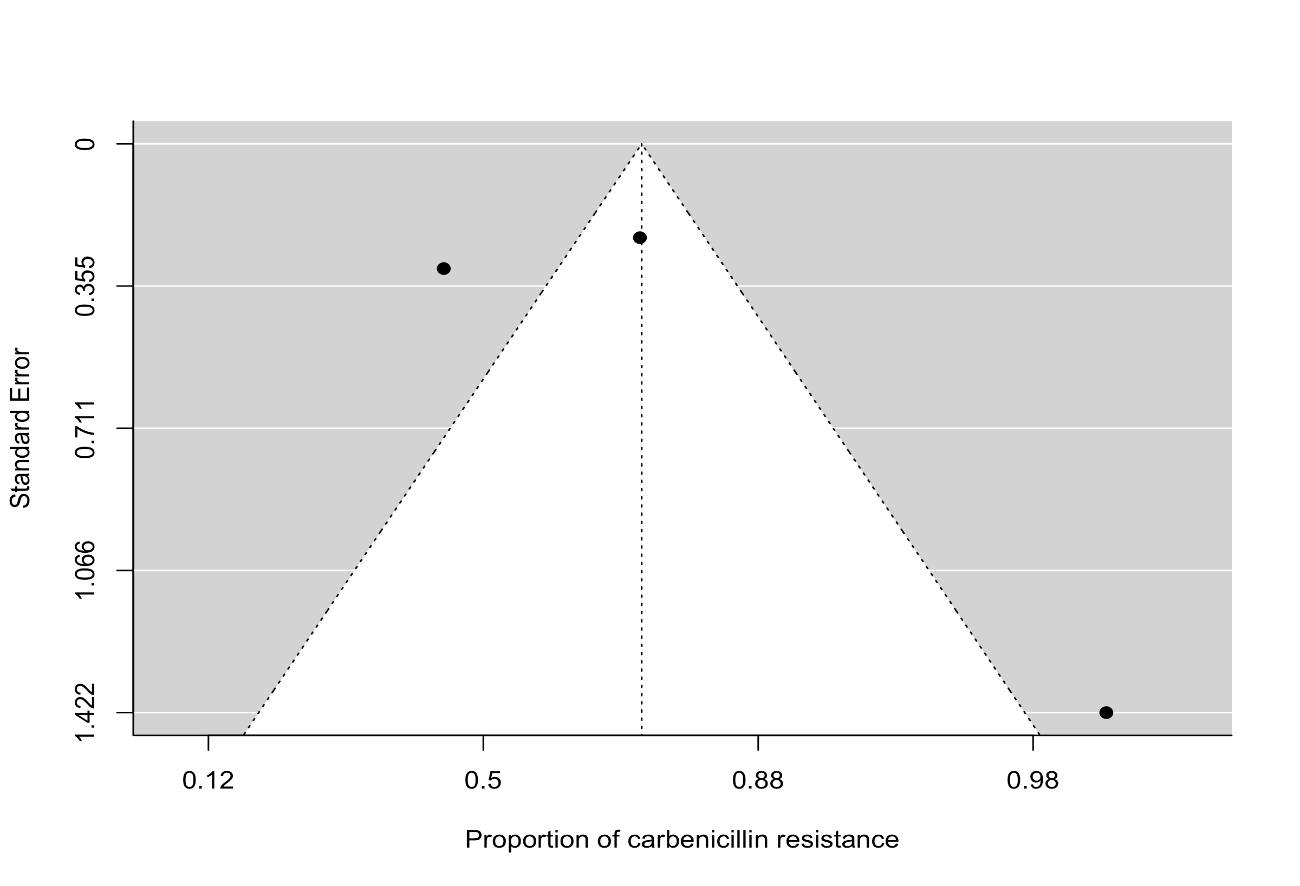
Figure 7. The funnel plot of meta-analysis of publication studies. Each black dot represents a study. The black line in the middle represents the average effect size


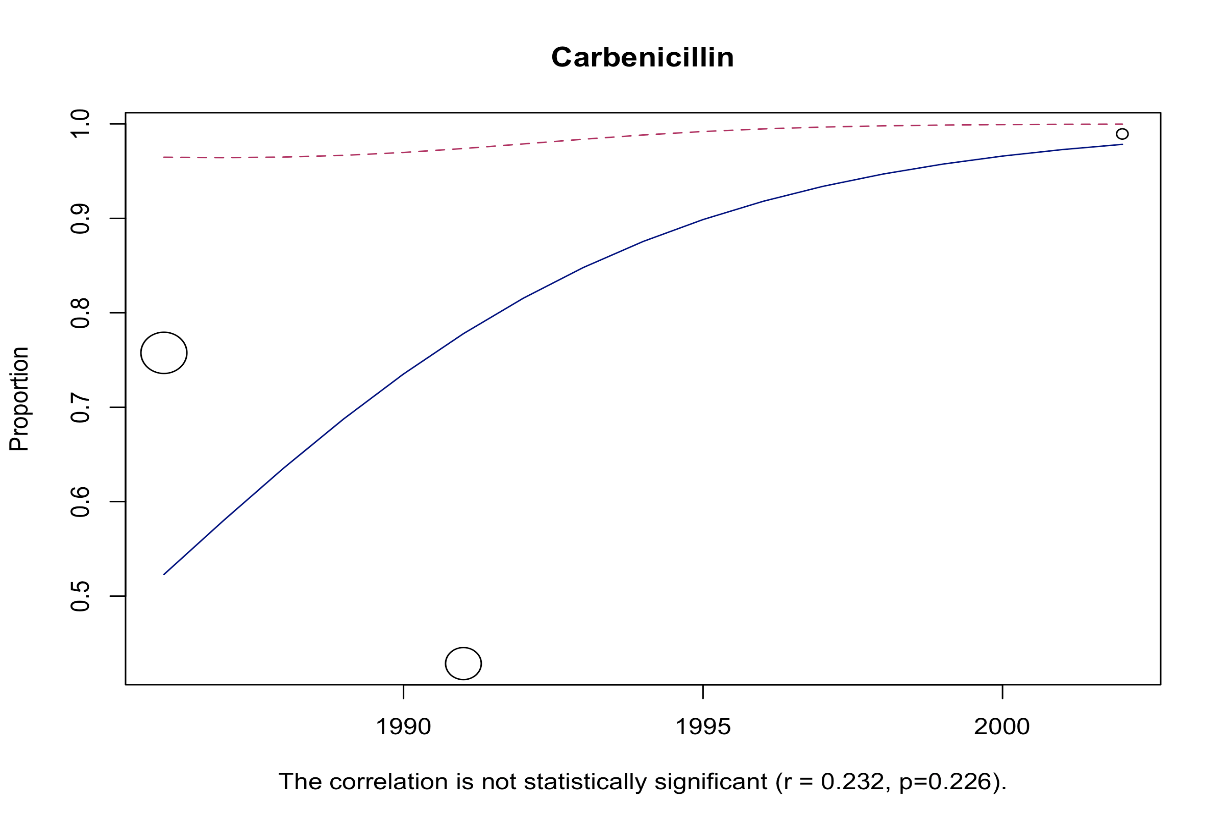


Figure 8. Bubble plot with fitted meta-regression for the year of publication.


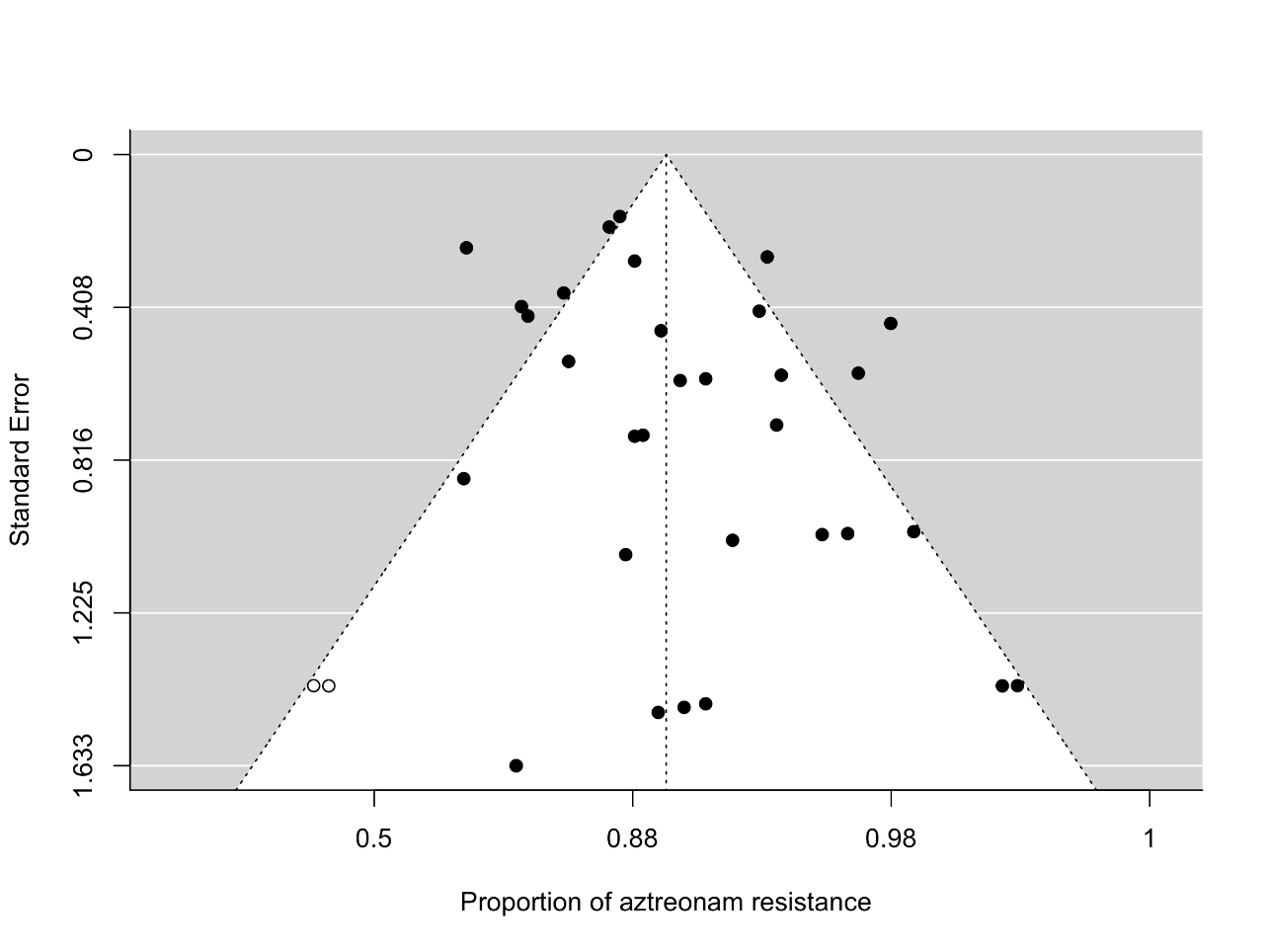


**Monobactam**


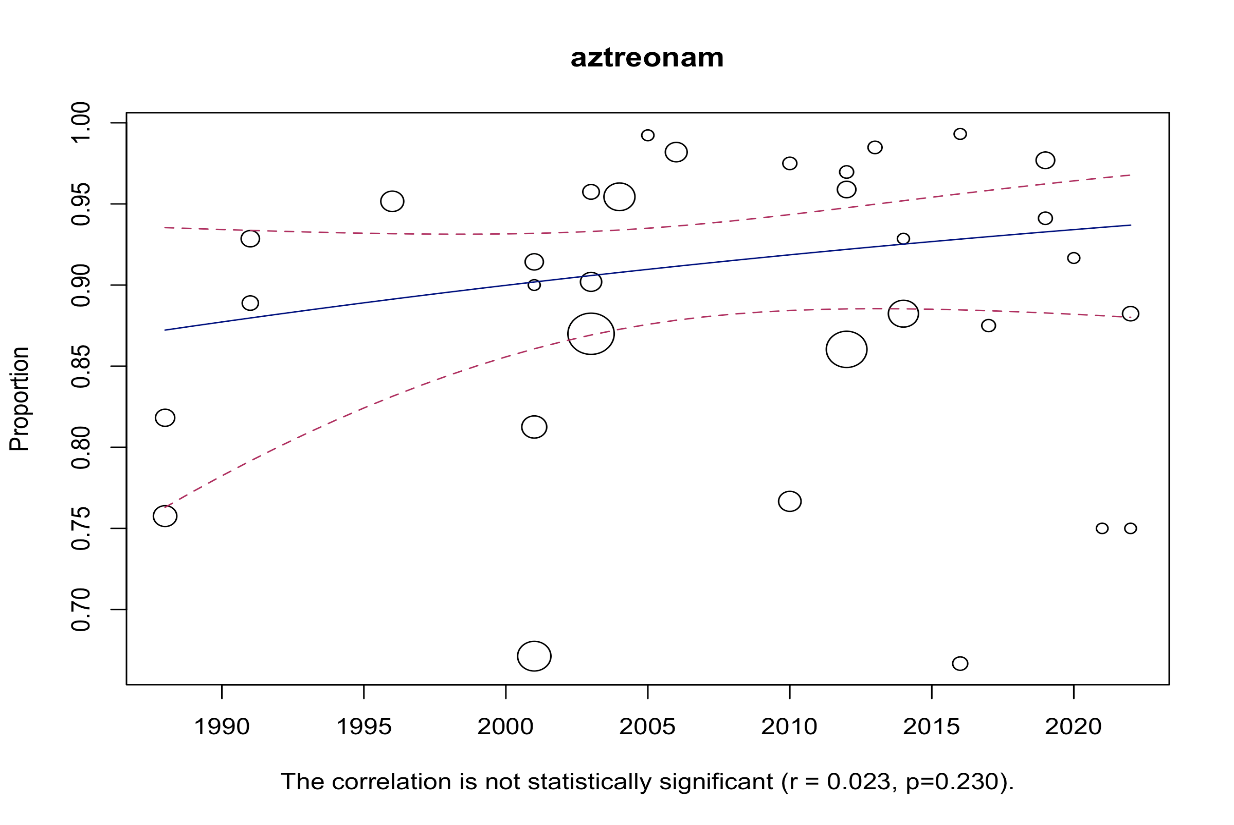
Figure 9. The funnel plot of meta-analysis of publication studies. Each black dot represents a study. The white dots represent missing studies. The black line in the middle represents the average effect size.

Figure 10. Bubble plot with fitted meta-regression for the year of publication.

**Fosfomycin**


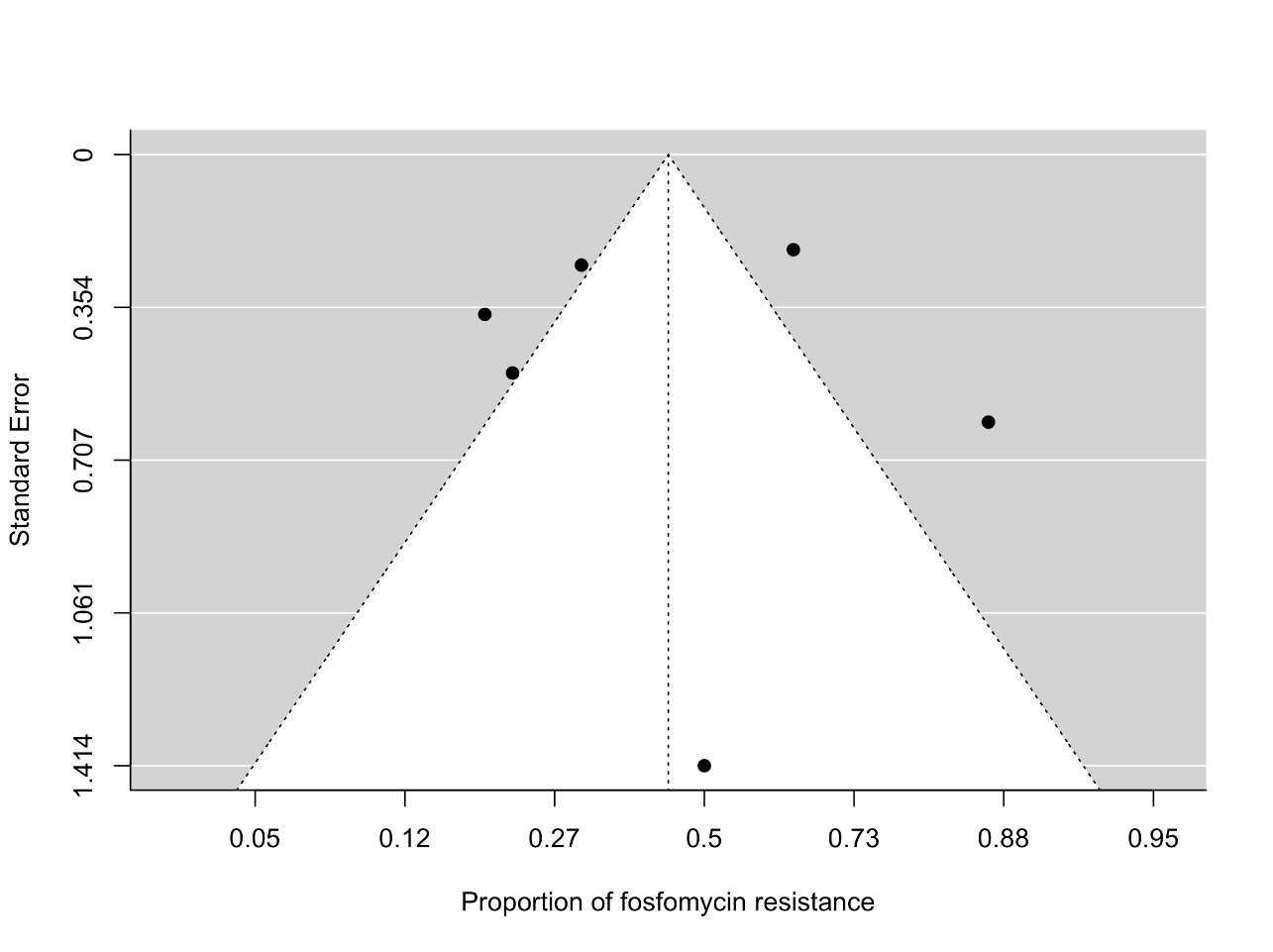


Figure 11. The funnel plot of meta-analysis of publication studies. Each black dot represents a study. The white dots represent missing studies. The black line in the middle represents the average effect size.


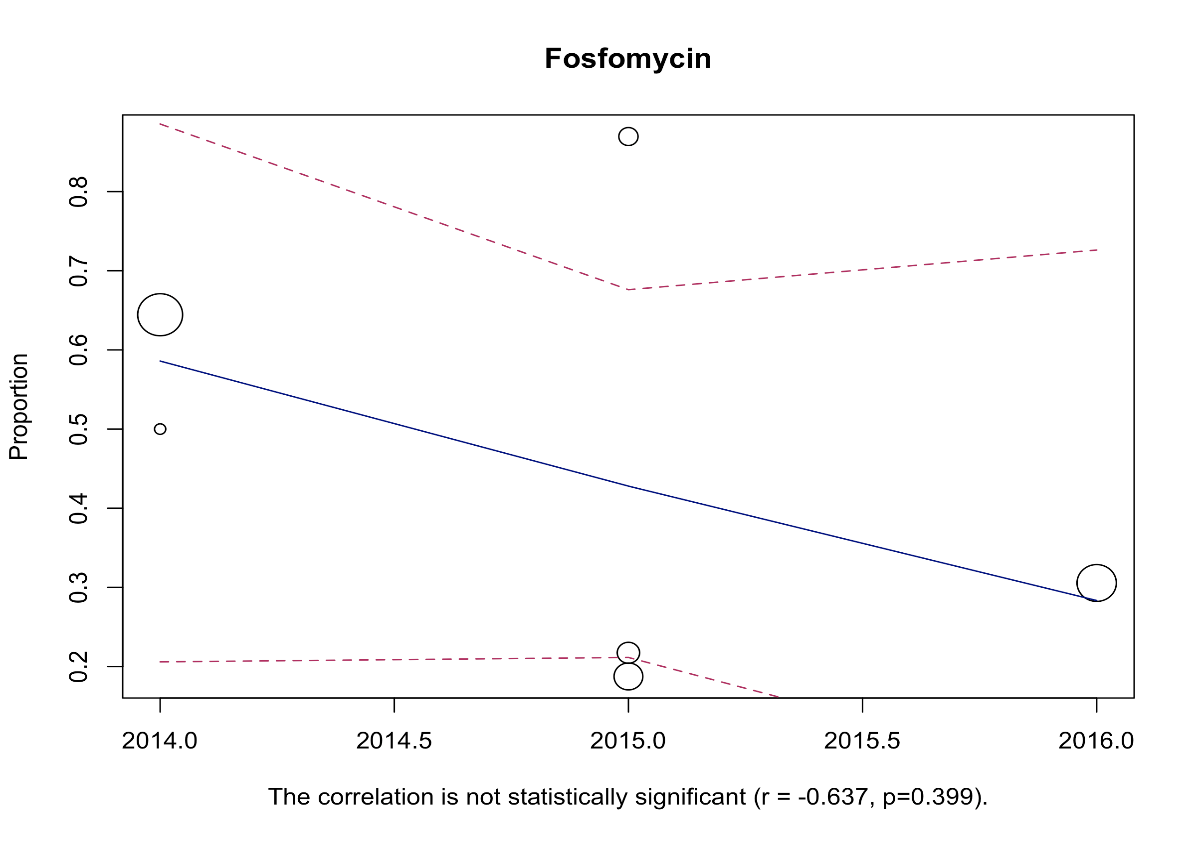


Figure 12. Bubble plot with fitted meta-regression for the year of publication.


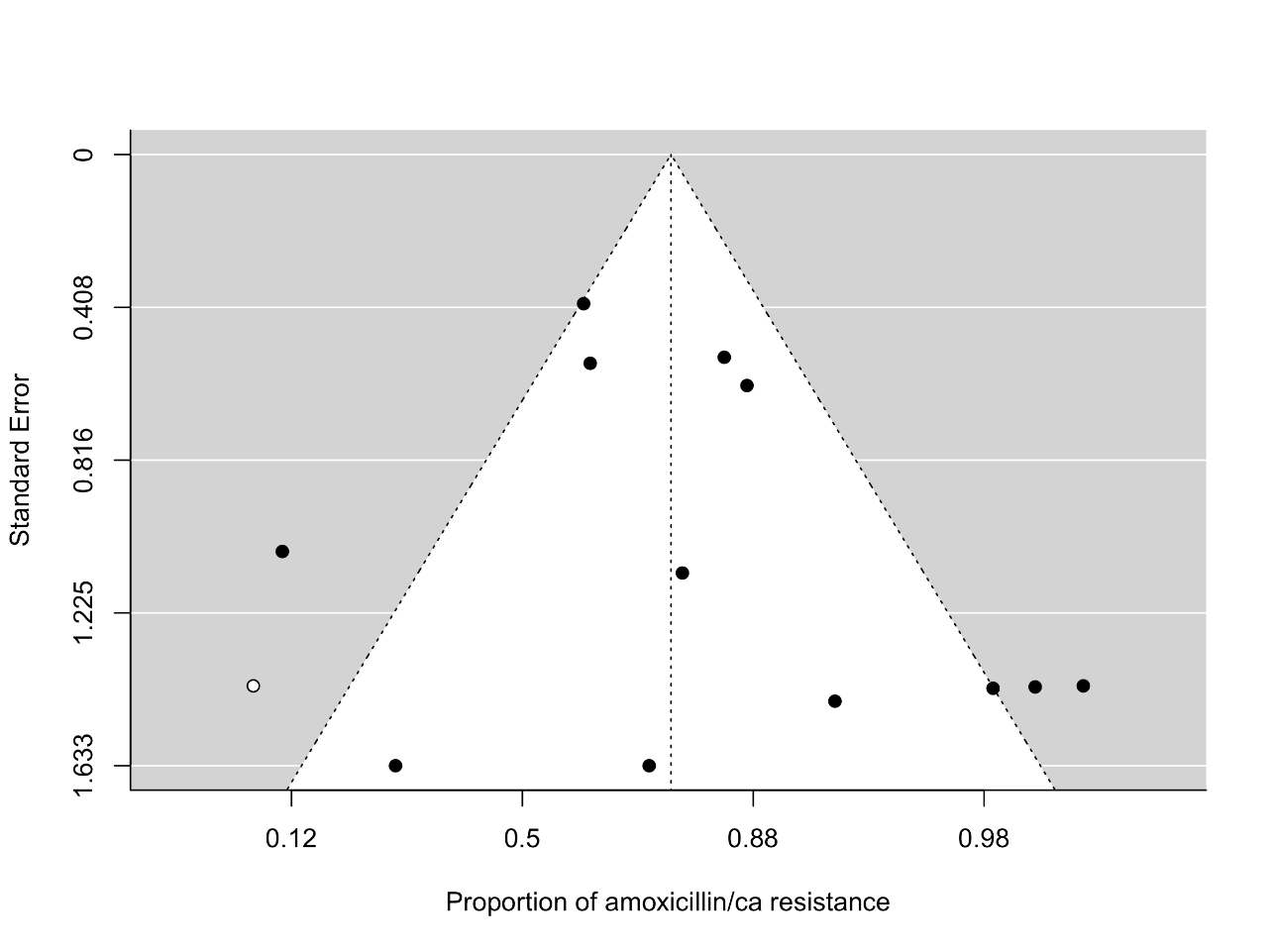


**β-lactam combination agent**


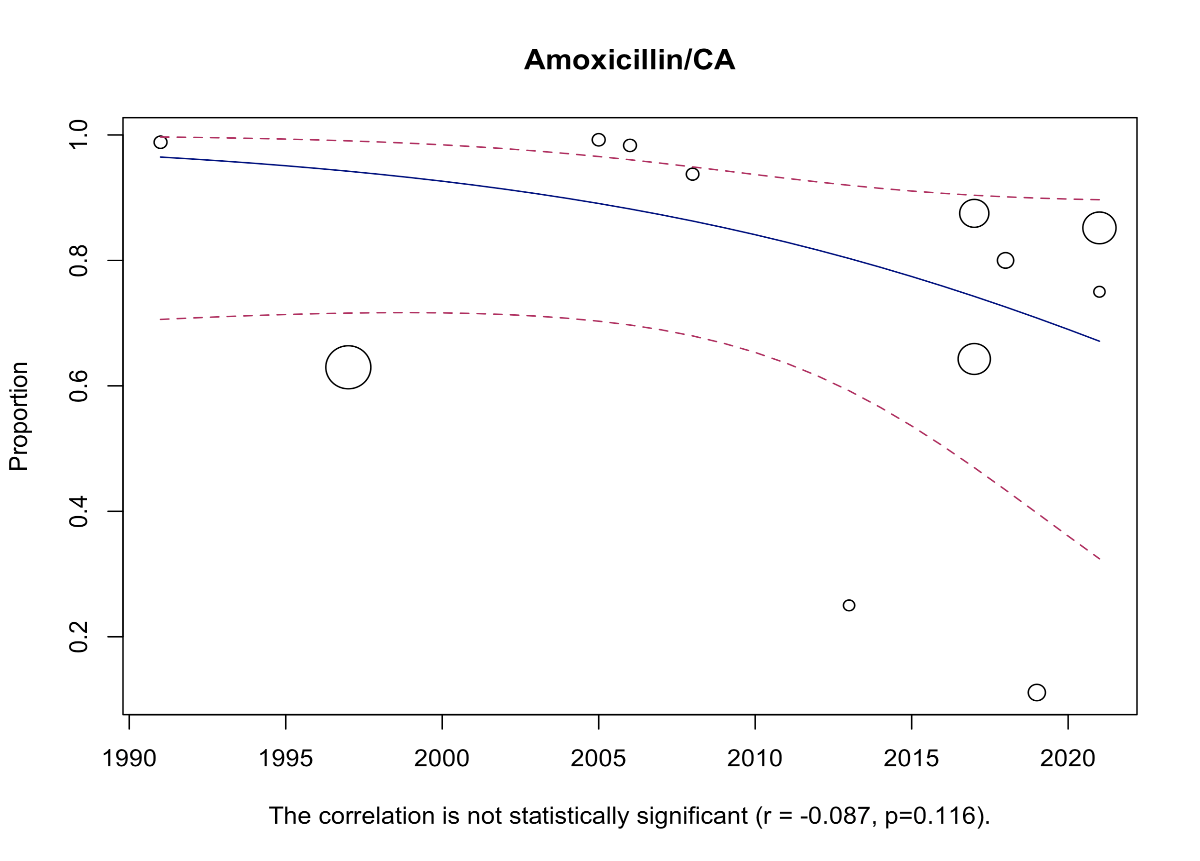
Figure 13. The funnel plot of meta-analysis of publication studies. Each black dot represents a study. The white dots represent missing studies. The black line in the middle represents the average effect size.

Figure 14. Bubble plot with fitted meta-regression for the year of publication.
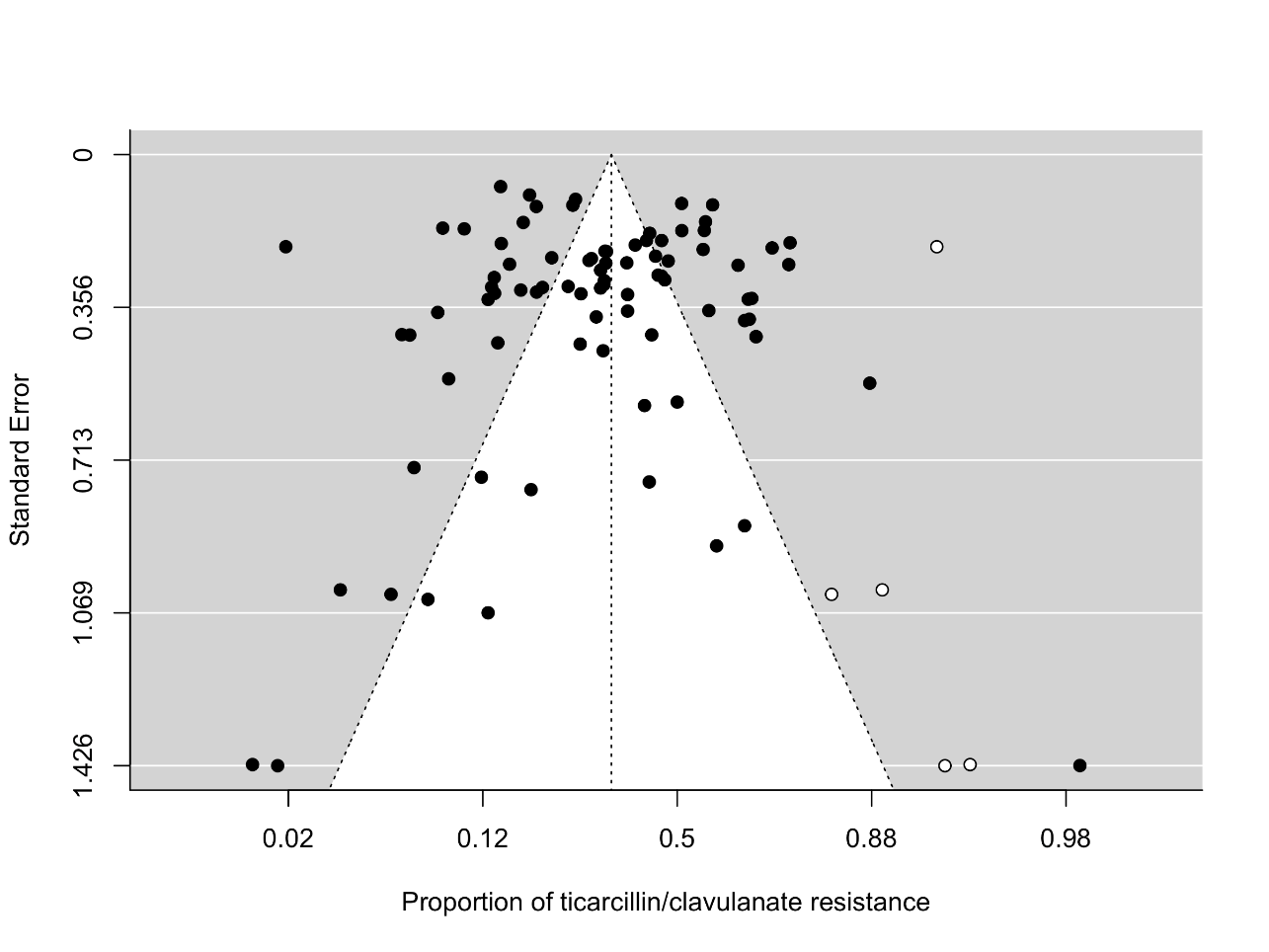
Figure 15. The funnel plot of meta-analysis of publication studies. Each black dot represents a study. The white dots represent missing studies. The black line in the middle represents the average effect size.


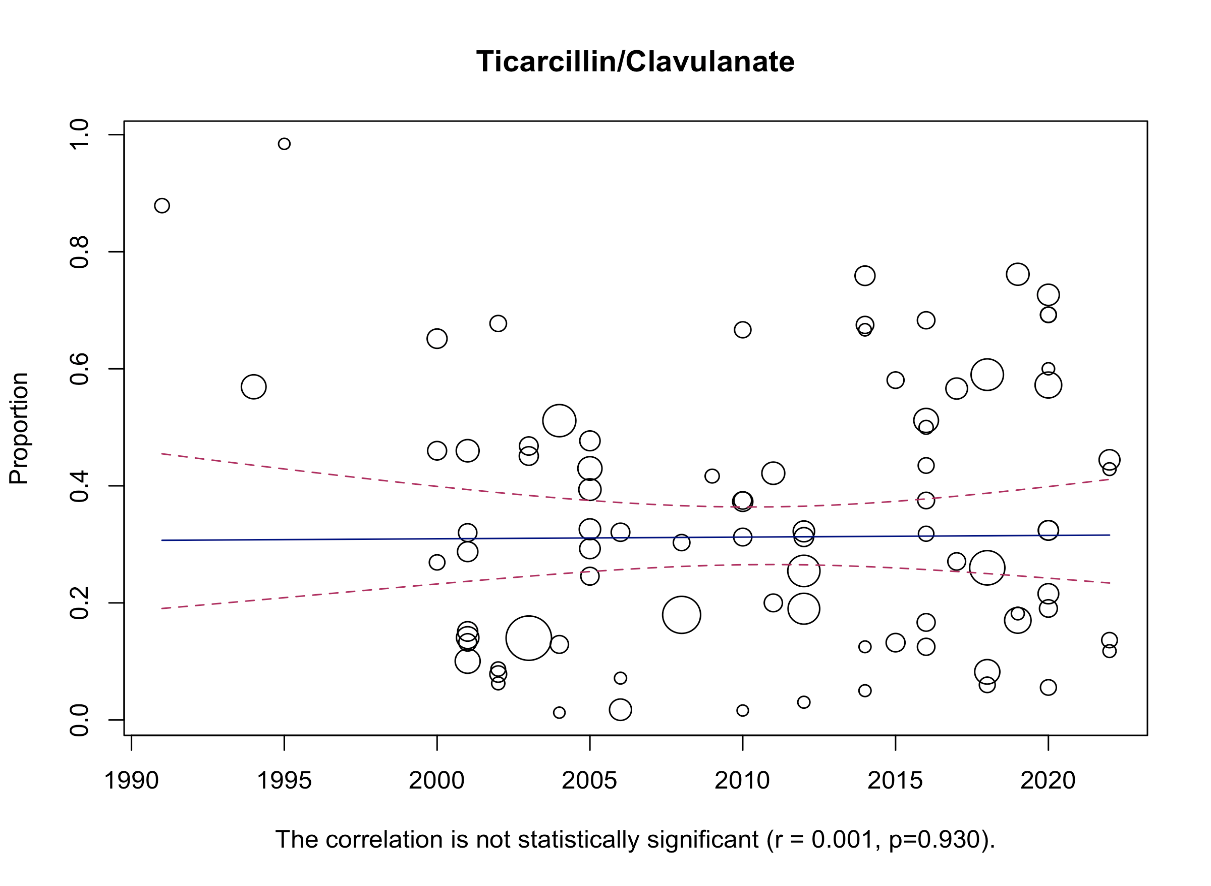


Figure 16. Bubble plot with fitted meta-regression for the year of publication.


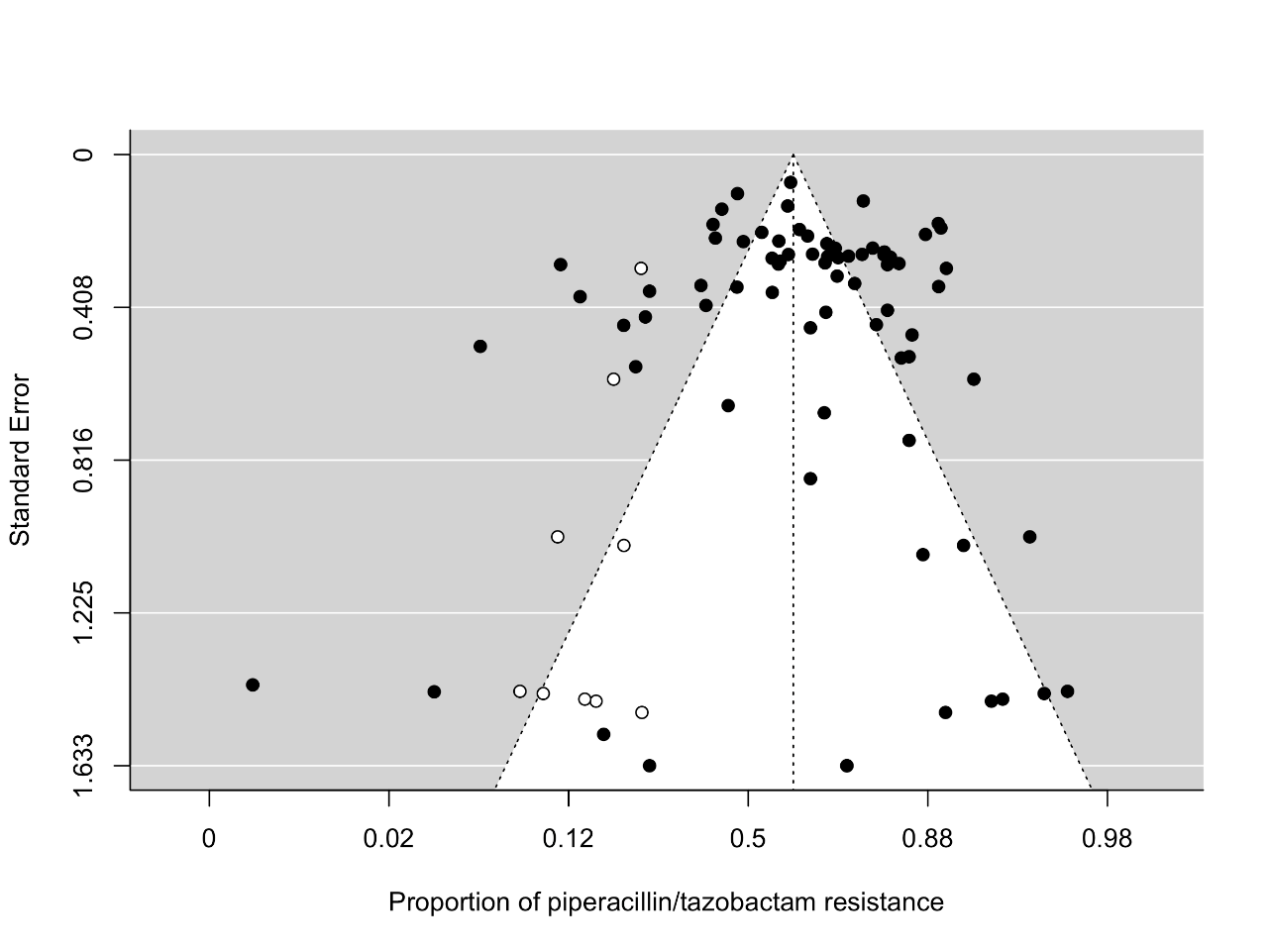


Figure 17. The funnel plot of meta-analysis of publication studies. Each black dot represents a study. The white dots represent missing studies. The black line in the middle represents the average effect size.


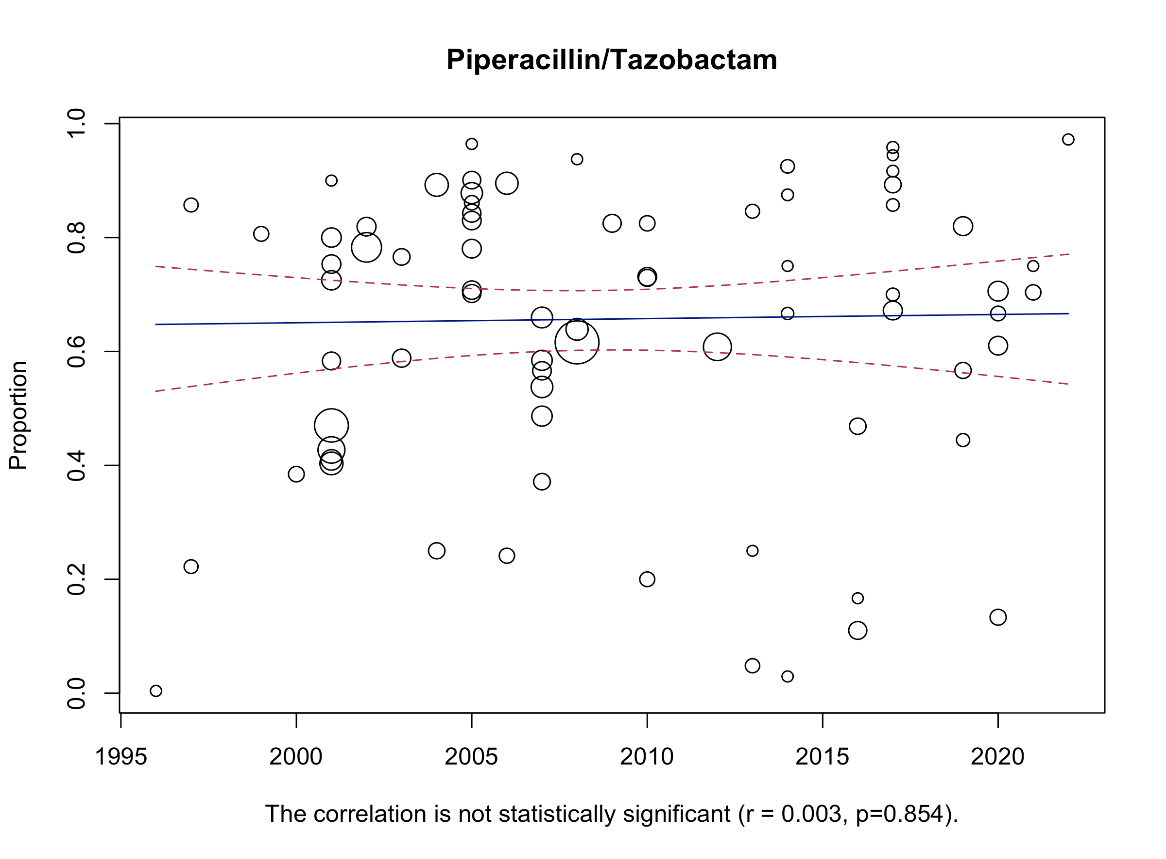


Figure 18. Bubble plot with fitted meta-regression for the year of publication.


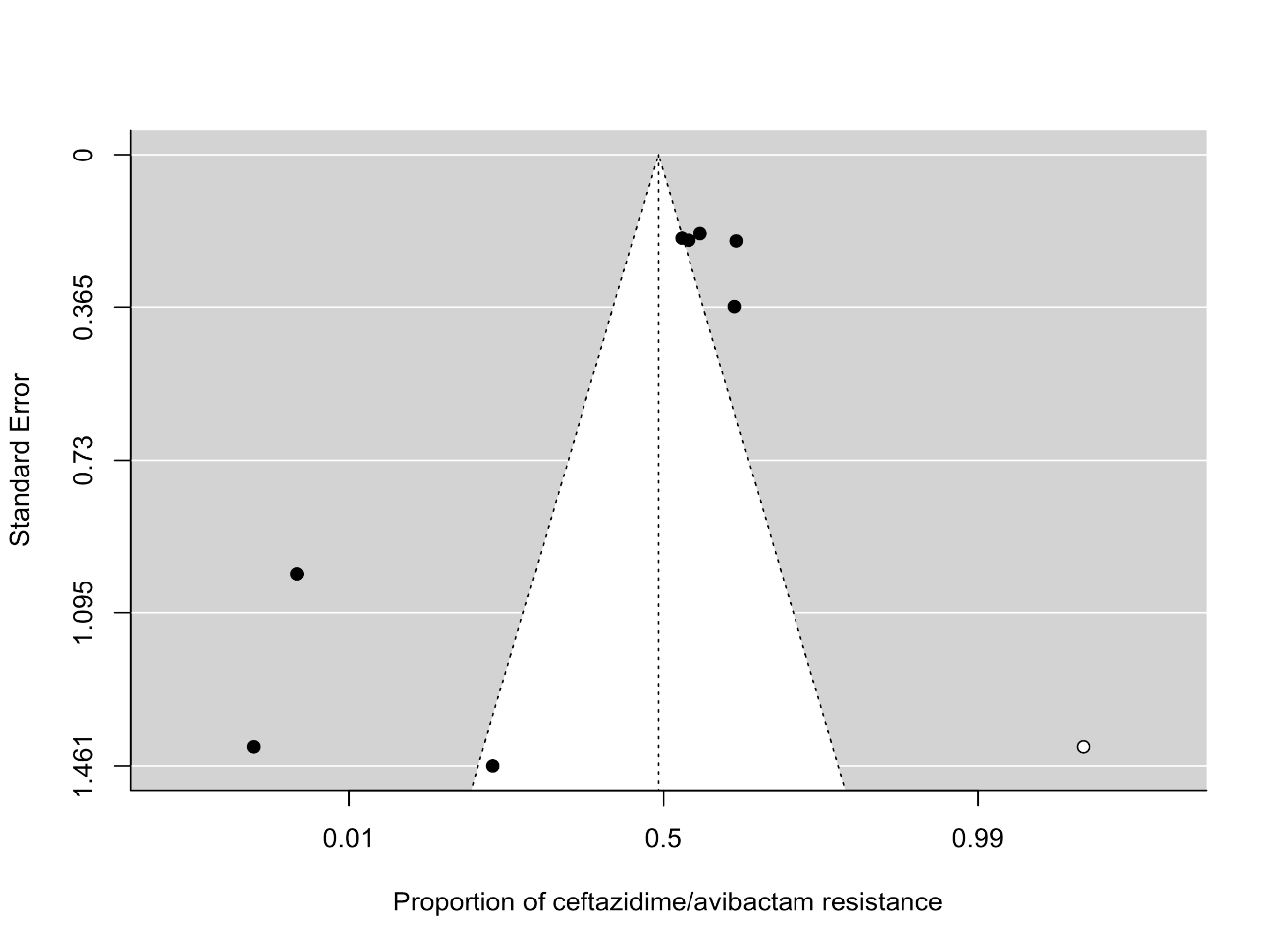
Figure 19. The funnel plot of meta-analysis of publication studies. Each black dot represents a study. The white dots represent missing studies. The black line in the middle represents the average effect size.


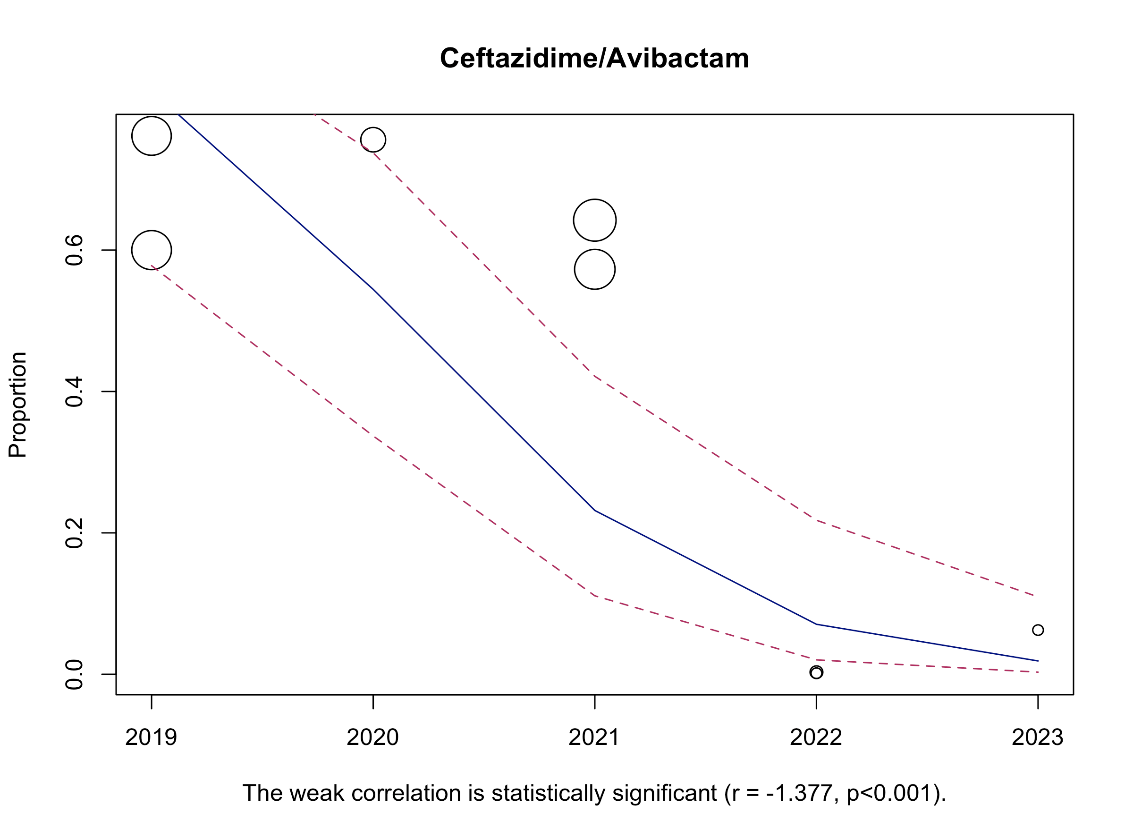


Figure 20. Bubble plot with fitted meta-regression for the year of publication.

Figure 21. The funnel plot of meta-analysis of publication studies. Each black dot represents a study. The black line in the middle represents the average effect size
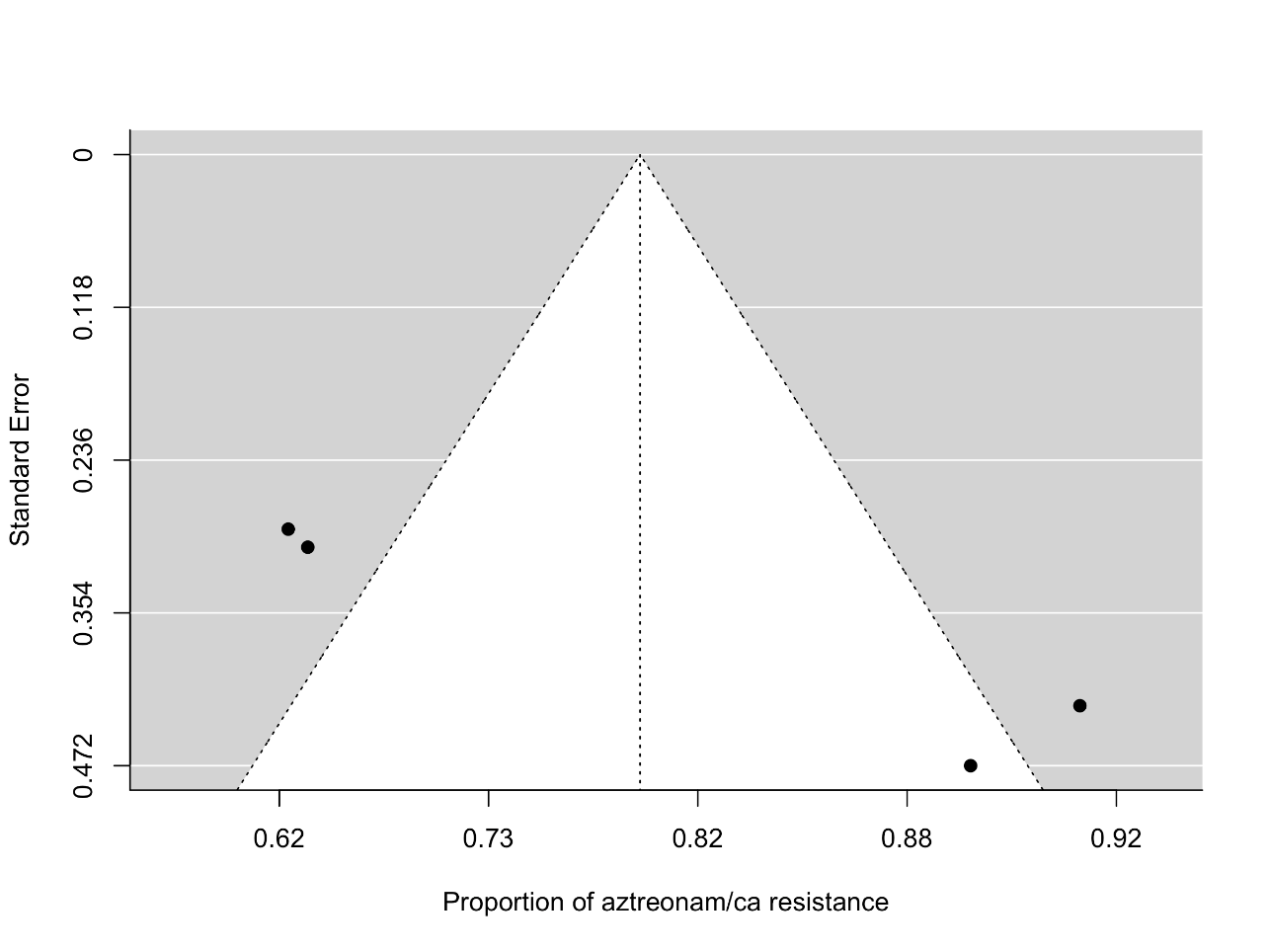
.


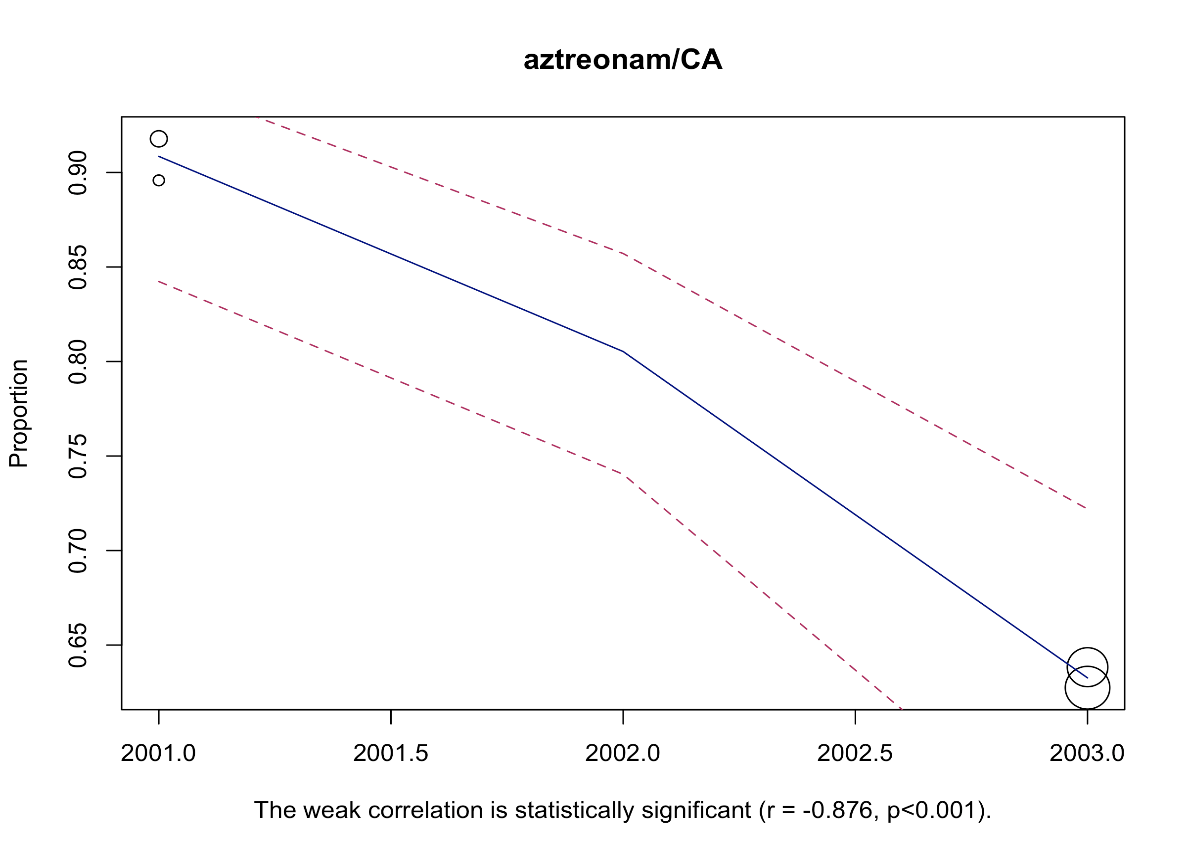


Figure 22. Bubble plot with fitted meta-regression for the year of publication.

Figure 23. The funnel plot of meta-analysis of publication studies. Each black dot represents a study.
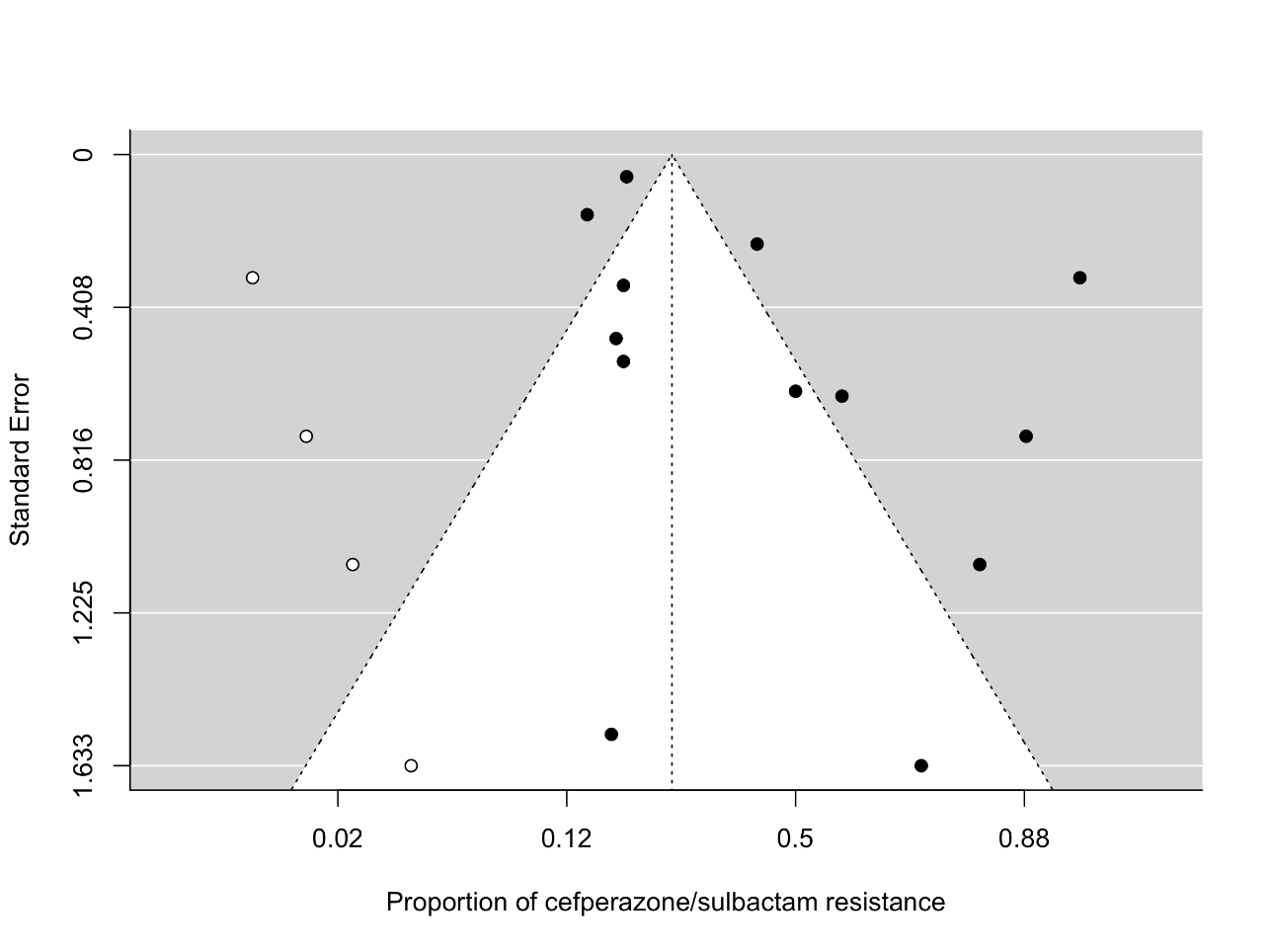
 The white dots represent missing studies. The black line in the middle represents the average effect size.


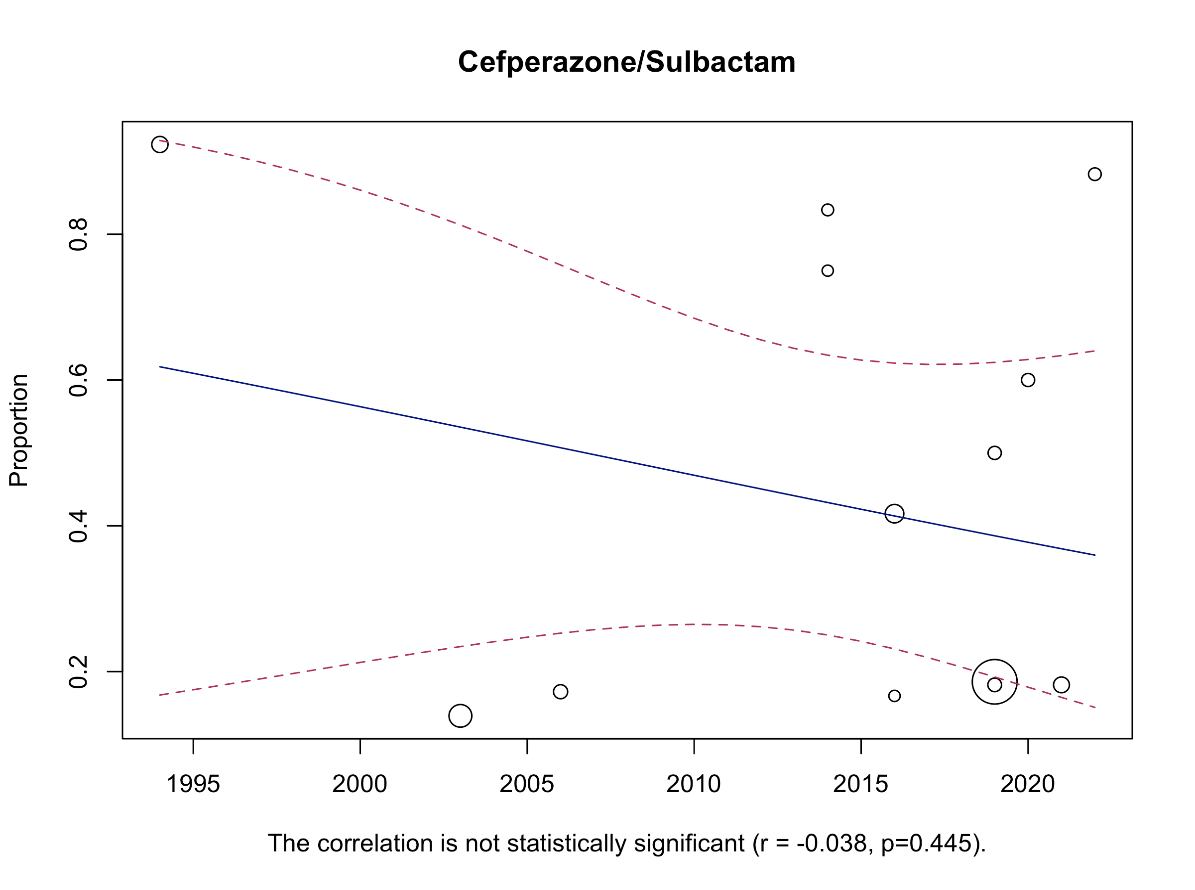


Figure 24. Bubble plot with fitted meta-regression for the year of publication.


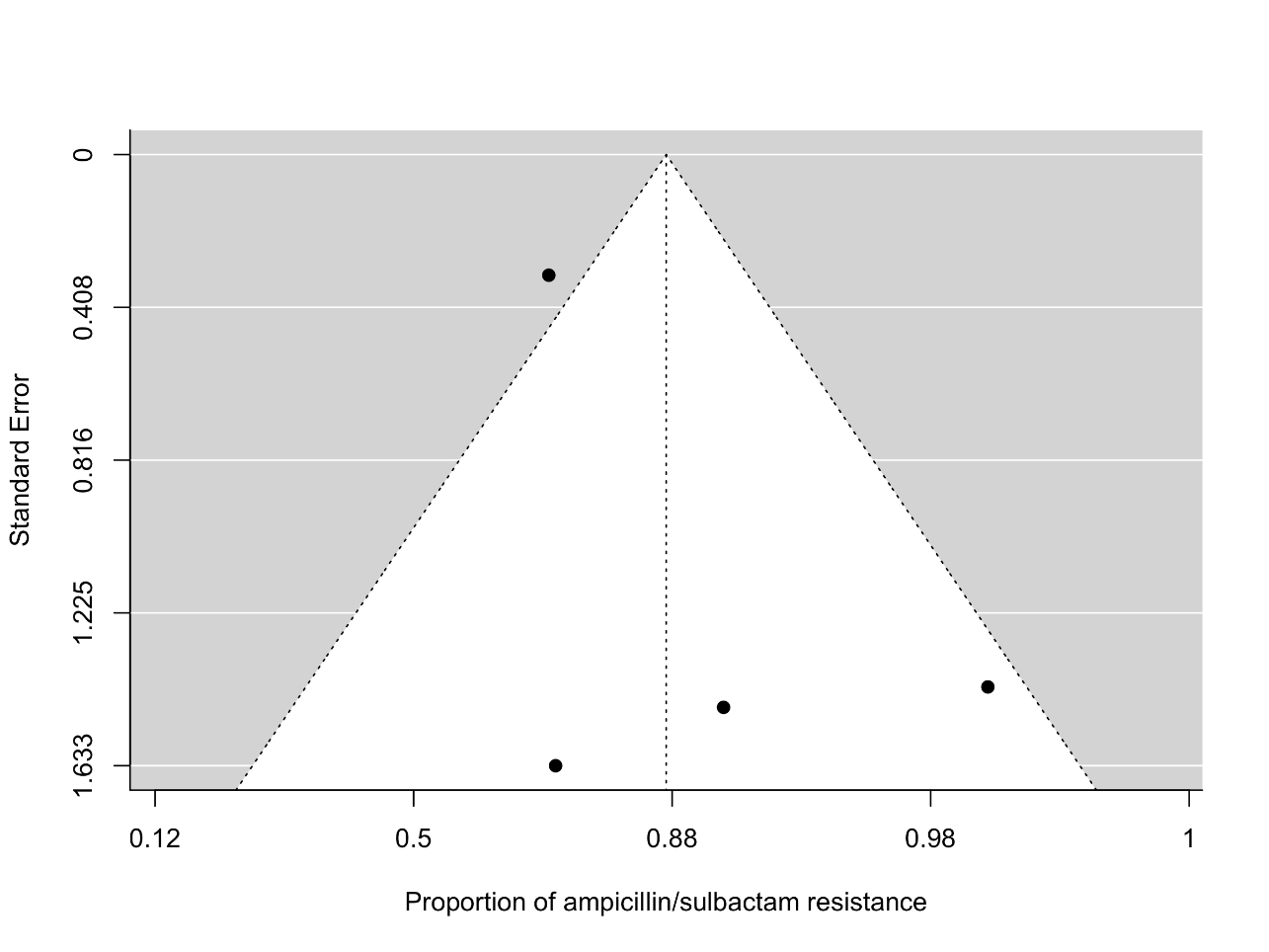


Figure 25. The funnel plot of meta-analysis of publication studies. Each black dot represents a study. The black line in the middle represents the average effect size.


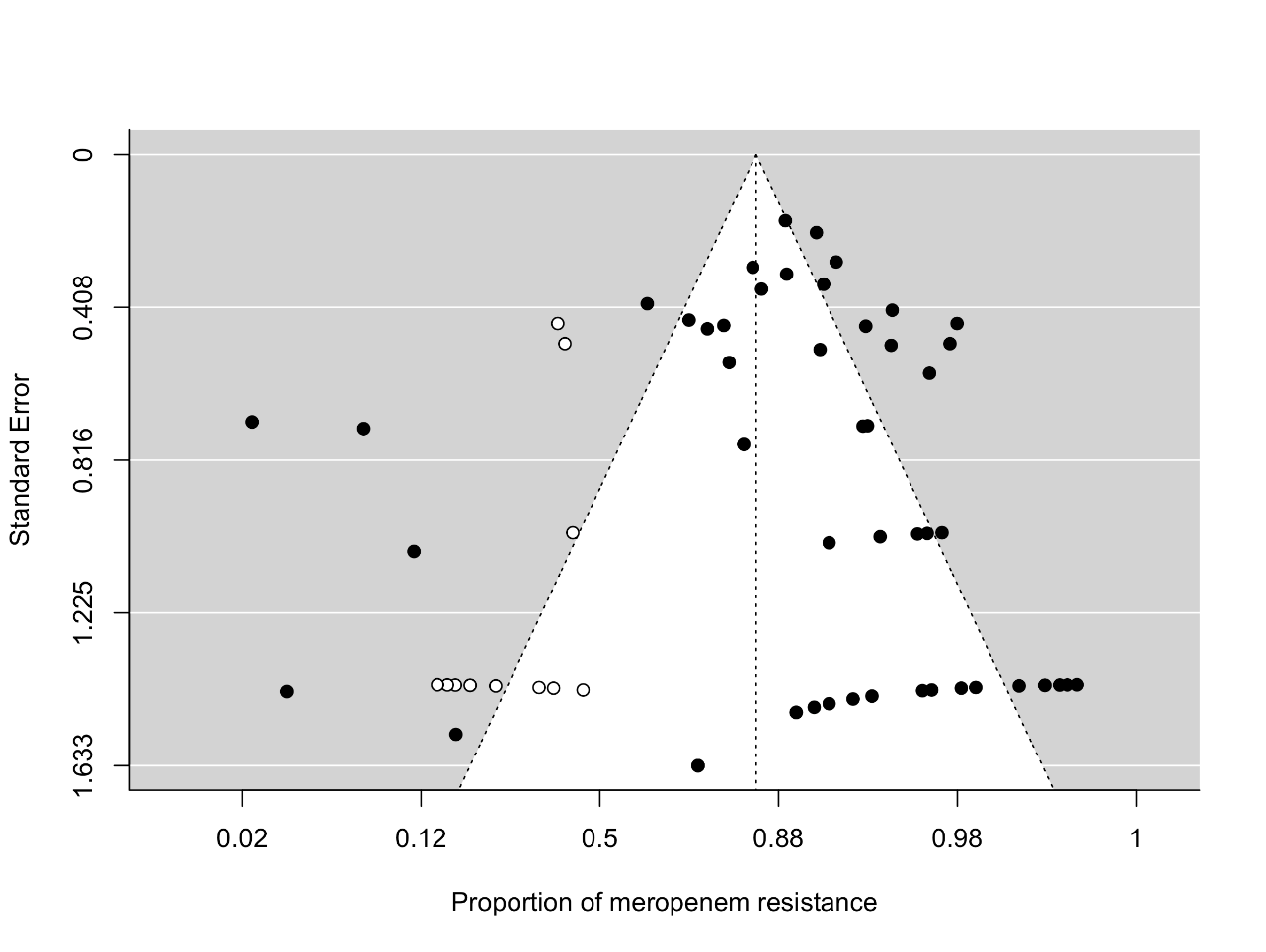


**Carbapenems**

Figure 26. The funnel plot of meta-analysis of publication studies. Each black dot represents a study. The white dots represent missing studies. The black line in the middle represents the average effect size.


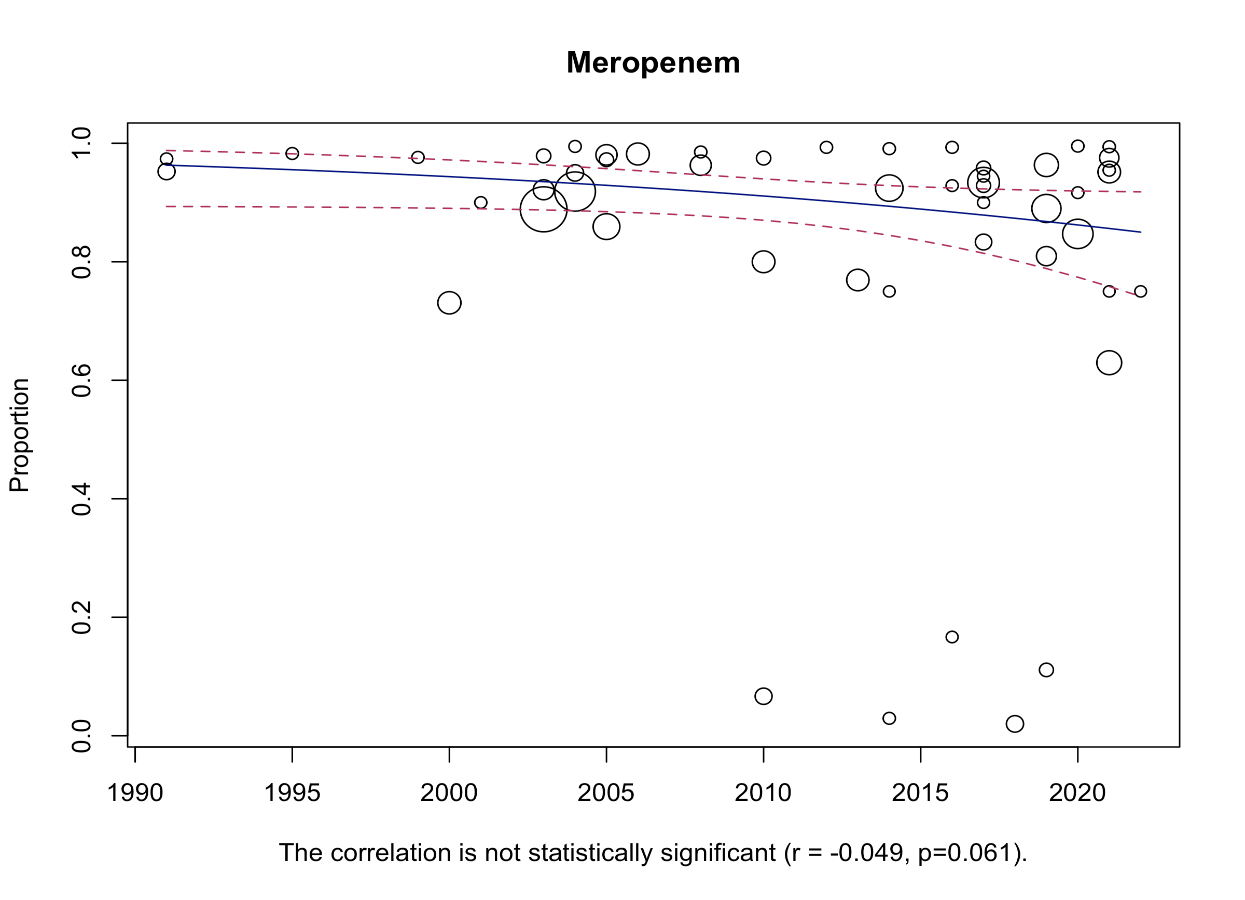


Figure 27. Bubble plot with fitted meta-regression for the year of publication


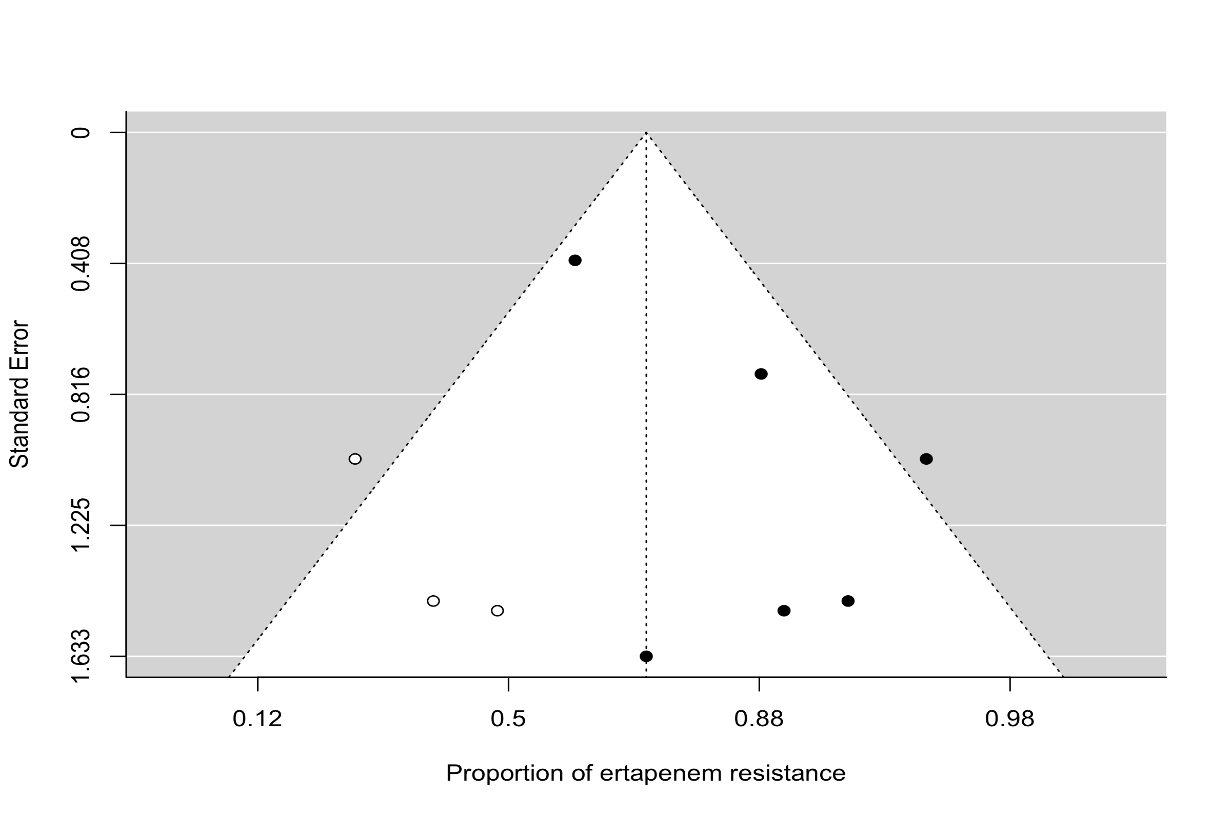
.

Figure 28. The funnel plot of meta-analysis of publication studies. Each black dot represents a study. The white dots represent missing studies. The black line in the middle represents the average effect size.


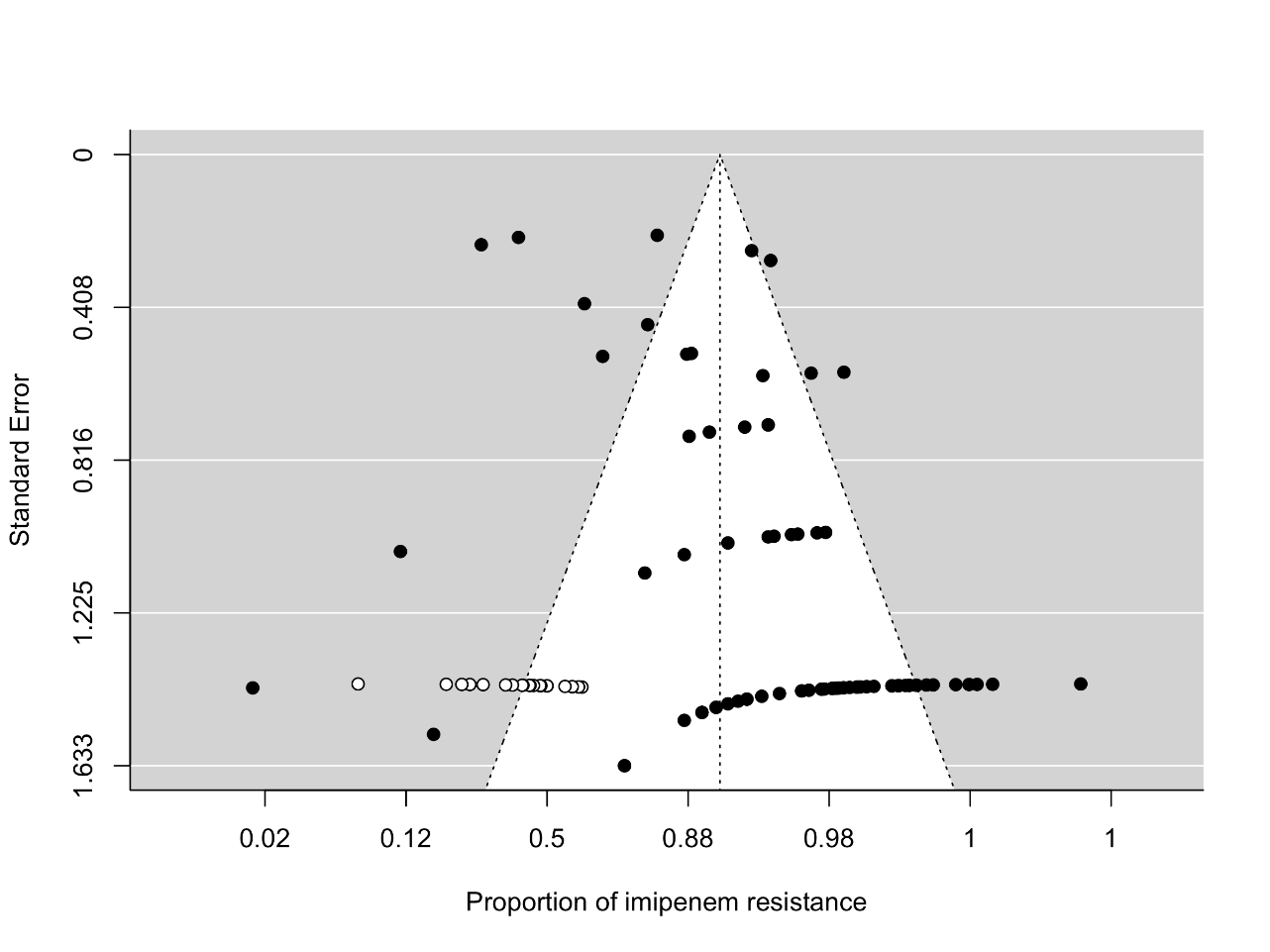
Figure 29. The funnel plot of meta-analysis of publication studies. Each black dot represents a study. The white dots represent missing studies. The black line in the middle represents the average effect size.


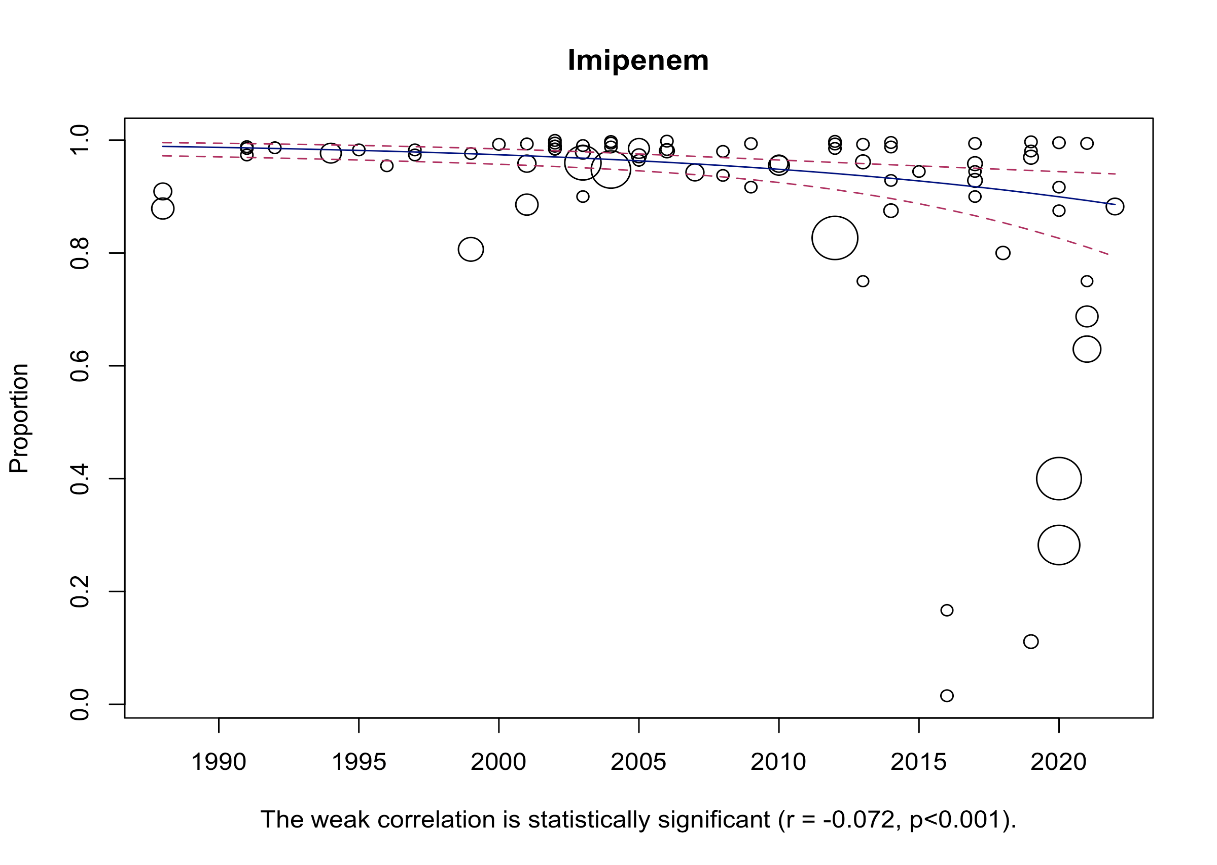


Figure 30. Bubble plot with fitted meta-regression for the year of publication.


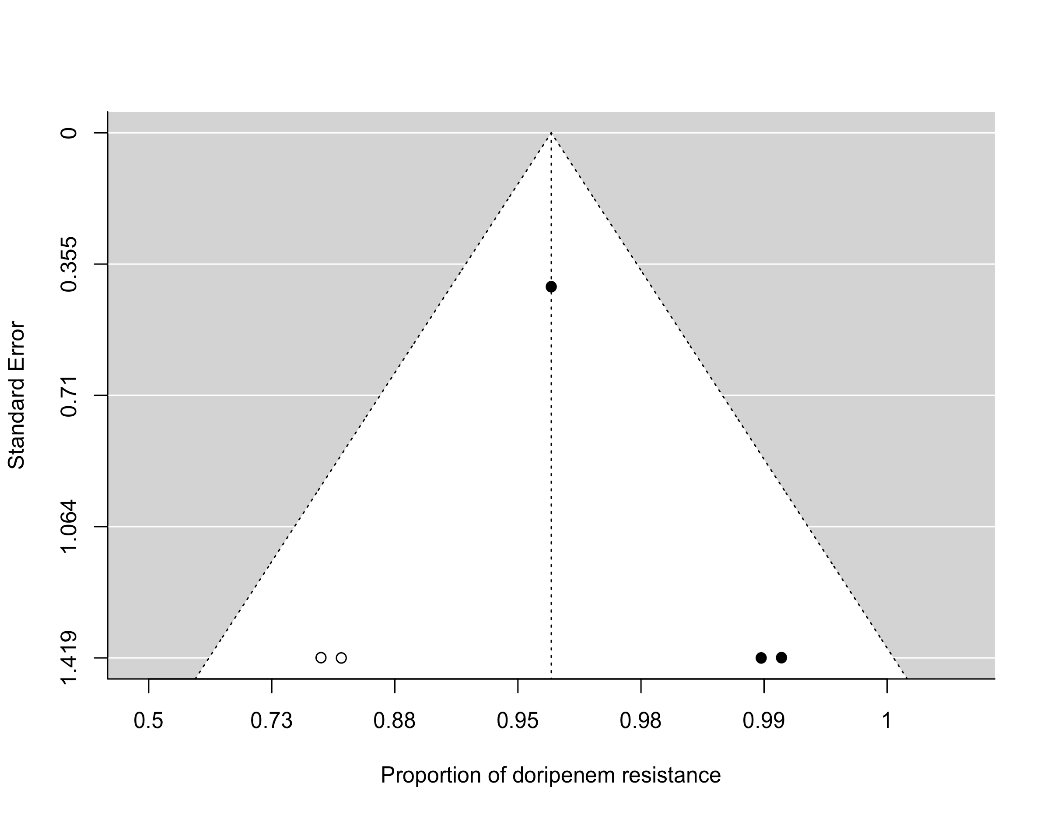


Figure 31. The funnel plot of meta-analysis of publication studies. Each black dot represents a study. The black line in the middle represents the average effect size

**Cephalosporins**

Figure 32. The funnel plot of meta-analysis of publication studies. Each black dot represents a study. The white dots represent missing studies. The black line in the middle represents the average effect size
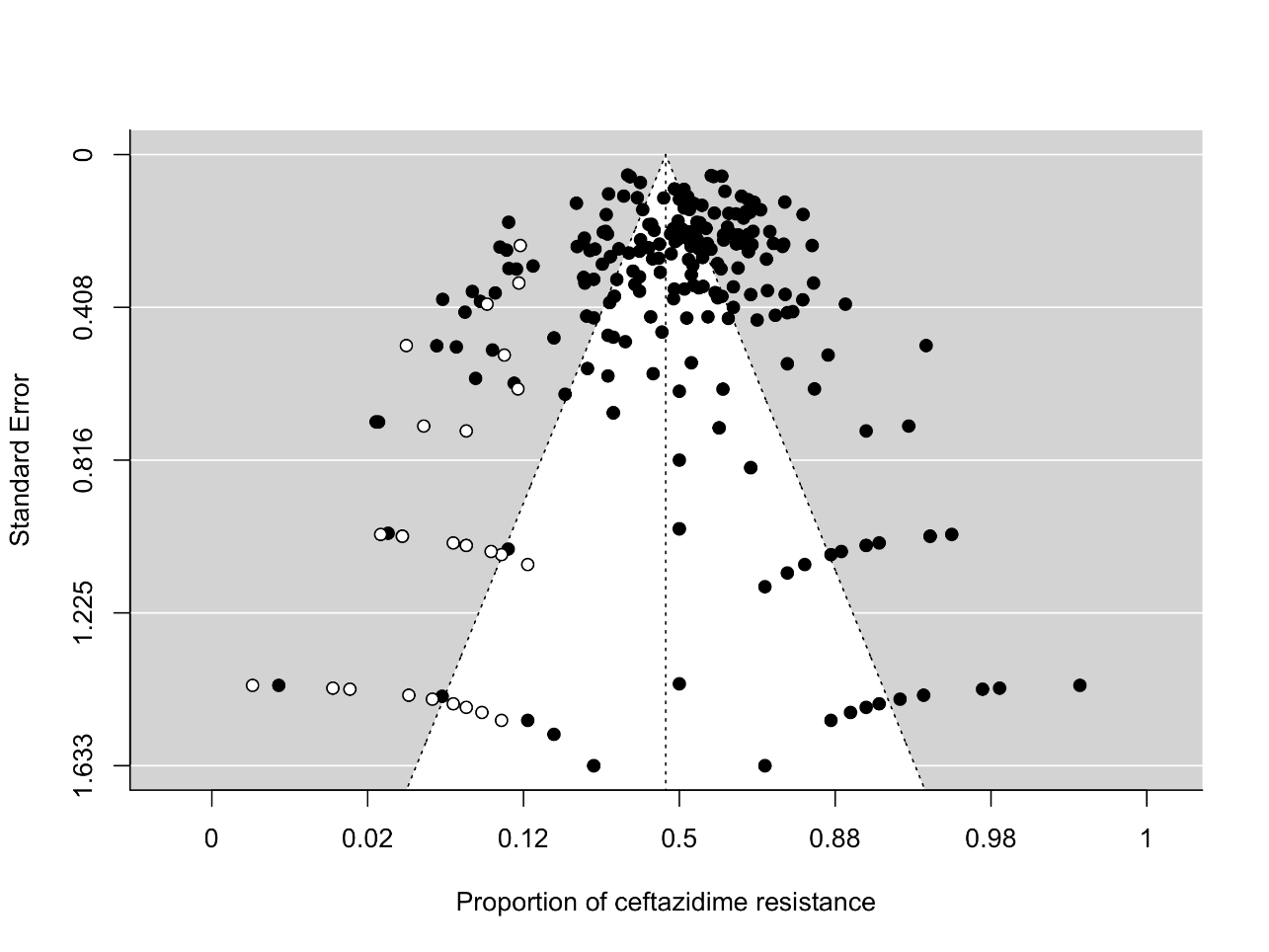
.


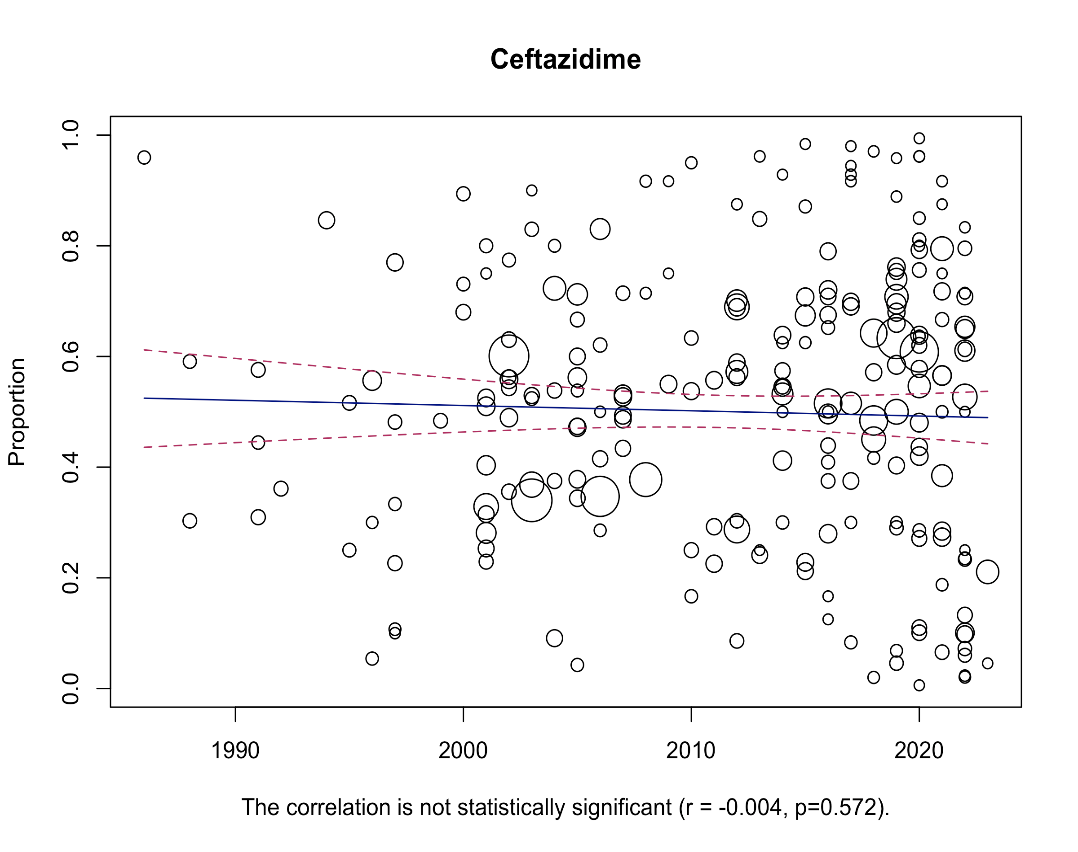


Figure 33. Bubble plot with fitted meta-regression for the year of publication.

Figure 34. The funnel plot of meta-analysis of publication studies. Each black dot represents a study. The white dots represent missing studies. The black line in the middle represents the average effect siz
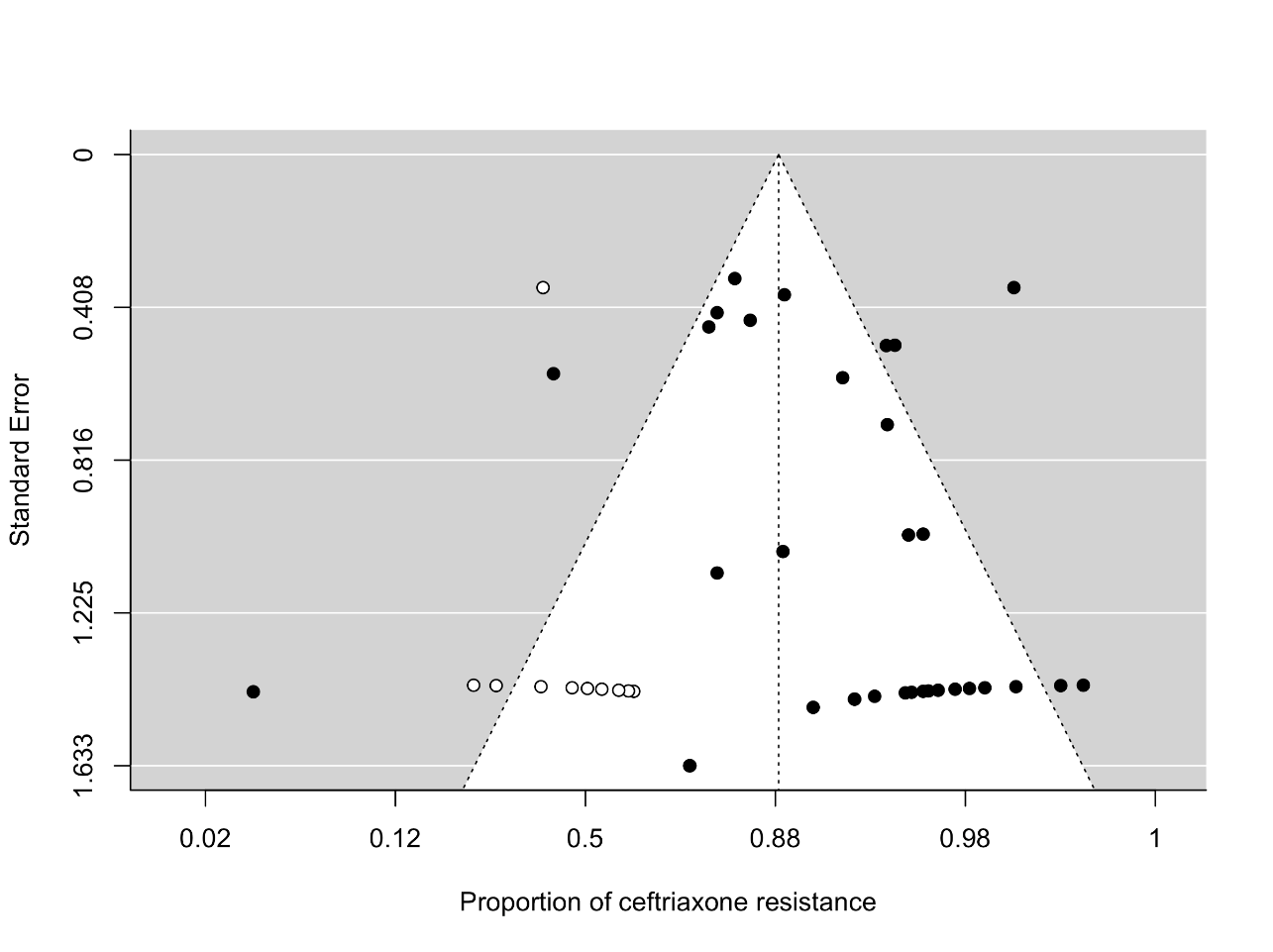
e


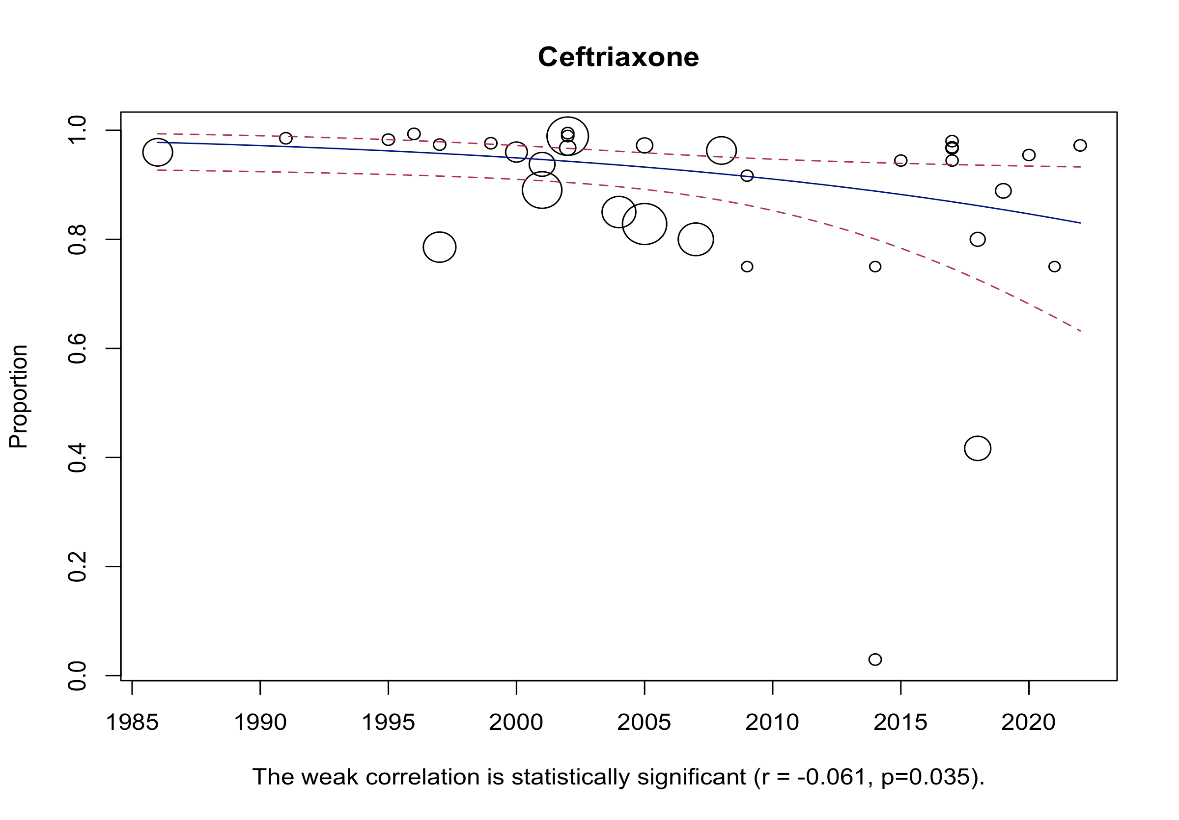


Figure 35. Bubble plot with fitted meta-regression for the year of publication.


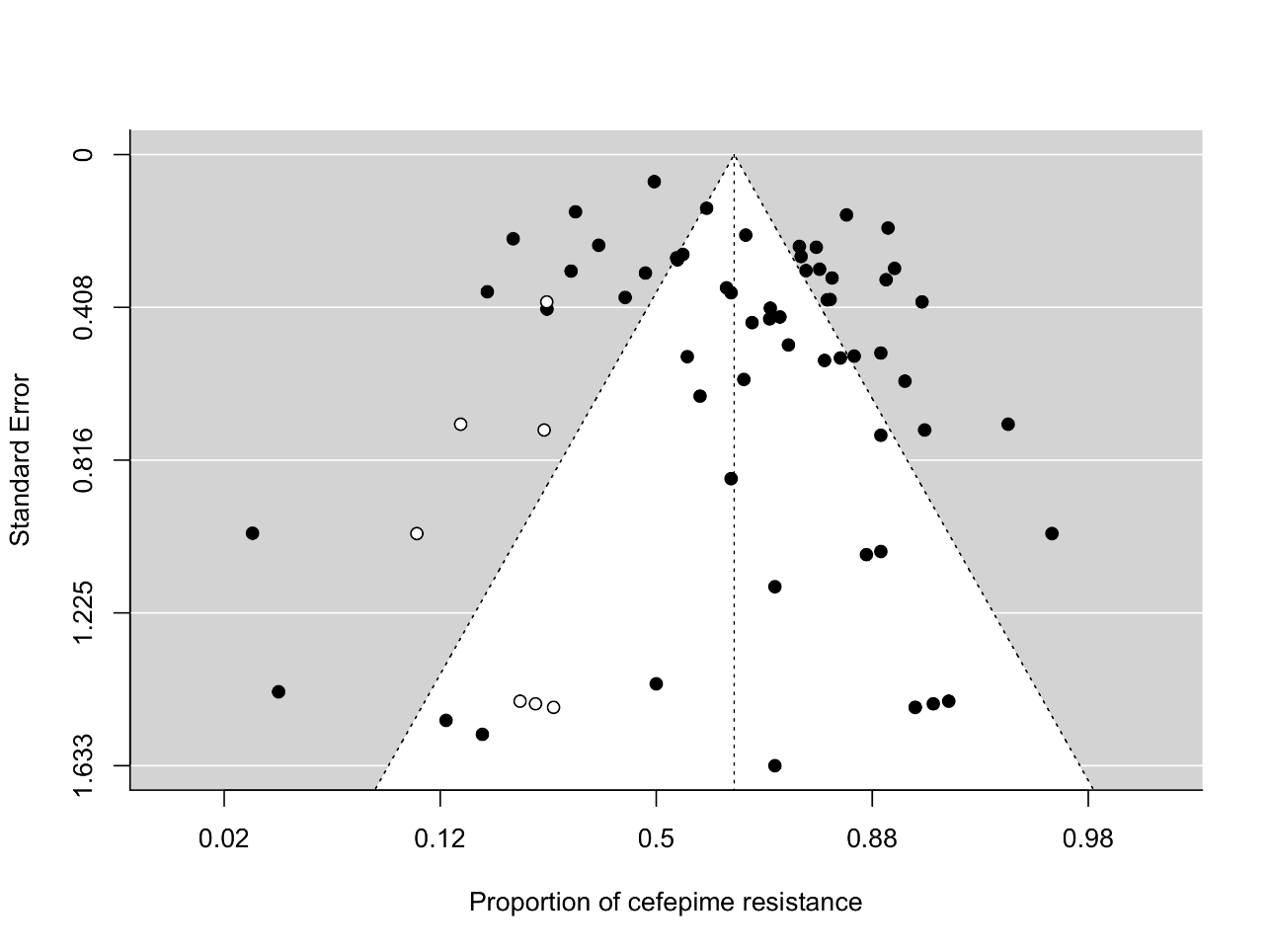
Figure 36. The funnel plot of meta-analysis of publication studies. Each black dot represents a study. The white dots represent missing studies. The black line in the middle represents the average effect size.


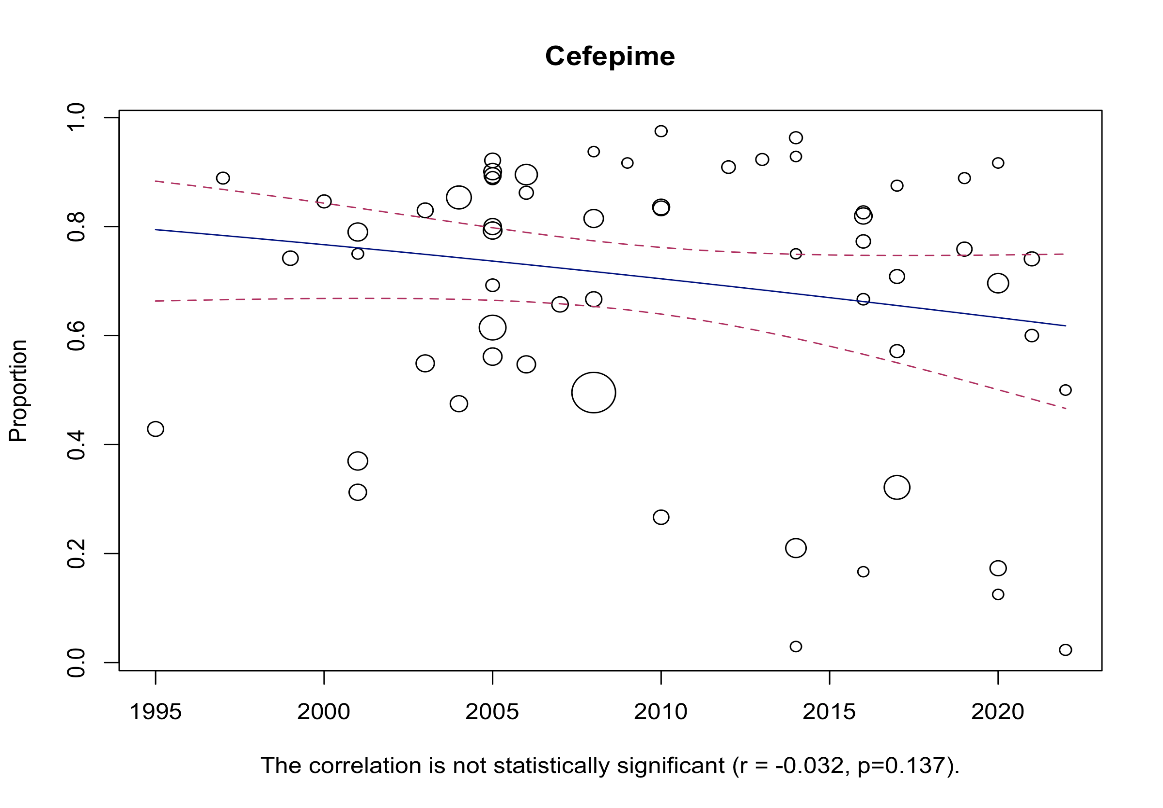


Figure 37. Bubble plot with fitted meta-regression for the year of publication.


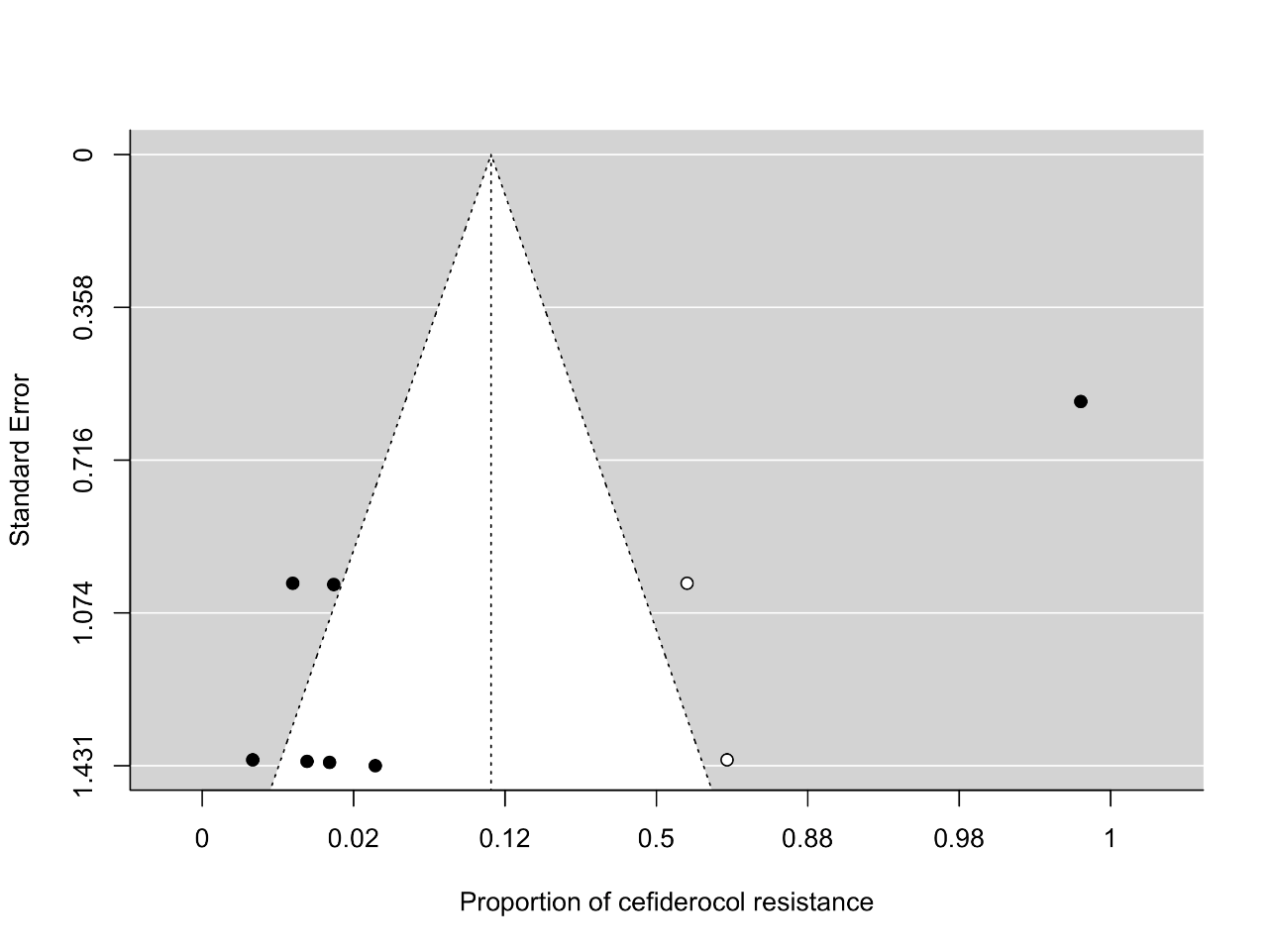
Figure 38. The funnel plot of meta-analysis of publication studies. Each black dot represents a study. The white dots represent missing studies. The black line in the middle represents the average effect size


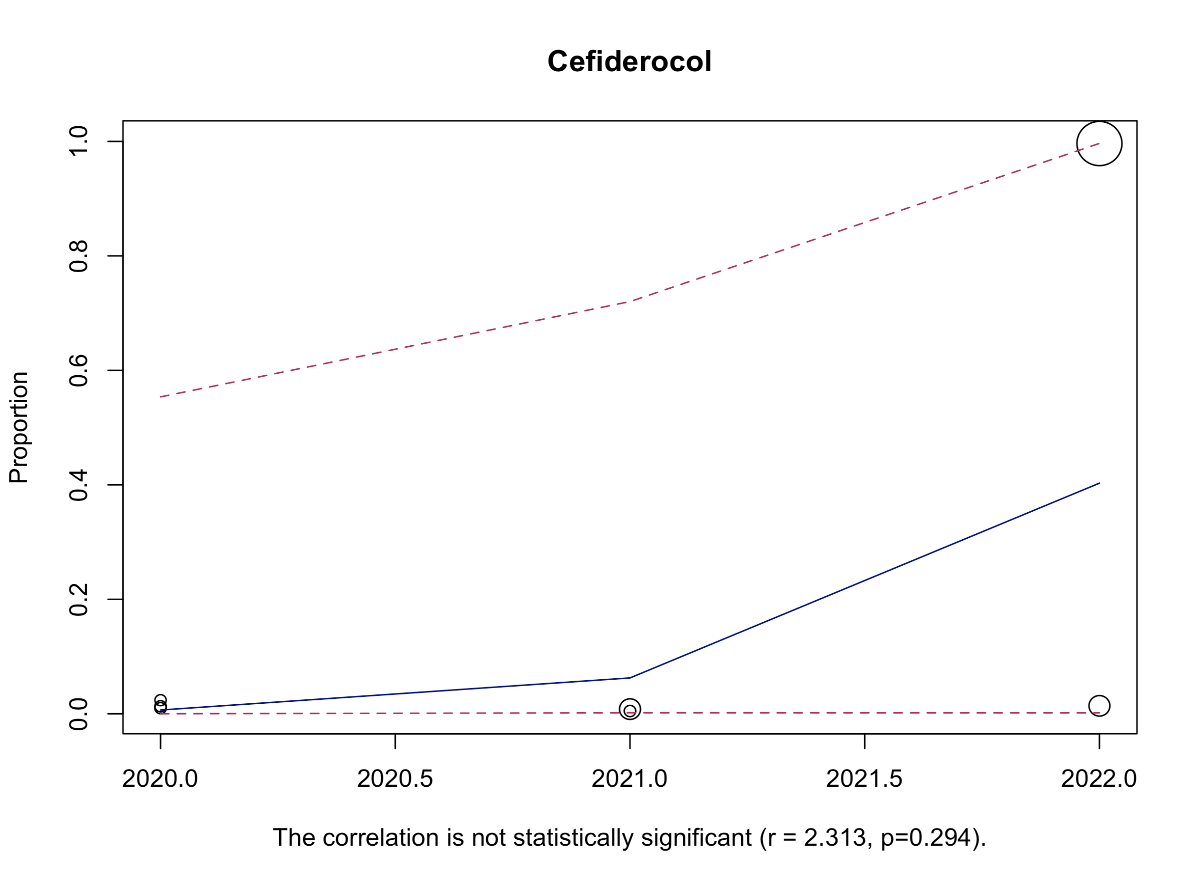


Figure 39. Bubble plot with fitted meta-regression for the year of publication.


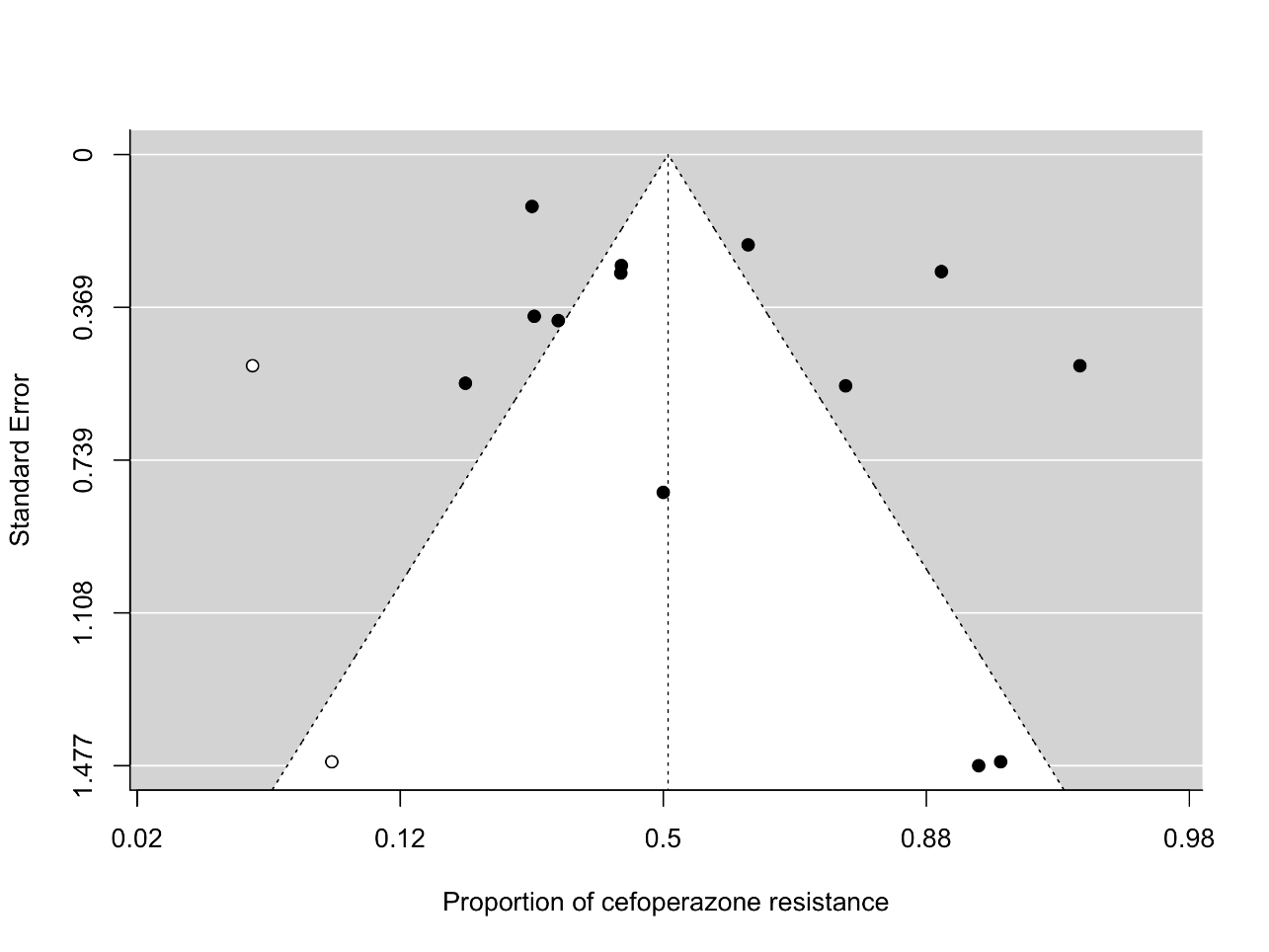


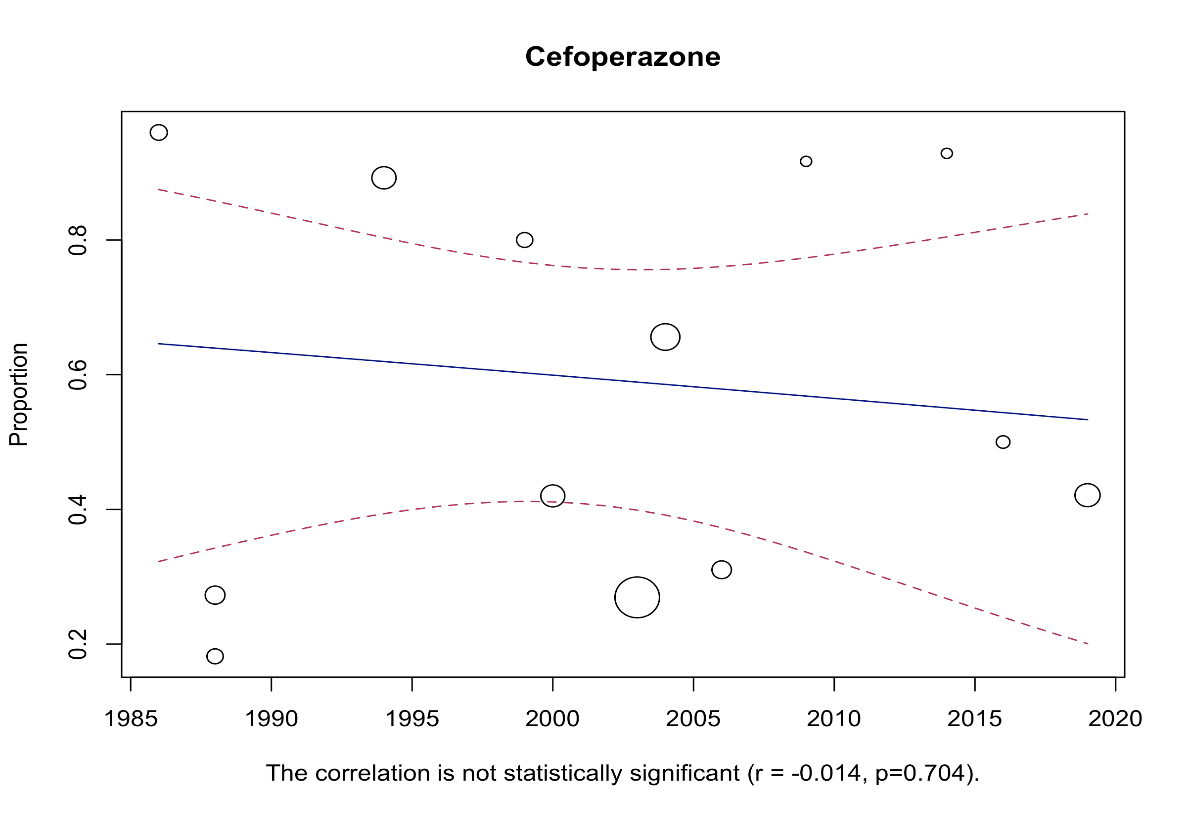
Figure 40. The funnel plot of meta-analysis of publication studies. Each black dot represents a study. The white dots represent missing studies. The black line in the middle represents the average effect size

Figure 41. Bubble plot with fitted meta-regression for the year of publication


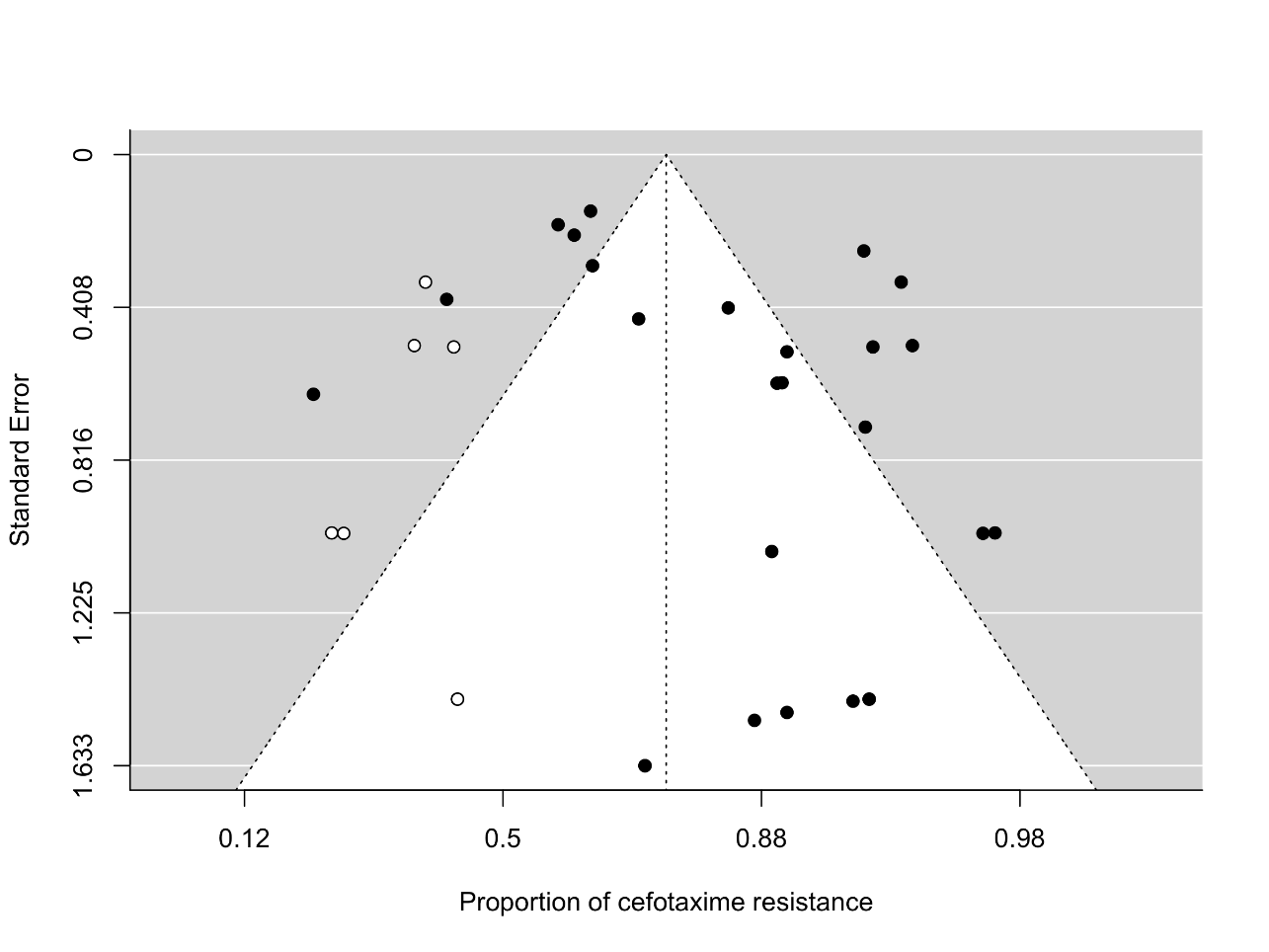


Figure 42. The funnel plot of meta-analysis of publication studies. Each black dot represents a study. The white dots represent missing studies. The black line in the middle represents the average effect size.


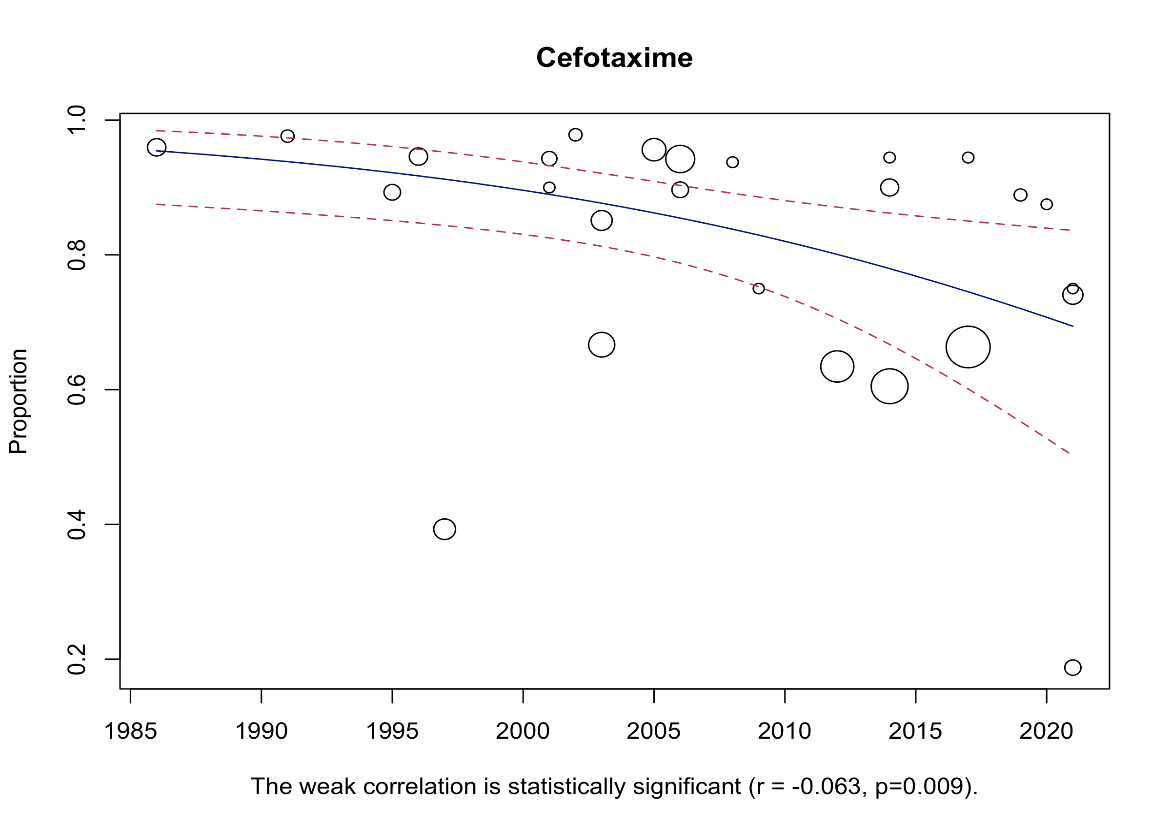


Figure 43. Bubble plot with fitted meta-regression for the year of publication.


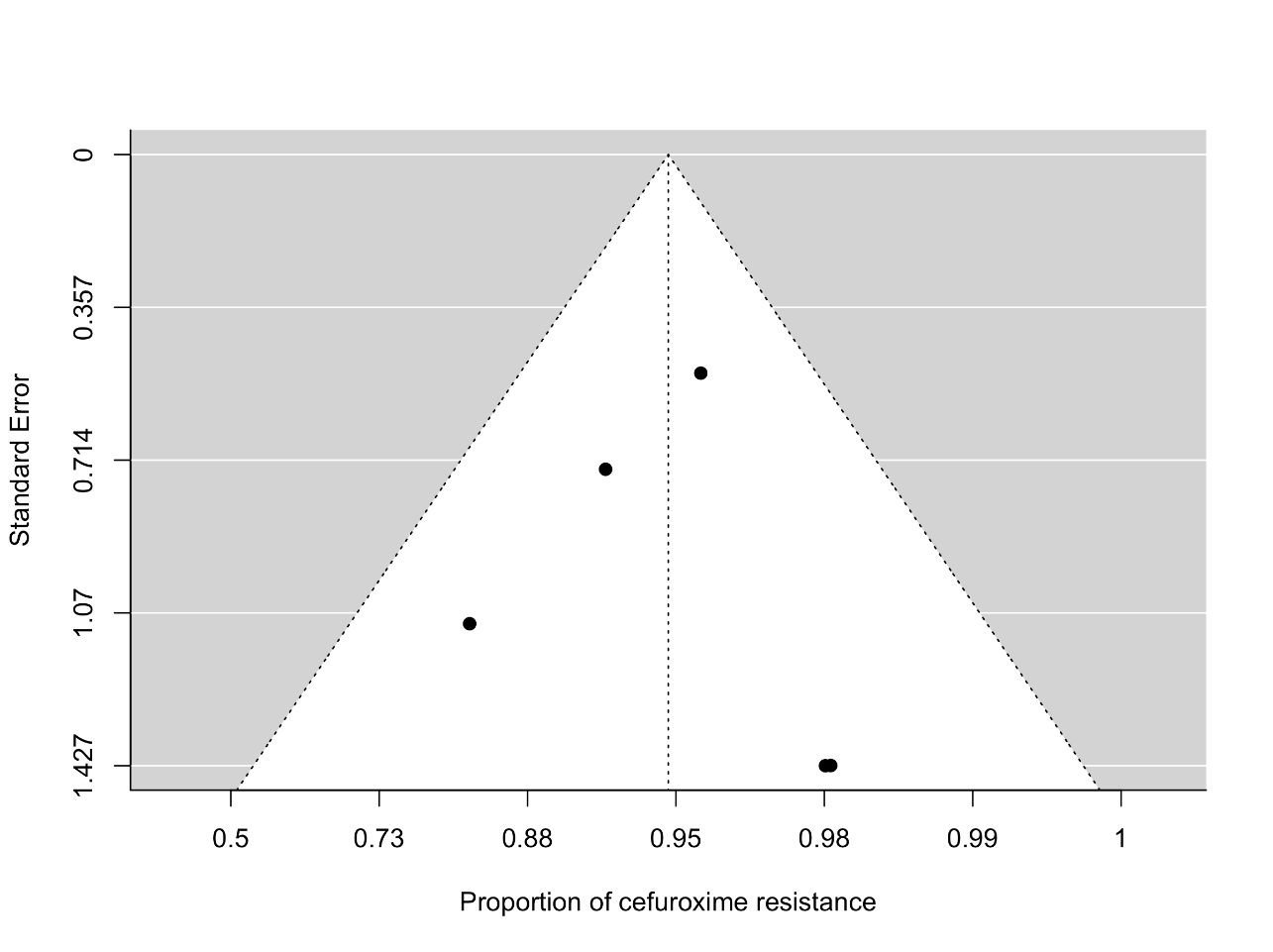


Figure 44. The funnel plot of meta-analysis of publication studies. Each black dot represents a study. The black line in the middle represents the average effect size.


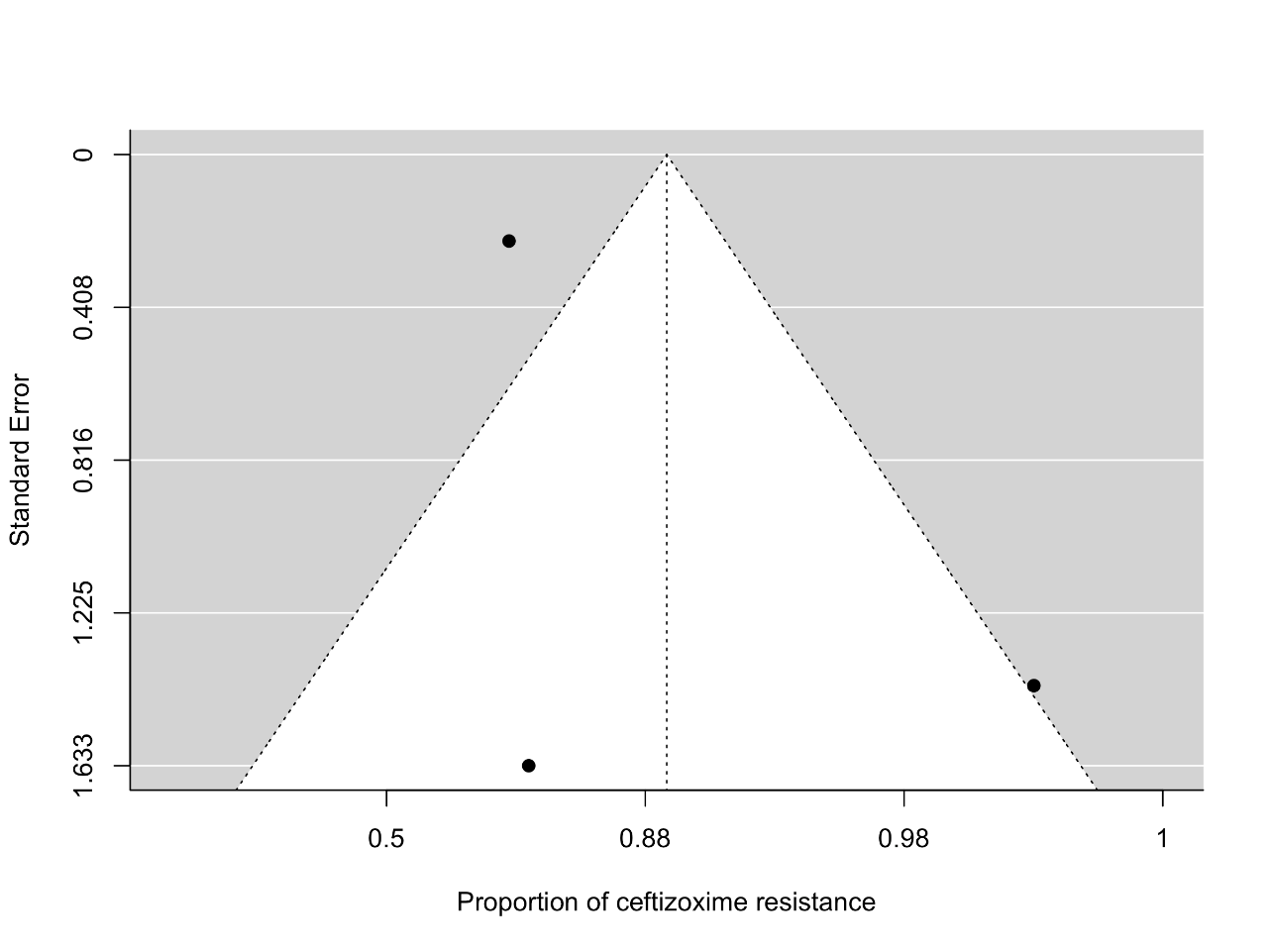


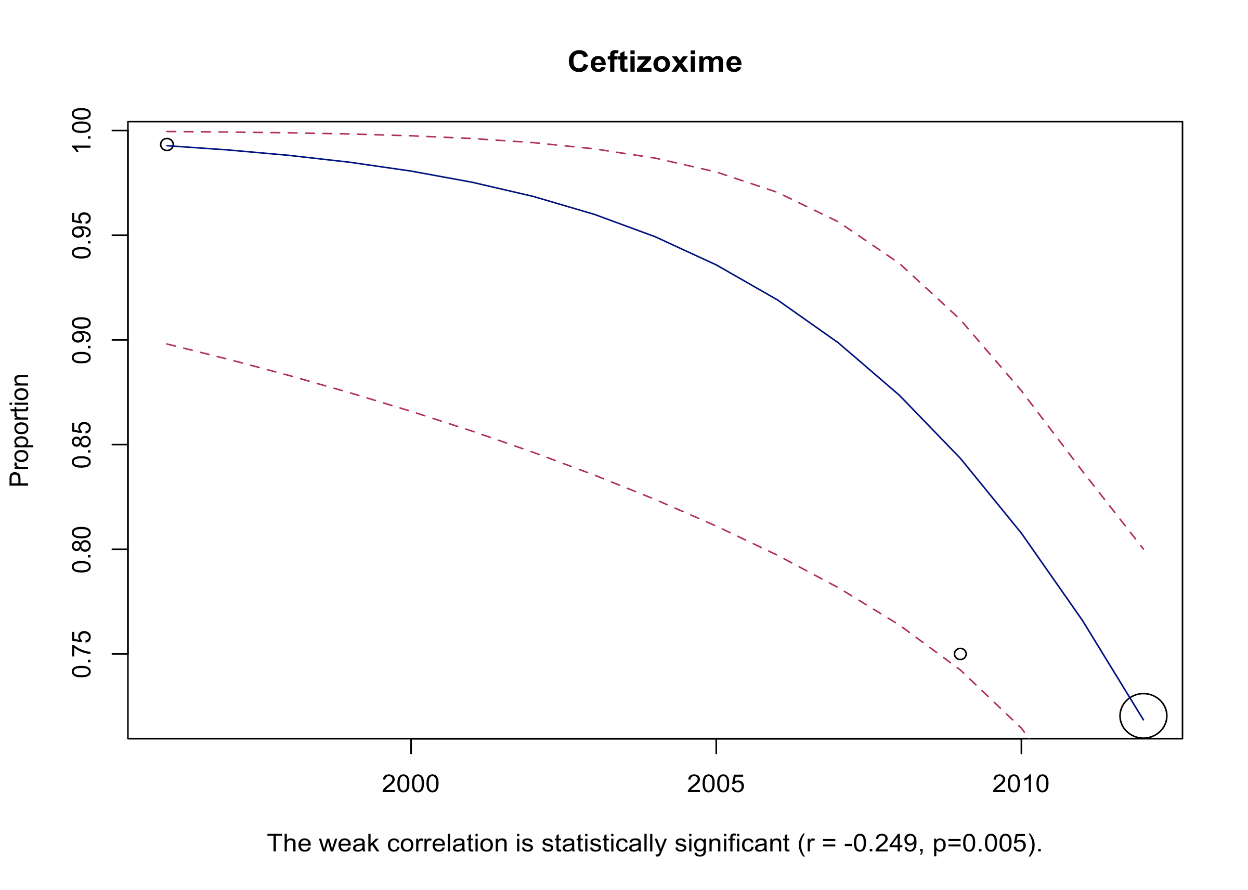
Figure 45. The funnel plot of meta-analysis of publication studies. Each black dot represents a study. The black line in the middle represents the average effect size

Figure 46. Bubble plot with fitted meta-regression for the year of publication.


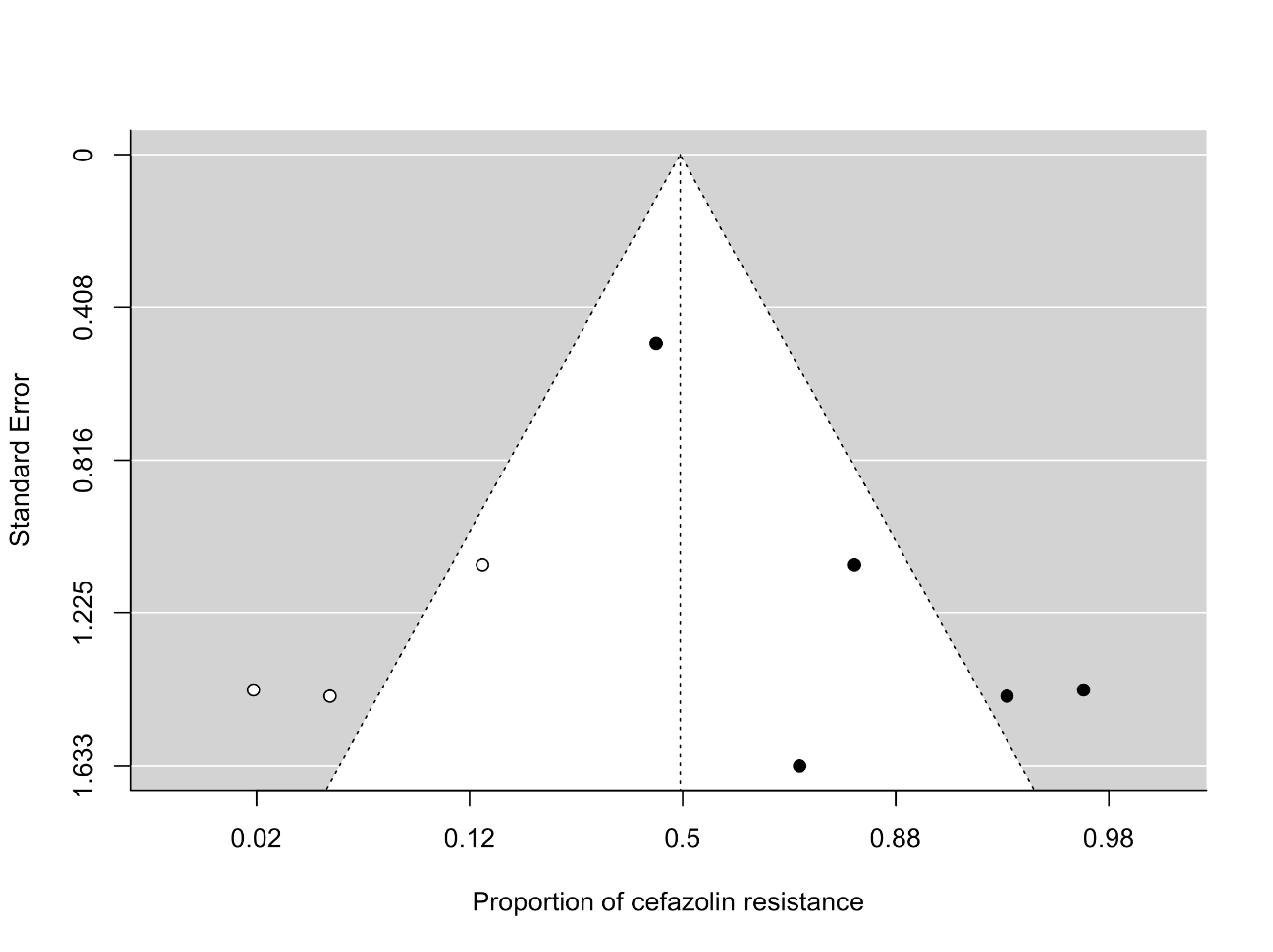


Figure 47. The funnel plot of meta-analysis of publication studies. Each black dot represents a study. The white dots represent missing studies. The black line in the middle represents the average effect size


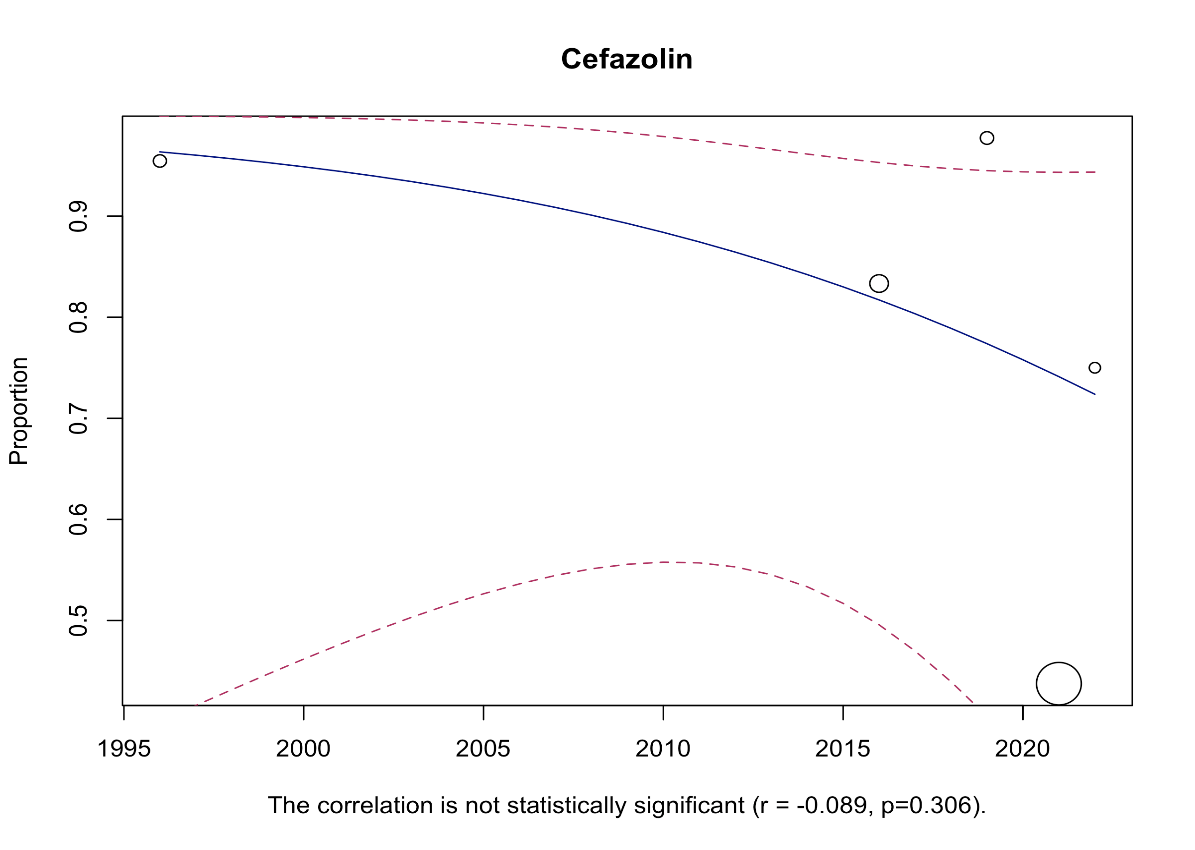


Figure 48. Bubble plot with fitted meta-regression for the year of publication.


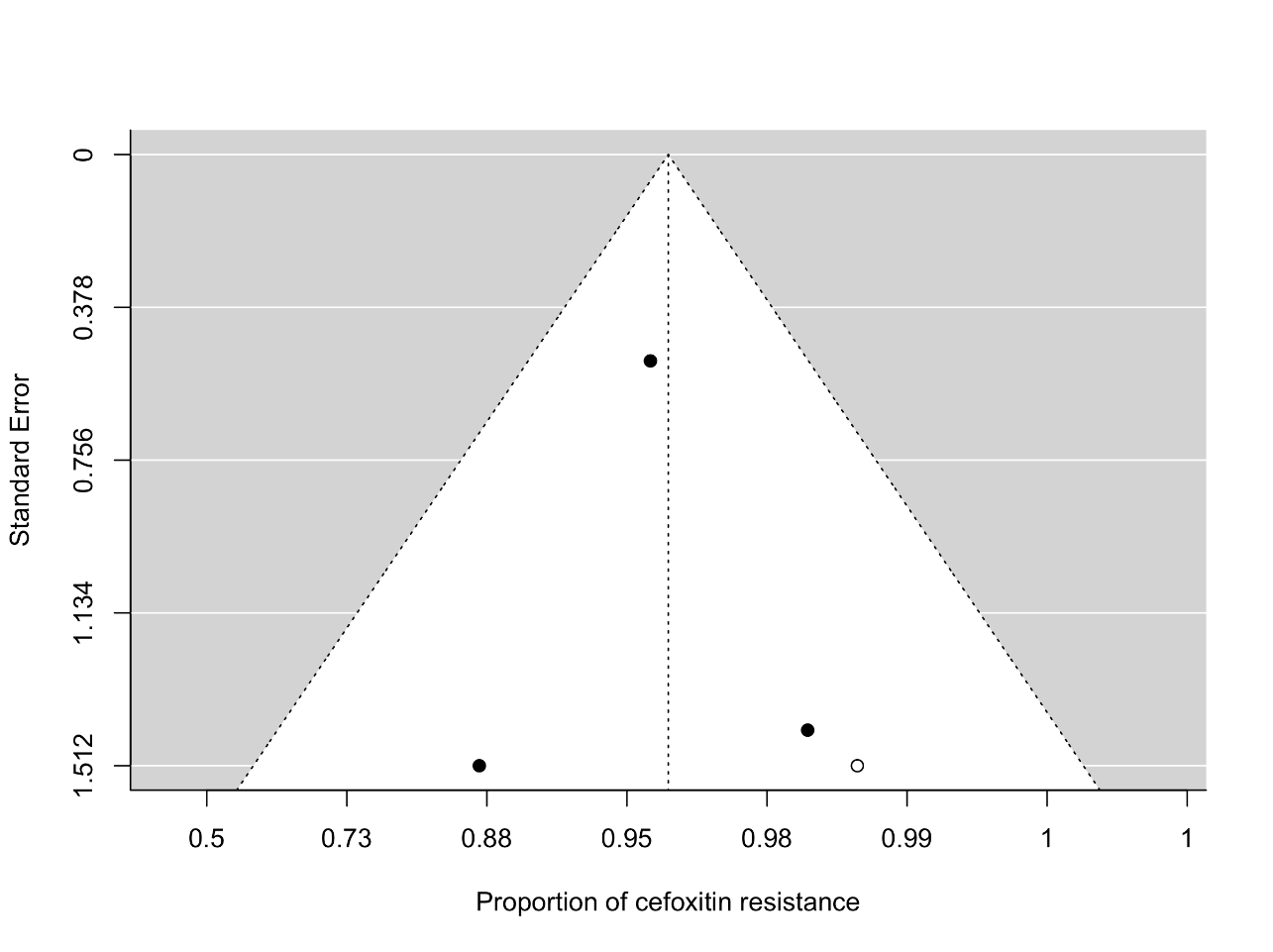


Figure 49. The funnel plot of meta-analysis of publication studies. Each black dot represents a study. The white dots represent missing studies. The black line in the middle represents the average effect size.


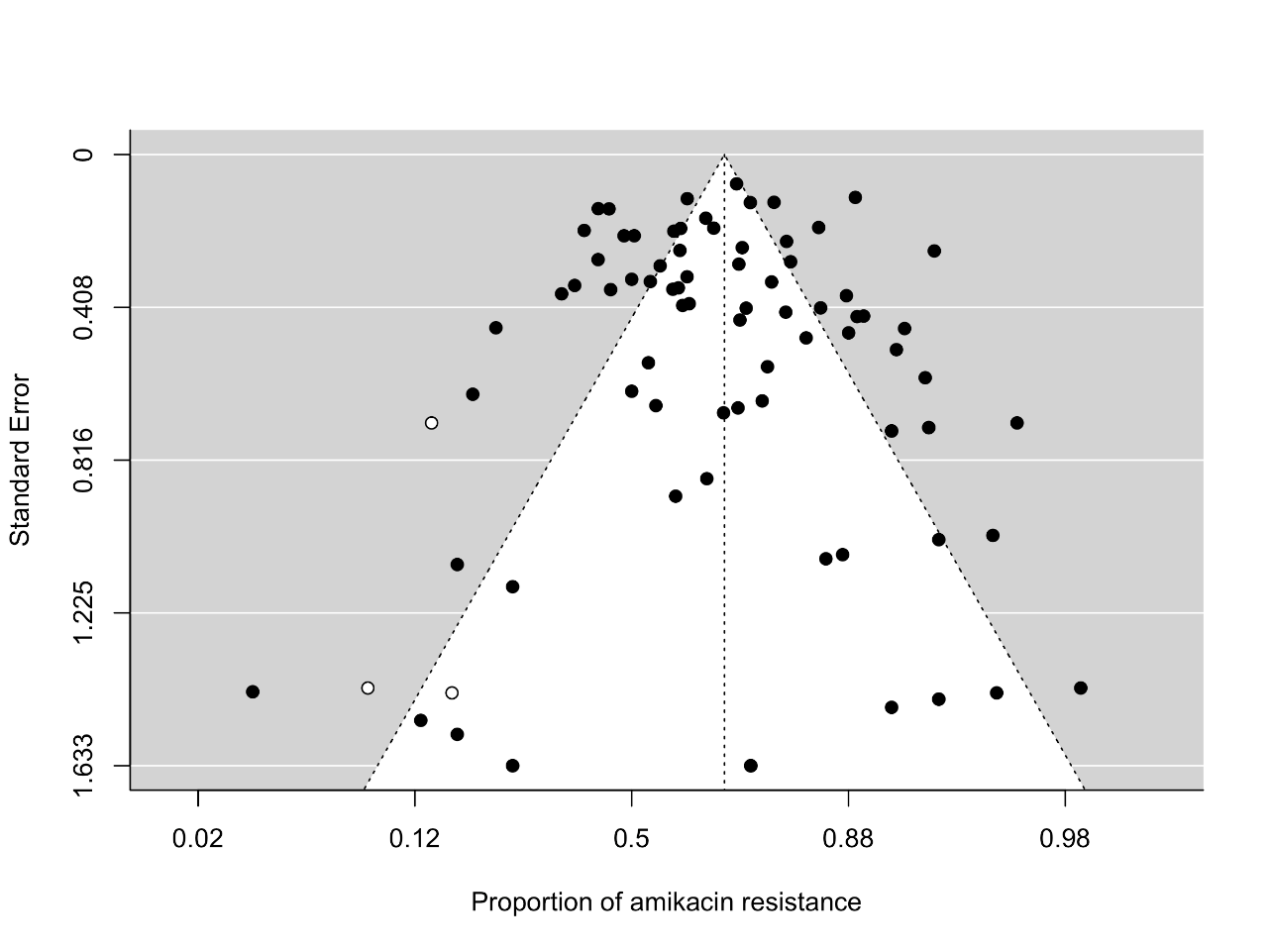


Figure 50. The funnel plot of meta-analysis of publication studies. Each black dot represents a study. The white dots represent missing studies. The black line in the middle represents the average effect size.


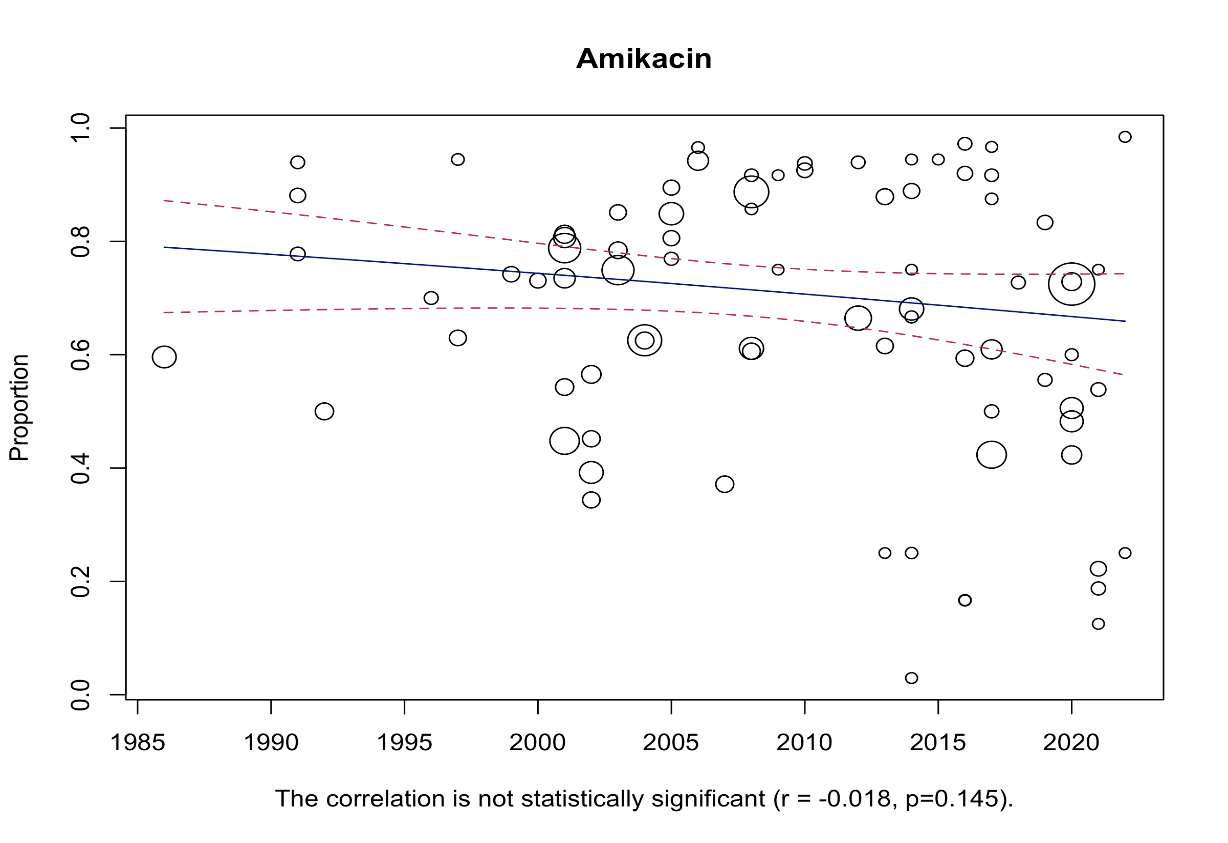


Figure 51. Bubble plot with fitted meta-regression for the year of publication.

Figure 52. The funnel plot of meta-analysis of publication studies. Each black dot represents a study. The white dots represent missing studies. The black line in the middle represents the average effect size.
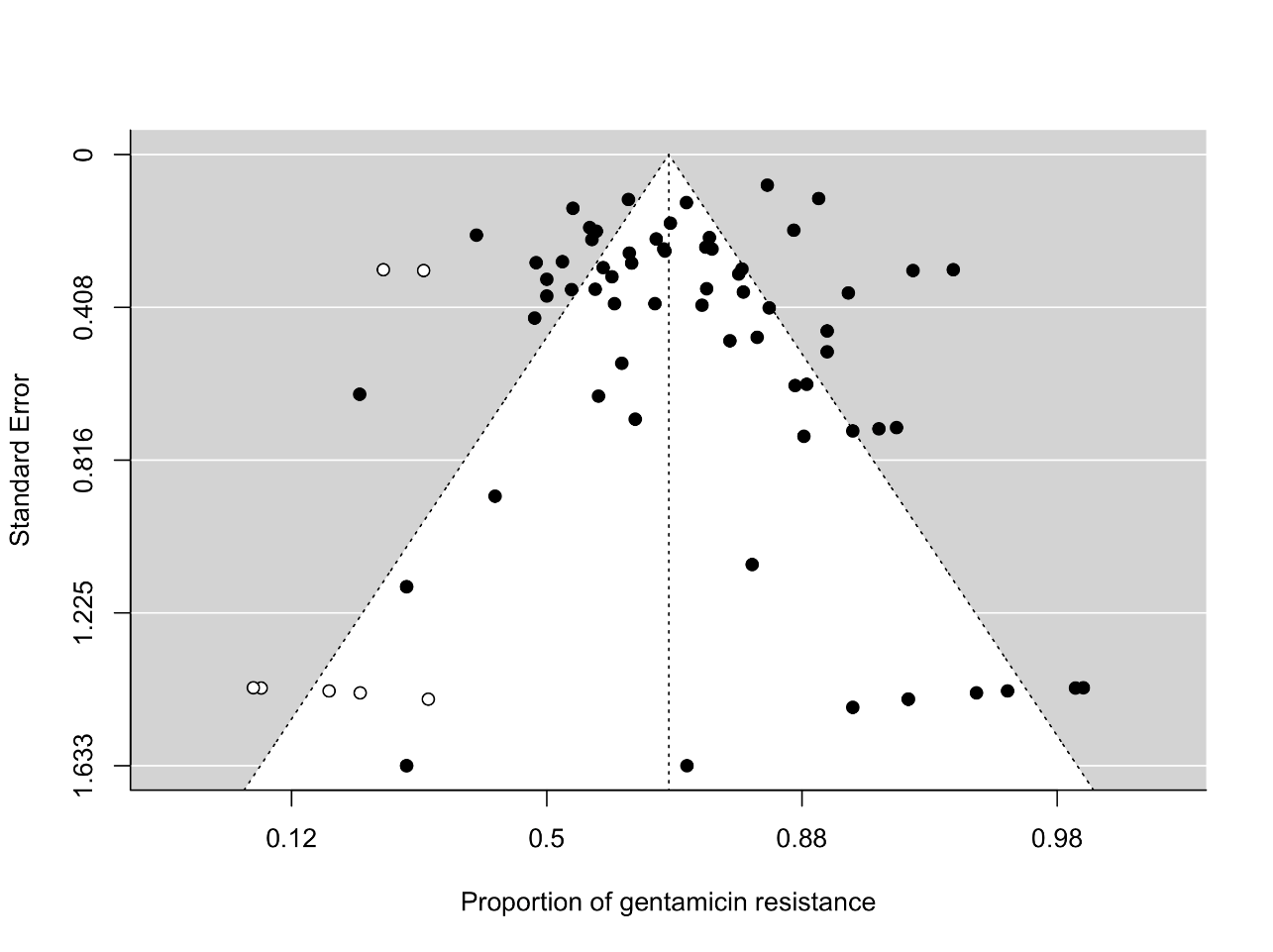


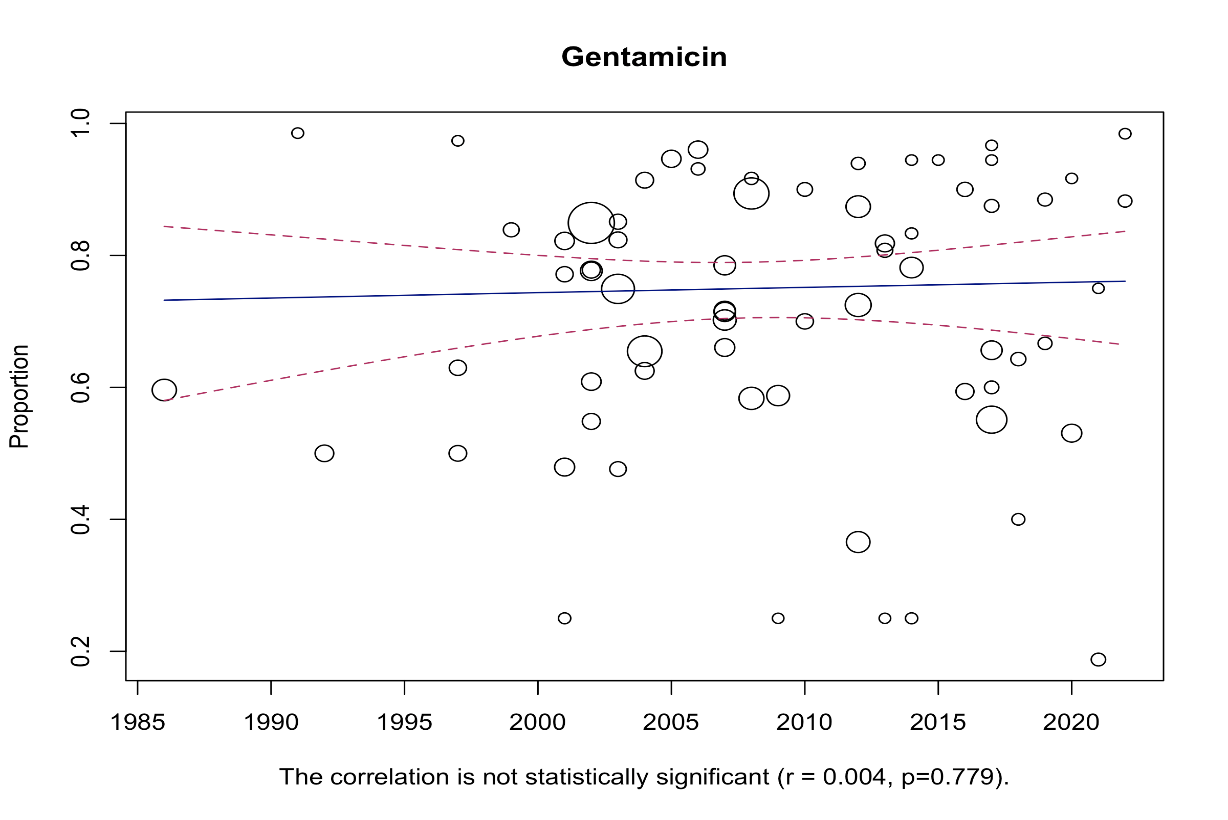


Figure 53. Bubble plot with fitted meta-regression for the year of publication.

Figure 54. The funnel plot of meta-analysis of publication studies. Each black dot represents a study. The white dots represent missing studies. The black line in the middle represents the average effect
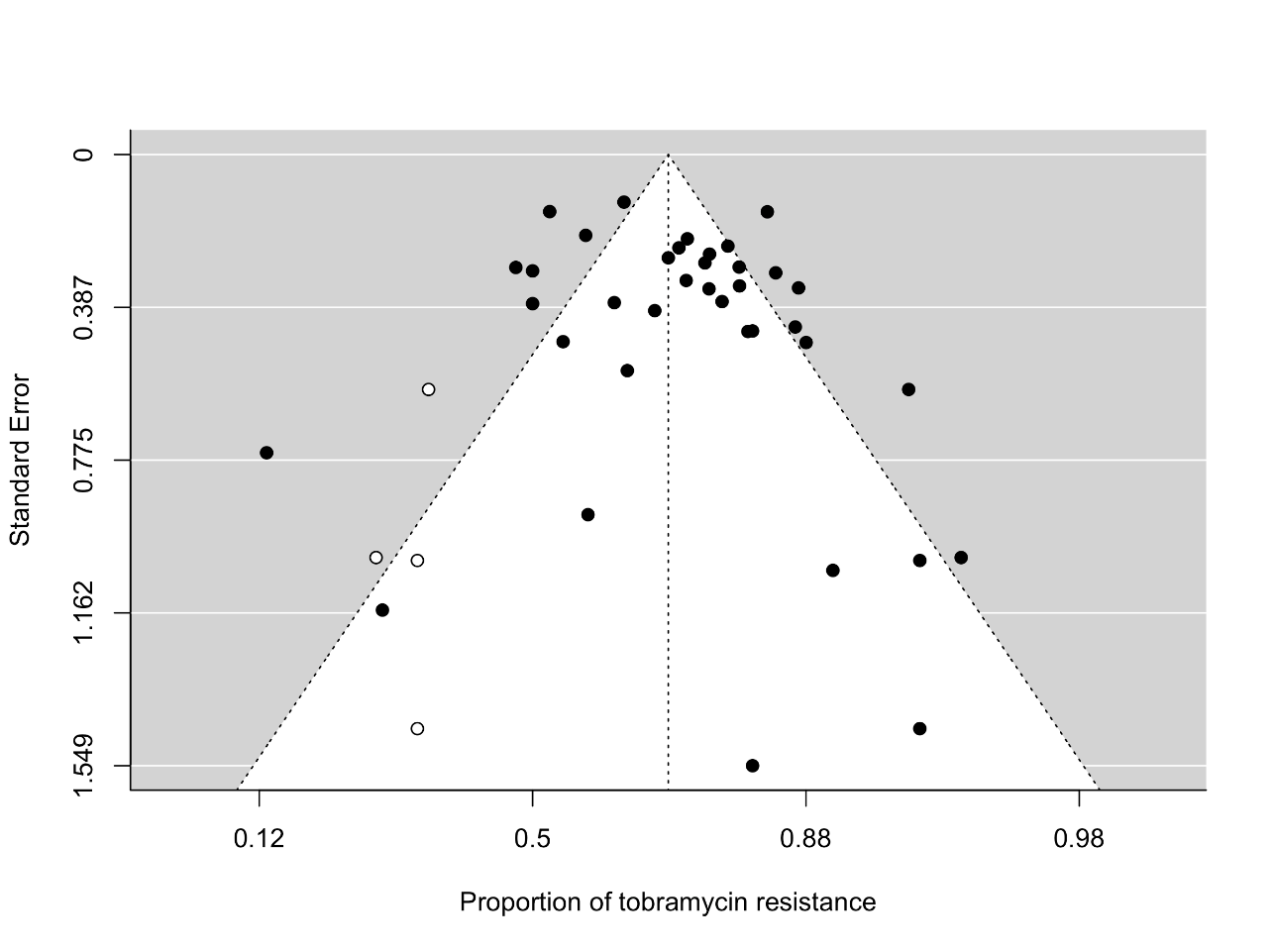
size.


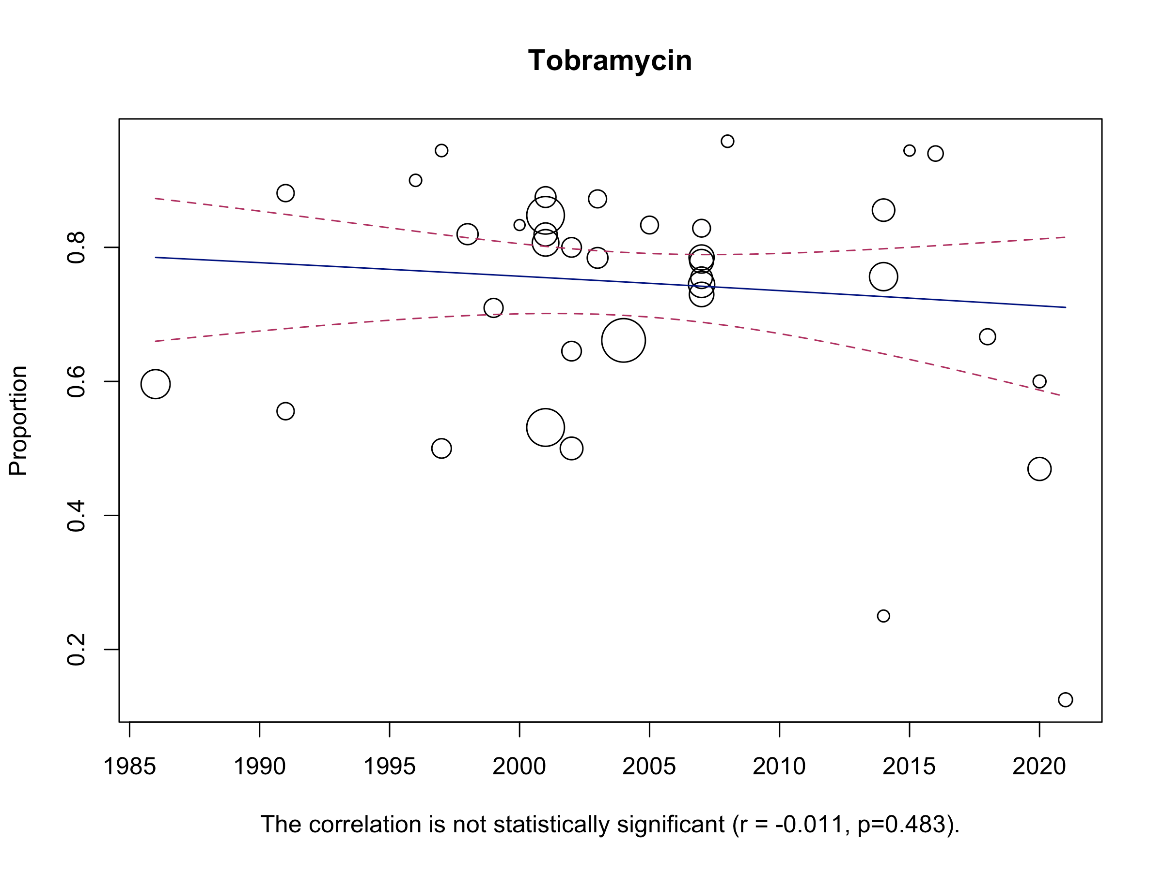


Figure 55. Bubble plot with fitted meta-regression for the year of publication.

Figure 56. The funnel plot of meta-analysis of publication studies. Each black dot represents a study. The white dots represent missing studies. The black line in the middle represents the average effect
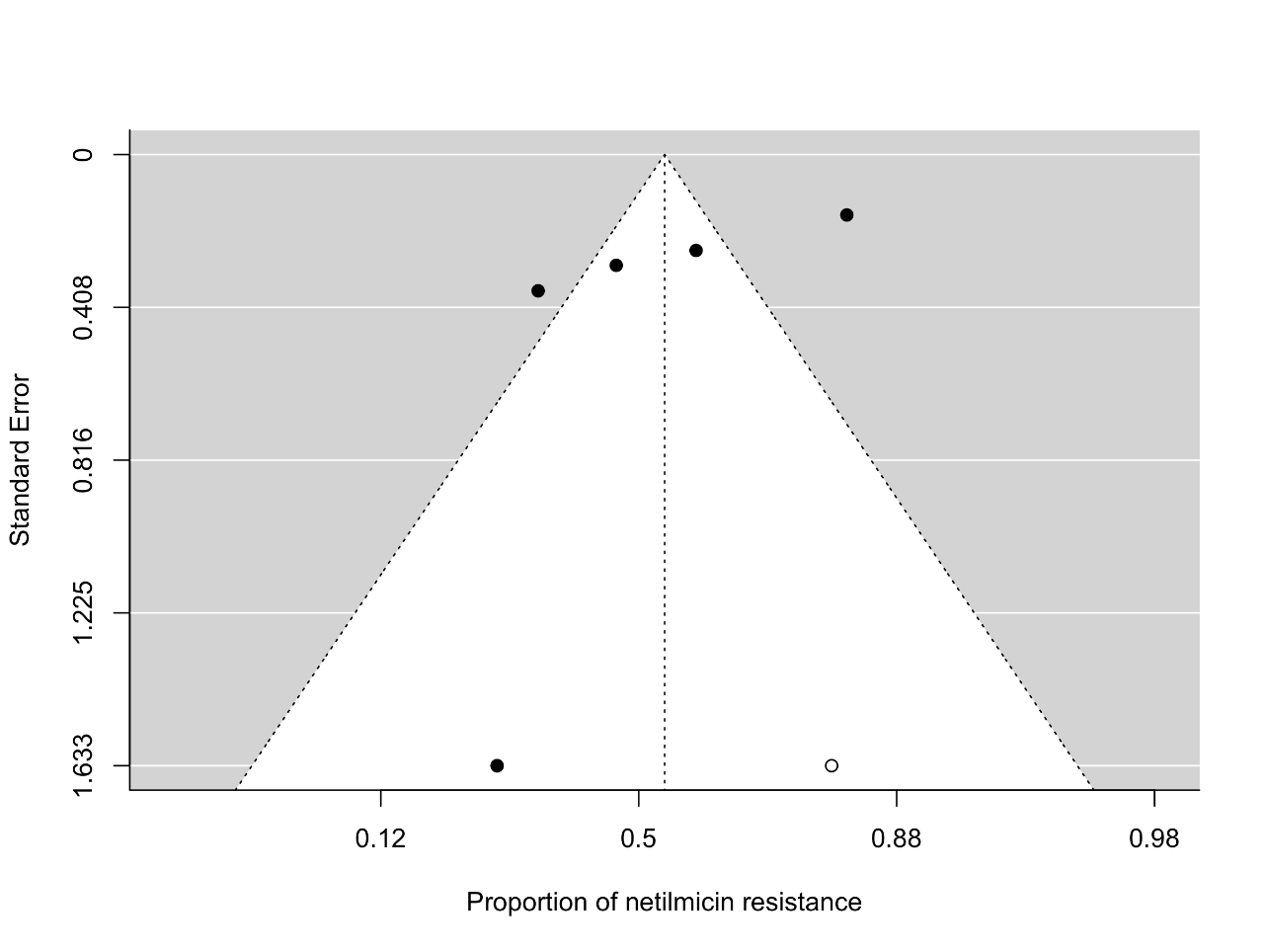
size.


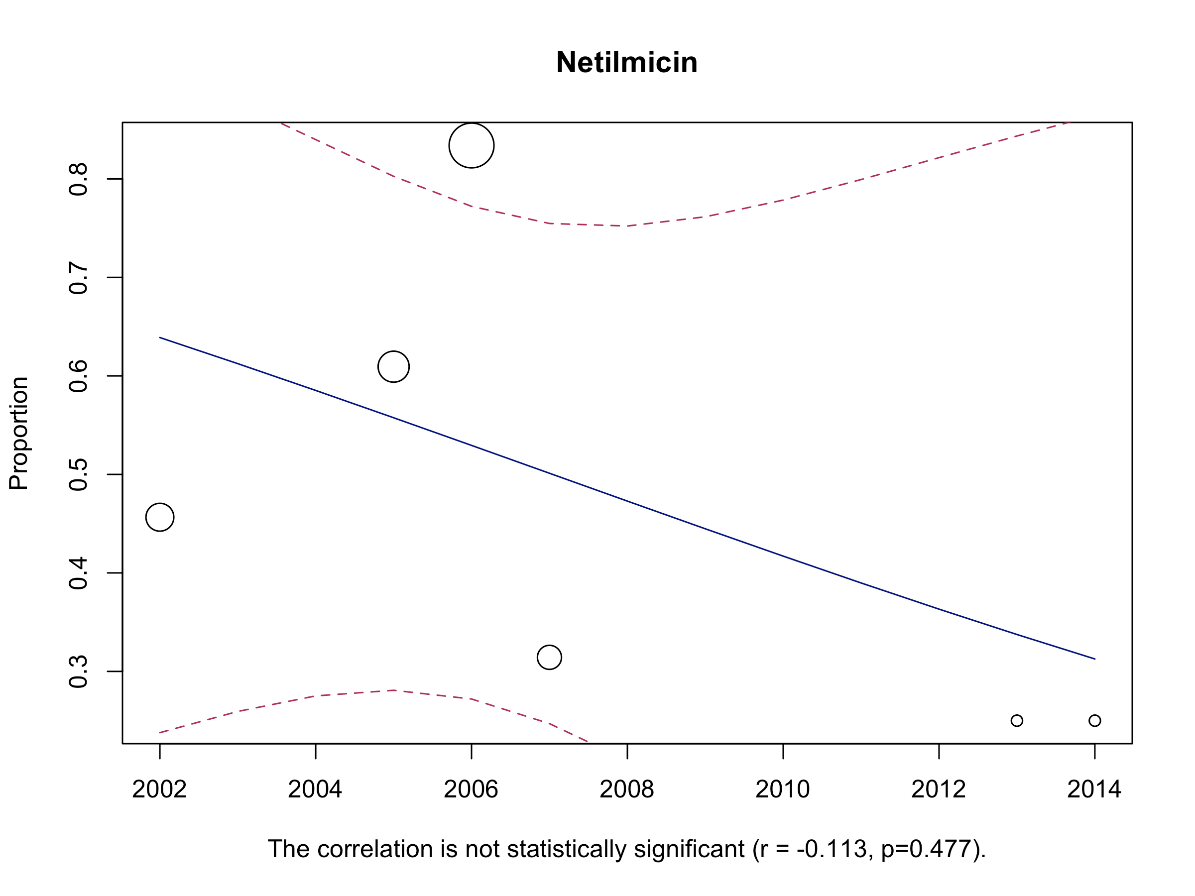


Figure 57. Bubble plot with fitted meta-regression for the year of publication.


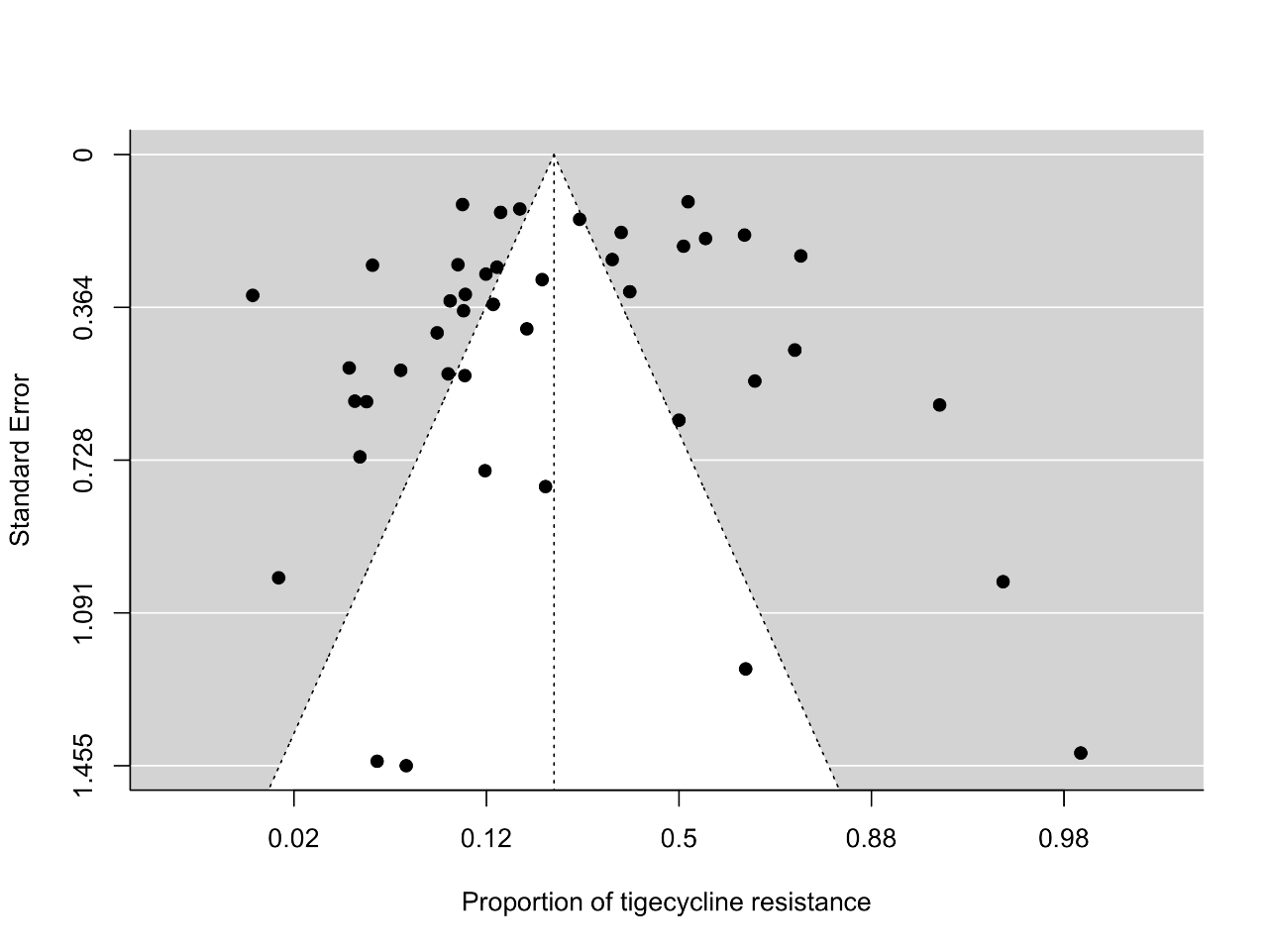
Figure 58. The funnel plot of meta-analysis of publication studies. Each black dot represents a study. The white dots represent missing studies. The black line in the middle represents the average effect size.

**Tetracyclines**


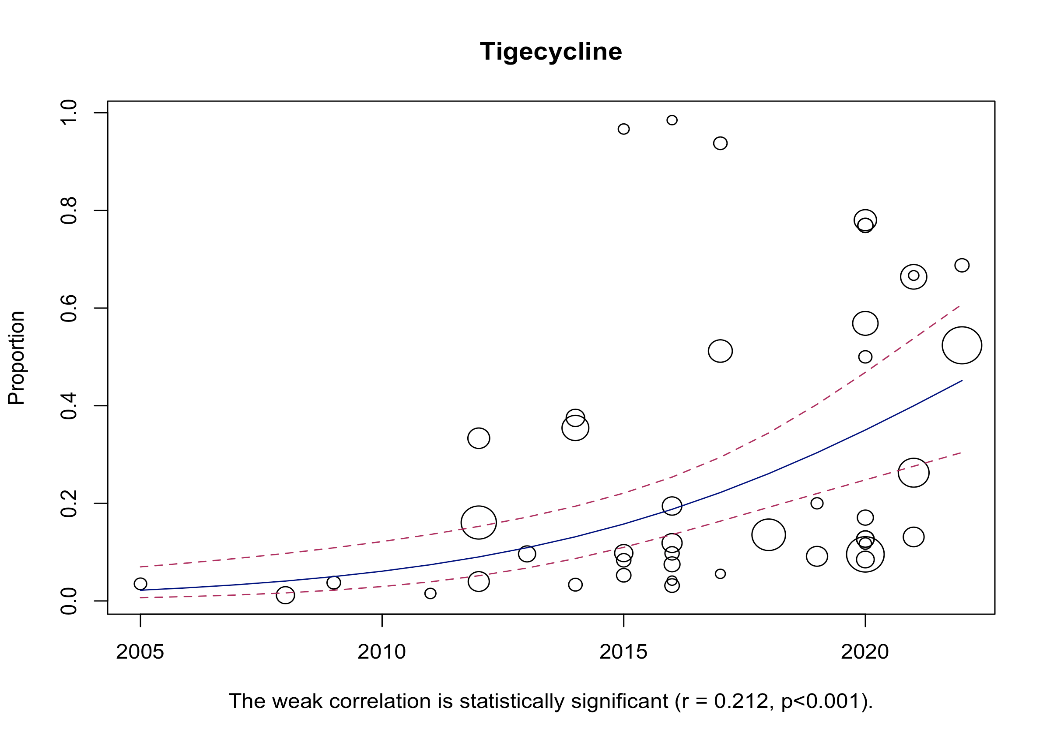


Figure 59. Bubble plot with fitted meta-regression for the year of publication.

Figure 60. The funnel plot of meta-analysis of publication studies. Each black dot represents a study. The white dots represent missing studies. The black line in the middle represents the average effect size
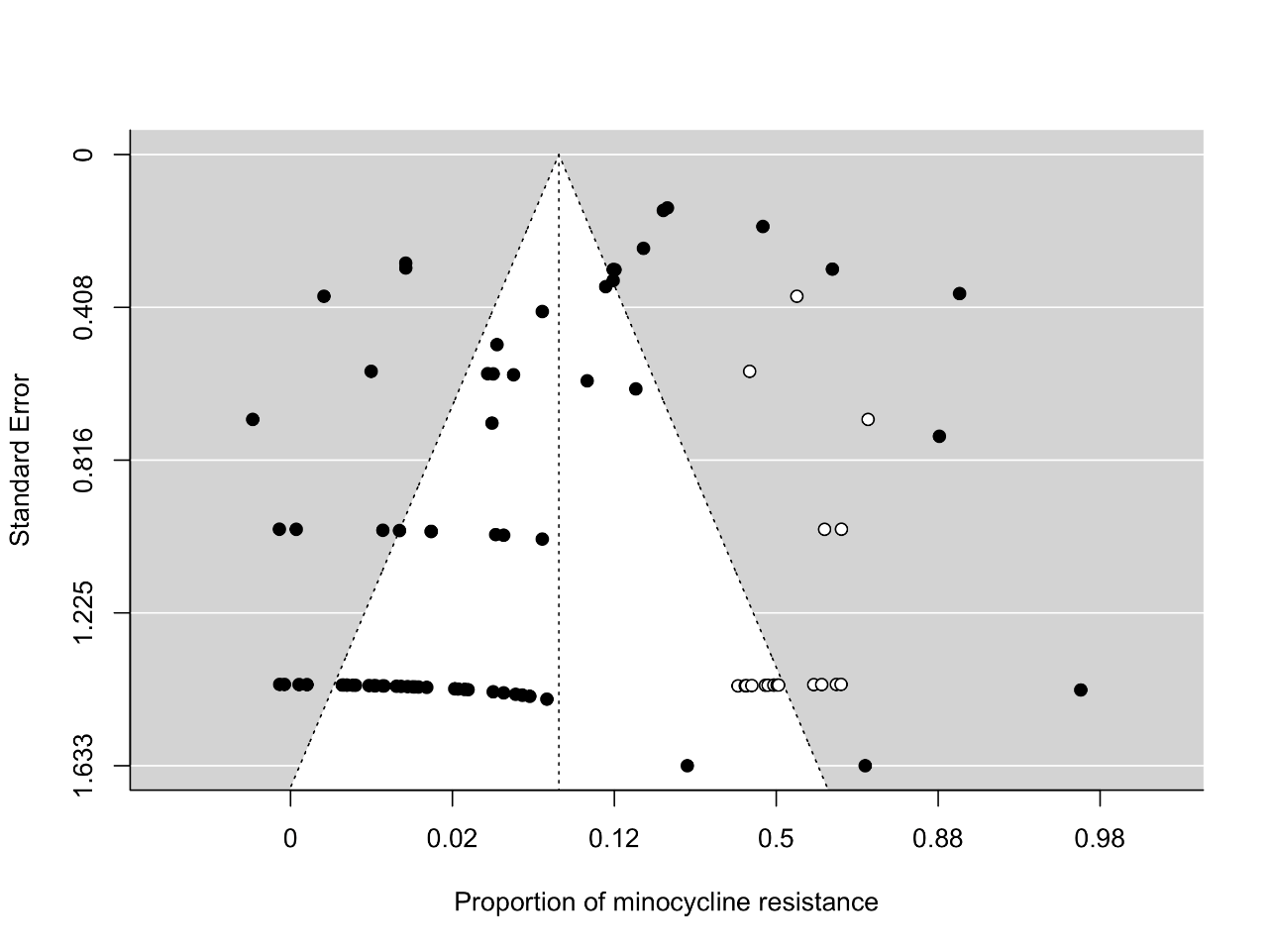


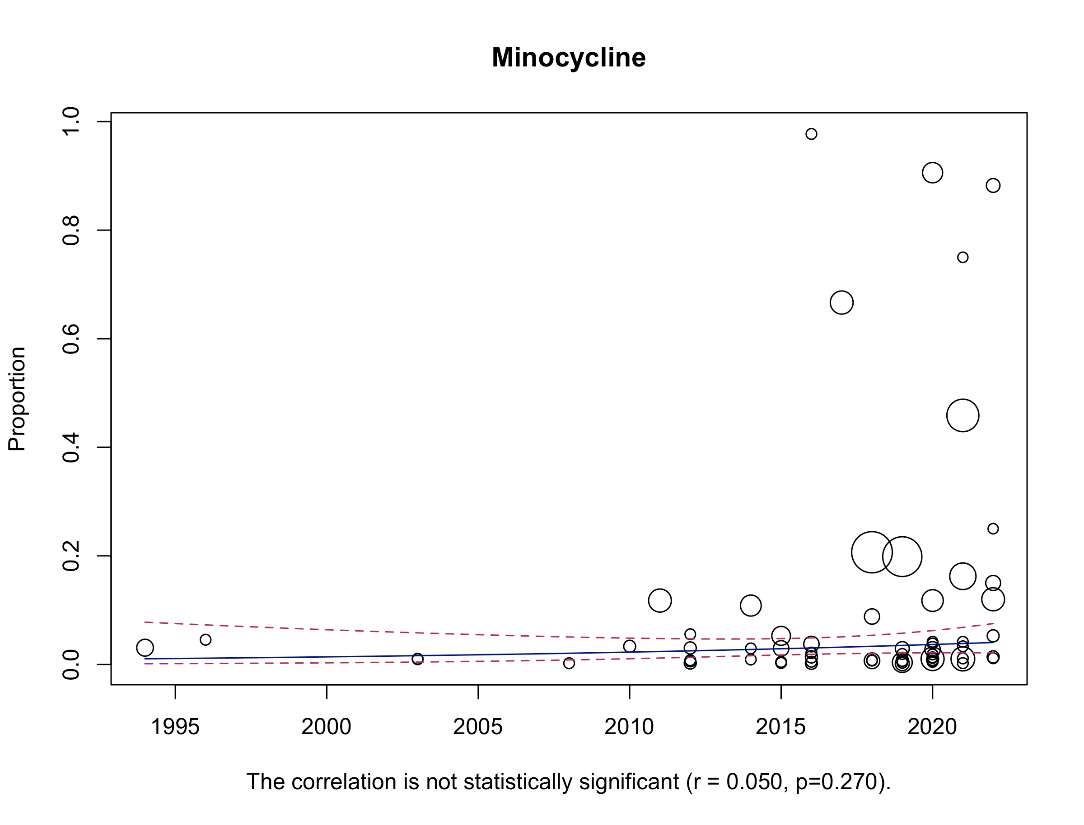


Figure 61. Bubble plot with fitted meta-regression for the year of publication


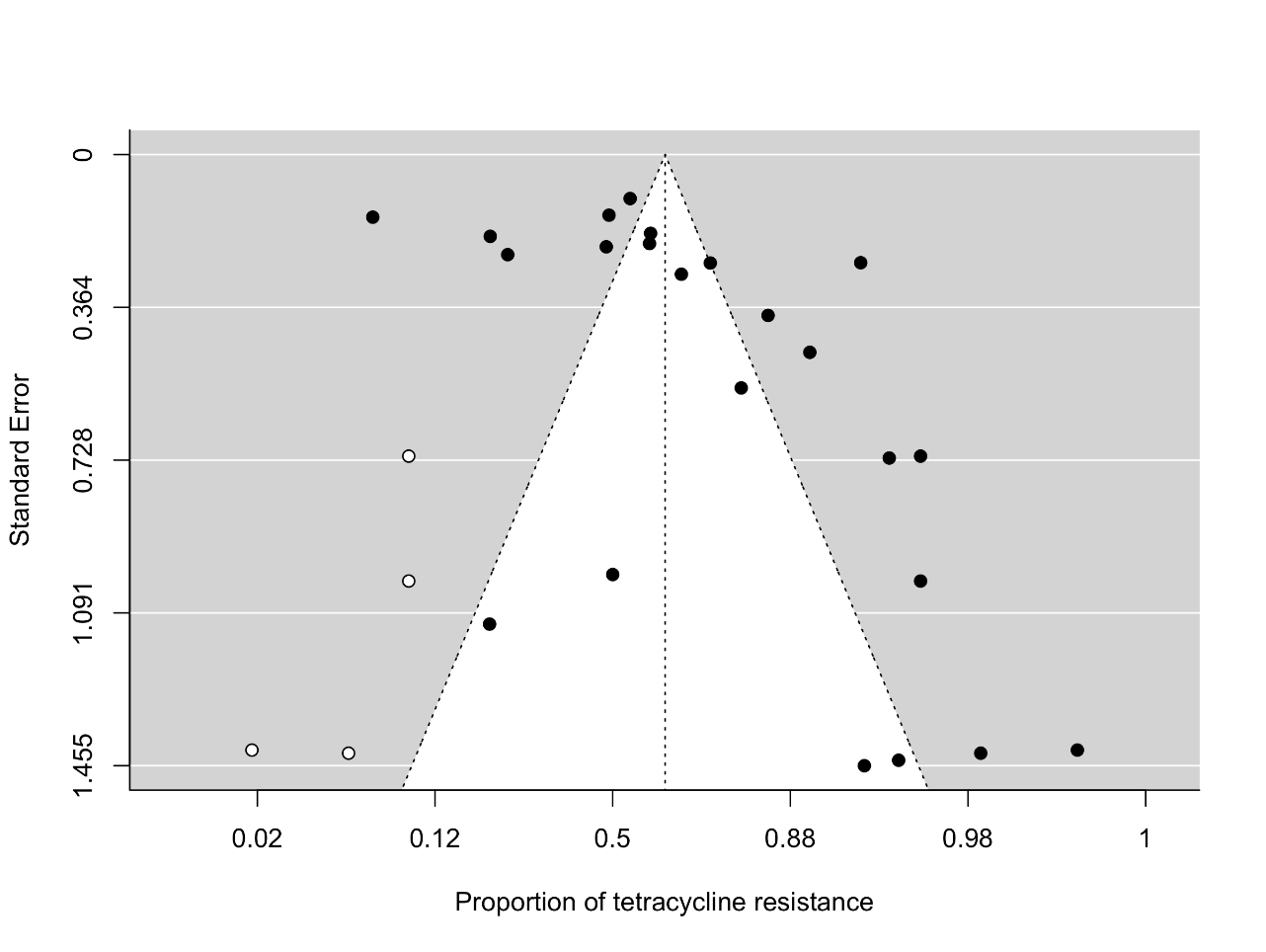


Figure 62. The funnel plot of meta-analysis of publication studies. Each black dot represents a study. The white dots represent missing studies. The black line in the middle represents the average effect size.


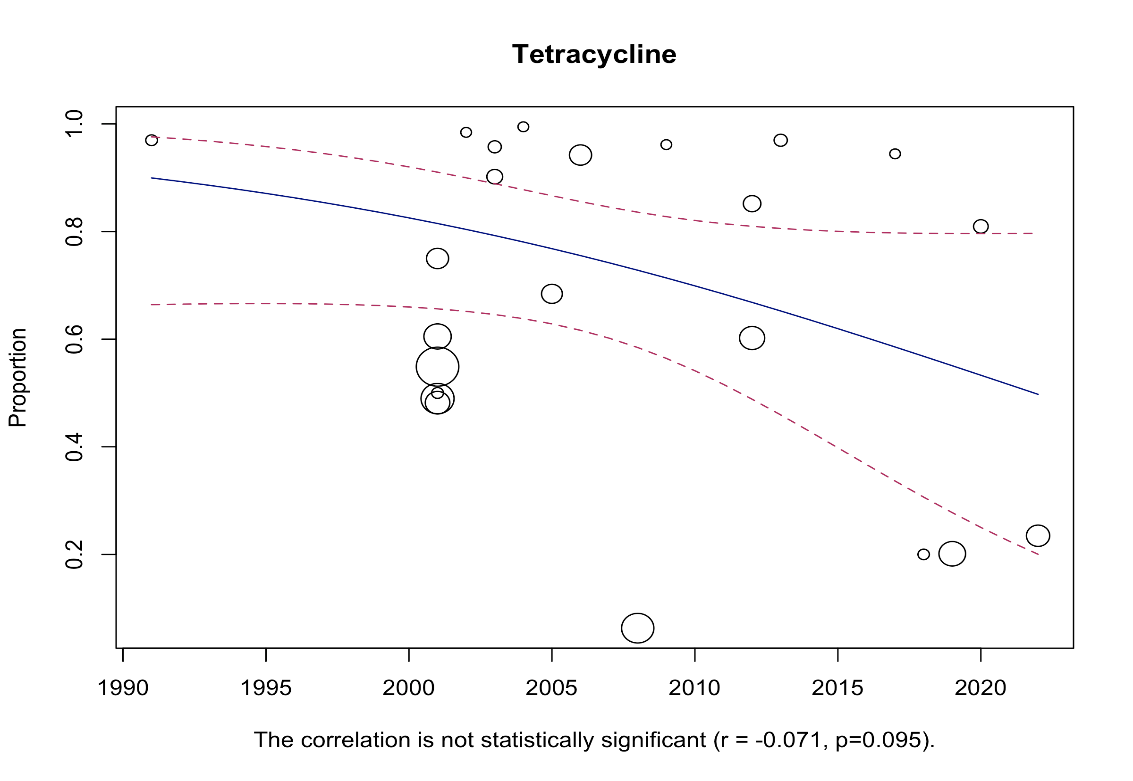


Figure 63. Bubble plot with fitted meta-regression for the year of publication


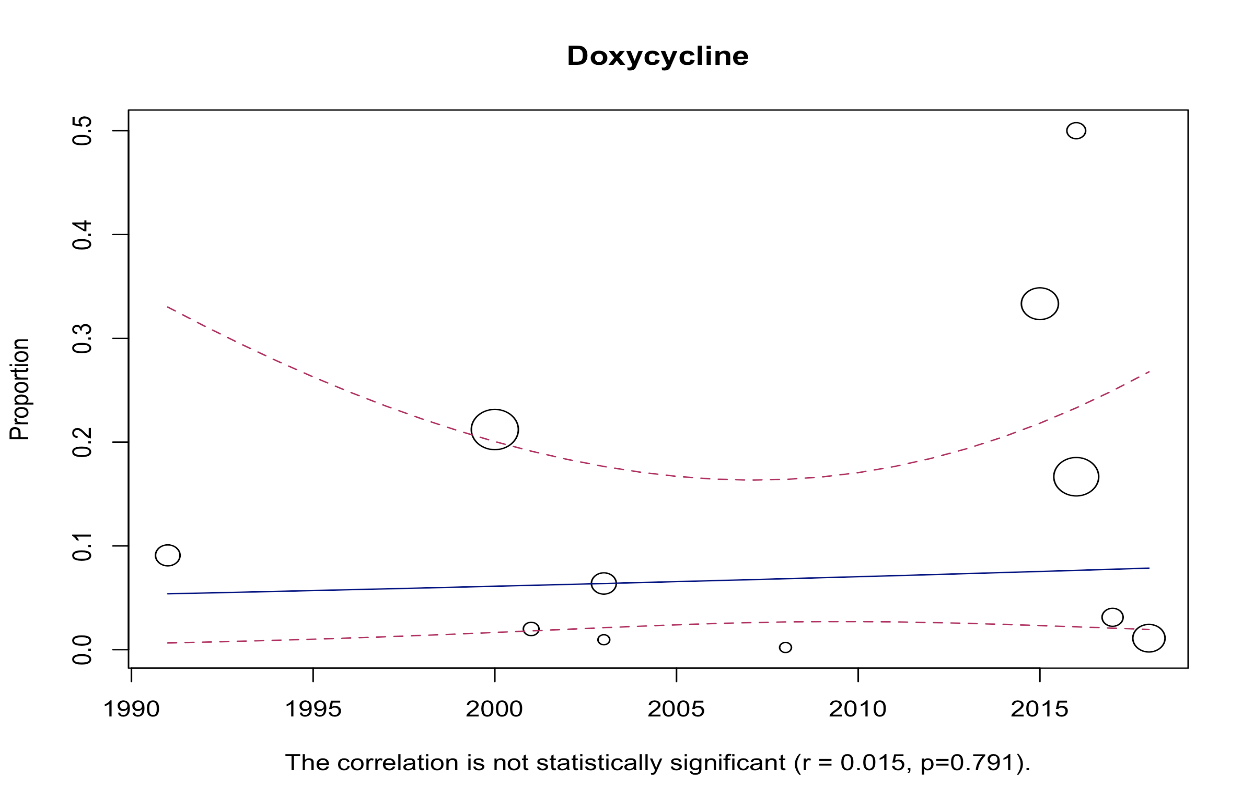

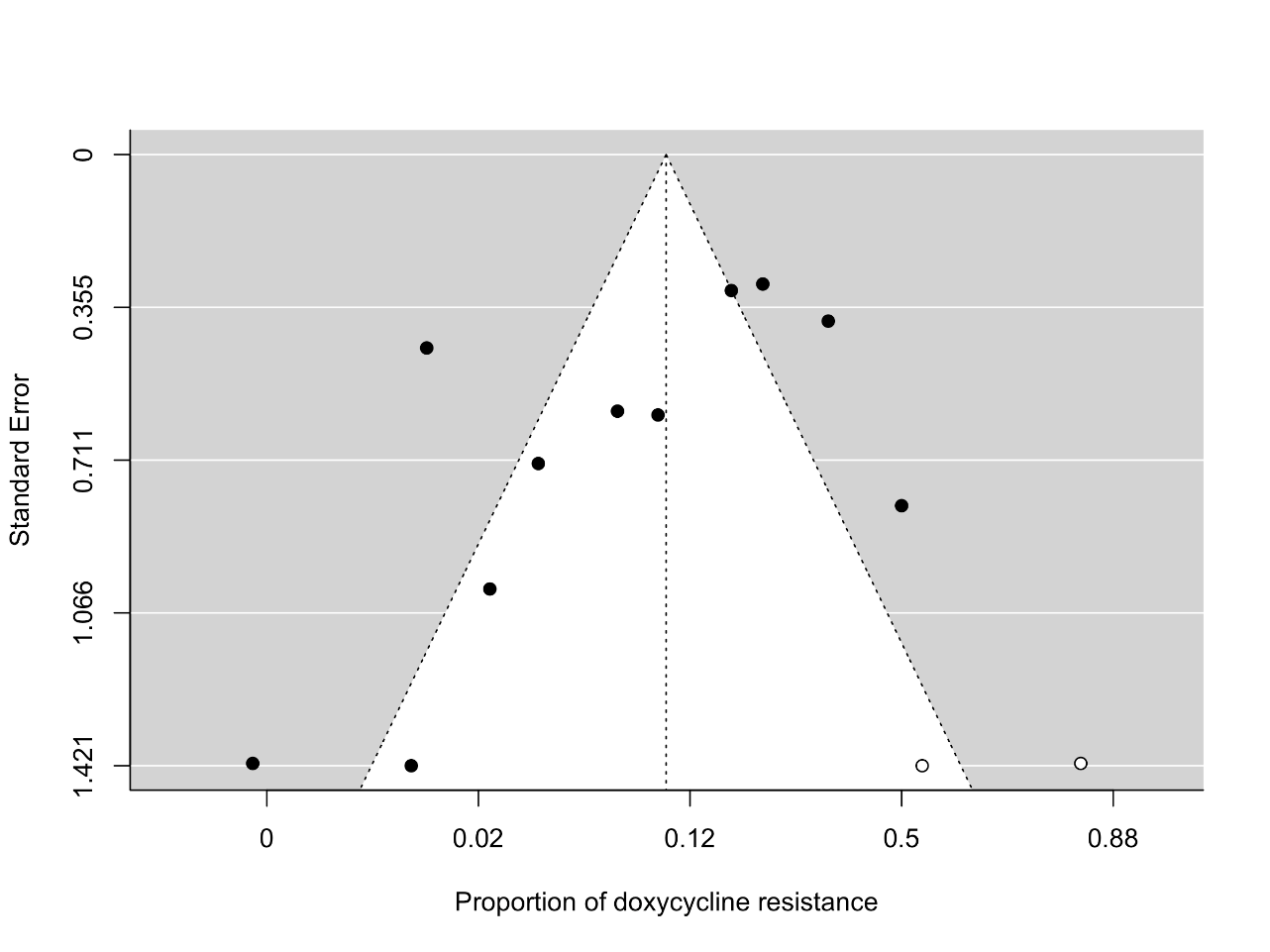
Figure 64. The funnel plot of meta-analysis of publication studies. Each black dot represents a study. The white dots represent missing studies. The black line in the middle represents the average effect size

Figure 65. Bubble plot with fitted meta-regression for the year of publication


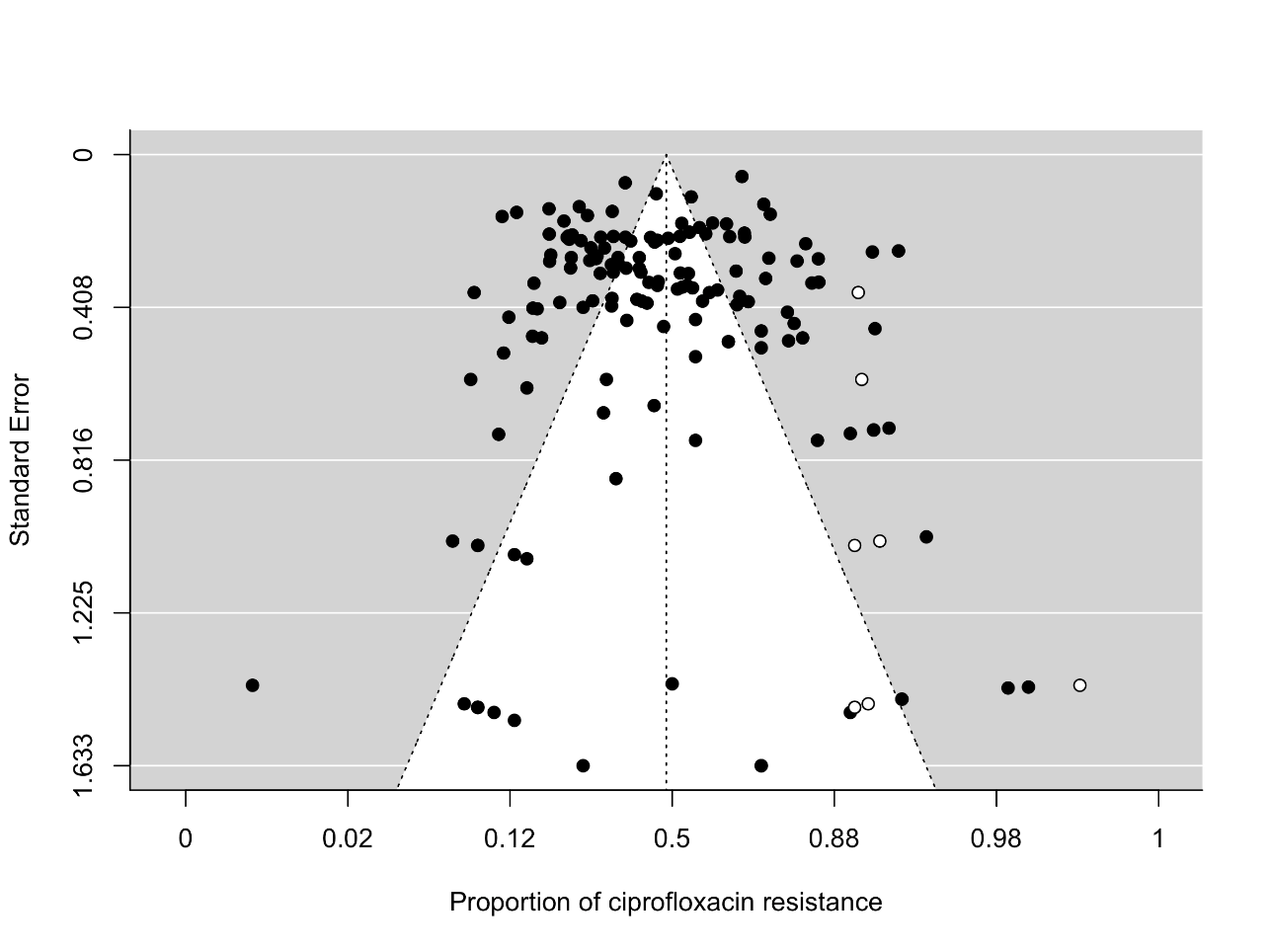


**Fluoroquinolones**

Figure 66. The funnel plot of meta-analysis of publication studies. Each black dot represents a study. The white dots represent missing studies. The black line in the middle represents the average effect size.


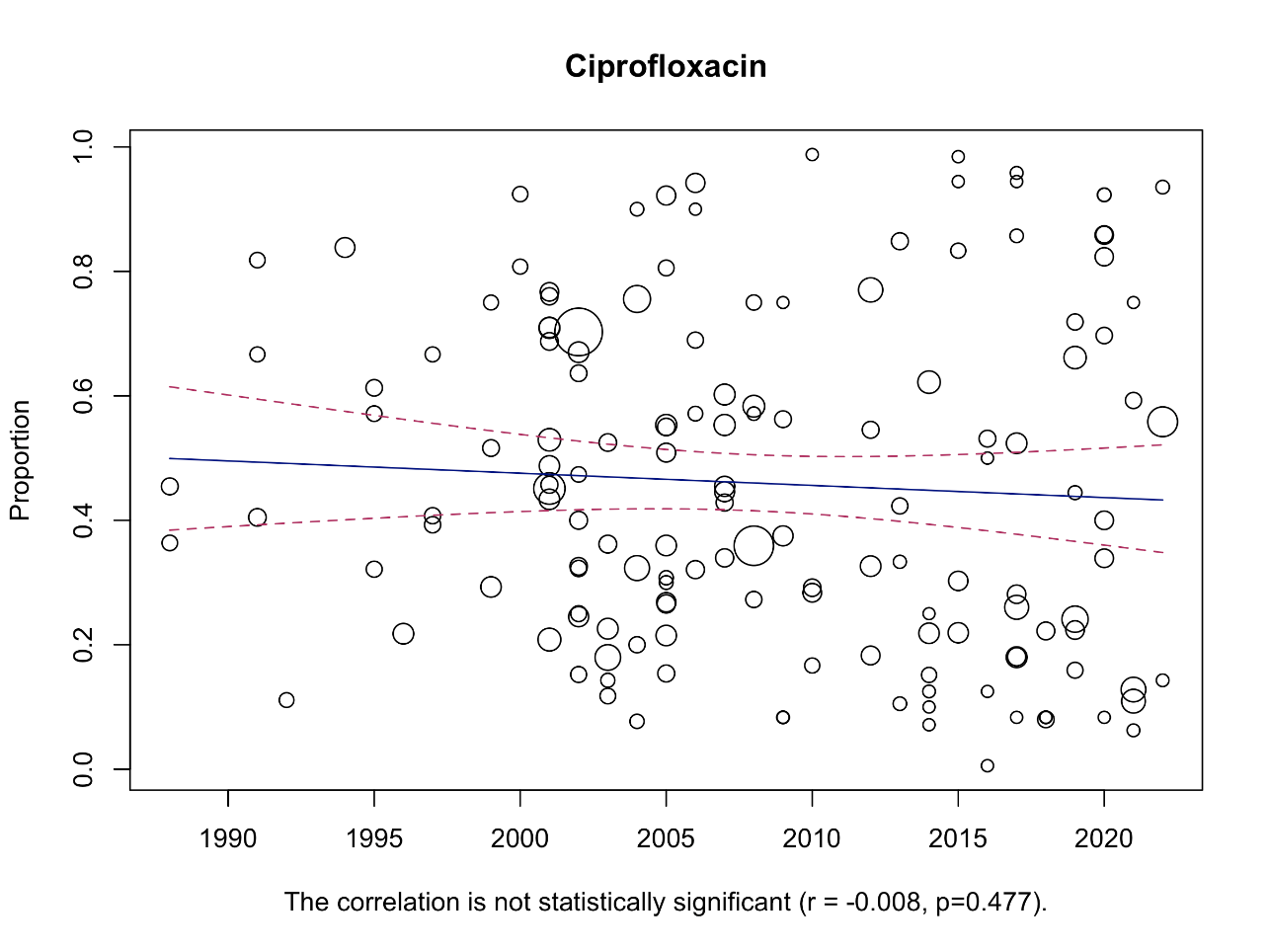


Figure 67. Bubble plot with fitted meta-regression for the year of publication


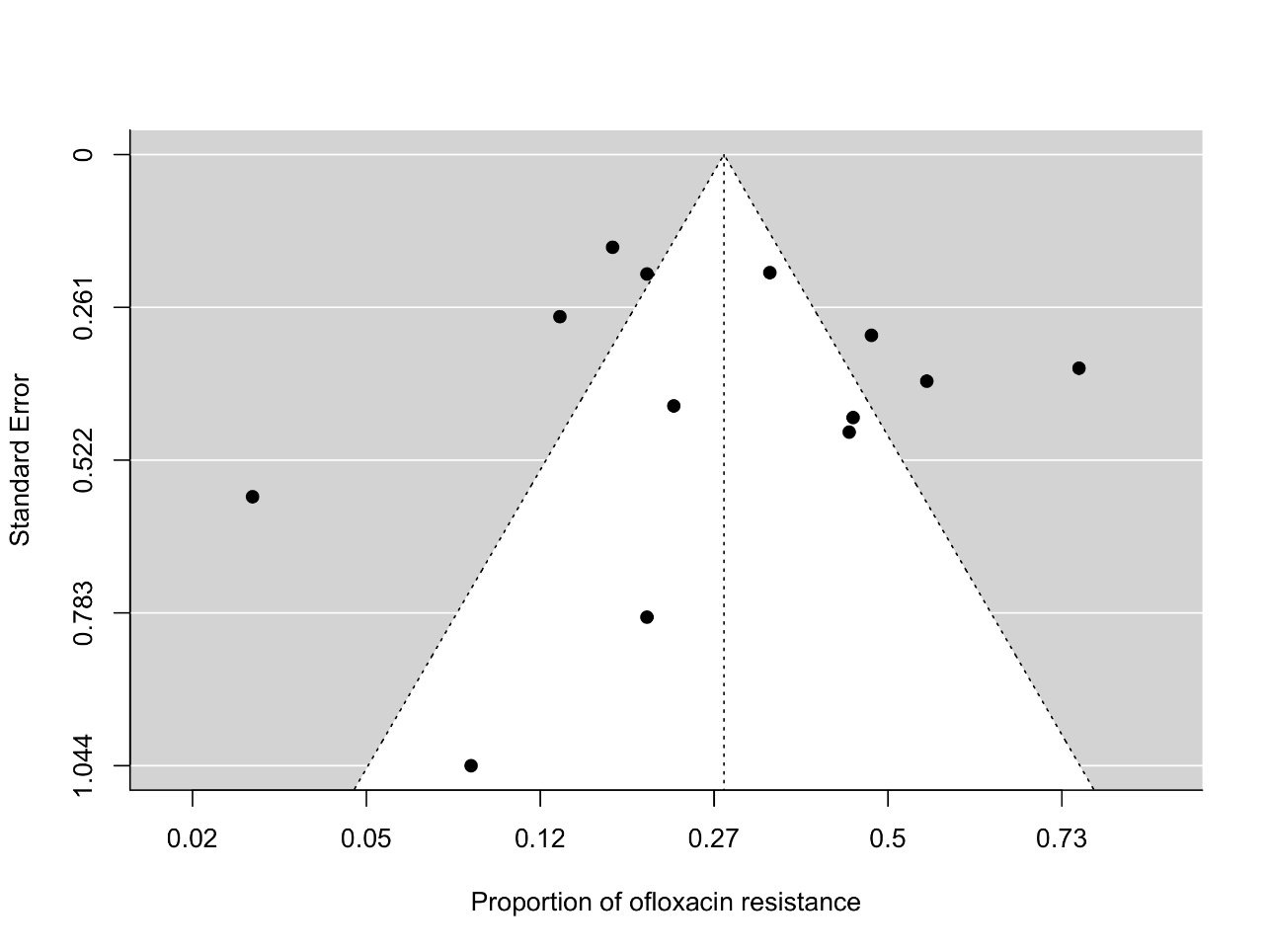
Figure 68. The funnel plot of meta-analysis of publication studies. Each black dot represents a study. The black line in the middle represents the average effect size


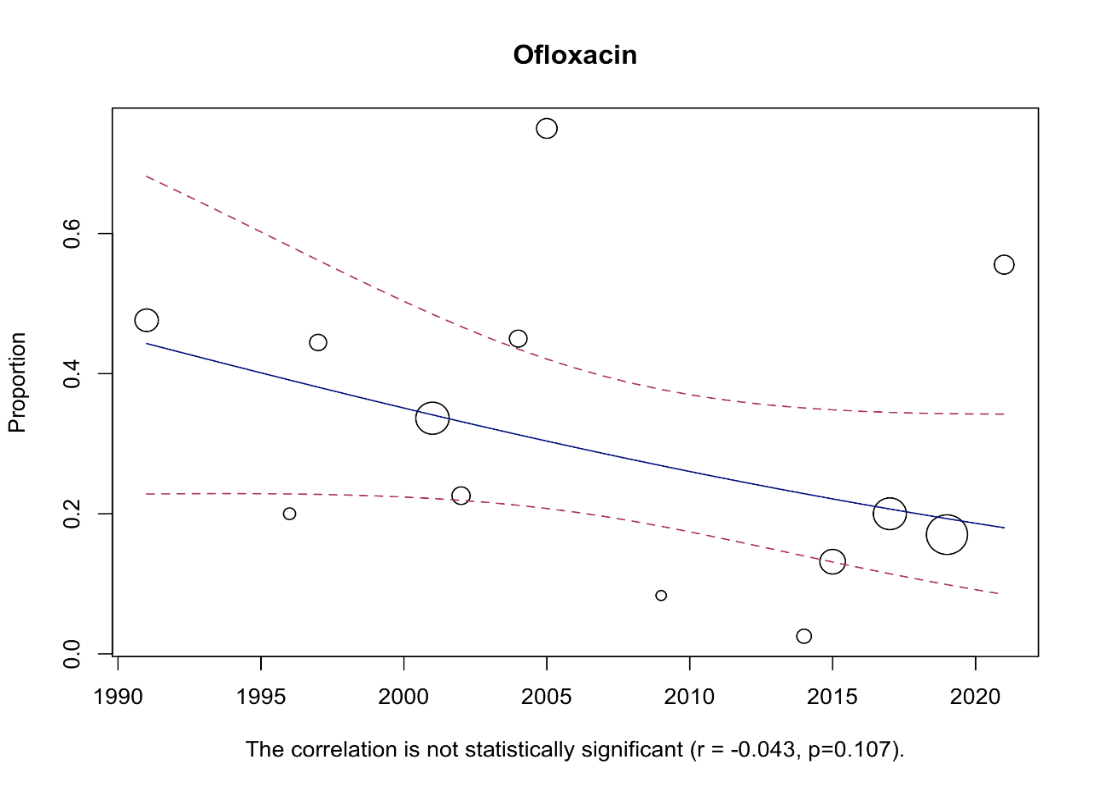


Figure 69. Bubble plot with fitted meta-regression for the year of publication


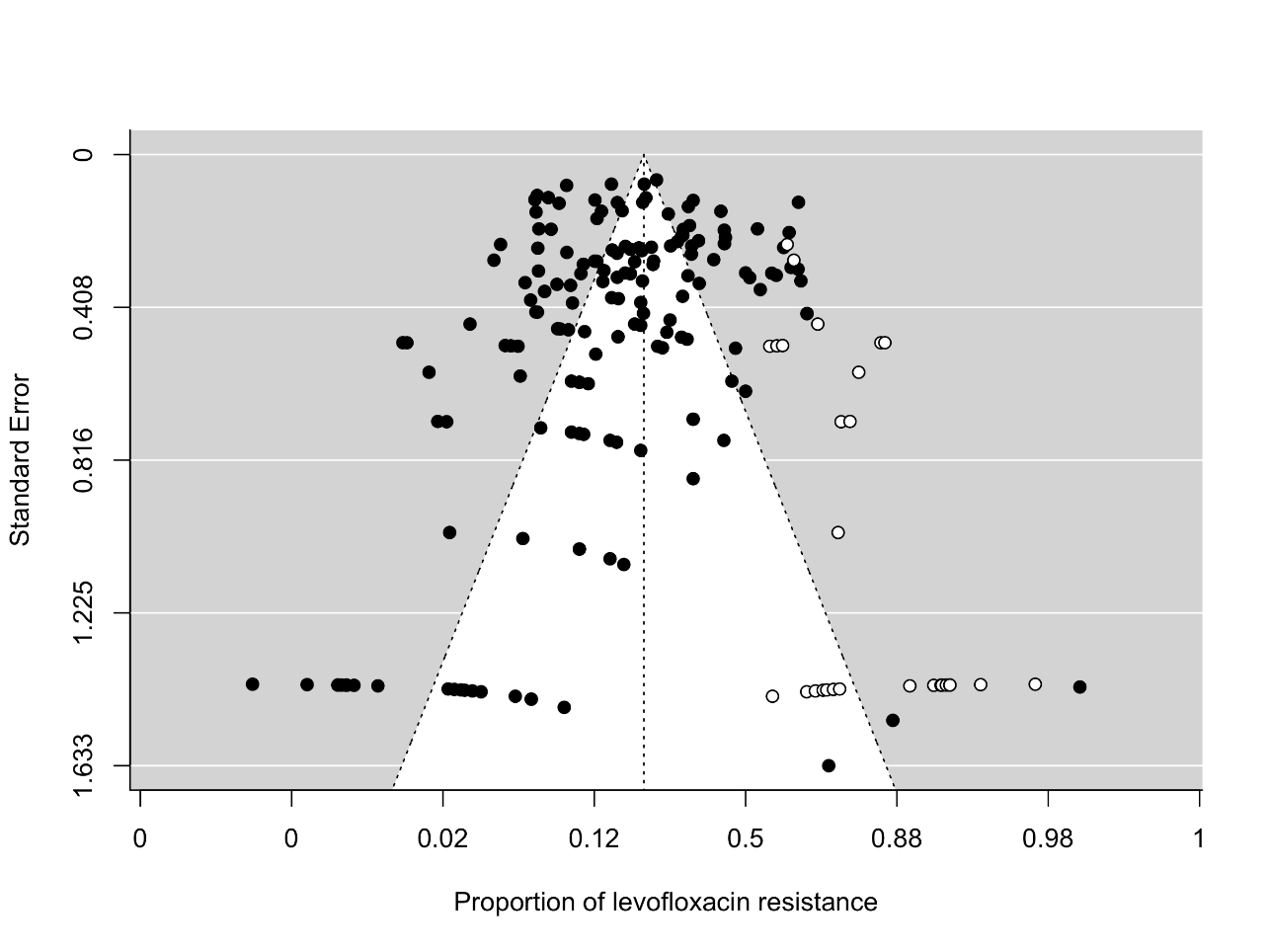


Figure 70. The funnel plot of meta-analysis of publication studies. Each black dot represents a study. The white dots represent missing studies. The black line in the middle represents the average effect size.


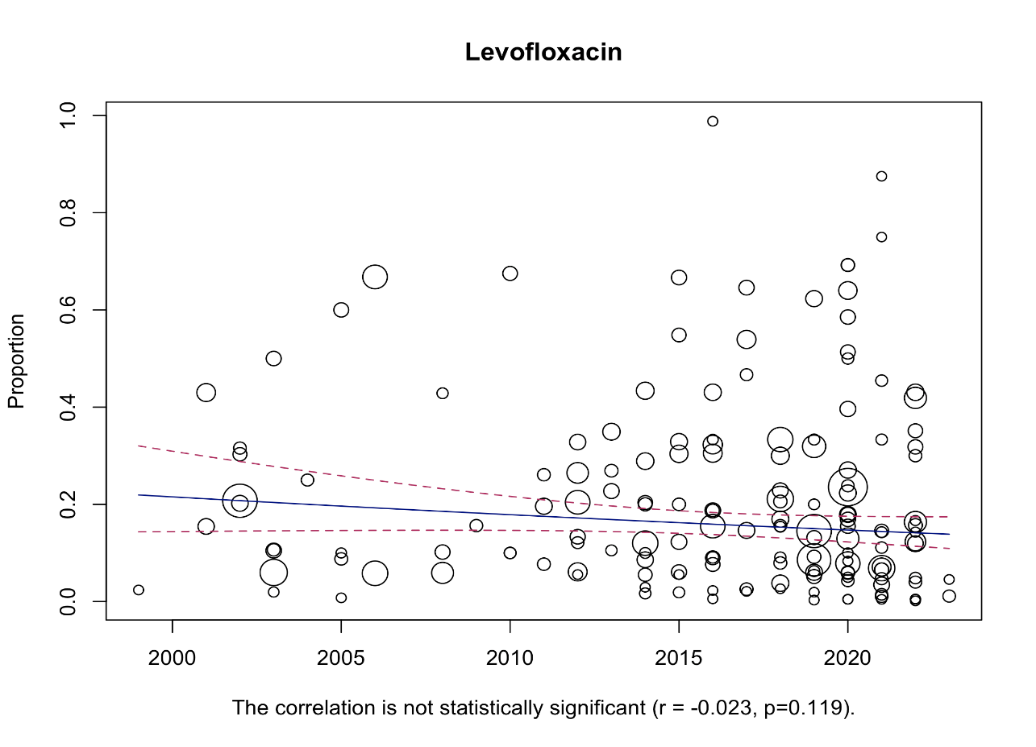


Figure 71. Bubble plot with fitted meta-regression for the year of publication


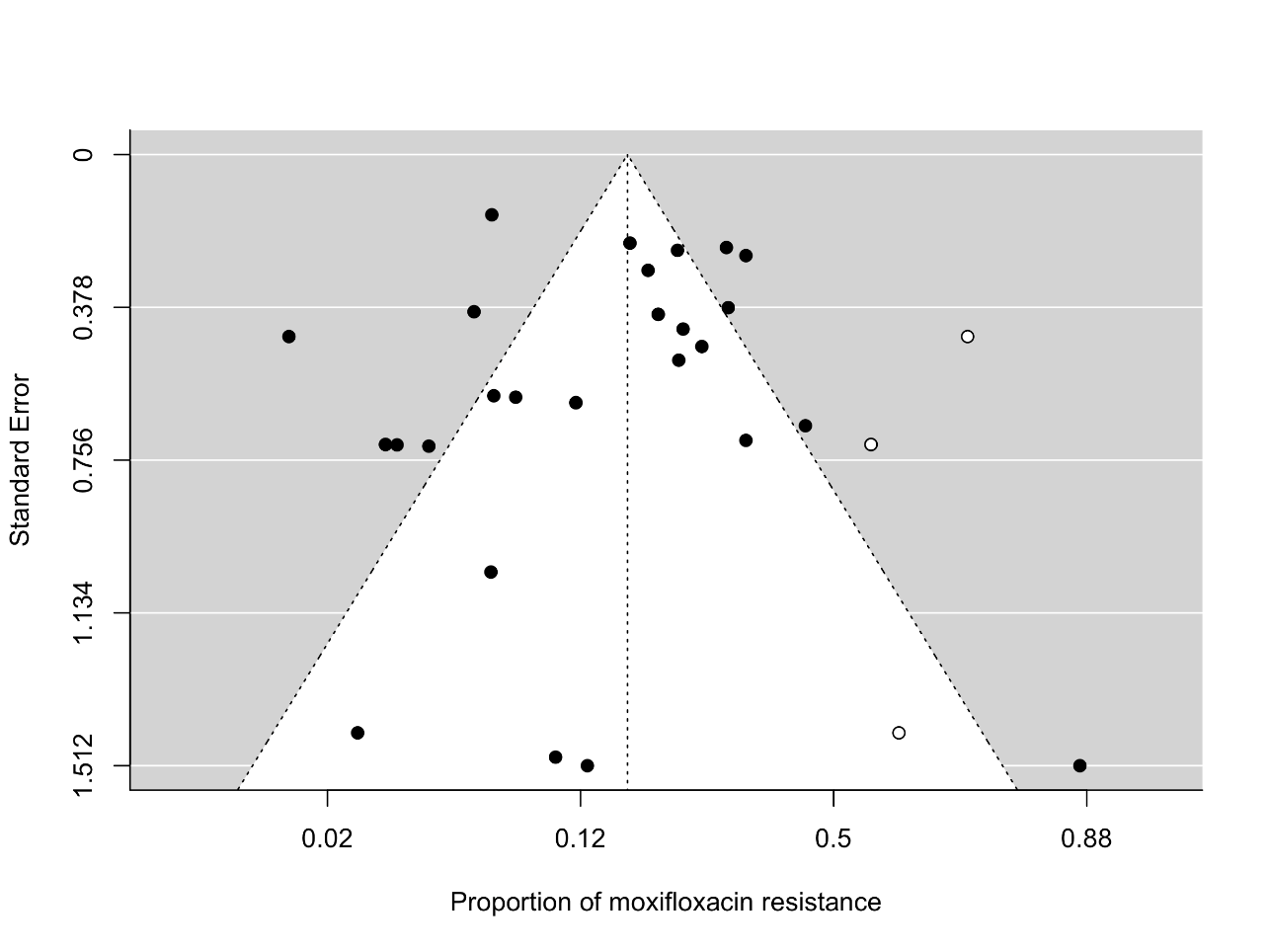


Figure 72. The funnel plot of meta-analysis of publication studies. Each black dot represents a study. The white dots represent missing studies. The black line in the middle represents the average effect size.


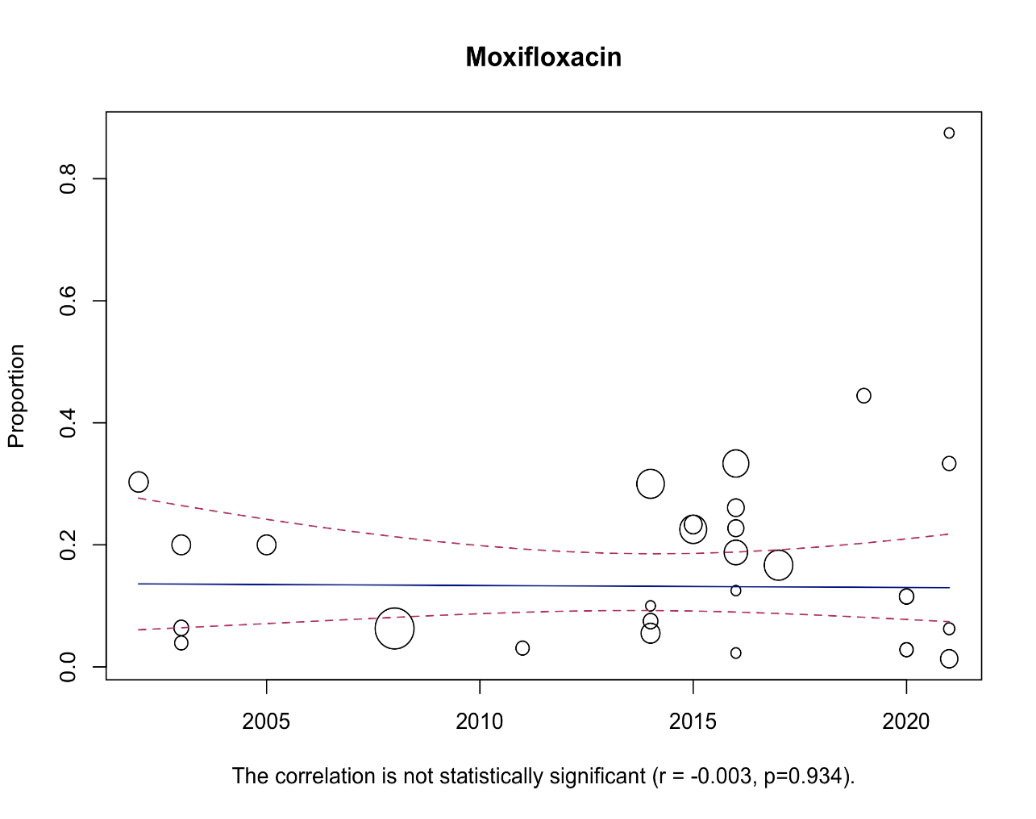


Figure 73. Bubble plot with fitted meta-regression for the year of publication


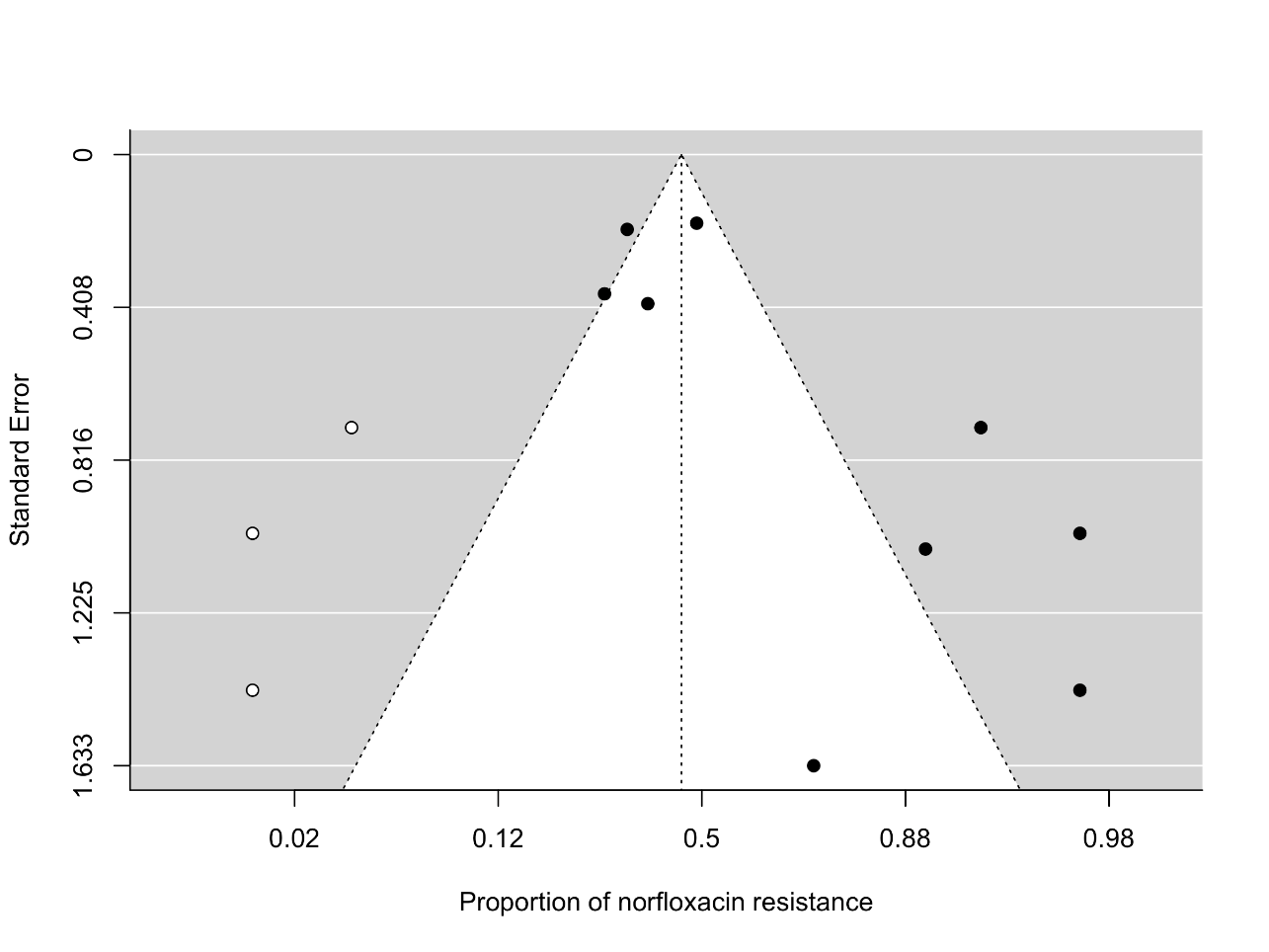
Figure 74. The funnel plot of meta-analysis of publication studies. Each black dot represents a study. The white dots represent missing studies. The black line in the middle represents the average effect size.


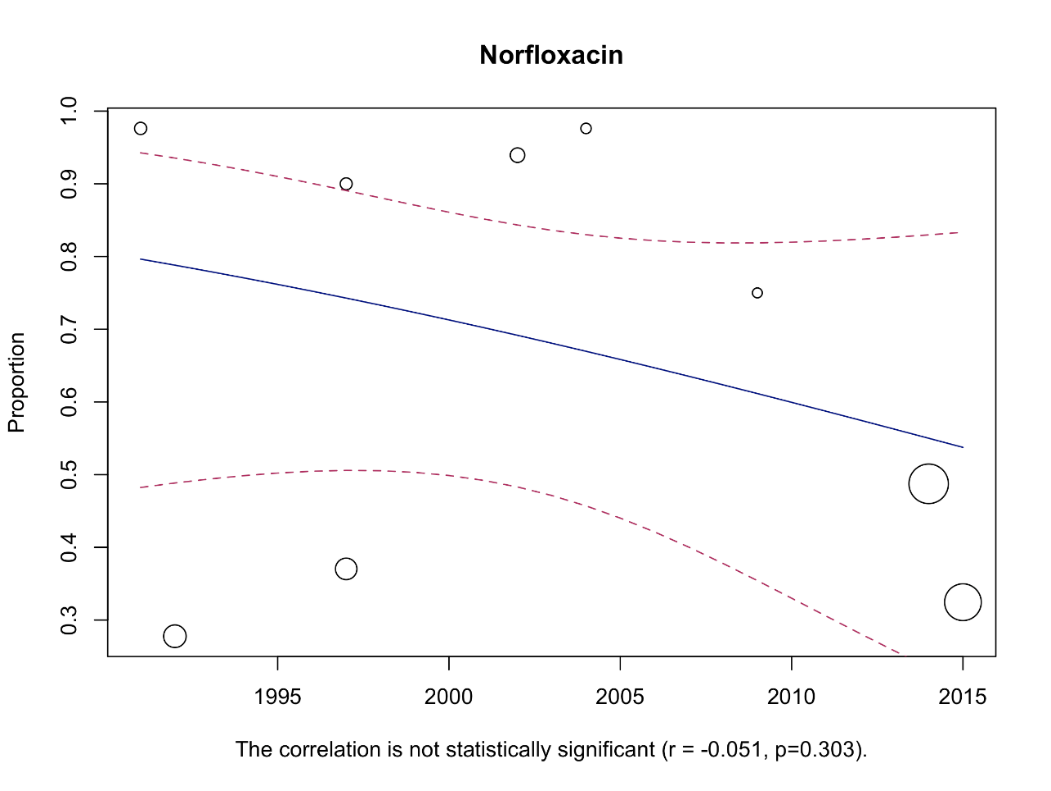


Figure 75. Bubble plot with fitted meta-regression for the year of publication

Figure 76. The funnel plot of meta-analysis of publication studies. Each black dot represents a study. The white dots represent missing studies. The black line in the middle represents the average effect size.
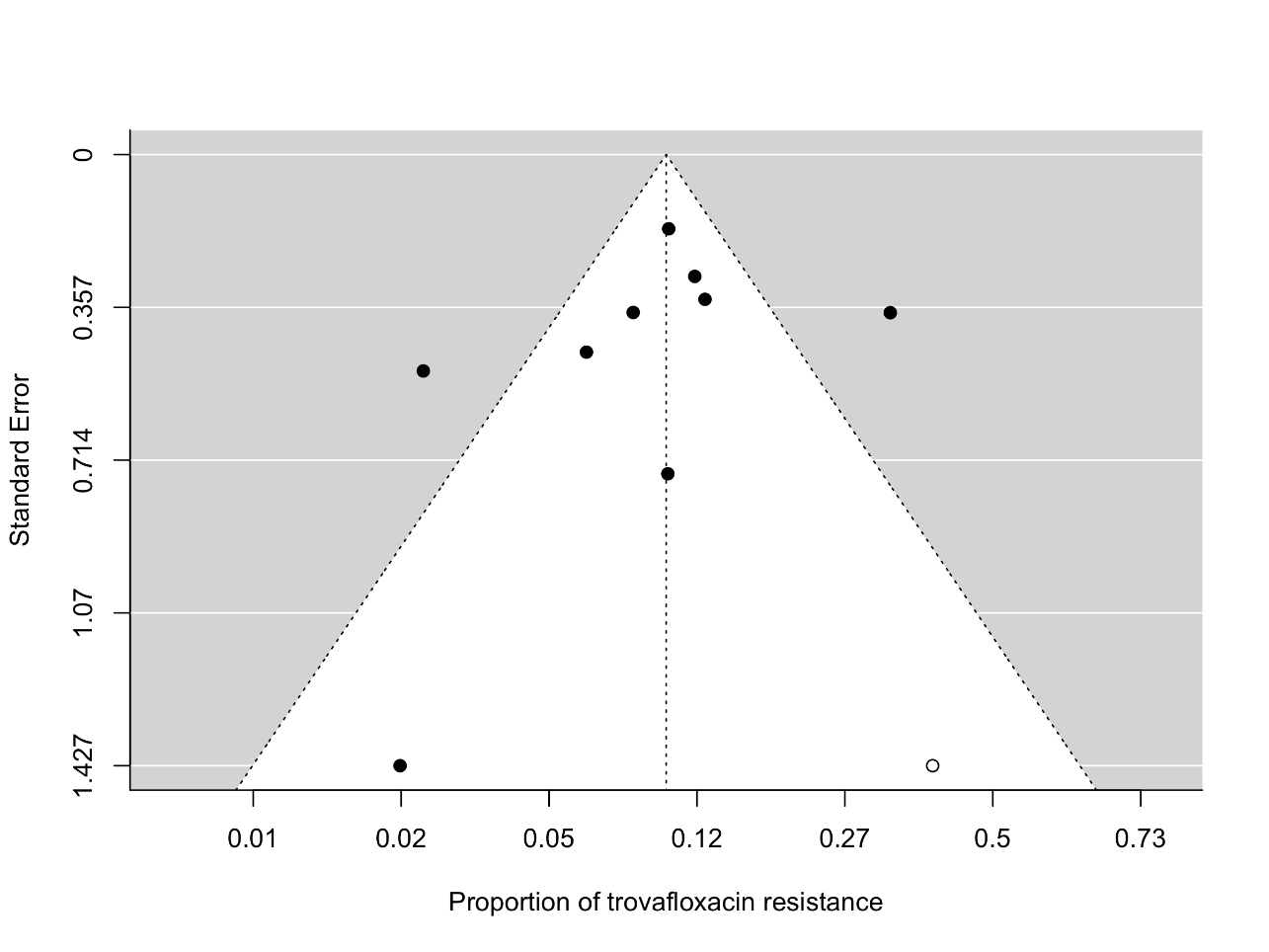


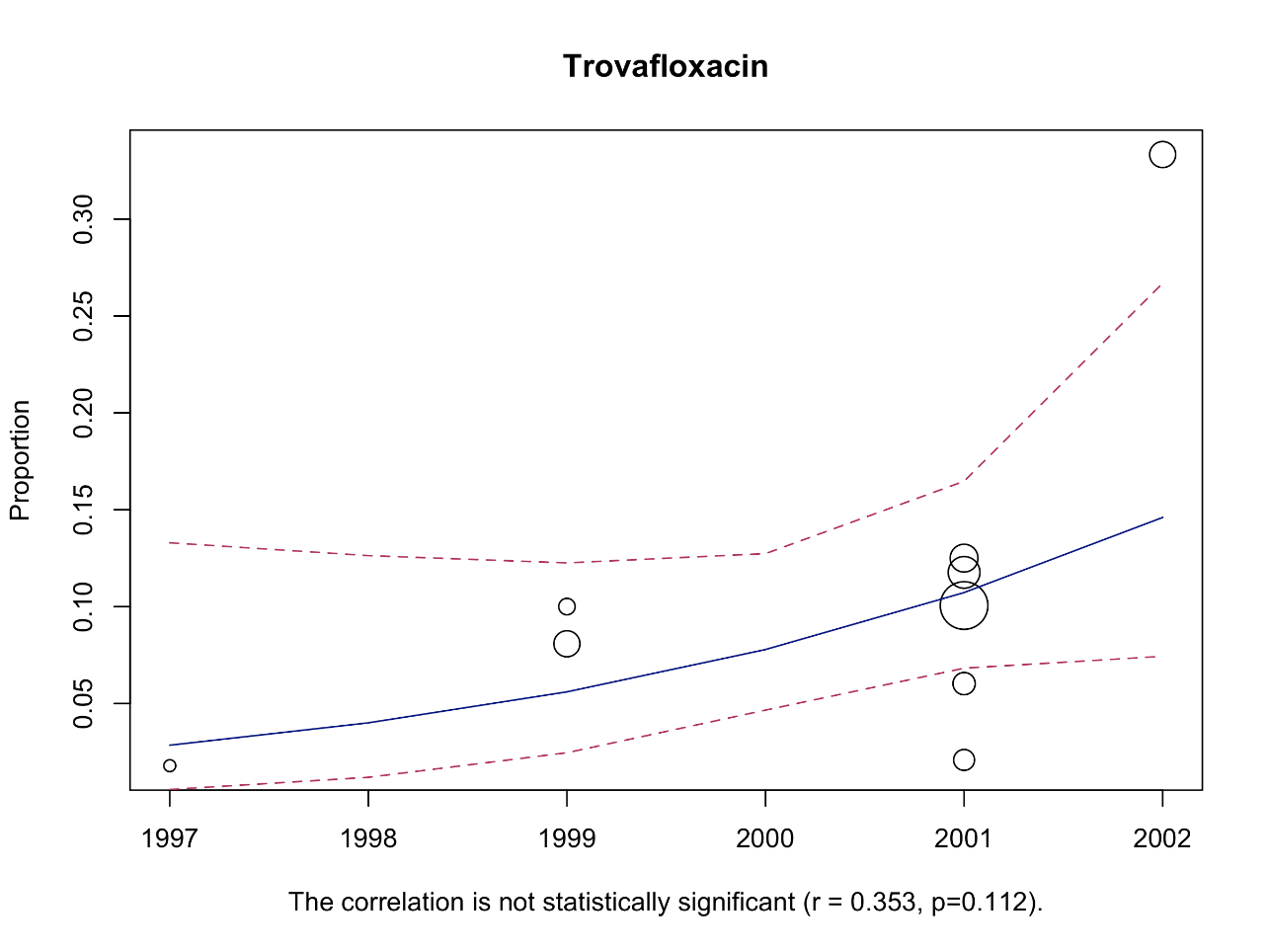


Figure 77. Bubble plot with fitted meta-regression for the year of publication


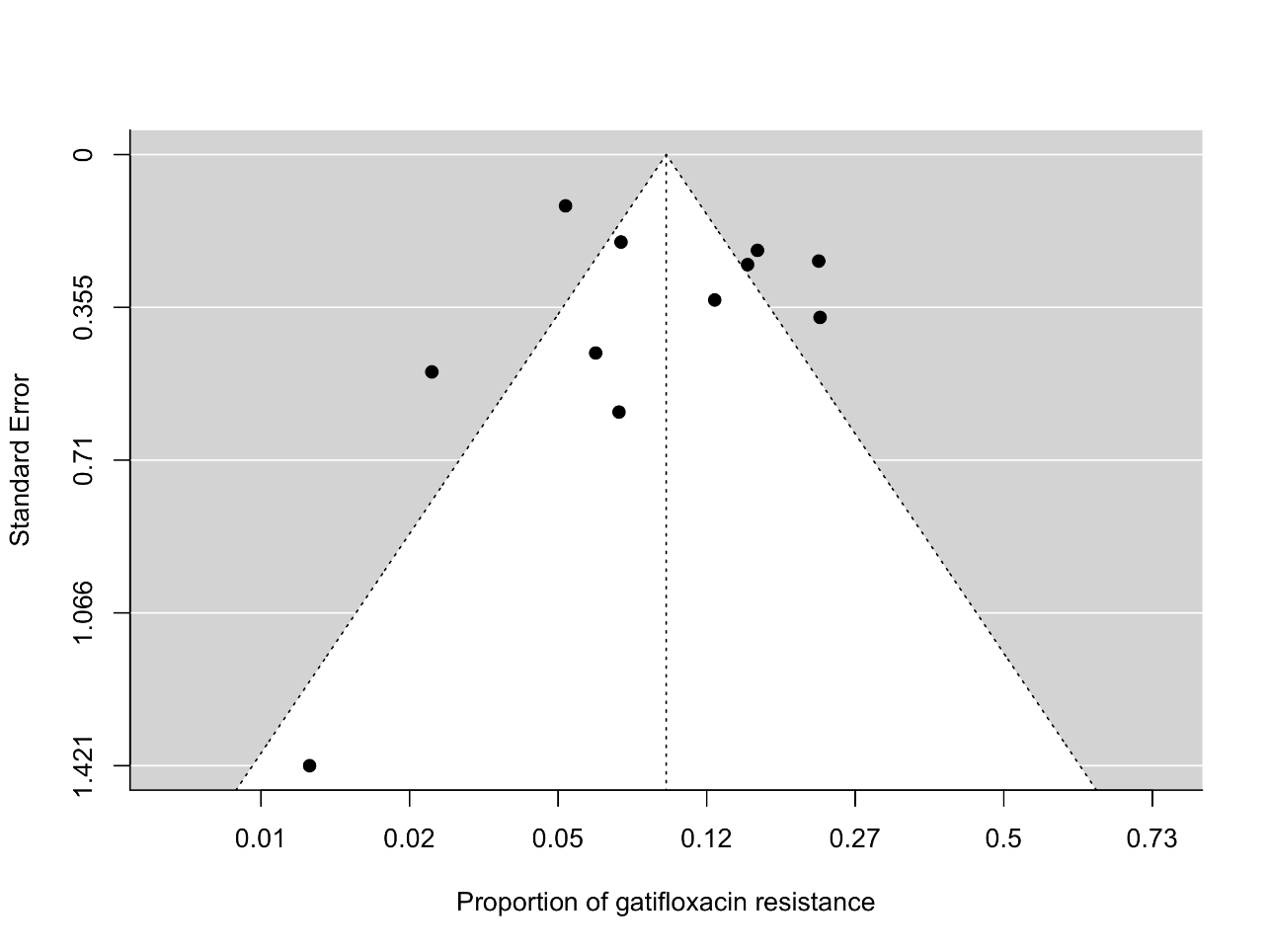
Figure 78. The funnel plot of meta-analysis of publication studies. Each black dot represents a study. The white dots represent missing studies. The black line in the middle represents the average effect size.


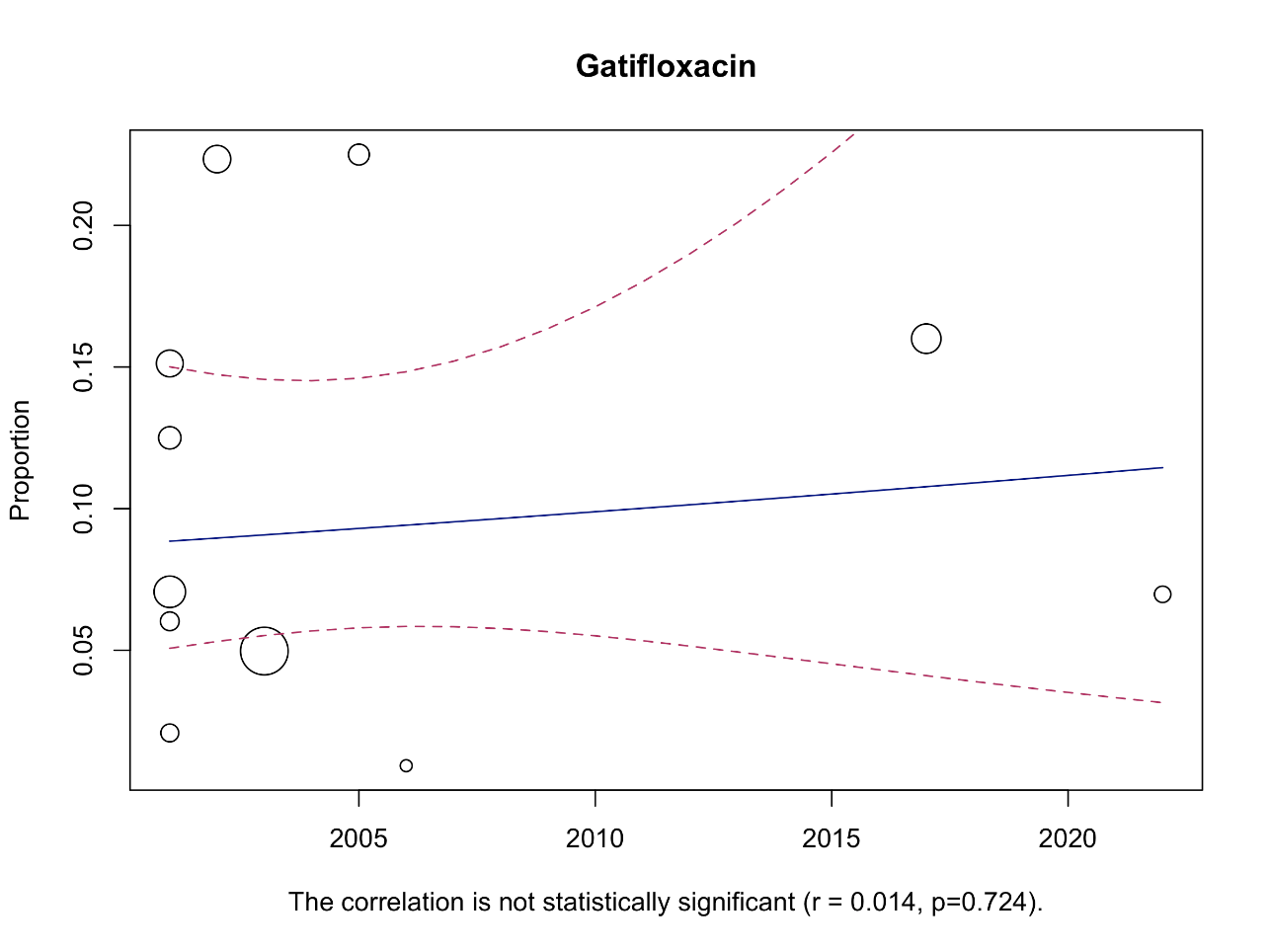


Figure 79. Bubble plot with fitted meta-regression for the year of publication


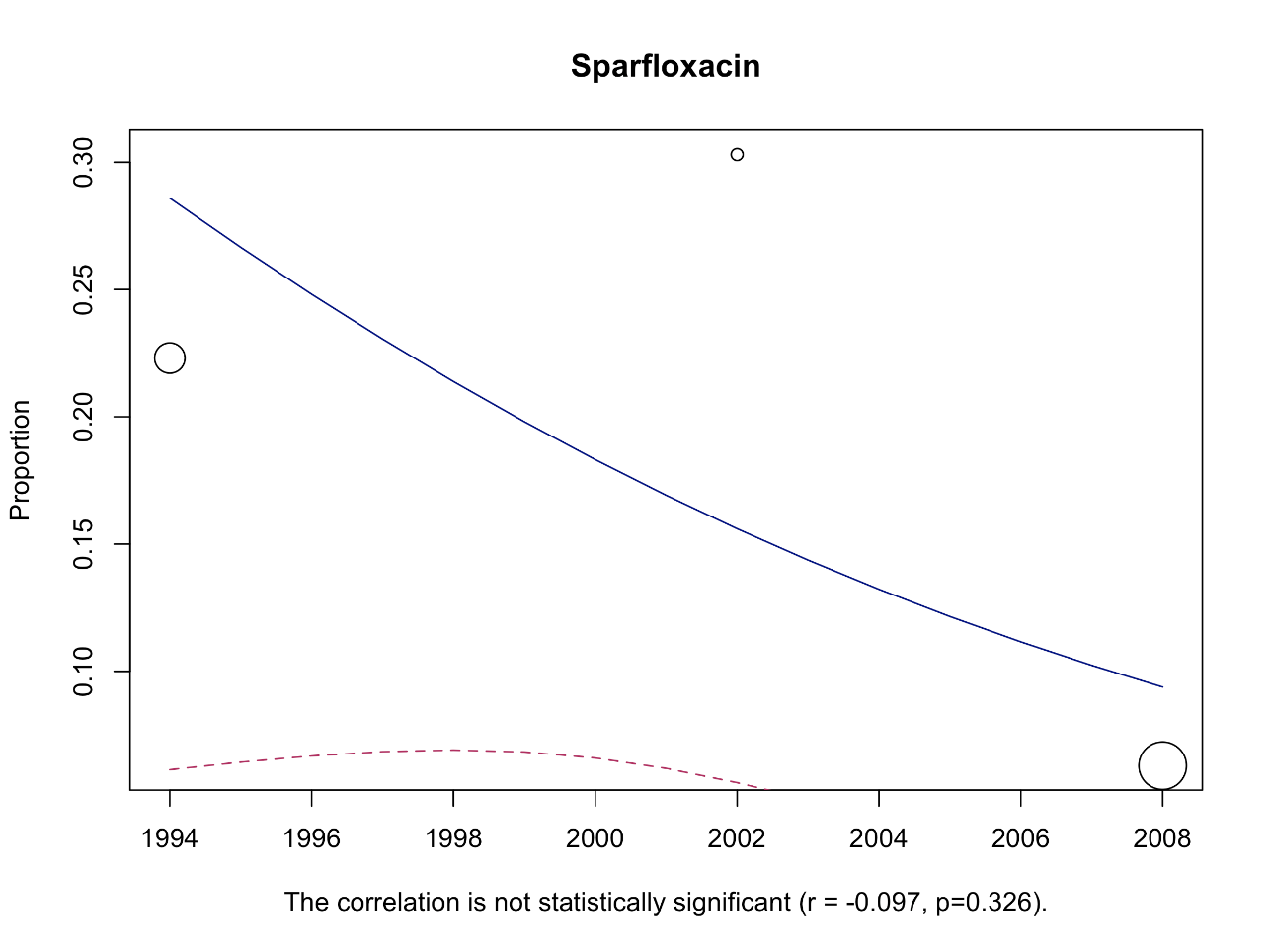

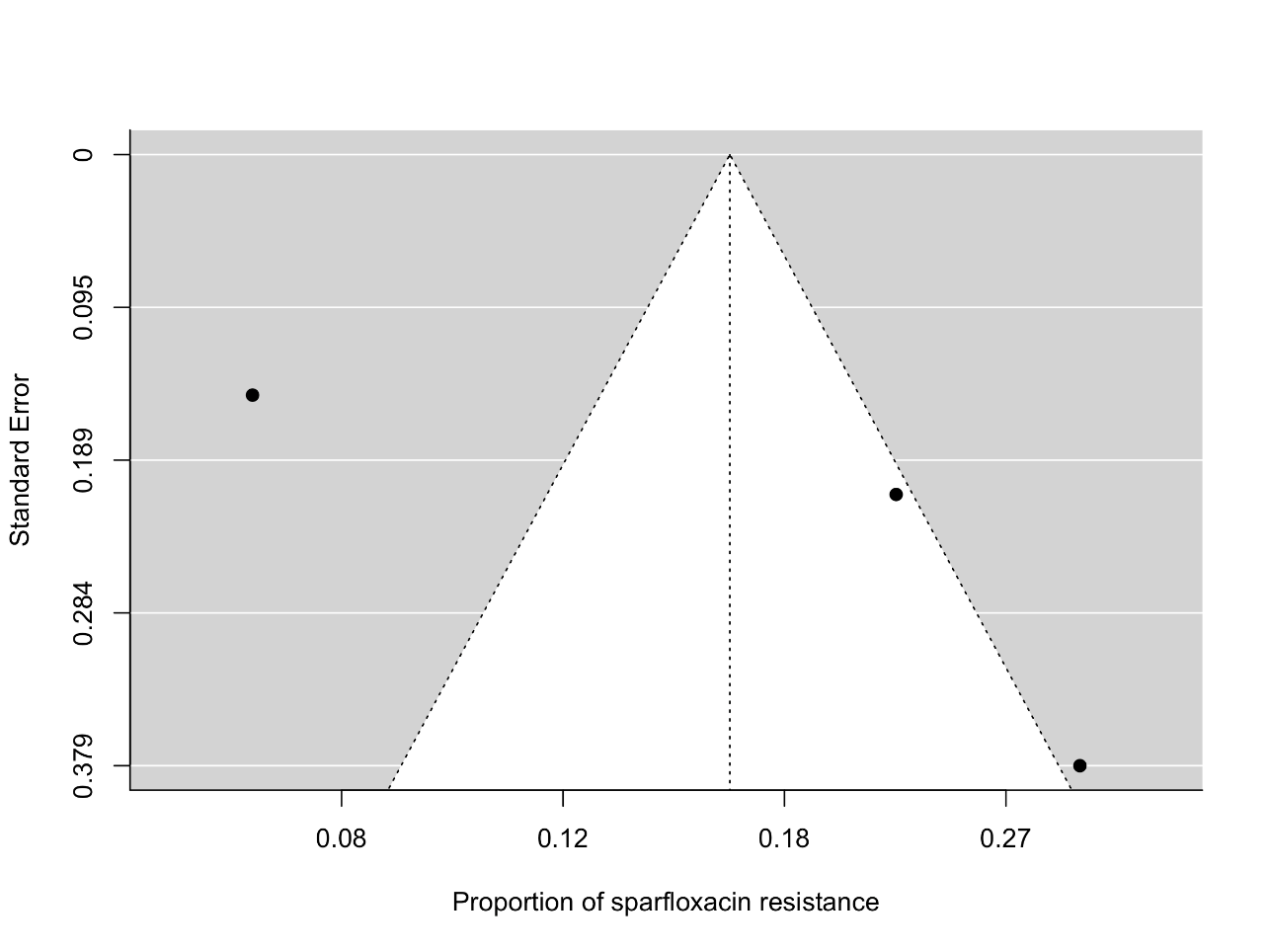
Figure 80. The funnel plot of meta-analysis of publication studies. Each black dot represents a study. The white dots represent missing studies. The black line in the middle represents the average effect size.

Figure 81. Bubble plot with fitted meta-regression for the year of publication


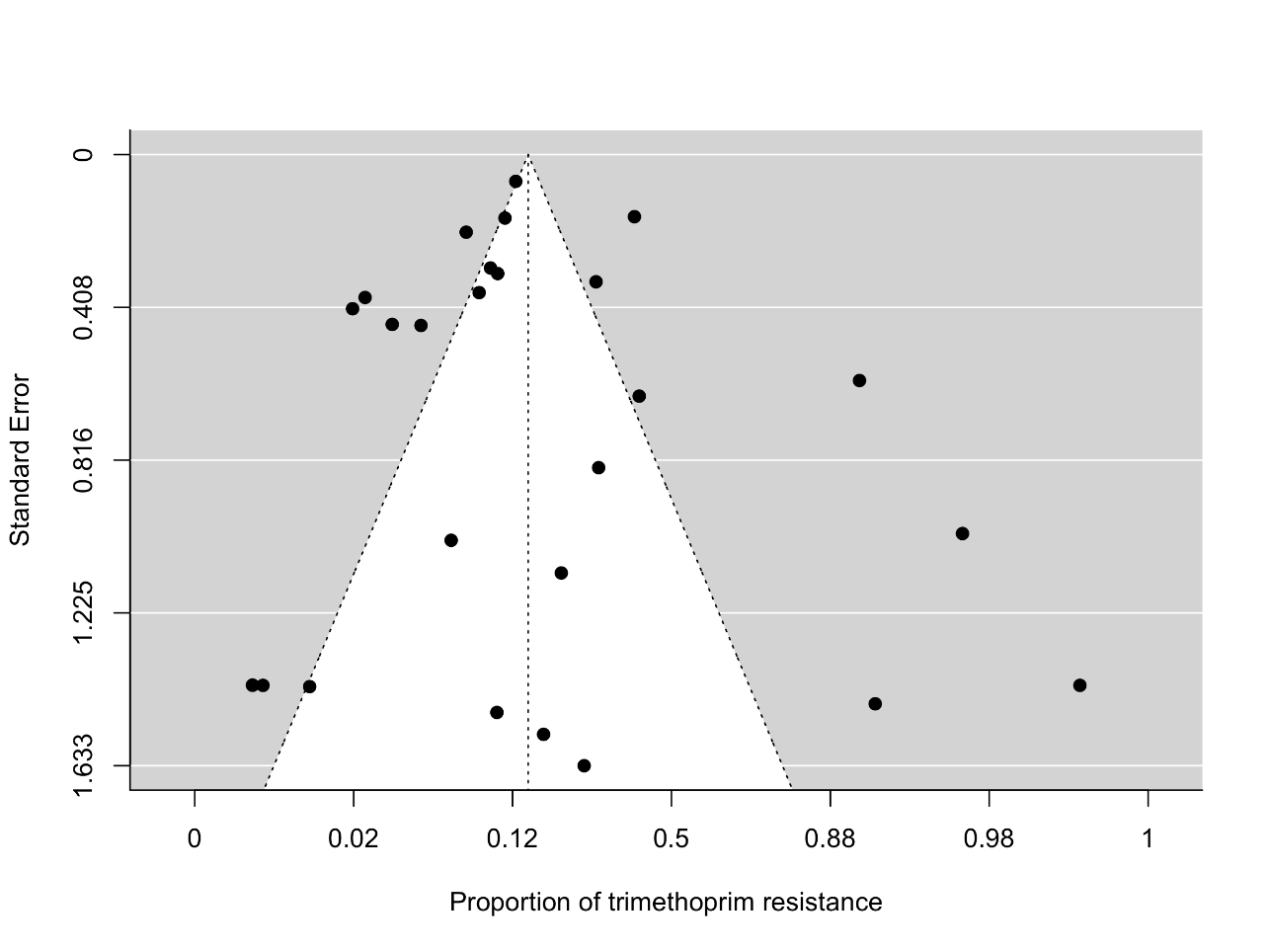


**Sulfonamides**

Figure 82. The funnel plot of meta-analysis of publication studies. Each black dot represents a study. The white dots represent missing studies. The black line in the middle represents the average effect size


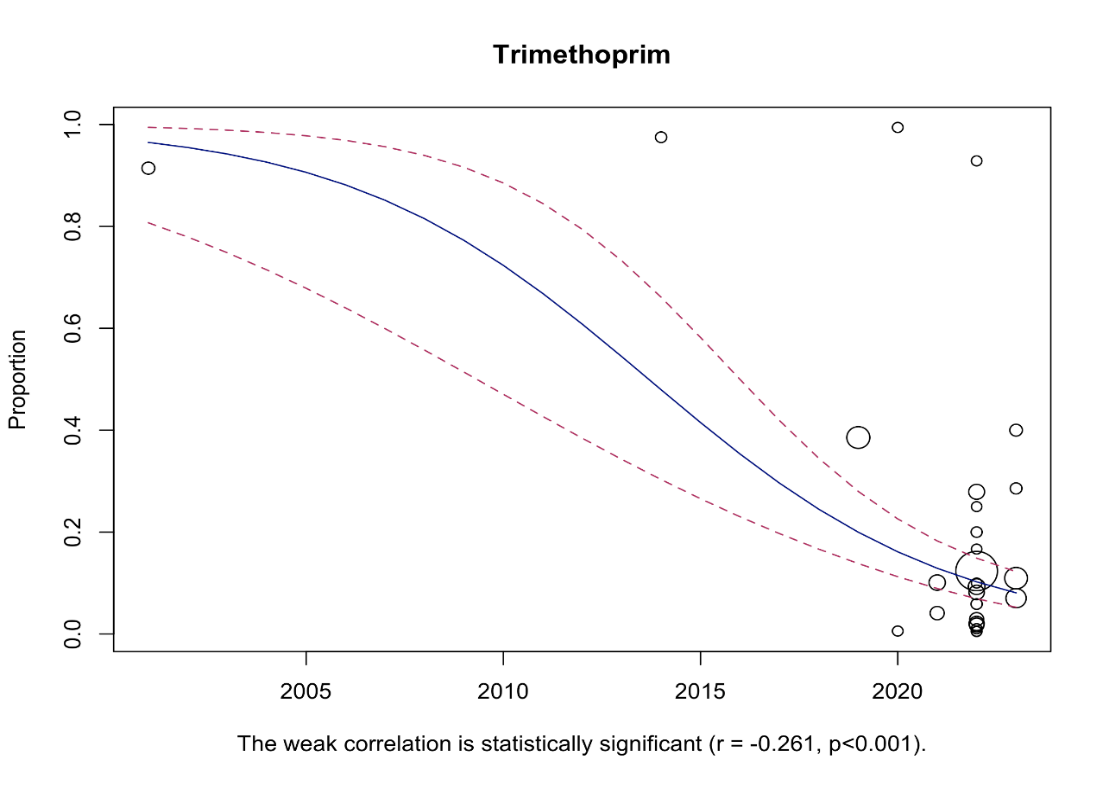


Figure 83. Bubble plot with fitted meta-regression for the year of publication

Figure 84. The funnel plot of meta-analysis of publication studies. Each black dot represents a study. The white dots represent missing studies. The black line in the middle represents the average effect size
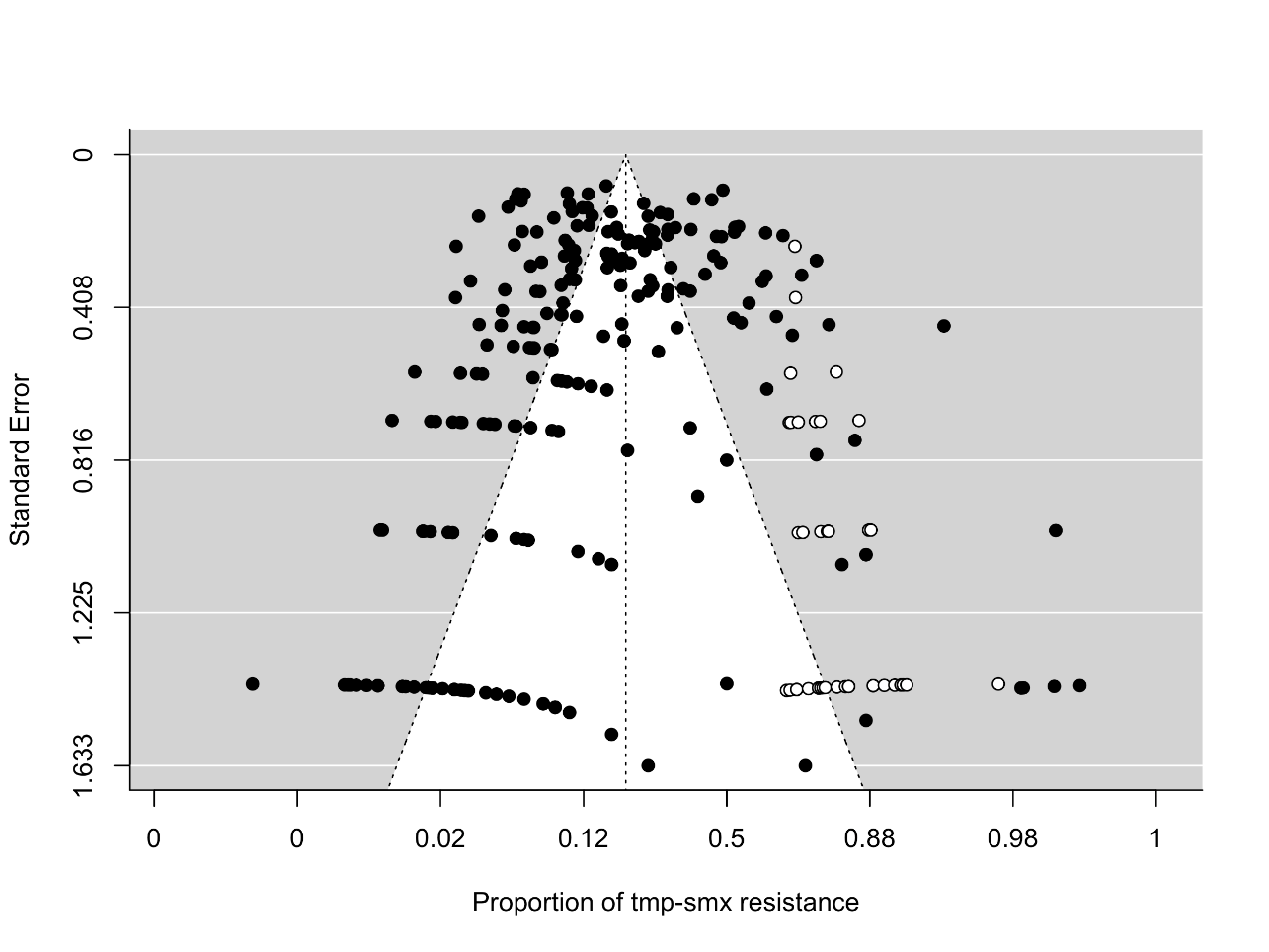


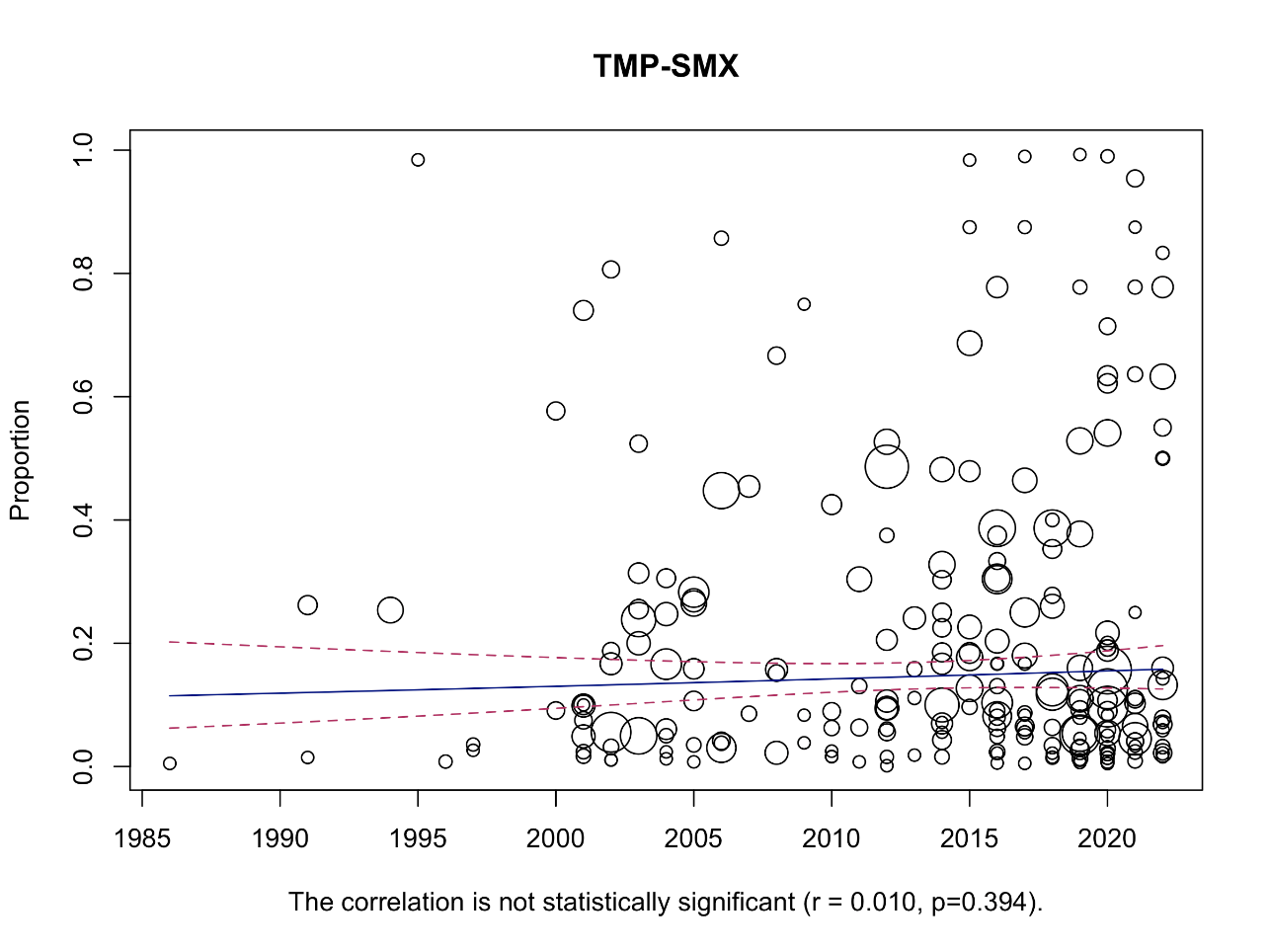


Figure 85. Bubble plot with fitted meta-regression for the year of publication

**Chloramphenicol**

Figure 86. The funnel plot of meta-analysis of publication studies. Each black dot represents a study. The white dots represent missing studies. The black line in the middle represents the average effect size
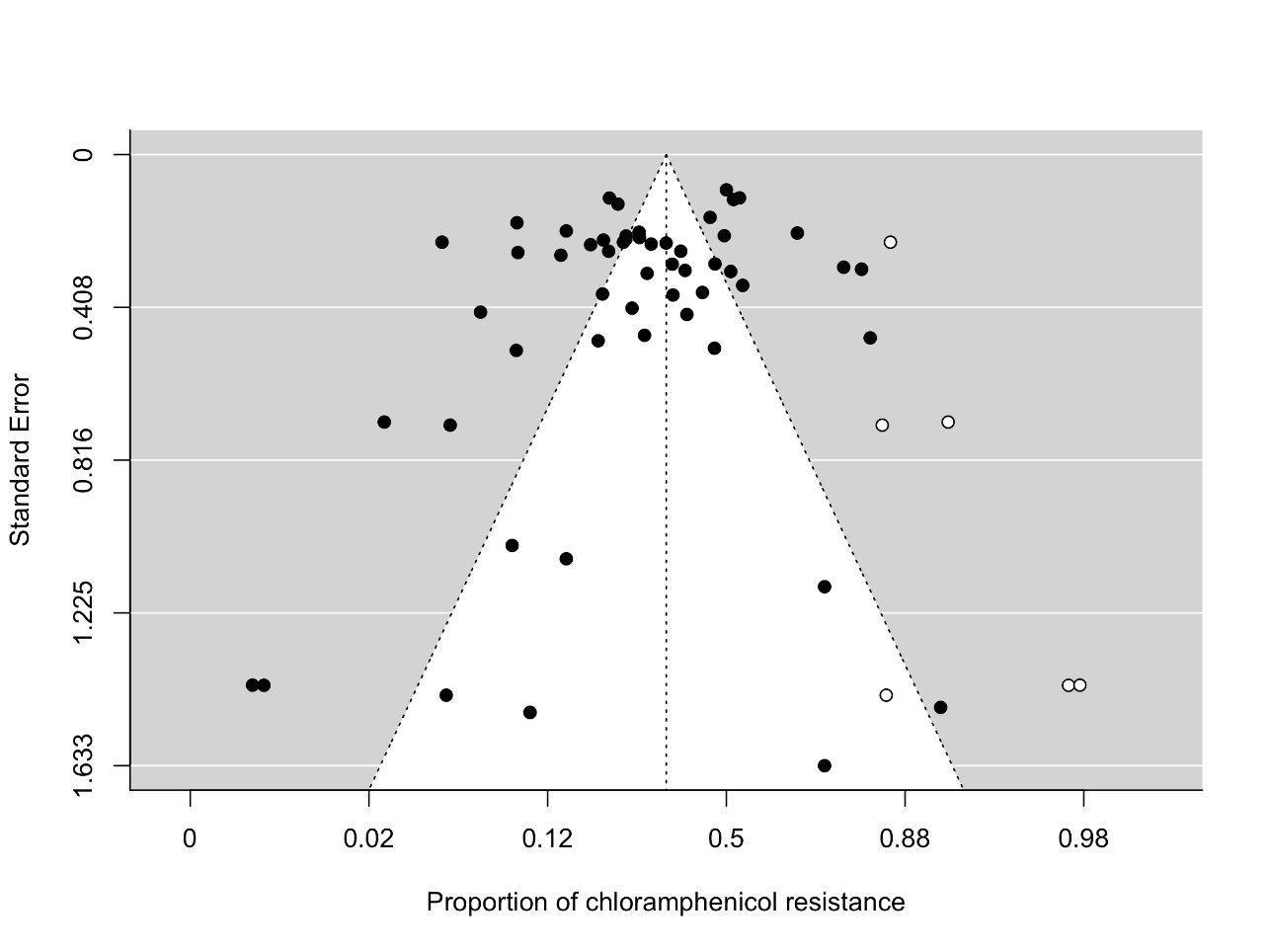


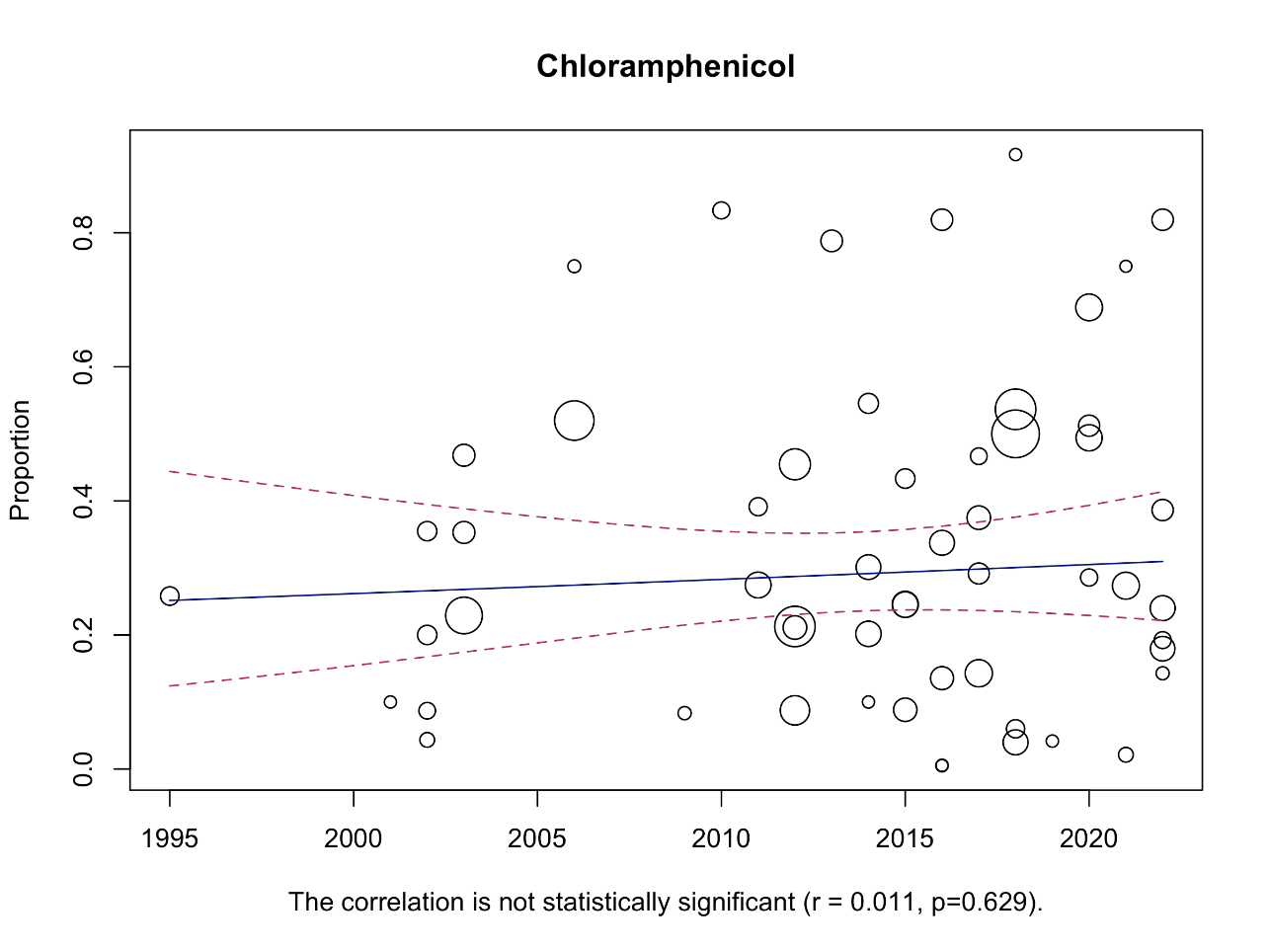


Figure 87. Bubble plot with fitted meta-regression for the year of publication

**Colistin**

Figure 88. The funnel plot of meta-analysis of publication studies. Each black dot represents a study. The white dots represent missing studies. The black line in the middle represents the average effect size
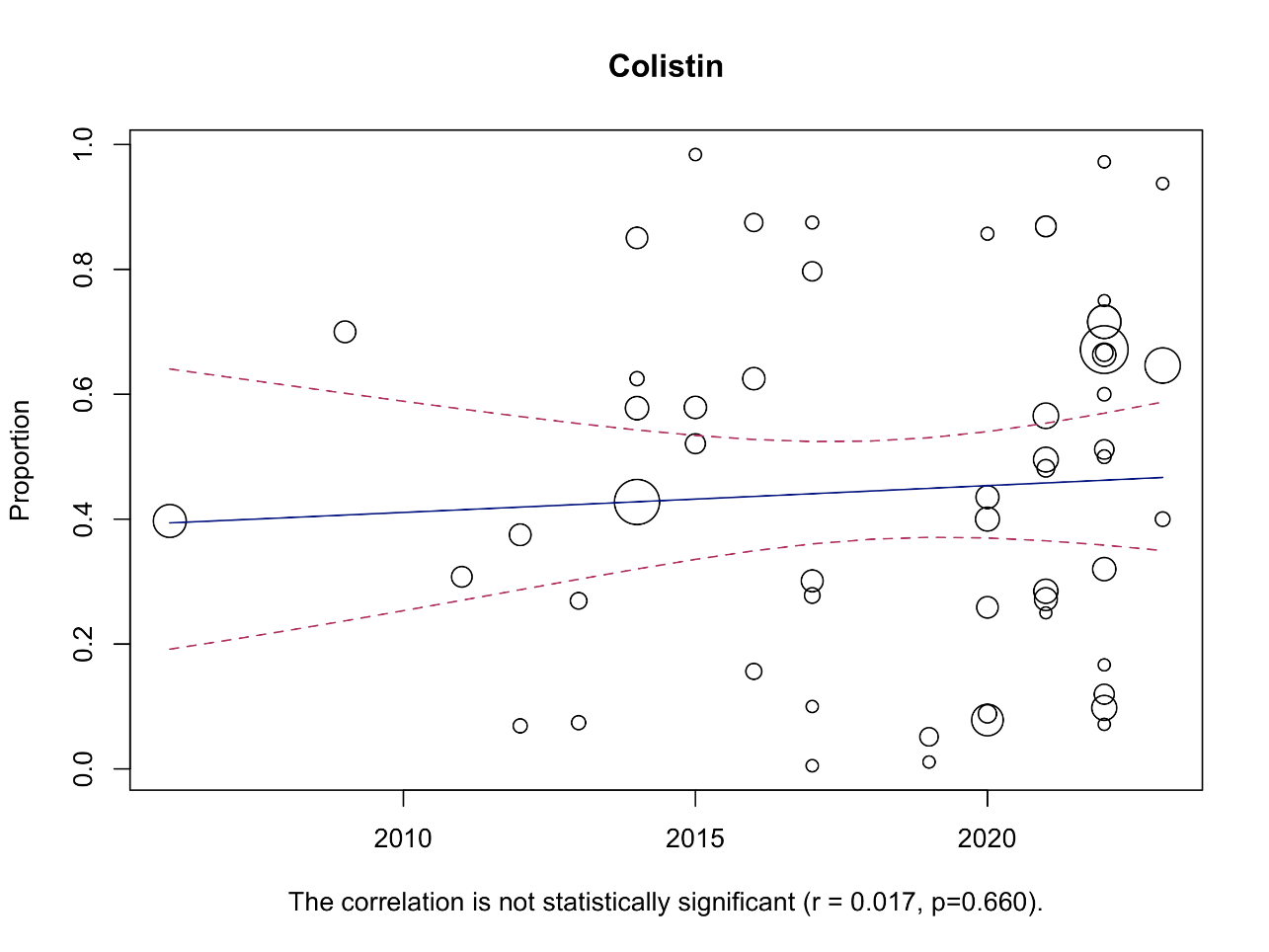

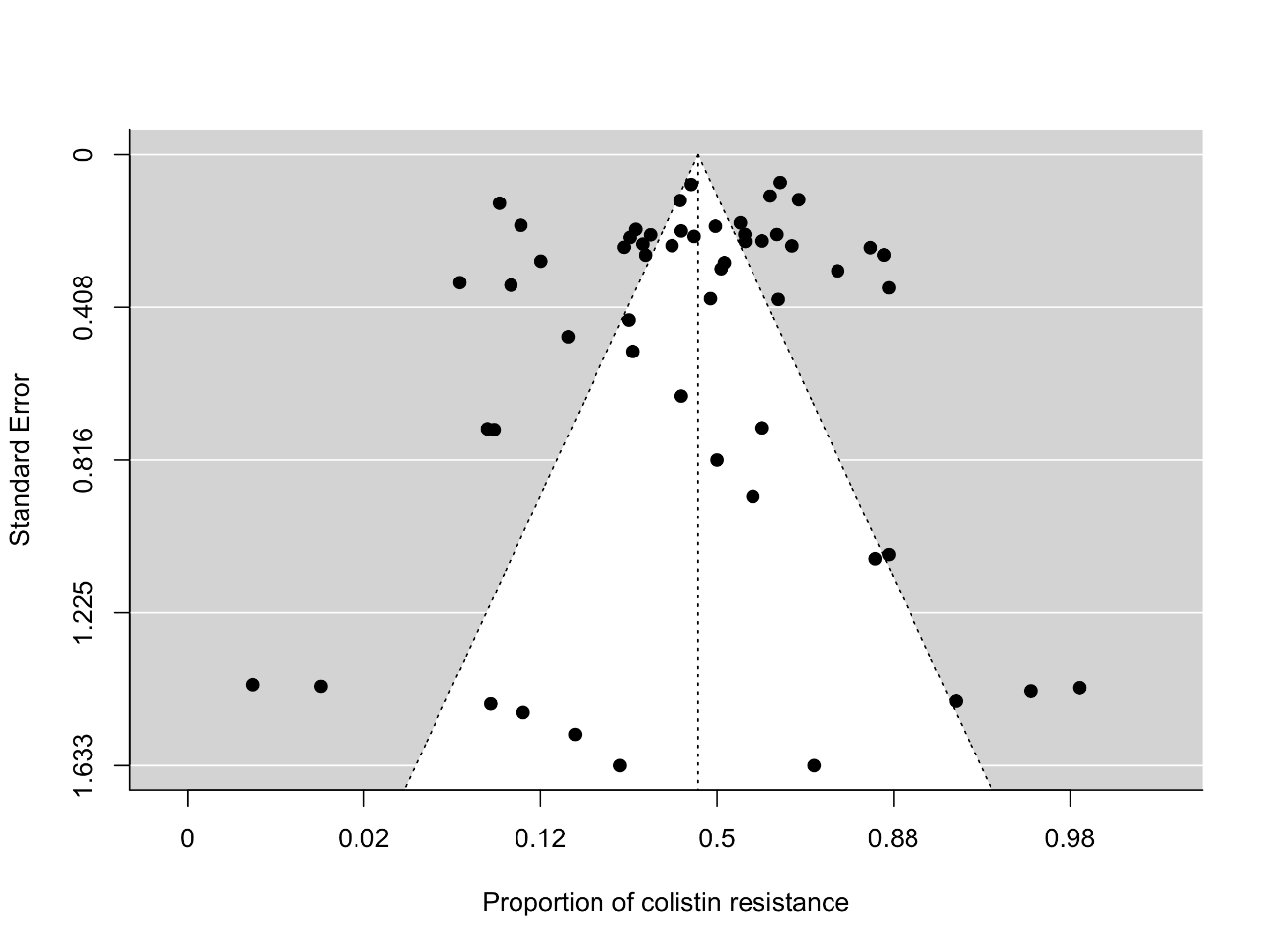


Figure 89. Bubble plot with fitted meta-regression for the year of publication


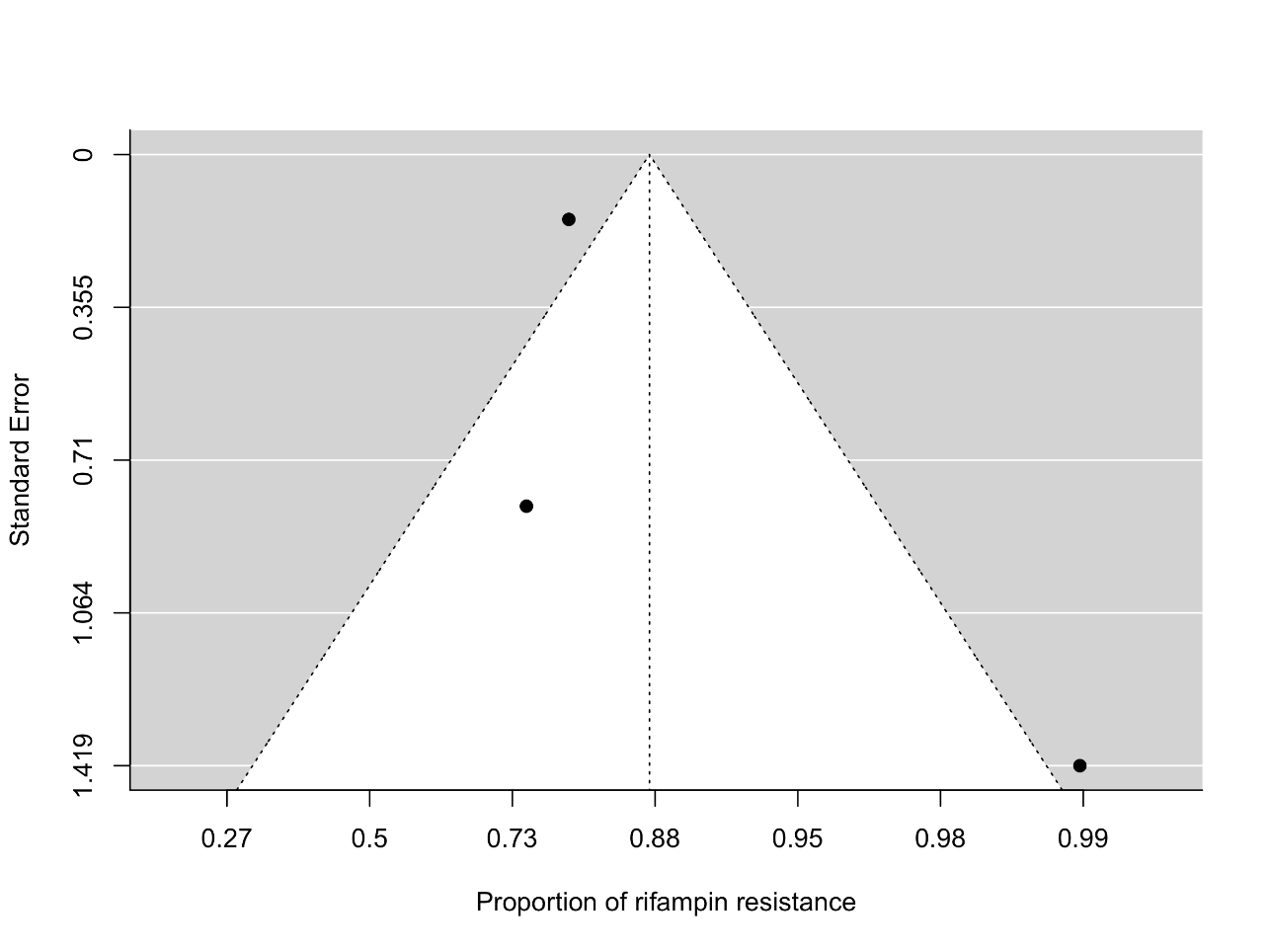


**Rifampin**

Figure 90. The funnel plot of meta-analysis of publication studies. Each black dot represents a study. The black line in the middle represents the average effect size


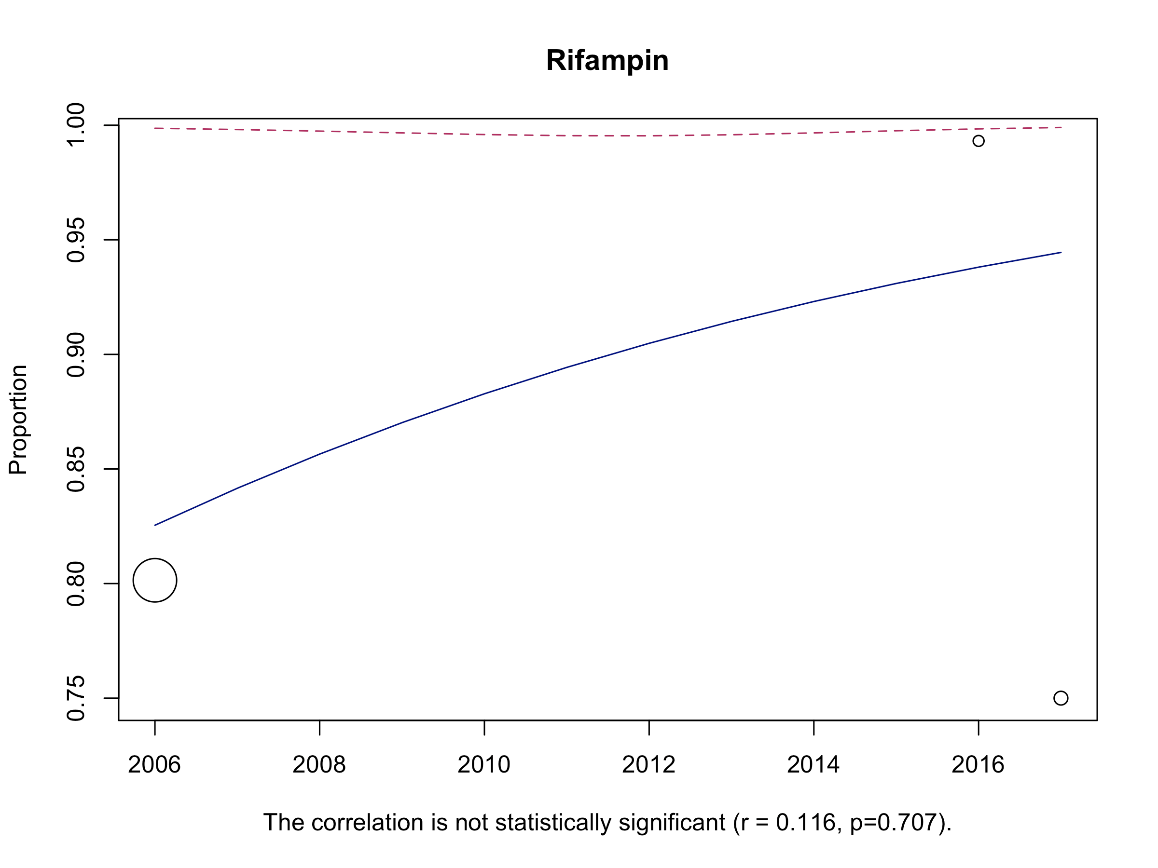


Figure 91. Bubble plot with fitted meta-regression for the year of publication.
