# Supplementary material for: Global mapping of antibiotic resistance rates among clinical isolates of Stenotrophomonas maltophilia: a systematic review and meta-analysis
Source: Ann Clin Microbiol Antimicrob. 2024 Mar 19;23:26. doi: 10.1186/s12941-024-00685-4 (PMC10953290; doi:10.1186/s12941-024-00685-4)
Supplement: Supplementary file 4 — Supplementary Material 4 [file 12941_2024_685_MOESM4_ESM.docx]

Supplementary Table 2. The resistance data of each antimicrobial agent and the subgroup analyses by continents, country, year of publication, method of susceptibility testing, guideline and quality score.

| **Trimethoprim** | | | | | | | |
| --- | --- | --- | --- | --- | --- | --- | --- |
| Subgroup | Proportion | K (n, N) | Proportion (LCI, HCI) | I^2^ | P1 | P2 | P3 |
| Overall |  | 27 (582, 4558) | 0.141 (0.091, 0.214) | 91.73% | <0.001 | <0.001 | NA |
| Year | 1958-2010 | 1 (32, 35) | 0.914 (0.766, 0.972) | 0.00% | <0.001 | >0.999 | <0.001 |
|  | 2011-2023 | 25 (544, 4517) | 0.110 (0.071, 0.166) | 90.57% | <0.001 | <0.001 |  |
| Guideline | Other | 1 (32, 35) | 0.914 (0.766, 0.972) | 0.00% | <0.001 | >0.999 | 0.001 |
|  | CLSI | 18 (415, 3399) | 0.133 (0.080, 0.212) | 90.84% | <0.001 | <0.001 |  |
|  | EUCAST | 4 (43, 825) | 0.056 (0.026, 0.116) | 76.79% | <0.001 | 0.005 |  |
|  | CLSI-EUCAST | 4 (92, 299) | 0.248 (0.013, 0.890) | 92.06% | 0.497 | <0.001 |  |
| Quality score | Low Quality | 7 (333, 2649) | 0.194 (0.110, 0.321) | 91.97% | <0.001 | <0.001 | 0.595 |
|  | Moderate Quality | 17 (173, 1505) | 0.133 (0.055, 0.290) | 88.01% | <0.001 | <0.001 |  |
|  | High Quality | 3 (76, 404) | 0.127 (0.027, 0.429) | 95.99% | 0.021 | <0.001 |  |
| AST Method | DD | 6 (54, 410) | 0.147 (0.028, 0.503) | 92.90% | 0.052 | <0.001 | 0.527 |
|  | AS | 10 (379, 2362) | 0.199 (0.083, 0.404) | 92.83% | 0.007 | <0.001 |  |
|  | DD, DM | 1 (12, 43) | 0.279 (0.166, 0.430) | 0.00% | 0.005 | >0.999 |  |
|  | DM | 10 (137, 1743) | 0.090 (0.046, 0.169) | 88.35% | <0.001 | <0.001 |  |
| Country | Saudi Arabia | 1 (32, 35) | 0.914 (0.766, 0.972) | 0.00% | <0.001 | >0.999 | <0.001 |
|  | India | 2 (5, 220) | 0.026 (0.011, 0.059) | 0.00% | <0.001 | 0.486 |  |
|  | Taiwan | 3 (97, 213) | 0.415 (0.009, 0.983) | 92.62% | 0.878 | <0.001 |  |
|  | Multi-Country | 4 (77, 1388) | 0.044 (0.020, 0.097) | 90.45% | <0.001 | <0.001 |  |
|  | Italy | 1 (2, 7) | 0.286 (0.072, 0.673) | 0.00% | 0.273 | >0.999 |  |
|  | Korea | 2 (223, 1809) | 0.123 (0.109, 0.139) | 0.00% | <0.001 | 0.873 |  |
|  | Iraq | 1 (6, 6) | 0.929 (0.423, 0.996) | 0.00% | 0.081 | >0.999 |  |
|  | Somalia | 1 (1, 17) | 0.059 (0.008, 0.320) | 0.00% | 0.007 | >0.999 |  |
|  | Iran | 1 (0, 1) | 0.250 (0.013, 0.891) | 0.00% | 0.501 | >0.999 |  |
|  | Poland | 2 (0, 194) | 0.005 (0.001, 0.035) | 0.00% | <0.001 | >0.999 |  |
|  | China | 1 (8, 98) | 0.082 (0.041, 0.155) | 0.00% | <0.001 | >0.999 |  |
|  | Peru | 1 (4, 10) | 0.400 (0.158, 0.703) | 0.00% | 0.530 | >0.999 |  |
|  | United Kingdom | 1 (39, 40) | 0.975 (0.843, 0.996) | 0.00% | <0.001 | >0.999 |  |
|  | Turkey | 4 (76, 406) | 0.132 (0.033, 0.404) | 94.00% | 0.014 | <0.001 |  |
|  | Palestine | 1 (1, 5) | 0.200 (0.027, 0.691) | 0.00% | 0.215 | >0.999 |  |
|  | United States | 1 (11, 109) | 0.101 (0.057, 0.173) | 0.00% | <0.001 | >0.999 |  |
| Continent | Asia | 12 (372, 2387) | 0.233 (0.102, 0.450) | 90.53% | 0.018 | <0.001 | 0.243 |
|  | Multi Continent | 4 (77, 1388) | 0.044 (0.020, 0.097) | 90.45% | <0.001 | <0.001 |  |
|  | Europe | 8 (117, 647) | 0.149 (0.045, 0.397) | 92.18% | 0.010 | <0.001 |  |
|  | Africa | 1 (1, 17) | 0.059 (0.008, 0.320) | 0.00% | 0.007 | >0.999 |  |
|  | South America | 1 (4, 10) | 0.400 (0.158, 0.703) | 0.00% | 0.530 | >0.999 |  |
|  | North America | 1 (11, 109) | 0.101 (0.057, 0.173) | 0.00% | <0.001 | >0.999 |  |
| **TMP-SMX** | | | | | | | |
| Subgroup | Proportion | K (n, N) | Proportion (LCI, HCI) | I^2^ | P1 | P2 | P3 |
| Overall |  | 225 (4423, 33188) | 0.147 (0.127, 0.169) | 94.34% | <0.001 | <0.001 | NA |
| year | 2011-2023 | 108 (2241, 17743) | 0.146 (0.118, 0.180) | 94.23% | <0.001 | <0.001 | 0.872 |
|  | 1958-2010 | 99 (1819, 14185) | 0.144 (0.117, 0.175) | 93.72% | <0.001 | <0.001 |  |
| Guideline | CLSI | 156 (2977, 20985) | 0.162 (0.134, 0.193) | 94.51% | <0.001 | <0.001 | 0.040 |
|  | Other | 45 (904, 7307) | 0.124 (0.090, 0.169) | 93.97% | <0.001 | <0.001 |  |
|  | EUCAST | 21 (531, 4490) | 0.128 (0.085, 0.187) | 92.79% | <0.001 | <0.001 |  |
|  | CLSI-EUCAST | 3 (11, 406) | 0.032 (0.012, 0.081) | 59.79% | <0.001 | 0.083 |  |
| Quality score | Low Quality | 17 (287, 2902) | 0.155 (0.093, 0.247) | 92.07% | <0.001 | <0.001 | 0.090 |
|  | Moderate Quality | 150 (3046, 21811) | 0.162 (0.135, 0.193) | 94.40% | <0.001 | <0.001 |  |
|  | High Quality | 58 (1090, 8475) | 0.110 (0.081, 0.148) | 94.53% | <0.001 | <0.001 |  |
| AST Method | DM | 117 (3026, 21573) | 0.156 (0.128, 0.190) | 95.63% | <0.001 | <0.001 | 0.278 |
|  | DD | 65 (774, 5667) | 0.165 (0.126, 0.215) | 91.23% | <0.001 | <0.001 |  |
|  | AS | 26 (399, 3972) | 0.095 (0.050, 0.172) | 94.40% | <0.001 | <0.001 |  |
|  | DD, AS | 5 (66, 632) | 0.105 (0.078, 0.141) | 30.12% | <0.001 | 0.221 |  |
|  | DD, DM | 8 (107, 823) | 0.139 (0.088, 0.211) | 69.66% | <0.001 | 0.002 |  |
|  | AS, DD, DM | 1 (43, 325) | 0.132 (0.100, 0.174) | 0.00% | <0.001 | >0.999 |  |
|  | DM, AS | 1 (0, 76) | 0.006 (0.000, 0.095) | 0.00% | <0.001 | >0.999 |  |
|  | DD, DM, AS | 1 (5, 18) | 0.278 (0.121, 0.519) | 0.00% | 0.069 | >0.999 |  |
| Country | Saudi Arabia | 5 (91, 378) | 0.355 (0.111, 0.707) | 90.69% | 0.429 | <0.001 | 0.003 |
|  | France | 6 (71, 689) | 0.090 (0.052, 0.152) | 76.06% | <0.001 | <0.001 |  |
|  | Taiwan | 21 (338, 1613) | 0.238 (0.167, 0.328) | 84.74% | <0.001 | <0.001 |  |
|  | Korea | 10 (113, 816) | 0.124 (0.069, 0.213) | 85.25% | <0.001 | <0.001 |  |
|  | Italy | 8 (271, 802) | 0.423 (0.245, 0.623) | 94.17% | 0.452 | <0.001 |  |
|  | Spain | 3 (39, 140) | 0.279 (0.211, 0.359) | 0.00% | <0.001 | 0.780 |  |
|  | Tunis | 2 (0, 20) | 0.046 (0.006, 0.265) | 0.00% | 0.003 | 0.851 |  |
|  | Sweden | 3 (24, 247) | 0.130 (0.017, 0.572) | 93.48% | 0.089 | <0.001 |  |
|  | Japan | 4 (45, 315) | 0.147 (0.079, 0.255) | 57.89% | <0.001 | 0.068 |  |
|  | United States | 28 (609, 5633) | 0.122 (0.076, 0.191) | 94.65% | <0.001 | <0.001 |  |
|  | China | 26 (1099, 6515) | 0.205 (0.132, 0.304) | 97.61% | <0.001 | <0.001 |  |
|  | India | 10 (129, 516) | 0.151 (0.063, 0.320) | 91.14% | <0.001 | <0.001 |  |
|  | Egypt | 3 (39, 152) | 0.332 (0.142, 0.598) | 86.42% | 0.212 | <0.001 |  |
|  | Iraq | 1 (5, 6) | 0.833 (0.369, 0.977) | 0.00% | 0.142 | >0.999 |  |
|  | Argentina | 3 (75, 715) | 0.179 (0.047, 0.492) | 89.93% | 0.046 | <0.001 |  |
|  | Iran | 9 (155, 794) | 0.133 (0.053, 0.298) | 94.29% | <0.001 | <0.001 |  |
|  | Multi-Country | 18 (657, 7915) | 0.087 (0.055, 0.136) | 96.58% | <0.001 | <0.001 |  |
|  | Mexico | 2 (54, 140) | 0.510 (0.174, 0.837) | 89.84% | 0.962 | 0.002 |  |
|  | Germany | 9 (76, 746) | 0.116 (0.056, 0.224) | 86.47% | <0.001 | <0.001 |  |
|  | Malaysia | 3 (41, 157) | 0.120 (0.010, 0.640) | 88.69% | 0.129 | <0.001 |  |
|  | Turkey | 12 (135, 1157) | 0.100 (0.058, 0.169) | 86.02% | <0.001 | <0.001 |  |
|  | Serbia | 1 (0, 88) | 0.006 (0.000, 0.083) | 0.00% | <0.001 | >0.999 |  |
|  | Brazil | 4 (23, 179) | 0.039 (0.002, 0.462) | 88.24% | 0.039 | <0.001 |  |
|  | Israel | 2 (1, 37) | 0.042 (0.009, 0.186) | 0.00% | <0.001 | 0.638 |  |
|  | Somalia | 1 (1, 17) | 0.059 (0.008, 0.320) | 0.00% | 0.007 | >0.999 |  |
|  | Tunisia | 1 (10, 33) | 0.303 (0.171, 0.477) | 0.00% | 0.028 | >0.999 |  |
|  | Hungary | 4 (163, 1149) | 0.147 (0.063, 0.307) | 89.69% | <0.001 | <0.001 |  |
|  | Ireland | 1 (12, 34) | 0.353 (0.213, 0.524) | 0.00% | 0.091 | >0.999 |  |
|  | Poland | 2 (0, 102) | 0.019 (0.001, 0.215) | 42.78% | 0.003 | 0.186 |  |
|  | United Kingdom | 2 (10, 44) | 0.239 (0.135, 0.386) | 0.00% | 0.001 | 0.474 |  |
|  | Switzerland | 1 (0, 33) | 0.015 (0.001, 0.196) | 0.00% | 0.003 | >0.999 |  |
|  | European country | 2 (22, 219) | 0.101 (0.067, 0.148) | 0.00% | <0.001 | 0.844 |  |
|  | Thailand | 3 (21, 281) | 0.076 (0.050, 0.113) | 0.00% | <0.001 | 0.763 |  |
|  | Canada | 5 (64, 669) | 0.111 (0.030, 0.336) | 91.41% | 0.004 | <0.001 |  |
|  | Nepal | 1 (1, 7) | 0.143 (0.020, 0.581) | 0.00% | 0.097 | >0.999 |  |
|  | Malawi | 1 (0, 1) | 0.250 (0.013, 0.891) | 0.00% | 0.501 | >0.999 |  |
|  | Qatar | 1 (7, 317) | 0.022 (0.011, 0.046) | 0.00% | <0.001 | >0.999 |  |
|  | Greece | 1 (2, 62) | 0.032 (0.008, 0.120) | 0.00% | <0.001 | >0.999 |  |
|  | South Korea | 1 (4, 118) | 0.034 (0.013, 0.087) | 0.00% | <0.001 | >0.999 |  |
|  | Ethiopia | 1 (2, 5) | 0.400 (0.100, 0.800) | 0.00% | 0.657 | >0.999 |  |
|  | Denmark | 1 (1, 124) | 0.008 (0.001, 0.055) | 0.00% | <0.001 | >0.999 |  |
|  | England | 1 (10, 100) | 0.100 (0.055, 0.176) | 0.00% | <0.001 | >0.999 |  |
|  | Andalusia | 1 (1, 20) | 0.050 (0.007, 0.282) | 0.00% | 0.004 | >0.999 |  |
|  | Latin America | 1 (2, 83) | 0.024 (0.006, 0.091) | 0.00% | <0.001 | >0.999 |  |
| Continent | Asia | 98 (2056, 11950) | 0.173 (0.139, 0.214) | 94.40% | <0.001 | <0.001 | 0.091 |
|  | Europe | 58 (837, 5756) | 0.138 (0.107, 0.177) | 90.26% | <0.001 | <0.001 |  |
|  | Africa | 9 (52, 228) | 0.253 (0.148, 0.398) | 64.06% | 0.002 | 0.004 |  |
|  | North America | 35 (727, 6442) | 0.134 (0.088, 0.200) | 94.69% | <0.001 | <0.001 |  |
|  | South America | 8 (100, 977) | 0.090 (0.030, 0.236) | 91.10% | <0.001 | <0.001 |  |
|  | Multi Continent | 17 (651, 7835) | 0.088 (0.055, 0.139) | 96.78% | <0.001 | <0.001 |  |
| **Ciprofloxacin** | | | | | | | |
| Subgroup | Proportion | K (n, N) | Proportion (LCI, HCI) | I^2^ | P1 | P2 | P3 |
| Overall |  | 149 (5814, 12373) | 0.460 (0.415, 0.506) | 94.03% | 0.089 | <0.001 | NA |
| Year | 2011-2023 | 46 (1253, 3435) | 0.466 (0.373, 0.562) | 93.92% | 0.492 | <0.001 | 0.908  0.908 |
|  | 1958-2010 | 90 (4291, 8434) | 0.458 (0.405, 0.512) | 94.06% | 0.127 | <0.001 |  |
| Guideline | CLSI | 83 (2368, 5820) | 0.468 (0.406, 0.531) | 92.53% | 0.318 | <0.001 | 0.889 |
|  | Other | 62 (3282, 6109) | 0.454 (0.388, 0.522) | 94.80% | 0.189 | <0.001 |  |
|  | EUCAST | 4 (164, 444) | 0.405 (0.178, 0.681) | 94.84% | 0.510 | <0.001 |  |
| Quality score | Low Quality | 15 (325, 550) | 0.548 (0.375, 0.710) | 89.36% | 0.595 | <0.001 | 0.294 |
|  | Moderate Quality | 88 (3678, 7799) | 0.471 (0.410, 0.532) | 94.50% | 0.348 | <0.001 |  |
|  | High Quality | 46 (1811, 4024) | 0.416 (0.343, 0.494) | 93.94% | 0.034 | <0.001 |  |
| AST Method | DM | 87 (4165, 8228) | 0.488 (0.432, 0.545) | 94.45% | 0.686 | <0.001 | <0.001 |
|  | DD | 48 (881, 2733) | 0.366 (0.299, 0.438) | 88.19% | <0.001 | <0.001 |  |
|  | AS | 9 (632, 860) | 0.760 (0.549, 0.892) | 94.40% | 0.018 | <0.001 |  |
|  | DD, DM | 1 (27, 150) | 0.180 (0.126, 0.250) | 0.00% | <0.001 | >0.999 |  |
|  | DD, AS | 3 (108, 390) | 0.368 (0.169, 0.625) | 92.35% | 0.314 | <0.001 |  |
|  | DD, DM, AS | 1 (1, 12) | 0.083 (0.012, 0.413) | 0.00% | 0.022 | >0.999 |  |
| Country | Saudi Arabia | 7 (248, 450) | 0.415 (0.229, 0.628) | 92.66% | 0.436 | <0.001 | <0.001 |
|  | France | 5 (239, 550) | 0.539 (0.203, 0.844) | 97.23% | 0.839 | <0.001 |  |
|  | Canada | 5 (163, 295) | 0.553 (0.495, 0.609) | 0.00% | 0.072 | 0.666 |  |
|  | India | 6 (9, 122) | 0.091 (0.050, 0.157) | 0.00% | <0.001 | 0.725 |  |
|  | Italy | 15 (898, 1394) | 0.602 (0.459, 0.729) | 94.78% | 0.162 | <0.001 |  |
|  | United States | 20 (1762, 2837) | 0.597 (0.499, 0.688) | 93.41% | 0.053 | <0.001 |  |
|  | Spain | 8 (119, 299) | 0.437 (0.313, 0.570) | 75.97% | 0.352 | <0.001 |  |
|  | Serbia | 3 (35, 126) | 0.515 (0.017, 0.985) | 91.79% | 0.977 | <0.001 |  |
|  | Tunis | 2 (2, 20) | 0.102 (0.026, 0.329) | 0.00% | 0.004 | 0.762 |  |
|  | Sweden | 3 (78, 247) | 0.310 (0.213, 0.427) | 42.15% | 0.002 | 0.178 |  |
|  | Taiwan | 16 (295, 910) | 0.297 (0.231, 0.373) | 76.63% | <0.001 | <0.001 |  |
|  | Korea | 4 (58, 88) | 0.709 (0.181, 0.964) | 84.08% | 0.468 | <0.001 |  |
|  | Ireland | 1 (3, 10) | 0.300 (0.100, 0.624) | 0.00% | 0.220 | >0.999 |  |
|  | Argentina | 2 (32, 95) | 0.375 (0.121, 0.723) | 90.33% | 0.496 | 0.001 |  |
|  | Iran | 7 (170, 1069) | 0.221 (0.136, 0.338) | 87.34% | <0.001 | <0.001 |  |
|  | Mexico | 1 (26, 119) | 0.218 (0.153, 0.302) | 0.00% | <0.001 | >0.999 |  |
|  | Multi-Country | 5 (388, 989) | 0.483 (0.354, 0.614) | 86.79% | 0.805 | <0.001 |  |
|  | Turkey | 5 (221, 314) | 0.457 (0.125, 0.833) | 96.02% | 0.850 | <0.001 |  |
|  | China | 8 (193, 696) | 0.400 (0.259, 0.560) | 90.32% | 0.220 | <0.001 |  |
|  | Japan | 1 (56, 66) | 0.848 (0.741, 0.916) | 0.00% | <0.001 | >0.999 |  |
|  | Tunisia | 1 (5, 33) | 0.152 (0.065, 0.316) | 0.00% | <0.001 | >0.999 |  |
|  | Australia | 1 (18, 45) | 0.400 (0.269, 0.548) | 0.00% | 0.183 | >0.999 |  |
|  | Belgium | 2 (89, 121) | 0.734 (0.648, 0.805) | 0.00% | <0.001 | 0.333 |  |
|  | Switzerland | 1 (27, 33) | 0.818 (0.650, 0.916) | 0.00% | <0.001 | >0.999 |  |
|  | European country | 2 (56, 219) | 0.374 (0.101, 0.761) | 93.67% | 0.546 | <0.001 |  |
|  | Malaysia | 1 (44, 84) | 0.524 (0.418, 0.628) | 0.00% | 0.663 | >0.999 |  |
|  | Hungary | 2 (104, 157) | 0.723 (0.472, 0.884) | 77.85% | 0.079 | 0.034 |  |
|  | Nepal | 1 (1, 7) | 0.143 (0.020, 0.581) | 0.00% | 0.097 | >0.999 |  |
|  | Malawi | 1 (1, 1) | 0.750 (0.109, 0.987) | 0.00% | 0.501 | >0.999 |  |
|  | Egypt | 1 (17, 32) | 0.531 (0.361, 0.694) | 0.00% | 0.724 | >0.999 |  |
|  | Qatar | 1 (177, 317) | 0.558 (0.503, 0.612) | 0.00% | 0.038 | >0.999 |  |
|  | Thailand | 1 (18, 64) | 0.281 (0.185, 0.403) | 0.00% | <0.001 | >0.999 |  |
|  | Germany | 3 (103, 121) | 0.844 (0.739, 0.911) | 30.03% | <0.001 | 0.239 |  |
|  | Ethiopia | 1 (0, 5) | 0.083 (0.005, 0.622) | 0.00% | 0.105 | >0.999 |  |
|  | Denmark | 1 (27, 124) | 0.218 (0.154, 0.299) | 0.00% | <0.001 | >0.999 |  |
|  | England | 1 (71, 100) | 0.710 (0.614, 0.790) | 0.00% | <0.001 | >0.999 |  |
|  | Latin America | 1 (36, 83) | 0.434 (0.332, 0.542) | 0.00% | 0.229 | >0.999 |  |
|  | Brazil | 3 (25, 131) | 0.175 (0.073, 0.363) | 75.77% | 0.002 | 0.016 |  |
| Continent | Asia | 54 (1308, 3953) | 0.351 (0.285, 0.423) | 92.25% | <0.001 | <0.001 | <0.001 |
|  | Europe | 52 (2070, 3815) | 0.556 (0.472, 0.638) | 94.28% | 0.192 | <0.001 |  |
|  | North America | 26 (1951, 3251) | 0.566 (0.481, 0.648) | 92.95% | 0.126 | <0.001 |  |
|  | Africa | 6 (25, 91) | 0.233 (0.086, 0.495) | 69.48% | 0.046 | 0.006 |  |
|  | South America | 6 (93, 309) | 0.280 (0.168, 0.429) | 82.90% | 0.005 | <0.001 |  |
|  | Multi Continent | 4 (349, 909) | 0.488 (0.323, 0.656) | 88.77% | 0.891 | <0.001 |  |
|  | Australia | 1 (18, 45) | 0.400 (0.269, 0.548) | 0.00% | 0.183 | >0.999 |  |
| **Ofloxacin** | | | | | | | |
| Subgroup | Proportion | K (n, N) | Proportion (LCI, HCI) | I^2^ | P1 | P2 | P3 |
| Overall |  | 13 (225, 975) | 0.280 (0.185, 0.400) | 89.09% | <0.001 | <0.001 | NA |
| Year | 1958-2010 | 8 (108, 382) | 0.283 (0.146, 0.477) | 88.01% | 0.030 | <0.001 | 0.569 |
|  | 2011-2023 | 4 (108, 573) | 0.226 (0.135, 0.354) | 86.32% | <0.001 | <0.001 |  |
| Guideline | Other | 6 (87, 210) | 0.401 (0.233, 0.596) | 79.29% | 0.319 | <0.001 | 0.182 |
|  | CLSI | 5 (75, 456) | 0.173 (0.082, 0.328) | 88.85% | <0.001 | <0.001 |  |
|  | EUCAST | 2 (63, 309) | 0.328 (0.077, 0.742) | 94.64% | 0.428 | <0.001 |  |
| Quality score | Low Quality | 2 (29, 62) | 0.468 (0.348, 0.591) | 0.00% | 0.612 | 0.847 | 0.213 |
|  | Moderate Quality | 9 (133, 517) | 0.279 (0.155, 0.450) | 88.86% | 0.013 | <0.001 |  |
|  | High Quality | 2 (63, 396) | 0.160 (0.127, 0.200) | 0.00% | <0.001 | 0.343 |  |
| AST Method | DM | 8 (124, 473) | 0.305 (0.161, 0.501) | 90.67% | 0.052 | <0.001 | 0.904 |
|  | DD | 3 (23, 70) | 0.286 (0.094, 0.607) | 79.24% | 0.184 | 0.008 |  |
|  | DD, DM | 1 (30, 150) | 0.200 (0.144, 0.272) | 0.00% | <0.001 | >0.999 |  |
|  | DD, AS | 1 (48, 282) | 0.170 (0.131, 0.219) | 0.00% | <0.001 | >0.999 |  |
| Country | Italy | 2 (16, 51) | 0.326 (0.150, 0.571) | 63.67% | 0.160 | 0.097 | 0.083 |
|  | Spain | 1 (20, 42) | 0.476 (0.332, 0.625) | 0.00% | 0.758 | >0.999 |  |
|  | Tunis | 1 (1, 12) | 0.083 (0.012, 0.413) | 0.00% | 0.022 | >0.999 |  |
|  | Mexico | 1 (3, 119) | 0.025 (0.008, 0.075) | 0.00% | <0.001 | >0.999 |  |
|  | Japan | 1 (2, 10) | 0.200 (0.050, 0.541) | 0.00% | 0.080 | >0.999 |  |
|  | United States | 3 (75, 168) | 0.512 (0.250, 0.768) | 88.99% | 0.934 | <0.001 |  |
|  | Iran | 1 (30, 150) | 0.200 (0.144, 0.272) | 0.00% | <0.001 | >0.999 |  |
|  | European country | 1 (15, 27) | 0.556 (0.369, 0.728) | 0.00% | 0.565 | >0.999 |  |
|  | France | 1 (48, 282) | 0.170 (0.131, 0.219) | 0.00% | <0.001 | >0.999 |  |
|  | China | 1 (15, 114) | 0.132 (0.081, 0.207) | 0.00% | <0.001 | >0.999 |  |
| Continent | Europe | 5 (99, 402) | 0.354 (0.193, 0.557) | 88.72% | 0.156 | <0.001 | 0.432 |
|  | Africa | 1 (1, 12) | 0.083 (0.012, 0.413) | 0.00% | 0.022 | >0.999 |  |
|  | North America | 4 (78, 287) | 0.308 (0.092, 0.663) | 93.93% | 0.285 | <0.001 |  |
|  | Asia | 3 (47, 274) | 0.173 (0.130, 0.227) | 7.62% | <0.001 | 0.339 |  |
| **Tetracycline** | | | | | | | |
| Subgroup | Proportion | K (n, N) | Proportion (LCI, HCI) | I^2^ | P1 | P2 | P3 |
| Overall |  | 23 (1325, 2719) | 0.737 (0.594, 0.843) | 96.82% | 0.002 | <0.001 | NA |
| Year | 2011-2023 | 5 (82, 296) | 0.423 (0.205, 0.675) | 86.89% | 0.557 | <0.001 | 0.053 |
|  | 1958-2010 | 17 (1241, 2419) | 0.805 (0.659, 0.899) | 97.36% | <0.001 | <0.001 |  |
| Guideline | CLSI | 11 (366, 1360) | 0.630 (0.360, 0.838) | 97.12% | 0.347 | <0.001 | 0.077 |
|  | Other | 12 (959, 1359) | 0.799 (0.680, 0.881) | 93.28% | <0.001 | <0.001 |  |
| Quality score | Low Quality | 1 (8, 8) | 0.944 (0.495, 0.997) | 0.00% | 0.052 | >0.999 | 0.159 |
|  | Moderate Quality | 13 (666, 1514) | 0.837 (0.547, 0.956) | 97.92% | 0.027 | <0.001 |  |
|  | High Quality | 9 (651, 1197) | 0.540 (0.418, 0.658) | 91.57% | 0.520 | <0.001 |  |
| AST Method | DM | 17 (941, 2176) | 0.714 (0.557, 0.833) | 96.70% | 0.009 | <0.001 | 0.429 |
|  | DD | 5 (123, 266) | 0.743 (0.254, 0.961) | 94.46% | 0.331 | <0.001 |  |
|  | AS | 1 (261, 277) | 0.942 (0.908, 0.964) | 0.00% | <0.001 | >0.999 |  |
| Country | Korea | 1 (8, 8) | 0.944 (0.495, 0.997) | 0.00% | 0.052 | >0.999 | 0.772 |
|  | Tunis | 1 (12, 12) | 0.962 (0.597, 0.998) | 0.00% | 0.026 | >0.999 |  |
|  | Multi-Country | 3 (147, 900) | 0.430 (0.047, 0.920) | 99.12% | 0.839 | <0.001 |  |
|  | Italy | 2 (292, 308) | 0.945 (0.912, 0.965) | 0.00% | <0.001 | 0.351 |  |
|  | Japan | 1 (64, 66) | 0.970 (0.887, 0.992) | 0.00% | <0.001 | >0.999 |  |
|  | China | 2 (116, 191) | 0.867 (0.012, 1.000) | 94.97% | 0.559 | <0.001 |  |
|  | Spain | 2 (91, 98) | 0.924 (0.844, 0.965) | 6.98% | <0.001 | 0.300 |  |
|  | Mexico | 1 (17, 21) | 0.810 (0.588, 0.927) | 0.00% | 0.009 | >0.999 |  |
|  | United Kingdom | 1 (2, 4) | 0.500 (0.123, 0.877) | 0.00% | >0.999 | >0.999 |  |
|  | Switzerland | 1 (32, 33) | 0.970 (0.814, 0.996) | 0.00% | <0.001 | >0.999 |  |
|  | Saudi Arabia | 1 (46, 54) | 0.852 (0.731, 0.924) | 0.00% | <0.001 | >0.999 |  |
|  | Ethiopia | 1 (1, 5) | 0.200 (0.027, 0.691) | 0.00% | 0.215 | >0.999 |  |
|  | Iran | 2 (89, 257) | 0.381 (0.096, 0.781) | 97.42% | 0.589 | <0.001 |  |
|  | Canada | 1 (72, 119) | 0.605 (0.515, 0.689) | 0.00% | 0.023 | >0.999 |  |
|  | European country | 1 (94, 192) | 0.490 (0.420, 0.560) | 0.00% | 0.773 | >0.999 |  |
|  | Latin America | 1 (40, 83) | 0.482 (0.377, 0.589) | 0.00% | 0.742 | >0.999 |  |
|  | United States | 1 (202, 368) | 0.549 (0.498, 0.599) | 0.00% | 0.061 | >0.999 |  |
| Continent | Asia | 8 (383, 656) | 0.755 (0.505, 0.903) | 95.55% | 0.046 | <0.001 | 0.203 |
|  | Africa | 2 (13, 17) | 0.696 (0.025, 0.995) | 84.30% | 0.720 | 0.012 |  |
|  | Multi Continent | 2 (87, 820) | 0.274 (0.012, 0.919) | 99.14% | 0.576 | <0.001 |  |
|  | Europe | 7 (511, 635) | 0.895 (0.663, 0.973) | 95.24% | 0.004 | <0.001 |  |
|  | North America | 3 (291, 508) | 0.605 (0.505, 0.697) | 64.65% | 0.040 | 0.059 |  |
|  | South America | 1 (40, 83) | 0.482 (0.377, 0.589) | 0.00% | 0.742 | >0.999 |  |
| **Chloramphenicol** | | | | | | | |
| Subgroup | Proportion | K (n, N) | Proportion (LCI, HCI) | I^2^ | P1 | P2 | P3 |
| Overall |  | 56 (1666, 5815) | 0.292 (0.237, 0.353) | 93.77% | <0.001 | <0.001 | NA |
| Year | 1958-2010 | 21 (793, 2380) | 0.356 (0.258, 0.468) | 94.98% | 0.012 | <0.001 | 0.233 |
|  | 2011-2023 | 30 (765, 2866) | 0.275 (0.202, 0.363) | 93.12% | <0.001 | <0.001 |  |
| Guideline | CLSI | 45 (1358, 4901) | 0.296 (0.232, 0.369) | 94.43% | <0.001 | <0.001 | 0.735 |
|  | Other | 11 (308, 914) | 0.283 (0.188, 0.403) | 88.78% | <0.001 | <0.001 |  |
| Quality score | Low Quality | 2 (24, 104) | 0.235 (0.164, 0.326) | 0.00% | <0.001 | 0.489 | 0.385 |
|  | Moderate Quality | 40 (1303, 4028) | 0.317 (0.252, 0.390) | 93.41% | <0.001 | <0.001 |  |
|  | High Quality | 14 (339, 1683) | 0.238 (0.153, 0.349) | 92.40% | <0.001 | <0.001 |  |
| AST Method | DD | 14 (272, 979) | 0.317 (0.238, 0.408) | 78.30% | <0.001 | <0.001 | 0.013 |
|  | DM | 36 (1214, 3938) | 0.308 (0.238, 0.387) | 94.55% | <0.001 | <0.001 |  |
|  | AS | 3 (154, 301) | 0.512 (0.455, 0.568) | 0.00% | 0.687 | 0.407 |  |
|  | AS, DD, DM | 1 (5, 26) | 0.192 (0.082, 0.387) | 0.00% | 0.004 | >0.999 |  |
|  | DD, DM | 1 (19, 477) | 0.040 (0.026, 0.062) | 0.00% | <0.001 | >0.999 |  |
|  | AS, DD | 1 (2, 94) | 0.021 (0.005, 0.081) | 0.00% | <0.001 | >0.999 |  |
| Country | India | 3 (32, 210) | 0.126 (0.035, 0.361) | 83.09% | 0.005 | 0.003 | 0.229 |
|  | Egypt | 1 (24, 100) | 0.240 (0.166, 0.333) | 0.00% | <0.001 | >0.999 |  |
|  | Tunis | 1 (1, 12) | 0.083 (0.012, 0.413) | 0.00% | 0.022 | >0.999 |  |
|  | Iran | 1 (32, 117) | 0.274 (0.200, 0.361) | 0.00% | <0.001 | >0.999 |  |
|  | China | 14 (818, 2664) | 0.309 (0.205, 0.438) | 96.67% | 0.004 | <0.001 |  |
|  | Mexico | 2 (30, 140) | 0.216 (0.155, 0.292) | 0.00% | <0.001 | 0.390 |  |
|  | Italy | 3 (197, 393) | 0.490 (0.420, 0.561) | 32.86% | 0.788 | 0.225 |  |
|  | Serbia | 1 (0, 88) | 0.006 (0.000, 0.083) | 0.00% | <0.001 | >0.999 |  |
|  | Japan | 2 (68, 247) | 0.374 (0.017, 0.955) | 98.80% | 0.778 | <0.001 |  |
|  | Saudi Arabia | 2 (79, 191) | 0.381 (0.237, 0.550) | 74.04% | 0.166 | 0.050 |  |
|  | United States | 5 (82, 297) | 0.449 (0.175, 0.759) | 93.18% | 0.767 | <0.001 |  |
|  | Tunisia | 1 (18, 33) | 0.545 (0.377, 0.704) | 0.00% | 0.602 | >0.999 |  |
|  | Spain | 2 (40, 98) | 0.409 (0.303, 0.526) | 25.12% | 0.125 | 0.248 |  |
|  | Turkey | 2 (25, 141) | 0.234 (0.071, 0.548) | 87.19% | 0.092 | 0.005 |  |
|  | Australia | 1 (9, 45) | 0.200 (0.107, 0.342) | 0.00% | <0.001 | >0.999 |  |
|  | Brazil | 4 (13, 207) | 0.080 (0.014, 0.342) | 85.56% | 0.007 | <0.001 |  |
|  | United Kingdom | 1 (0, 4) | 0.100 (0.006, 0.674) | 0.00% | 0.140 | >0.999 |  |
|  | Hungary | 1 (13, 30) | 0.433 (0.271, 0.612) | 0.00% | 0.467 | >0.999 |  |
|  | Thailand | 2 (45, 181) | 0.265 (0.118, 0.492) | 87.75% | 0.043 | 0.004 |  |
|  | Nepal | 1 (1, 7) | 0.143 (0.020, 0.581) | 0.00% | 0.097 | >0.999 |  |
|  | Malawi | 1 (1, 1) | 0.750 (0.109, 0.987) | 0.00% | 0.501 | >0.999 |  |
|  | France | 1 (73, 106) | 0.689 (0.595, 0.769) | 0.00% | <0.001 | >0.999 |  |
|  | Canada | 1 (8, 31) | 0.258 (0.135, 0.437) | 0.00% | 0.010 | >0.999 |  |
|  | Ethiopia | 1 (5, 5) | 0.917 (0.378, 0.995) | 0.00% | 0.105 | >0.999 |  |
|  | Korea | 1 (19, 90) | 0.211 (0.139, 0.307) | 0.00% | <0.001 | >0.999 |  |
|  | Taiwan | 1 (33, 377) | 0.088 (0.063, 0.121) | 0.00% | <0.001 | >0.999 |  |
| continent | Asia | 27 (1127, 4084) | 0.271 (0.199, 0.358) | 95.79% | <0.001 | <0.001 | 0.178 |
|  | Africa | 5 (49, 151) | 0.414 (0.181, 0.693) | 78.20% | 0.556 | 0.001 |  |
|  | North America | 8 (120, 468) | 0.350 (0.203, 0.533) | 88.56% | 0.107 | <0.001 |  |
|  | Europe | 11 (348, 860) | 0.379 (0.269, 0.503) | 87.97% | 0.056 | <0.001 |  |
|  | Australia | 1 (9, 45) | 0.200 (0.107, 0.342) | 0.00% | <0.001 | >0.999 |  |
|  | South America | 4 (13, 207) | 0.080 (0.014, 0.342) | 85.56% | 0.007 | <0.001 |  |
| **Ticarcillin/Clavulanate** | | | | | | | |
| Subgroup | Proportion | K (n, N) | Proportion (LCI, HCI) | I^2^ | P1 | P2 | P3 |
| Overall |  | 85 (2753, 11059) | 0.313 (0.265, 0.364) | 94.91% | <0.001 | <0.001 | NA |
| year | 1958-2010 | 47 (1709, 7419) | 0.314 (0.252, 0.383) | 95.56% | <0.001 | <0.001 | 0.993 |
|  | 2011-2023 | 32 (835, 2902) | 0.313 (0.234, 0.404) | 93.96% | <0.001 | <0.001 |  |
| Guideline | Other | 28 (999, 3997) | 0.305 (0.228, 0.395) | 95.18% | <0.001 | <0.001 | 0.478 |
|  | EUCAST | 3 (71, 392) | 0.182 (0.147, 0.224) | 0.00% | <0.001 | 0.546 |  |
|  | CLSI | 54 (1683, 6670) | 0.324 (0.262, 0.393) | 94.95% | <0.001 | <0.001 |  |
| Quality score | Low Quality | 3 (118, 204) | 0.562 (0.405, 0.708) | 66.18% | 0.440 | 0.052 | 0.033 |
|  | Moderate Quality | 47 (1576, 5772) | 0.360 (0.291, 0.437) | 95.08% | <0.001 | <0.001 |  |
|  | High Quality | 35 (1059, 5083) | 0.244 (0.186, 0.314) | 94.62% | <0.001 | <0.001 |  |
| AST Method | DM | 51 (1990, 8609) | 0.302 (0.238, 0.374) | 96.42% | <0.001 | <0.001 | 0.685 |
|  | DD | 22 (456, 1213) | 0.350 (0.283, 0.423) | 80.70% | <0.001 | <0.001 |  |
|  | AS | 6 (94, 314) | 0.386 (0.198, 0.615) | 90.38% | 0.328 | <0.001 |  |
|  | DD, AS | 2 (50, 299) | 0.168 (0.129, 0.214) | 0.00% | <0.001 | 0.575 |  |
|  | DD, DM | 3 (141, 522) | 0.304 (0.217, 0.408) | 43.34% | <0.001 | 0.171 |  |
| Country | France | 5 (237, 639) | 0.449 (0.215, 0.707) | 97.02% | 0.710 | <0.001 | 0.015 |
|  | Tunis | 2 (6, 20) | 0.291 (0.084, 0.647) | 42.64% | 0.243 | 0.187 |  |
|  | United States | 11 (398, 1034) | 0.455 (0.285, 0.636) | 95.55% | 0.631 | <0.001 |  |
|  | Korea | 6 (147, 334) | 0.453 (0.304, 0.610) | 82.07% | 0.558 | <0.001 |  |
|  | Taiwan | 13 (335, 1161) | 0.287 (0.222, 0.362) | 80.70% | <0.001 | <0.001 |  |
|  | China | 15 (633, 2401) | 0.243 (0.156, 0.357) | 96.08% | <0.001 | <0.001 |  |
|  | Multi-Country | 6 (421, 3697) | 0.153 (0.085, 0.260) | 96.58% | <0.001 | <0.001 |  |
|  | India | 3 (24, 212) | 0.175 (0.052, 0.450) | 84.61% | 0.025 | 0.002 |  |
|  | Italy | 4 (201, 414) | 0.447 (0.301, 0.602) | 79.92% | 0.504 | 0.002 |  |
|  | Saudi Arabia | 1 (13, 48) | 0.271 (0.164, 0.412) | 0.00% | 0.002 | >0.999 |  |
|  | Spain | 2 (45, 98) | 0.459 (0.363, 0.558) | 0.00% | 0.420 | 0.865 |  |
|  | Germany | 3 (54, 162) | 0.383 (0.174, 0.647) | 88.60% | 0.387 | <0.001 |  |
|  | Switzerland | 1 (29, 33) | 0.879 (0.718, 0.954) | 0.00% | <0.001 | >0.999 |  |
|  | Nepal | 1 (3, 7) | 0.429 (0.144, 0.770) | 0.00% | 0.706 | >0.999 |  |
|  | United Kingdom | 1 (27, 40) | 0.675 (0.517, 0.801) | 0.00% | 0.030 | >0.999 |  |
|  | Egypt | 1 (12, 32) | 0.375 (0.227, 0.551) | 0.00% | 0.162 | >0.999 |  |
|  | Canada | 2 (49, 150) | 0.739 (0.009, 0.999) | 93.91% | 0.722 | <0.001 |  |
|  | Georgia | 1 (1, 20) | 0.050 (0.007, 0.282) | 0.00% | 0.004 | >0.999 |  |
|  | England | 1 (46, 100) | 0.460 (0.365, 0.558) | 0.00% | 0.424 | >0.999 |  |
|  | European country | 1 (27, 192) | 0.141 (0.098, 0.197) | 0.00% | <0.001 | >0.999 |  |
|  | Latin America | 1 (11, 83) | 0.133 (0.075, 0.224) | 0.00% | <0.001 | >0.999 |  |
|  | Brazil | 2 (4, 85) | 0.051 (0.009, 0.242) | 43.55% | 0.001 | 0.183 |  |
|  | Turkey | 1 (10, 33) | 0.303 (0.171, 0.477) | 0.00% | 0.028 | >0.999 |  |
|  | Malaysia | 1 (20, 64) | 0.312 (0.211, 0.435) | 0.00% | 0.003 | >0.999 |  |
| Continent | Europe | 20 (677, 1731) | 0.428 (0.328, 0.533) | 92.93% | 0.178 | <0.001 | <0.001 |
|  | Africa | 3 (18, 52) | 0.360 (0.239, 0.502) | 0.00% | 0.053 | 0.398 |  |
|  | North America | 13 (447, 1184) | 0.463 (0.297, 0.637) | 95.47% | 0.682 | <0.001 |  |
|  | Asia | 41 (1198, 4307) | 0.276 (0.223, 0.336) | 92.36% | <0.001 | <0.001 |  |
|  | Multi Continent | 5 (398, 3617) | 0.134 (0.069, 0.244) | 97.01% | <0.001 | <0.001 |  |
|  | South America | 3 (15, 168) | 0.096 (0.045, 0.194) | 38.28% | <0.001 | 0.198 |  |
| **Amikacin** | | | | | | | |
| Subgroup | Proportion | K (n, N) | Proportion (LCI, HCI) | I^2^ | P1 | P2 | P3 |
| Overall |  | 81 (4454, 6124) | 0.713 (0.667, 0.754) | 88.72% | <0.001 | <0.001 | NA |
| Year | 2011-2023 | 27 (1151, 1729) | 0.662 (0.569, 0.743) | 85.37% | <0.001 | <0.001 | 0.135 |
|  | 1958-2010 | 51 (3245, 4295) | 0.739 (0.685, 0.787) | 89.62% | <0.001 | <0.001 |  |
| Guideline | CLSI | 50 (1897, 2494) | 0.742 (0.672, 0.801) | 86.90% | <0.001 | <0.001 | 0.273 |
|  | Other | 26 (1867, 2608) | 0.700 (0.627, 0.764) | 90.88% | <0.001 | <0.001 |  |
|  | EUCAST | 3 (606, 852) | 0.628 (0.206, 0.917) | 91.71% | 0.583 | <0.001 |  |
|  | CLSI-EUCAST | 2 (84, 170) | 0.494 (0.420, 0.569) | 0.00% | 0.878 | 0.759 |  |
| Quality score | Low Quality | 10 (140, 194) | 0.705 (0.552, 0.822) | 65.34% | 0.010 | 0.002 | 0.883 |
|  | Moderate Quality | 48 (2896, 3874) | 0.705 (0.642, 0.762) | 89.51% | <0.001 | <0.001 |  |
|  | High Quality | 23 (1418, 2056) | 0.726 (0.647, 0.793) | 89.84% | <0.001 | <0.001 |  |
| AST Method | DM | 40 (3258, 4405) | 0.765 (0.711, 0.811) | 91.12% | <0.001 | <0.001 | 0.028 |
|  | DD | 31 (791, 1155) | 0.648 (0.565, 0.723) | 76.40% | <0.001 | <0.001 |  |
|  | AS | 7 (371, 517) | 0.552 (0.283, 0.794) | 94.53% | 0.719 | <0.001 |  |
|  | DD, DM | 1 (1, 6) | 0.167 (0.023, 0.631) | 0.00% | 0.142 | >0.999 |  |
|  | DD, AS | 1 (25, 30) | 0.833 (0.657, 0.929) | 0.00% | 0.001 | >0.999 |  |
|  | DD, DM, AS | 1 (8, 11) | 0.727 (0.414, 0.910) | 0.00% | 0.147 | >0.999 |  |
| Country | Saudi Arabia | 6 (178, 288) | 0.617 (0.559, 0.672) | 0.00% | <0.001 | 0.481 | 0.322 |
|  | Canada | 3 (185, 258) | 0.724 (0.569, 0.839) | 80.91% | 0.006 | 0.005 |  |
|  | Taiwan | 9 (295, 485) | 0.652 (0.472, 0.796) | 89.99% | 0.096 | <0.001 |  |
|  | India | 5 (10, 18) | 0.491 (0.203, 0.786) | 29.94% | 0.959 | 0.222 |  |
|  | Spain | 5 (137, 165) | 0.826 (0.759, 0.877) | 0.00% | <0.001 | 0.732 |  |
|  | Serbia | 2 (36, 38) | 0.931 (0.788, 0.980) | 0.00% | <0.001 | 0.549 |  |
|  | Tunis | 1 (8, 8) | 0.944 (0.495, 0.997) | 0.00% | 0.052 | >0.999 |  |
|  | Korea | 3 (41, 57) | 0.737 (0.136, 0.980) | 90.29% | 0.483 | <0.001 |  |
|  | Mexico | 1 (81, 119) | 0.681 (0.592, 0.758) | 0.00% | <0.001 | >0.999 |  |
|  | Japan | 3 (113, 130) | 0.861 (0.774, 0.918) | 20.40% | <0.001 | 0.285 |  |
|  | Multi-Country | 5 (822, 952) | 0.821 (0.699, 0.901) | 79.96% | <0.001 | <0.001 |  |
|  | United States | 6 (460, 697) | 0.682 (0.482, 0.831) | 93.50% | 0.073 | <0.001 |  |
|  | Turkey | 5 (233, 314) | 0.645 (0.406, 0.828) | 89.35% | 0.231 | <0.001 |  |
|  | Italy | 4 (486, 641) | 0.738 (0.442, 0.909) | 95.92% | 0.109 | <0.001 |  |
|  | China | 8 (439, 579) | 0.780 (0.621, 0.885) | 85.72% | 0.001 | <0.001 |  |
|  | Iran | 3 (32, 33) | 0.812 (0.168, 0.989) | 66.70% | 0.349 | 0.050 |  |
|  | Hungary | 1 (592, 817) | 0.725 (0.693, 0.754) | 0.00% | <0.001 | >0.999 |  |
|  | Switzerland | 1 (31, 33) | 0.939 (0.788, 0.985) | 0.00% | <0.001 | >0.999 |  |
|  | European country | 2 (92, 219) | 0.346 (0.162, 0.591) | 78.39% | 0.214 | 0.031 |  |
|  | Malawi | 1 (1, 1) | 0.750 (0.109, 0.987) | 0.00% | 0.501 | >0.999 |  |
|  | France | 1 (10, 13) | 0.769 (0.478, 0.924) | 0.00% | 0.067 | >0.999 |  |
|  | Egypt | 1 (19, 32) | 0.594 (0.419, 0.747) | 0.00% | 0.292 | >0.999 |  |
|  | Thailand | 1 (39, 64) | 0.609 (0.486, 0.720) | 0.00% | 0.083 | >0.999 |  |
|  | Germany | 1 (22, 24) | 0.917 (0.721, 0.979) | 0.00% | 0.001 | >0.999 |  |
|  | Israel | 1 (5, 10) | 0.500 (0.225, 0.775) | 0.00% | >0.999 | >0.999 |  |
|  | Latin America | 1 (61, 83) | 0.735 (0.630, 0.819) | 0.00% | <0.001 | >0.999 |  |
|  | Brazil | 1 (26, 46) | 0.565 (0.421, 0.700) | 0.00% | 0.378 | >0.999 |  |
| Continent | Asia | 40 (1217, 1744) | 0.690 (0.616, 0.756) | 83.55% | <0.001 | <0.001 | 0.763 |
|  | North America | 10 (726, 1074) | 0.691 (0.578, 0.785) | 90.09% | 0.001 | <0.001 |  |
|  | Europe | 22 (1639, 2264) | 0.743 (0.660, 0.811) | 90.45% | <0.001 | <0.001 |  |
|  | Africa | 3 (28, 41) | 0.709 (0.399, 0.900) | 28.54% | 0.179 | 0.247 |  |
|  | Multi Continent | 4 (757, 872) | 0.805 (0.612, 0.915) | 82.31% | 0.004 | <0.001 |  |
|  | South America | 2 (87, 129) | 0.659 (0.479, 0.802) | 73.80% | 0.082 | 0.051 |  |
| **Gentamicin** | | | | | | | |
| Subgroup | Proportion | K (n, N) | Proportion (LCI, HCI) | I^2^ | P1 | P2 | P3 |
| Overall |  | 68 (4666, 5951) | 0.750 (0.706, 0.789) | 88.21% | <0.001 | <0.001 | NA |
| Year | 2011-2023 | 21 (411, 596) | 0.749 (0.650, 0.828) | 71.73% | <0.001 | <0.001 | 0.768 |
|  | 1958-2010 | 44 (4208, 5269) | 0.761 (0.713, 0.804) | 90.13% | <0.001 | <0.001 |  |
| Guideline | CLSI | 38 (1880, 2433) | 0.759 (0.690, 0.816) | 87.49% | <0.001 | <0.001 | 0.536 |
|  | Other | 29 (2778, 3510) | 0.740 (0.678, 0.793) | 89.50% | <0.001 | <0.001 |  |
|  | EUCAST | 1 (8, 8) | 0.944 (0.495, 0.997) | 0.00% | 0.052 | >0.999 |  |
| Quality score | Low Quality | 8 (126, 164) | 0.768 (0.619, 0.870) | 62.29% | <0.001 | 0.010 | 0.904 |
|  | Moderate Quality | 43 (3478, 4334) | 0.742 (0.691, 0.787) | 86.77% | <0.001 | <0.001 |  |
|  | High Quality | 17 (1062, 1453) | 0.765 (0.663, 0.844) | 91.31% | <0.001 | <0.001 |  |
| AST Method | DM | 35 (3396, 4264) | 0.775 (0.715, 0.826) | 91.88% | <0.001 | <0.001 | 0.545 |
|  | DD | 27 (942, 1304) | 0.702 (0.654, 0.746) | 51.48% | <0.001 | 0.001 |  |
|  | AS | 4 (296, 343) | 0.676 (0.181, 0.952) | 95.89% | 0.520 | <0.001 |  |
|  | DD, AS | 1 (23, 26) | 0.885 (0.697, 0.962) | 0.00% | <0.001 | >0.999 |  |
|  | DD, DM, AS | 1 (9, 14) | 0.643 (0.376, 0.843) | 0.00% | 0.292 | >0.999 |  |
| Country | Saudi Arabia | 7 (391, 504) | 0.730 (0.609, 0.825) | 80.75% | <0.001 | <0.001 | 0.543 |
|  | Canada | 2 (89, 139) | 0.714 (0.411, 0.900) | 83.94% | 0.160 | 0.013 |  |
|  | India | 3 (6, 11) | 0.489 (0.125, 0.865) | 43.03% | 0.963 | 0.173 |  |
|  | Serbia | 2 (35, 38) | 0.897 (0.742, 0.964) | 0.00% | <0.001 | 0.364 |  |
|  | Tunis | 1 (8, 8) | 0.944 (0.495, 0.997) | 0.00% | 0.052 | >0.999 |  |
|  | Sweden | 2 (24, 49) | 0.490 (0.354, 0.627) | 0.00% | 0.887 | 0.869 |  |
|  | Korea | 3 (42, 57) | 0.780 (0.127, 0.989) | 90.64% | 0.438 | <0.001 |  |
|  | Mexico | 1 (93, 119) | 0.782 (0.698, 0.847) | 0.00% | <0.001 | >0.999 |  |
|  | Multi-Country | 1 (682, 763) | 0.894 (0.870, 0.914) | 0.00% | <0.001 | >0.999 |  |
|  | United States | 7 (1263, 1593) | 0.745 (0.578, 0.862) | 94.52% | 0.005 | <0.001 |  |
|  | Turkey | 2 (219, 245) | 0.845 (0.350, 0.982) | 96.36% | 0.151 | <0.001 |  |
|  | Italy | 8 (766, 1006) | 0.746 (0.639, 0.830) | 89.52% | <0.001 | <0.001 |  |
|  | China | 6 (411, 528) | 0.830 (0.690, 0.915) | 83.96% | <0.001 | <0.001 |  |
|  | Japan | 1 (54, 66) | 0.818 (0.707, 0.894) | 0.00% | <0.001 | >0.999 |  |
|  | Somalia | 1 (15, 17) | 0.882 (0.632, 0.970) | 0.00% | 0.007 | >0.999 |  |
|  | Spain | 3 (129, 178) | 0.763 (0.554, 0.893) | 84.66% | 0.016 | 0.001 |  |
|  | Australia | 1 (35, 45) | 0.778 (0.634, 0.876) | 0.00% | <0.001 | >0.999 |  |
|  | Belgium | 2 (83, 121) | 0.673 (0.297, 0.909) | 93.19% | 0.372 | <0.001 |  |
|  | United Kingdom | 1 (1, 4) | 0.250 (0.034, 0.762) | 0.00% | 0.341 | >0.999 |  |
|  | Switzerland | 1 (33, 33) | 0.985 (0.804, 0.999) | 0.00% | 0.003 | >0.999 |  |
|  | Malawi | 1 (1, 1) | 0.750 (0.109, 0.987) | 0.00% | 0.501 | >0.999 |  |
|  | Taiwan | 3 (102, 120) | 0.846 (0.682, 0.934) | 69.03% | <0.001 | 0.040 |  |
|  | Egypt | 1 (19, 32) | 0.594 (0.419, 0.747) | 0.00% | 0.292 | >0.999 |  |
|  | Thailand | 1 (42, 64) | 0.656 (0.533, 0.762) | 0.00% | 0.014 | >0.999 |  |
|  | Iran | 3 (65, 125) | 0.673 (0.101, 0.974) | 81.41% | 0.627 | 0.005 |  |
|  | Germany | 1 (22, 24) | 0.917 (0.721, 0.979) | 0.00% | 0.001 | >0.999 |  |
|  | Ethiopia | 1 (2, 5) | 0.400 (0.100, 0.800) | 0.00% | 0.657 | >0.999 |  |
|  | Israel | 1 (6, 10) | 0.600 (0.297, 0.842) | 0.00% | 0.530 | >0.999 |  |
|  | Brazil | 1 (28, 46) | 0.609 (0.462, 0.738) | 0.00% | 0.144 | >0.999 |  |
| Continent | Asia | 28 (1119, 1485) | 0.748 (0.671, 0.813) | 83.97% | <0.001 | <0.001 | 0.878 |
|  | North America | 10 (1445, 1851) | 0.739 (0.619, 0.832) | 93.10% | <0.001 | <0.001 |  |
|  | Europe | 22 (1312, 1698) | 0.754 (0.677, 0.818) | 87.37% | <0.001 | <0.001 |  |
|  | Africa | 5 (45, 63) | 0.717 (0.475, 0.876) | 46.74% | 0.077 | 0.111 |  |
|  | Multi Continent | 1 (682, 763) | 0.894 (0.870, 0.914) | 0.00% | <0.001 | >0.999 |  |
|  | Australia | 1 (35, 45) | 0.778 (0.634, 0.876) | 0.00% | <0.001 | >0.999 |  |
|  | South America | 1 (28, 46) | 0.609 (0.462, 0.738) | 0.00% | 0.144 | >0.999 |  |
| **Meropenem** | | | | | | | |
| Subgroup | Proportion | K (n, N) | Proportion (LCI, HCI) | I^2^ | P1 | P2 | P3 |
| Overall |  | 51 (3043, 3386) | 0.904 (0.863, 0.934) | 82.65% | <0.001 | <0.001 | NA |
| Year | 2011-2023 | 24 (1175, 1356) | 0.882 (0.788, 0.938) | 85.29% | <0.001 | <0.001 | 0.264 |
|  | 1958-2010 | 24 (1836, 1996) | 0.922 (0.874, 0.953) | 81.74% | <0.001 | <0.001 |  |
| AST Method | CLSI | 34 (1345, 1584) | 0.864 (0.777, 0.920) | 84.19% | <0.001 | <0.001 | 0.016 |
|  | Other | 15 (1609, 1703) | 0.952 (0.925, 0.969) | 66.58% | <0.001 | <0.001 |  |
|  | EUCAST | 2 (89, 99) | 0.927 (0.143, 0.999) | 89.01% | 0.250 | 0.003 |  |
| Quality score | Low Quality | 5 (95, 104) | 0.905 (0.780, 0.962) | 37.39% | <0.001 | 0.172 | 0.032 |
|  | Moderate Quality | 32 (1691, 1962) | 0.864 (0.779, 0.920) | 86.42% | <0.001 | <0.001 |  |
|  | High Quality | 14 (1257, 1320) | 0.949 (0.920, 0.968) | 55.97% | <0.001 | 0.006 |  |
| AST Method | AS | 5 (382, 387) | 0.957 (0.832, 0.990) | 49.02% | <0.001 | 0.097 | 0.691 |
|  | DM | 32 (1943, 2200) | 0.911 (0.856, 0.946) | 85.03% | <0.001 | <0.001 |  |
|  | DD | 12 (695, 772) | 0.866 (0.748, 0.934) | 79.34% | <0.001 | <0.001 |  |
|  | DD, DM | 1 (6, 6) | 0.929 (0.423, 0.996) | 0.00% | 0.081 | >0.999 |  |
|  | DD, AS | 1 (17, 21) | 0.810 (0.588, 0.927) | 0.00% | 0.009 | >0.999 |  |
| Country | India | 3 (6, 106) | 0.221 (0.007, 0.920) | 85.75% | 0.505 | <0.001 | 0.010 |
|  | Saudi Arabia | 2 (21, 35) | 0.426 (0.029, 0.948) | 87.56% | 0.855 | 0.005 |  |
|  | Spain | 5 (271, 281) | 0.957 (0.924, 0.977) | 0.00% | <0.001 | 0.529 |  |
|  | Serbia | 2 (36, 38) | 0.946 (0.807, 0.986) | 0.00% | <0.001 | 0.695 |  |
|  | Korea | 1 (8, 8) | 0.944 (0.495, 0.997) | 0.00% | 0.052 | >0.999 |  |
|  | Mexico | 1 (110, 119) | 0.924 (0.861, 0.960) | 0.00% | <0.001 | >0.999 |  |
|  | United States | 4 (210, 251) | 0.807 (0.155, 0.990) | 93.58% | 0.370 | <0.001 |  |
|  | Turkey | 4 (273, 279) | 0.968 (0.924, 0.987) | 18.38% | <0.001 | 0.299 |  |
|  | Italy | 5 (700, 759) | 0.898 (0.803, 0.950) | 85.80% | <0.001 | <0.001 |  |
|  | Taiwan | 3 (91, 98) | 0.940 (0.701, 0.990) | 69.42% | 0.004 | 0.038 |  |
|  | Iran | 3 (244, 250) | 0.964 (0.844, 0.993) | 42.98% | <0.001 | 0.173 |  |
|  | Canada | 2 (193, 208) | 0.931 (0.813, 0.977) | 73.45% | <0.001 | 0.052 |  |
|  | China | 6 (579, 619) | 0.954 (0.865, 0.985) | 67.98% | <0.001 | 0.008 |  |
|  | United Kingdom | 1 (4, 4) | 0.900 (0.326, 0.994) | 0.00% | 0.140 | >0.999 |  |
|  | Japan | 1 (54, 54) | 0.991 (0.871, 0.999) | 0.00% | <0.001 | >0.999 |  |
|  | European country | 1 (17, 27) | 0.630 (0.438, 0.788) | 0.00% | 0.183 | >0.999 |  |
|  | France | 3 (180, 187) | 0.946 (0.809, 0.986) | 58.86% | <0.001 | 0.088 |  |
|  | Malawi | 1 (1, 1) | 0.750 (0.109, 0.987) | 0.00% | 0.501 | >0.999 |  |
|  | Multi-Country | 2 (35, 52) | 0.526 (0.001, 0.999) | 93.79% | 0.977 | <0.001 |  |
|  | England | 1 (10, 10) | 0.955 (0.552, 0.997) | 0.00% | 0.035 | >0.999 |  |
| Continent | Asia | 19 (1003, 1170) | 0.892 (0.769, 0.953) | 86.40% | <0.001 | <0.001 | 0.359 |
|  | Europe | 22 (1491, 1585) | 0.931 (0.893, 0.957) | 73.42% | <0.001 | <0.001 |  |
|  | North America | 7 (513, 578) | 0.875 (0.700, 0.954) | 88.51% | <0.001 | <0.001 |  |
|  | Africa | 1 (1, 1) | 0.750 (0.109, 0.987) | 0.00% | 0.501 | >0.999 |  |
|  | Multi Continent | 2 (35, 52) | 0.526 (0.001, 0.999) | 93.79% | 0.977 | <0.001 |  |
| **Ertapenem** | | | | | | | |
| Subgroup | Proportion | K (n, N) | Proportion (LCI, HCI) | I^2^ | P1 | P2 | P3 |
| Overall |  | 6 (72, 85) | 0.855 (0.665, 0.946) | 49.42% | 0.001 | 0.079 | NA |
| **Imipenem** | | | | | | | |
| Subgroup | Proportion | K (n, N) | Proportion (LCI, HCI) | I^2^ | P1 | P2 | P3 |
| Overall |  | 78 (5149, 5415) | 0.958 (0.934, 0.973) | 86.05% | <0.001 | <0.001 | NA |
| Year | 2011-2023 | 28 (816, 992) | 0.881 (0.770, 0.942) | 85.19% | <0.001 | <0.001 | <0.001 |
|  | 1958-2010 | 45 (4060, 4147) | 0.971 (0.956, 0.980) | 62.85% | <0.001 | <0.001 |  |
| Guideline | CLSI | 44 (1944, 2034) | 0.952 (0.920, 0.971) | 66.18% | <0.001 | <0.001 | <0.001 |
|  | Other | 29 (3051, 3104) | 0.972 (0.954, 0.983) | 61.02% | <0.001 | <0.001 |  |
|  | EUCAST | 3 (96, 107) | 0.899 (0.455, 0.989) | 80.04% | 0.070 | 0.007 |  |
|  | CLSI-EUCAST | 2 (58, 170) | 0.341 (0.236, 0.464) | 61.50% | 0.012 | 0.107 |  |
| Quality score | Low Quality | 11 (386, 402) | 0.943 (0.889, 0.972) | 44.25% | <0.001 | 0.056 | 0.509 |
|  | Moderate Quality | 51 (3511, 3736) | 0.953 (0.916, 0.974) | 88.31% | <0.001 | <0.001 |  |
|  | High Quality | 16 (1252, 1277) | 0.970 (0.949, 0.983) | 27.65% | <0.001 | 0.146 |  |
| AST Method | AS | 8 (462, 579) | 0.807 (0.564, 0.931) | 87.24% | 0.017 | <0.001 | <0.001 |
|  | DM | 42 (3406, 3446) | 0.977 (0.966, 0.985) | 37.92% | <0.001 | 0.008 |  |
|  | DD | 26 (1245, 1352) | 0.909 (0.840, 0.950) | 77.08% | <0.001 | <0.001 |  |
|  | DD, AS | 1 (32, 33) | 0.970 (0.814, 0.996) | 0.00% | <0.001 | >0.999 |  |
|  | DD, DM, AS | 1 (4, 5) | 0.800 (0.309, 0.973) | 0.00% | 0.215 | >0.999 |  |
| Country | India | 5 (16, 18) | 0.799 (0.466, 0.948) | 22.78% | 0.075 | 0.269 | 0.002 |
|  | Saudi Arabia | 6 (439, 478) | 0.905 (0.699, 0.975) | 82.58% | 0.002 | <0.001 |  |
|  | France | 3 (151, 151) | 0.988 (0.942, 0.998) | 0.00% | <0.001 | 0.647 |  |
|  | Canada | 1 (25, 31) | 0.806 (0.631, 0.910) | 0.00% | 0.002 | >0.999 |  |
|  | Spain | 7 (247, 248) | 0.978 (0.943, 0.992) | 0.00% | <0.001 | 0.751 |  |
|  | Serbia | 2 (36, 38) | 0.946 (0.807, 0.986) | 0.00% | <0.001 | 0.695 |  |
|  | Tunis | 1 (7, 8) | 0.875 (0.463, 0.983) | 0.00% | 0.069 | >0.999 |  |
|  | Sweden | 2 (226, 226) | 0.993 (0.955, 0.999) | 0.00% | <0.001 | 0.334 |  |
|  | Korea | 3 (52, 57) | 0.909 (0.521, 0.989) | 67.38% | 0.042 | 0.047 |  |
|  | United States | 9 (1285, 1295) | 0.965 (0.904, 0.988) | 63.25% | <0.001 | 0.005 |  |
|  | Taiwan | 5 (196, 313) | 0.814 (0.487, 0.953) | 94.34% | 0.058 | <0.001 |  |
|  | Mexico | 1 (119, 119) | 0.996 (0.937, 1.000) | 0.00% | <0.001 | >0.999 |  |
|  | Japan | 3 (80, 80) | 0.968 (0.855, 0.994) | 0.00% | <0.001 | 0.404 |  |
|  | Turkey | 3 (275, 280) | 0.976 (0.938, 0.991) | 20.55% | <0.001 | 0.284 |  |
|  | Italy | 3 (599, 615) | 0.984 (0.886, 0.998) | 68.05% | <0.001 | 0.044 |  |
|  | China | 7 (605, 619) | 0.967 (0.946, 0.979) | 0.00% | <0.001 | 0.574 |  |
|  | Somalia | 1 (15, 17) | 0.882 (0.632, 0.970) | 0.00% | 0.007 | >0.999 |  |
|  | Iran | 2 (249, 249) | 0.996 (0.971, 0.999) | 0.00% | <0.001 | 0.744 |  |
|  | Australia | 1 (30, 30) | 0.984 (0.789, 0.999) | 0.00% | 0.004 | >0.999 |  |
|  | Belgium | 2 (119, 121) | 0.975 (0.883, 0.995) | 26.32% | <0.001 | 0.244 |  |
|  | Switzerland | 1 (33, 33) | 0.985 (0.804, 0.999) | 0.00% | 0.003 | >0.999 |  |
|  | European country | 1 (17, 27) | 0.630 (0.438, 0.788) | 0.00% | 0.183 | >0.999 |  |
|  | Malaysia | 1 (84, 84) | 0.994 (0.913, 1.000) | 0.00% | <0.001 | >0.999 |  |
|  | Malawi | 1 (1, 1) | 0.750 (0.109, 0.987) | 0.00% | 0.501 | >0.999 |  |
|  | United Kingdom | 1 (40, 40) | 0.988 (0.833, 0.999) | 0.00% | 0.002 | >0.999 |  |
|  | Egypt | 1 (0, 32) | 0.015 (0.001, 0.201) | 0.00% | 0.003 | >0.999 |  |
|  | Germany | 1 (24, 24) | 0.980 (0.749, 0.999) | 0.00% | 0.006 | >0.999 |  |
|  | Multi-Country | 2 (87, 89) | 0.977 (0.913, 0.994) | 0.00% | <0.001 | 0.782 |  |
|  | Brazil | 2 (92, 92) | 0.989 (0.928, 0.999) | 0.00% | <0.001 | >0.999 |  |
| Continent | Asia | 32 (1721, 1898) | 0.939 (0.878, 0.971) | 90.10% | <0.001 | <0.001 | 0.062 |
|  | Europe | 26 (1767, 1803) | 0.975 (0.954, 0.987) | 63.71% | <0.001 | <0.001 |  |
|  | North America | 11 (1429, 1445) | 0.962 (0.903, 0.986) | 70.79% | <0.001 | <0.001 |  |
|  | Africa | 4 (23, 58) | 0.589 (0.095, 0.951) | 80.99% | 0.788 | 0.001 |  |
|  | Australia | 1 (30, 30) | 0.984 (0.789, 0.999) | 0.00% | 0.004 | >0.999 |  |
|  | Multi Continent | 2 (87, 89) | 0.977 (0.913, 0.994) | 0.00% | <0.001 | 0.782 |  |
|  | South America | 2 (92, 92) | 0.989 (0.928, 0.999) | 0.00% | <0.001 | >0.999 |  |
| **Colistin** | | | | | | | |
| Subgroup | Proportion | K (n, N) | Proportion (LCI, HCI) | I^2^ | P1 | P2 | P3 |
| Overall |  | 57 (3049, 6807) | 0.446 (0.371, 0.525) | 95.90% | 0.180 | <0.001 | NA |
| Year | 2011-2023 | 47 (2353, 5362) | 0.440 (0.351, 0.533) | 96.06% | 0.202 | <0.001 | 0.226 |
|  | 1958-2010 | 6 (456, 1147) | 0.319 (0.203, 0.463) | 92.65% | 0.015 | <0.001 |  |
| Guideline | CLSI | 34 (1578, 3183) | 0.502 (0.412, 0.592) | 93.59% | 0.966 | <0.001 | 0.338 |
|  | Other | 6 (442, 951) | 0.337 (0.189, 0.527) | 96.21% | 0.091 | <0.001 |  |
|  | EUCAST | 13 (894, 2374) | 0.325 (0.141, 0.586) | 98.25% | 0.184 | <0.001 |  |
|  | CLSI-EUCAST | 4 (135, 299) | 0.461 (0.285, 0.648) | 86.56% | 0.689 | <0.001 |  |
| Quality score | Low Quality | 9 (312, 579) | 0.452 (0.283, 0.633) | 87.23% | 0.607 | <0.001 | 0.562 |
|  | Moderate Quality | 39 (2431, 5690) | 0.422 (0.328, 0.522) | 96.91% | 0.126 | <0.001 |  |
|  | High Quality | 9 (306, 538) | 0.581 (0.463, 0.689) | 79.57% | 0.177 | <0.001 |  |
| AST Method | AS | 12 (248, 983) | 0.284 (0.178, 0.423) | 88.00% | 0.003 | <0.001 | 0.042 |
|  | DM | 33 (2638, 5351) | 0.537 (0.440, 0.632) | 96.88% | 0.452 | <0.001 |  |
|  | DD, DM | 2 (24, 70) | 0.240 (0.025, 0.796) | 90.43% | 0.369 | 0.001 |  |
|  | DD | 8 (130, 295) | 0.429 (0.228, 0.656) | 88.64% | 0.547 | <0.001 |  |
|  | AS, DD, DM | 1 (0, 6) | 0.071 (0.004, 0.577) | 0.00% | 0.081 | >0.999 |  |
| Country | India | 1 (0, 4) | 0.100 (0.006, 0.674) | 0.00% | 0.140 | >0.999 | 0.003 |
|  | Saudi Arabia | 2 (9, 55) | 0.155 (0.037, 0.466) | 71.53% | 0.033 | 0.061 |  |
|  | France | 5 (91, 387) | 0.216 (0.128, 0.343) | 82.86% | <0.001 | <0.001 |  |
|  | Tunis | 1 (5, 8) | 0.625 (0.285, 0.875) | 0.00% | 0.484 | >0.999 |  |
|  | Korea | 1 (7, 8) | 0.875 (0.463, 0.983) | 0.00% | 0.069 | >0.999 |  |
|  | Egypt | 2 (37, 132) | 0.246 (0.118, 0.444) | 67.46% | 0.014 | 0.080 |  |
|  | Taiwan | 5 (165, 389) | 0.431 (0.328, 0.541) | 77.63% | 0.220 | 0.001 |  |
|  | Multi-Country | 4 (1264, 1851) | 0.685 (0.653, 0.716) | 50.96% | <0.001 | 0.106 |  |
|  | Italy | 2 (117, 284) | 0.691 (0.101, 0.978) | 78.01% | 0.597 | 0.033 |  |
|  | Brazil | 1 (25, 48) | 0.521 (0.382, 0.657) | 0.00% | 0.773 | >0.999 |  |
|  | Somalia | 1 (17, 17) | 0.972 (0.678, 0.998) | 0.00% | 0.013 | >0.999 |  |
|  | Iran | 2 (1, 45) | 0.148 (0.001, 0.976) | 84.98% | 0.530 | 0.010 |  |
|  | China | 4 (198, 280) | 0.715 (0.588, 0.815) | 75.63% | 0.001 | 0.006 |  |
|  | Peru | 1 (4, 10) | 0.400 (0.158, 0.703) | 0.00% | 0.530 | >0.999 |  |
|  | Spain | 2 (91, 203) | 0.489 (0.145, 0.844) | 96.82% | 0.961 | <0.001 |  |
|  | Hungary | 4 (211, 1149) | 0.468 (0.062, 0.921) | 98.82% | 0.923 | <0.001 |  |
|  | Germany | 2 (72, 155) | 0.441 (0.208, 0.704) | 90.69% | 0.674 | 0.001 |  |
|  | Poland | 2 (3, 102) | 0.076 (0.000, 0.934) | 90.33% | 0.341 | 0.001 |  |
|  | United States | 3 (60, 122) | 0.492 (0.148, 0.844) | 65.60% | 0.971 | 0.055 |  |
|  | European country | 1 (13, 27) | 0.481 (0.304, 0.664) | 0.00% | 0.847 | >0.999 |  |
|  | Thailand | 2 (65, 181) | 0.421 (0.026, 0.952) | 98.43% | 0.851 | <0.001 |  |
|  | Malawi | 1 (0, 1) | 0.250 (0.013, 0.891) | 0.00% | 0.501 | >0.999 |  |
|  | Qatar | 1 (31, 317) | 0.098 (0.070, 0.136) | 0.00% | <0.001 | >0.999 |  |
|  | Argentina | 1 (274, 641) | 0.427 (0.390, 0.466) | 0.00% | <0.001 | >0.999 |  |
|  | Turkey | 4 (281, 368) | 0.741 (0.479, 0.899) | 92.70% | 0.070 | <0.001 |  |
|  | Palestine | 1 (3, 5) | 0.600 (0.200, 0.900) | 0.00% | 0.657 | >0.999 |  |
|  | Israel | 1 (5, 18) | 0.278 (0.121, 0.519) | 0.00% | 0.069 | >0.999 |  |
| Continent | Asia | 20 (484, 1302) | 0.422 (0.290, 0.567) | 93.56% | 0.291 | <0.001 | 0.747 |
|  | Europe | 23 (1429, 3494) | 0.421 (0.287, 0.567) | 97.39% | 0.287 | <0.001 |  |
|  | Africa | 5 (59, 158) | 0.428 (0.194, 0.700) | 74.58% | 0.618 | 0.003 |  |
|  | Multi Continent | 3 (714, 1032) | 0.693 (0.645, 0.737) | 62.46% | <0.001 | 0.070 |  |
|  | South America | 3 (303, 699) | 0.434 (0.397, 0.471) | 0.00% | <0.001 | 0.446 |  |
|  | North America | 3 (60, 122) | 0.492 (0.148, 0.844) | 65.60% | 0.971 | 0.055 |  |
| **Ceftazidime** | | | | | | | |
| Subgroup | Proportion | K (n, N) | Proportion (LCI, HCI) | I^2^ | P1 | P2 | P3 |
| Overall |  | 225 (12986, 26269) | 0.500 (0.471, 0.529) | 93.45% | 0.980 | <0.001 | NA |
| Year | 2011-2023 | 102 (6278, 12333) | 0.479 (0.433, 0.526) | 94.20% | 0.385 | <0.001 | 0.289 |
|  | 1958-2010 | 109 (6254, 12974) | 0.510 (0.472, 0.548) | 92.53% | 0.612 | <0.001 |  |
| Guideline | CLSI | 153 (8579, 17684) | 0.490 (0.454, 0.526) | 93.55% | 0.571 | <0.001 | 0.043 |
|  | Other | 60 (3691, 7330) | 0.516 (0.464, 0.568) | 93.09% | 0.540 | <0.001 |  |
|  | EUCAST | 8 (554, 841) | 0.683 (0.518, 0.812) | 91.36% | 0.031 | <0.001 |  |
|  | CLSI-EUCAST | 4 (162, 414) | 0.336 (0.041, 0.858) | 96.12% | 0.591 | <0.001 |  |
| Quality score | Low Quality | 19 (431, 1030) | 0.586 (0.392, 0.756) | 93.88% | 0.385 | <0.001 | 0.602 |
|  | Moderate Quality | 144 (9057, 17774) | 0.504 (0.470, 0.538) | 92.94% | 0.815 | <0.001 |  |
|  | High Quality | 62 (3498, 7465) | 0.483 (0.427, 0.539) | 94.13% | 0.550 | <0.001 |  |
| AST Method | DM | 134 (9689, 19541) | 0.500 (0.466, 0.535) | 94.29% | 0.986 | <0.001 | 0.906 |
|  | DD | 60 (1739, 3598) | 0.530 (0.462, 0.597) | 89.97% | 0.391 | <0.001 |  |
|  | AS | 18 (869, 1667) | 0.491 (0.307, 0.677) | 96.12% | 0.926 | <0.001 |  |
|  | DD, DM | 5 (259, 571) | 0.414 (0.246, 0.606) | 75.26% | 0.383 | 0.003 |  |
|  | DD, AS | 4 (196, 411) | 0.456 (0.354, 0.563) | 64.28% | 0.422 | 0.038 |  |
|  | AS, DD, DM | 1 (105, 172) | 0.610 (0.536, 0.680) | 0.00% | 0.004 | >0.999 |  |
|  | DD, DM, AS | 1 (5, 12) | 0.417 (0.185, 0.692) | 0.00% | 0.566 | >0.999 |  |
|  | AS, DD | 1 (75, 195) | 0.385 (0.319, 0.455) | 0.00% | 0.001 | >0.999 |  |
| Country | Saudi Arabia | 8 (261, 476) | 0.565 (0.398, 0.718) | 86.23% | 0.448 | <0.001 | <0.001 |
|  | France | 8 (486, 764) | 0.704 (0.575, 0.808) | 89.93% | 0.003 | <0.001 |  |
|  | Canada | 6 (424, 692) | 0.563 (0.444, 0.676) | 86.99% | 0.298 | <0.001 |  |
|  | Taiwan | 23 (948, 1799) | 0.465 (0.371, 0.561) | 89.46% | 0.475 | <0.001 |  |
|  | India | 9 (128, 496) | 0.339 (0.125, 0.648) | 93.68% | 0.305 | <0.001 |  |
|  | Italy | 11 (729, 1116) | 0.612 (0.499, 0.714) | 90.85% | 0.052 | <0.001 |  |
|  | Spain | 6 (136, 245) | 0.559 (0.404, 0.704) | 78.54% | 0.459 | <0.001 |  |
|  | Serbia | 2 (37, 38) | 0.954 (0.799, 0.991) | 0.00% | <0.001 | 0.452 |  |
|  | Tunis | 1 (5, 8) | 0.625 (0.285, 0.875) | 0.00% | 0.484 | >0.999 |  |
|  | Sweden | 3 (32, 247) | 0.191 (0.046, 0.538) | 91.38% | 0.077 | <0.001 |  |
|  | Korea | 9 (244, 547) | 0.465 (0.319, 0.618) | 89.08% | 0.657 | <0.001 |  |
|  | Japan | 7 (263, 396) | 0.648 (0.533, 0.748) | 69.48% | 0.012 | 0.003 |  |
|  | United States | 29 (2796, 4787) | 0.576 (0.513, 0.637) | 91.41% | 0.018 | <0.001 |  |
|  | Egypt | 2 (18, 132) | 0.165 (0.022, 0.640) | 93.81% | 0.148 | <0.001 |  |
|  | Multi-Country | 14 (2766, 6631) | 0.444 (0.359, 0.533) | 97.59% | 0.216 | <0.001 |  |
|  | Iran | 8 (231, 614) | 0.296 (0.122, 0.560) | 95.68% | 0.125 | <0.001 |  |
|  | China | 26 (1450, 3301) | 0.460 (0.385, 0.538) | 93.05% | 0.316 | <0.001 |  |
|  | Mexico | 2 (55, 140) | 0.387 (0.293, 0.491) | 14.41% | 0.034 | 0.280 |  |
|  | Turkey | 11 (730, 1309) | 0.456 (0.294, 0.627) | 95.70% | 0.618 | <0.001 |  |
|  | Somalia | 1 (4, 17) | 0.235 (0.091, 0.486) | 0.00% | 0.039 | >0.999 |  |
|  | Poland | 3 (14, 200) | 0.144 (0.016, 0.635) | 88.70% | 0.134 | <0.001 |  |
|  | Peru | 1 (0, 10) | 0.045 (0.003, 0.448) | 0.00% | 0.035 | >0.999 |  |
|  | Tunisia | 1 (18, 33) | 0.545 (0.377, 0.704) | 0.00% | 0.602 | >0.999 |  |
|  | Australia | 1 (16, 45) | 0.356 (0.231, 0.504) | 0.00% | 0.056 | >0.999 |  |
|  | Brazil | 4 (154, 231) | 0.638 (0.496, 0.760) | 76.31% | 0.057 | 0.005 |  |
|  | Belgium | 2 (34, 121) | 0.282 (0.207, 0.372) | 4.78% | <0.001 | 0.305 |  |
|  | Germany | 5 (156, 276) | 0.654 (0.440, 0.819) | 87.87% | 0.156 | <0.001 |  |
|  | Ireland | 1 (33, 34) | 0.971 (0.819, 0.996) | 0.00% | <0.001 | >0.999 |  |
|  | United Kingdom | 1 (3, 4) | 0.750 (0.238, 0.966) | 0.00% | 0.341 | >0.999 |  |
|  | Switzerland | 1 (19, 33) | 0.576 (0.405, 0.730) | 0.00% | 0.386 | >0.999 |  |
|  | European country | 2 (72, 219) | 0.459 (0.147, 0.807) | 92.77% | 0.839 | <0.001 |  |
|  | Malaysia | 2 (94, 148) | 0.630 (0.499, 0.745) | 60.74% | 0.053 | 0.110 |  |
|  | Hungary | 1 (30, 30) | 0.984 (0.789, 0.999) | 0.00% | 0.004 | >0.999 |  |
|  | Thailand | 2 (100, 181) | 0.517 (0.261, 0.763) | 91.81% | 0.907 | <0.001 |  |
|  | Nepal | 1 (5, 7) | 0.714 (0.327, 0.928) | 0.00% | 0.273 | >0.999 |  |
|  | Malawi | 1 (1, 1) | 0.750 (0.109, 0.987) | 0.00% | 0.501 | >0.999 |  |
|  | Asia-Pacific Region | 1 (143, 204) | 0.701 (0.635, 0.760) | 0.00% | <0.001 | >0.999 |  |
|  | Qatar | 1 (167, 317) | 0.527 (0.472, 0.581) | 0.00% | 0.340 | >0.999 |  |
|  | Slovakia | 1 (12, 53) | 0.226 (0.133, 0.358) | 0.00% | <0.001 | >0.999 |  |
|  | Denmark | 1 (69, 124) | 0.556 (0.468, 0.641) | 0.00% | 0.210 | >0.999 |  |
|  | Israel | 1 (3, 10) | 0.300 (0.100, 0.624) | 0.00% | 0.220 | >0.999 |  |
|  | Georgia | 1 (6, 20) | 0.300 (0.141, 0.527) | 0.00% | 0.082 | >0.999 |  |
|  | England | 2 (56, 110) | 0.509 (0.416, 0.601) | 0.00% | 0.849 | 0.952 |  |
|  | Andalusia | 1 (17, 20) | 0.850 (0.624, 0.951) | 0.00% | 0.006 | >0.999 |  |
|  | Latin America | 1 (21, 83) | 0.253 (0.171, 0.357) | 0.00% | <0.001 | >0.999 |  |
| Continent | Asia | 99 (4079, 8576) | 0.471 (0.425, 0.517) | 91.79% | 0.214 | <0.001 | 0.066 |
|  | Europe | 63 (2671, 4963) | 0.546 (0.480, 0.610) | 92.94% | 0.175 | <0.001 |  |
|  | North America | 37 (3275, 5619) | 0.562 (0.508, 0.614) | 90.69% | 0.025 | <0.001 |  |
|  | Africa | 6 (46, 191) | 0.346 (0.149, 0.615) | 85.70% | 0.259 | <0.001 |  |
|  | Multi Continent | 13 (2724, 6551) | 0.438 (0.350, 0.531) | 97.76% | 0.190 | <0.001 |  |
|  | South America | 6 (175, 324) | 0.504 (0.301, 0.705) | 90.57% | 0.972 | <0.001 |  |
|  | Australia | 1 (16, 45) | 0.356 (0.231, 0.504) | 0.00% | 0.056 | >0.999 |  |
| **Levofloxacin** | | | | | | | |
| Subgroup | Proportion | K (n, N) | Proportion (LCI, HCI) | I^2^ | P1 | P2 | P3 |
| Overall |  | 163 (3743, 26496) | 0.160 (0.139, 0.184) | 93.72% | <0.001 | <0.001 | NA |
| Year | 2011-2023 | 99 (2244, 16849) | 0.151 (0.126, 0.180) | 93.35% | <0.001 | <0.001 | 0.444 |
|  | 1958-2010 | 54 (1387, 8893) | 0.168 (0.130, 0.215) | 94.57% | <0.001 | <0.001 |  |
| AST Method | CLSI | 135 (2990, 20956) | 0.165 (0.142, 0.191) | 92.90% | <0.001 | <0.001 | 0.068 |
|  | Other | 17 (611, 3455) | 0.193 (0.110, 0.316) | 96.82% | <0.001 | <0.001 |  |
|  | CLSI-EUCAST | 3 (65, 952) | 0.088 (0.043, 0.174) | 84.04% | <0.001 | 0.002 |  |
|  | EUCAST | 8 (77, 1133) | 0.074 (0.054, 0.101) | 41.26% | <0.001 | 0.103 |  |
| Quality score | Low Quality | 10 (217, 2503) | 0.128 (0.062, 0.248) | 90.48% | <0.001 | <0.001 | 0.729 |
|  | Moderate Quality | 117 (2867, 18040) | 0.164 (0.139, 0.192) | 93.68% | <0.001 | <0.001 |  |
|  | High Quality | 36 (659, 5953) | 0.160 (0.118, 0.214) | 92.93% | <0.001 | <0.001 |  |
| AST Method | DM | 95 (2608, 16966) | 0.182 (0.154, 0.214) | 93.74% | <0.001 | <0.001 | 0.010 |
|  | DD | 36 (369, 4167) | 0.108 (0.078, 0.148) | 86.39% | <0.001 | <0.001 |  |
|  | AS | 20 (622, 3596) | 0.234 (0.137, 0.369) | 96.94% | <0.001 | <0.001 |  |
|  | DD, AS | 5 (55, 564) | 0.108 (0.069, 0.166) | 62.37% | <0.001 | 0.031 |  |
|  | AS, DD, DM | 1 (53, 324) | 0.164 (0.127, 0.208) | 0.00% | <0.001 | >0.999 |  |
|  | DM, AS | 1 (7, 76) | 0.092 (0.045, 0.181) | 0.00% | <0.001 | >0.999 |  |
|  | DD, DM | 2 (20, 483) | 0.109 (0.010, 0.593) | 87.53% | 0.097 | 0.005 |  |
|  | DD, DM, AS | 1 (0, 18) | 0.026 (0.002, 0.310) | 0.00% | 0.012 | >0.999 |  |
|  | AS, DD | 1 (3, 200) | 0.015 (0.005, 0.045) | 0.00% | <0.001 | >0.999 |  |
| Country | Saudi Arabia | 3 (41, 83) | 0.426 (0.188, 0.704) | 80.05% | 0.615 | 0.007 | <0.001 |
|  | Taiwan | 11 (287, 894) | 0.320 (0.203, 0.464) | 90.77% | 0.016 | <0.001 |  |
|  | Italy | 6 (232, 589) | 0.225 (0.078, 0.498) | 95.76% | 0.048 | <0.001 |  |
|  | Korea | 8 (183, 556) | 0.292 (0.189, 0.422) | 84.28% | 0.002 | <0.001 |  |
|  | Japan | 5 (54, 381) | 0.158 (0.088, 0.268) | 77.30% | <0.001 | 0.001 |  |
|  | Ireland | 2 (8, 44) | 0.187 (0.096, 0.333) | 0.00% | <0.001 | 0.456 |  |
|  | China | 29 (739, 6226) | 0.148 (0.105, 0.204) | 94.27% | <0.001 | <0.001 |  |
|  | Egypt | 3 (16, 152) | 0.139 (0.042, 0.377) | 82.82% | 0.007 | 0.003 |  |
|  | Multi-Country | 11 (665, 6372) | 0.078 (0.044, 0.134) | 97.30% | <0.001 | <0.001 |  |
|  | Argentina | 4 (92, 747) | 0.155 (0.083, 0.270) | 70.23% | <0.001 | 0.018 |  |
|  | Iran | 5 (21, 1108) | 0.020 (0.009, 0.045) | 58.73% | <0.001 | 0.046 |  |
|  | United States | 25 (745, 4153) | 0.220 (0.166, 0.285) | 91.34% | <0.001 | <0.001 |  |
|  | Mexico | 2 (7, 140) | 0.071 (0.004, 0.568) | 90.87% | 0.076 | <0.001 |  |
|  | India | 4 (50, 352) | 0.100 (0.034, 0.260) | 87.58% | <0.001 | <0.001 |  |
|  | France | 3 (42, 490) | 0.089 (0.039, 0.192) | 85.46% | <0.001 | 0.001 |  |
|  | Serbia | 1 (0, 88) | 0.006 (0.000, 0.083) | 0.00% | <0.001 | >0.999 |  |
|  | Spain | 4 (19, 138) | 0.163 (0.055, 0.395) | 76.77% | 0.008 | 0.005 |  |
|  | Brazil | 3 (48, 163) | 0.357 (0.071, 0.801) | 95.31% | 0.561 | <0.001 |  |
|  | Poland | 3 (1, 200) | 0.019 (0.002, 0.207) | 67.14% | 0.003 | 0.048 |  |
|  | Peru | 1 (0, 10) | 0.045 (0.003, 0.448) | 0.00% | 0.035 | >0.999 |  |
|  | South Korea | 2 (75, 220) | 0.330 (0.082, 0.731) | 96.73% | 0.416 | <0.001 |  |
|  | Turkey | 7 (72, 842) | 0.104 (0.068, 0.157) | 67.31% | <0.001 | 0.005 |  |
|  | Hungary | 4 (86, 1149) | 0.079 (0.049, 0.124) | 63.56% | <0.001 | 0.041 |  |
|  | Germany | 4 (61, 252) | 0.262 (0.106, 0.515) | 90.63% | 0.064 | <0.001 |  |
|  | European country | 1 (3, 27) | 0.111 (0.036, 0.293) | 0.00% | <0.001 | >0.999 |  |
|  | Thailand | 2 (20, 217) | 0.090 (0.046, 0.171) | 54.76% | <0.001 | 0.137 |  |
|  | Nepal | 1 (1, 7) | 0.143 (0.020, 0.581) | 0.00% | 0.097 | >0.999 |  |
|  | Malawi | 1 (1, 1) | 0.750 (0.109, 0.987) | 0.00% | 0.501 | >0.999 |  |
|  | Asia-Pacific Region | 1 (54, 204) | 0.265 (0.209, 0.329) | 0.00% | <0.001 | >0.999 |  |
|  | Qatar | 1 (39, 317) | 0.123 (0.091, 0.164) | 0.00% | <0.001 | >0.999 |  |
|  | Greece | 1 (3, 62) | 0.048 (0.016, 0.140) | 0.00% | <0.001 | >0.999 |  |
|  | Georgia | 1 (2, 20) | 0.100 (0.025, 0.324) | 0.00% | 0.003 | >0.999 |  |
|  | England | 1 (43, 100) | 0.430 (0.337, 0.528) | 0.00% | 0.163 | >0.999 |  |
|  | Andalusia | 1 (1, 20) | 0.050 (0.007, 0.282) | 0.00% | 0.004 | >0.999 |  |
|  | Malaysia | 1 (21, 64) | 0.328 (0.225, 0.451) | 0.00% | 0.007 | >0.999 |  |
|  | Canada | 1 (11, 108) | 0.102 (0.057, 0.175) | 0.00% | <0.001 | >0.999 |  |
| Continent | Asia | 73 (1585, 10629) | 0.175 (0.141, 0.215) | 93.60% | <0.001 | <0.001 | 0.057 |
|  | Europe | 39 (573, 4021) | 0.126 (0.085, 0.184) | 93.78% | <0.001 | <0.001 |  |
|  | Africa | 4 (17, 153) | 0.184 (0.057, 0.456) | 79.52% | 0.026 | 0.002 |  |
|  | Multi Continent | 11 (665, 6372) | 0.078 (0.044, 0.134) | 97.30% | <0.001 | <0.001 |  |
|  | South America | 8 (140, 920) | 0.216 (0.099, 0.410) | 92.08% | 0.006 | <0.001 |  |
|  | North America | 28 (763, 4401) | 0.202 (0.153, 0.260) | 90.91% | <0.001 | <0.001 |  |
| **Minocycline** | | | | | | | |
| Subgroup | Proportion | K (n, N) | Proportion (LCI, HCI) | I^2^ | P1 | P2 | P3 |
| Overall |  | 71 (447, 11507) | 0.032 (0.020, 0.051) | 92.38% | <0.001 | <0.001 | NA |
| Year | 2011-2023 | 48 (350, 9380) | 0.034 (0.019, 0.063) | 94.08% | <0.001 | <0.001 | 0.543 |
|  | 1958-2010 | 20 (90, 1760) | 0.024 (0.009, 0.063) | 82.29% | <0.001 | <0.001 |  |
| Guideline | CLSI | 62 (247, 10674) | 0.025 (0.015, 0.041) | 89.89% | <0.001 | <0.001 | 0.003 |
|  | Other | 4 (4, 238) | 0.026 (0.011, 0.059) | 0.00% | <0.001 | 0.750 |  |
|  | EUCAST | 3 (109, 425) | 0.234 (0.096, 0.468) | 93.49% | 0.028 | <0.001 |  |
|  | CLSI-EUCAST | 2 (87, 170) | 0.530 (0.017, 0.987) | 98.63% | 0.955 | <0.001 |  |
| Quality score | Low Quality | 5 (23, 2160) | 0.024 (0.004, 0.129) | 92.71% | <0.001 | <0.001 | 0.554 |
|  | Moderate Quality | 50 (328, 6469) | 0.037 (0.020, 0.067) | 92.27% | <0.001 | <0.001 |  |
|  | High Quality | 16 (96, 2878) | 0.022 (0.009, 0.050) | 89.99% | <0.001 | <0.001 |  |
| AST Method | AS | 12 (116, 2344) | 0.092 (0.012, 0.450) | 95.75% | 0.032 | <0.001 | 0.481 |
|  | DM | 35 (207, 6248) | 0.023 (0.012, 0.045) | 92.72% | <0.001 | <0.001 |  |
|  | DD | 16 (67, 2343) | 0.034 (0.013, 0.085) | 88.80% | <0.001 | <0.001 |  |
|  | DD, DM | 4 (0, 151) | 0.014 (0.004, 0.055) | 0.00% | <0.001 | 0.946 |  |
|  | DD, AS | 3 (57, 377) | 0.061 (0.009, 0.323) | 76.35% | 0.007 | 0.015 |  |
|  | AS, DD | 1 (0, 44) | 0.011 (0.001, 0.154) | 0.00% | 0.002 | >0.999 |  |
| Country | Japan | 5 (0, 326) | 0.013 (0.004, 0.043) | 0.00% | <0.001 | 0.567 | 0.109 |
|  | United States | 13 (60, 1989) | 0.021 (0.005, 0.093) | 92.43% | <0.001 | <0.001 |  |
|  | China | 19 (120, 4813) | 0.025 (0.011, 0.055) | 92.22% | <0.001 | <0.001 |  |
|  | Egypt | 2 (15, 120) | 0.125 (0.077, 0.198) | 0.00% | <0.001 | 0.712 |  |
|  | Taiwan | 4 (88, 590) | 0.083 (0.003, 0.721) | 97.34% | 0.161 | <0.001 |  |
|  | Argentina | 2 (0, 74) | 0.018 (0.002, 0.117) | 0.00% | <0.001 | 0.399 |  |
|  | Iran | 4 (22, 391) | 0.051 (0.010, 0.231) | 82.01% | <0.001 | <0.001 |  |
|  | Multi-Country | 5 (12, 2007) | 0.007 (0.003, 0.016) | 17.33% | <0.001 | 0.304 |  |
|  | Somalia | 1 (15, 17) | 0.882 (0.632, 0.970) | 0.00% | 0.007 | >0.999 |  |
|  | Saudi Arabia | 1 (32, 48) | 0.667 (0.523, 0.785) | 0.00% | 0.024 | >0.999 |  |
|  | Spain | 2 (0, 98) | 0.010 (0.001, 0.068) | 0.00% | <0.001 | 0.968 |  |
|  | Brazil | 1 (0, 100) | 0.005 (0.000, 0.074) | 0.00% | <0.001 | >0.999 |  |
|  | Ireland | 1 (3, 34) | 0.088 (0.029, 0.240) | 0.00% | <0.001 | >0.999 |  |
|  | Thailand | 1 (0, 100) | 0.005 (0.000, 0.074) | 0.00% | <0.001 | >0.999 |  |
|  | India | 1 (0, 106) | 0.005 (0.000, 0.070) | 0.00% | <0.001 | >0.999 |  |
|  | Malawi | 1 (1, 1) | 0.750 (0.109, 0.987) | 0.00% | 0.501 | >0.999 |  |
|  | Korea | 3 (22, 144) | 0.159 (0.001, 0.964) | 91.44% | 0.508 | <0.001 |  |
|  | France | 2 (57, 388) | 0.053 (0.002, 0.576) | 90.28% | 0.077 | 0.001 |  |
|  | Germany | 2 (0, 97) | 0.011 (0.002, 0.076) | 0.00% | <0.001 | 0.622 |  |
|  | Malaysia | 1 (0, 64) | 0.008 (0.000, 0.111) | 0.00% | <0.001 | >0.999 |  |
| Continent | Asia | 39 (284, 6582) | 0.034 (0.017, 0.066) | 93.24% | <0.001 | <0.001 | 0.029 |
|  | North America | 13 (60, 1989) | 0.021 (0.005, 0.093) | 92.43% | <0.001 | <0.001 |  |
|  | Africa | 4 (31, 138) | 0.412 (0.090, 0.833) | 88.85% | 0.723 | <0.001 |  |
|  | South America | 3 (0, 174) | 0.012 (0.002, 0.056) | 0.00% | <0.001 | 0.531 |  |
|  | Multi Continent | 5 (12, 2007) | 0.007 (0.003, 0.016) | 17.33% | <0.001 | 0.304 |  |
|  | Europe | 7 (60, 617) | 0.030 (0.008, 0.108) | 79.86% | <0.001 | <0.001 |  |
| **Tigecycline** | | | | | | | |
| Subgroup | Proportion | K (n, N) | Proportion (LCI, HCI) | I^2^ | P1 | P2 | P3 |
| Overall |  | 45 (1112, 5892) | 0.214 (0.154, 0.291) | 95.68% | <0.001 | <0.001 | NA |
| Year | 1958-2010 | 12 (128, 1868) | 0.082 (0.031, 0.196) | 95.11% | <0.001 | <0.001 | 0.002 |
|  | 2011-2023 | 28 (755, 3073) | 0.302 (0.205, 0.421) | 95.52% | 0.002 | <0.001 |  |
| Guideline | CLSI | 35 (658, 3726) | 0.202 (0.136, 0.289) | 94.21% | <0.001 | <0.001 | 0.248 |
|  | EUCAST | 9 (452, 2109) | 0.296 (0.145, 0.509) | 98.07% | 0.060 | <0.001 |  |
|  | CLSI-EUCAST | 1 (2, 57) | 0.035 (0.009, 0.130) | 0.00% | <0.001 | >0.999 |  |
| Quality score | Low Quality | 3 (10, 94) | 0.174 (0.032, 0.578) | 64.03% | 0.103 | 0.062 | 0.112 |
|  | Moderate Quality | 34 (974, 4549) | 0.256 (0.176, 0.357) | 96.18% | <0.001 | <0.001 |  |
|  | High Quality | 8 (128, 1249) | 0.102 (0.058, 0.174) | 86.37% | <0.001 | <0.001 |  |
| AST Method | DD | 3 (34, 46) | 0.642 (0.034, 0.989) | 84.59% | 0.770 | 0.002 | <0.001 |
|  | DM | 34 (707, 5117) | 0.150 (0.105, 0.209) | 94.19% | <0.001 | <0.001 |  |
|  | AS | 6 (302, 611) | 0.494 (0.264, 0.726) | 94.96% | 0.959 | <0.001 |  |
|  | AS, DD, DM | 1 (11, 16) | 0.688 (0.433, 0.864) | 0.00% | 0.144 | >0.999 |  |
| Country | Taiwan | 5 (99, 567) | 0.192 (0.019, 0.749) | 97.71% | 0.266 | <0.001 | 0.014 |
|  | Korea | 5 (30, 377) | 0.084 (0.052, 0.132) | 35.09% | <0.001 | 0.187 |  |
|  | Multi-Country | 3 (68, 1037) | 0.052 (0.004, 0.416) | 97.78% | 0.026 | <0.001 |  |
|  | France | 3 (91, 280) | 0.288 (0.092, 0.617) | 95.52% | 0.199 | <0.001 |  |
|  | United States | 5 (49, 171) | 0.333 (0.112, 0.664) | 90.77% | 0.323 | <0.001 |  |
|  | Brazil | 1 (4, 48) | 0.083 (0.032, 0.202) | 0.00% | <0.001 | >0.999 |  |
|  | China | 6 (167, 1156) | 0.149 (0.110, 0.200) | 67.10% | <0.001 | 0.010 |  |
|  | Saudi Arabia | 1 (45, 48) | 0.938 (0.823, 0.980) | 0.00% | <0.001 | >0.999 |  |
|  | Hungary | 4 (168, 1149) | 0.290 (0.109, 0.577) | 96.49% | 0.146 | <0.001 |  |
|  | Spain | 1 (3, 80) | 0.038 (0.012, 0.110) | 0.00% | <0.001 | >0.999 |  |
|  | Germany | 4 (33, 252) | 0.122 (0.015, 0.553) | 94.25% | 0.077 | <0.001 |  |
|  | Malaysia | 1 (43, 84) | 0.512 (0.406, 0.617) | 0.00% | 0.827 | >0.999 |  |
|  | Turkey | 3 (99, 254) | 0.303 (0.055, 0.765) | 96.83% | 0.418 | <0.001 |  |
|  | United Kingdom | 1 (15, 40) | 0.375 (0.240, 0.532) | 0.00% | 0.118 | >0.999 |  |
|  | Egypt | 1 (32, 32) | 0.985 (0.799, 0.999) | 0.00% | 0.003 | >0.999 |  |
|  | Qatar | 1 (166, 317) | 0.524 (0.469, 0.578) | 0.00% | 0.400 | >0.999 |  |
| Continent | Asia | 19 (550, 2549) | 0.201 (0.117, 0.324) | 96.23% | <0.001 | <0.001 | 0.026 |
|  | Multi Continent | 3 (68, 1037) | 0.052 (0.004, 0.416) | 97.78% | 0.026 | <0.001 |  |
|  | Europe | 16 (409, 2055) | 0.231 (0.131, 0.374) | 95.93% | <0.001 | <0.001 |  |
|  | North America | 5 (49, 171) | 0.333 (0.112, 0.664) | 90.77% | 0.323 | <0.001 |  |
|  | South America | 1 (4, 48) | 0.083 (0.032, 0.202) | 0.00% | <0.001 | >0.999 |  |
|  | Africa | 1 (32, 32) | 0.985 (0.799, 0.999) | 0.00% | 0.003 | >0.999 |  |
| **Ampicillin** | | | | | | | |
| Subgroup | Proportion | K (n, N) | Proportion (LCI, HCI) | I^2^ | P1 | P2 | P3 |
| Overall |  | 12 (463, 469) | 0.941 (0.850, 0.978) | 49.63% | <0.001 | 0.026 | NA |
| Year | 1958-2010 | 3 (320, 320) | 0.983 (0.762, 0.999) | 65.97% | 0.006 | 0.053 | 0.164 |
|  | 2011-2023 | 9 (143, 149) | 0.919 (0.802, 0.969) | 36.43% | <0.001 | 0.127 |  |
| Guideline | CLSI | 8 (153, 155) | 0.948 (0.875, 0.979) | 4.99% | <0.001 | 0.392 | 0.049 |
|  | EUCAST | 3 (33, 37) | 0.811 (0.269, 0.980) | 68.06% | 0.245 | 0.044 |  |
|  | Other | 1 (277, 277) | 0.998 (0.972, 1.000) | 0.00% | <0.001 | >0.999 |  |
| Quality score | Low Quality | 4 (100, 101) | 0.971 (0.906, 0.992) | 0.00% | <0.001 | 0.822 | 0.537 |
|  | Moderate Quality | 7 (359, 363) | 0.928 (0.698, 0.986) | 66.53% | 0.004 | 0.006 |  |
|  | High Quality | 1 (4, 5) | 0.800 (0.309, 0.973) | 0.00% | 0.215 | >0.999 |  |
| AST Method | DM | 2 (50, 50) | 0.975 (0.841, 0.996) | 0.00% | <0.001 | 0.429 | 0.792 |
|  | DD | 5 (55, 58) | 0.911 (0.793, 0.965) | 0.00% | <0.001 | 0.699 |  |
|  | DD, DM | 1 (42, 43) | 0.977 (0.853, 0.997) | 0.00% | <0.001 | >0.999 |  |
|  | AS | 3 (278, 280) | 0.878 (0.059, 0.999) | 86.64% | 0.414 | <0.001 |  |
|  | DD, AS | 1 (38, 38) | 0.987 (0.825, 0.999) | 0.00% | 0.002 | >0.999 |  |
| Country | Spain | 1 (42, 42) | 0.988 (0.840, 0.999) | 0.00% | 0.002 | >0.999 | 0.016 |
|  | Tunis | 1 (8, 8) | 0.944 (0.495, 0.997) | 0.00% | 0.052 | >0.999 |  |
|  | Korea | 1 (8, 8) | 0.944 (0.495, 0.997) | 0.00% | 0.052 | >0.999 |  |
|  | Taiwan | 1 (42, 43) | 0.977 (0.853, 0.997) | 0.00% | <0.001 | >0.999 |  |
|  | Italy | 1 (277, 277) | 0.998 (0.972, 1.000) | 0.00% | <0.001 | >0.999 |  |
|  | Somalia | 1 (17, 17) | 0.972 (0.678, 0.998) | 0.00% | 0.013 | >0.999 |  |
|  | Iran | 2 (2, 2) | 0.750 (0.238, 0.966) | 0.00% | 0.341 | >0.999 |  |
|  | European country | 1 (25, 27) | 0.926 (0.748, 0.981) | 0.00% | <0.001 | >0.999 |  |
|  | China | 1 (38, 38) | 0.987 (0.825, 0.999) | 0.00% | 0.002 | >0.999 |  |
|  | Turkey | 1 (0, 2) | 0.167 (0.010, 0.806) | 0.00% | 0.299 | >0.999 |  |
|  | Ethiopia | 1 (4, 5) | 0.800 (0.309, 0.973) | 0.00% | 0.215 | >0.999 |  |
| Continent | Europe | 4 (344, 348) | 0.950 (0.543, 0.997) | 80.94% | 0.037 | 0.001 | 0.958 |
|  | Africa | 3 (29, 30) | 0.915 (0.710, 0.979) | 0.00% | 0.002 | 0.459 |  |
|  | Asia | 5 (90, 91) | 0.950 (0.848, 0.985) | 3.43% | <0.001 | 0.387 |  |
| **Ceftriaxone** | | | | | | | |
| Subgroup | Proportion | K (n, N) | Proportion (LCI, HCI) | I^2^ | P1 | P2 | P3 |
| Overall |  | 34 (1721, 1807) | 0.929 (0.885, 0.957) | 73.26% | <0.001 | <0.001 | NA |
| Year | 2011-2023 | 10 (109, 111) | 0.934 (0.860, 0.971) | 0.00% | <0.001 | 0.892 | 0.719 |
|  | 1958-2010 | 23 (1566, 1650) | 0.923 (0.862, 0.958) | 81.10% | <0.001 | <0.001 |  |
| Guideline | CLSI | 20 (392, 430) | 0.901 (0.803, 0.953) | 65.33% | <0.001 | <0.001 | 0.156 |
|  | Other | 14 (1329, 1377) | 0.950 (0.902, 0.975) | 79.28% | <0.001 | <0.001 |  |
| Quality score | Low Quality | 3 (54, 60) | 0.902 (0.644, 0.979) | 46.00% | 0.007 | 0.157 | 0.863 |
|  | Moderate Quality | 21 (1363, 1421) | 0.937 (0.881, 0.968) | 74.05% | <0.001 | <0.001 |  |
|  | High Quality | 10 (304, 326) | 0.919 (0.803, 0.969) | 73.96% | <0.001 | <0.001 |  |
| AST Method | DD | 13 (252, 266) | 0.918 (0.861, 0.953) | 14.14% | <0.001 | 0.302 | 0.091 |
|  | DM | 19 (1463, 1528) | 0.945 (0.894, 0.972) | 78.83% | <0.001 | <0.001 |  |
|  | AS | 1 (1, 1) | 0.750 (0.109, 0.987) | 0.00% | 0.501 | >0.999 |  |
|  | DD, DM, AS | 1 (5, 12) | 0.417 (0.185, 0.692) | 0.00% | 0.566 | >0.999 |  |
| Country | Serbia | 2 (38, 38) | 0.974 (0.838, 0.996) | 0.00% | <0.001 | 0.796 | 0.933 |
|  | Sweden | 1 (22, 28) | 0.786 (0.598, 0.900) | 0.00% | 0.005 | >0.999 |  |
|  | Korea | 1 (8, 8) | 0.944 (0.495, 0.997) | 0.00% | 0.052 | >0.999 |  |
|  | Saudi Arabia | 1 (8, 9) | 0.889 (0.500, 0.985) | 0.00% | 0.050 | >0.999 |  |
|  | United States | 8 (1044, 1063) | 0.967 (0.859, 0.993) | 86.97% | <0.001 | <0.001 |  |
|  | Turkey | 3 (63, 76) | 0.823 (0.721, 0.893) | 0.00% | <0.001 | 0.819 |  |
|  | Italy | 2 (83, 95) | 0.903 (0.625, 0.981) | 65.82% | 0.011 | 0.087 |  |
|  | Taiwan | 1 (28, 28) | 0.983 (0.777, 0.999) | 0.00% | 0.005 | >0.999 |  |
|  | Somalia | 1 (17, 17) | 0.972 (0.678, 0.998) | 0.00% | 0.013 | >0.999 |  |
|  | Belgium | 2 (110, 121) | 0.906 (0.838, 0.947) | 0.00% | <0.001 | 0.384 |  |
|  | Switzerland | 1 (33, 33) | 0.985 (0.804, 0.999) | 0.00% | 0.003 | >0.999 |  |
|  | China | 2 (56, 58) | 0.957 (0.863, 0.988) | 0.00% | <0.001 | 0.832 |  |
|  | Malawi | 1 (1, 1) | 0.750 (0.109, 0.987) | 0.00% | 0.501 | >0.999 |  |
|  | Iran | 1 (1, 1) | 0.750 (0.109, 0.987) | 0.00% | 0.501 | >0.999 |  |
|  | Multi-Country | 2 (35, 52) | 0.526 (0.001, 0.999) | 93.79% | 0.977 | <0.001 |  |
|  | Ethiopia | 1 (4, 5) | 0.800 (0.309, 0.973) | 0.00% | 0.215 | >0.999 |  |
|  | Brazil | 2 (61, 61) | 0.982 (0.882, 0.997) | 0.00% | <0.001 | 0.587 |  |
|  | India | 1 (5, 5) | 0.917 (0.378, 0.995) | 0.00% | 0.105 | >0.999 |  |
|  | Canada | 1 (104, 108) | 0.963 (0.905, 0.986) | 0.00% | <0.001 | >0.999 |  |
| Continent | Europe | 11 (349, 391) | 0.874 (0.820, 0.914) | 30.21% | <0.001 | 0.158 | 0.208 |
|  | Asia | 7 (106, 109) | 0.942 (0.871, 0.975) | 0.00% | <0.001 | 0.851 |  |
|  | North America | 9 (1148, 1171) | 0.965 (0.885, 0.990) | 85.11% | <0.001 | <0.001 |  |
|  | Africa | 3 (22, 23) | 0.876 (0.607, 0.970) | 0.00% | 0.012 | 0.411 |  |
|  | Multi Continent | 2 (35, 52) | 0.526 (0.001, 0.999) | 93.79% | 0.977 | <0.001 |  |
|  | South America | 2 (61, 61) | 0.982 (0.882, 0.997) | 0.00% | <0.001 | 0.587 |  |
| **Amoxicillin/CA** | | | | | | | |
| Subgroup | Proportion | K (n, N) | Proportion (LCI, HCI) | I^2^ | P1 | P2 | P3 |
| Overall |  | 12 (218, 250) | 0.818 (0.644, 0.918) | 68.62% | 0.001 | <0.001 | NA |
| Year | 1958-2010 | 5 (159, 169) | 0.958 (0.718, 0.995) | 79.58% | 0.005 | <0.001 | 0.034 |
|  | 2011-2023 | 7 (59, 81) | 0.683 (0.439, 0.856) | 59.97% | 0.138 | 0.020 |  |
| AST Method | CLSI | 9 (114, 132) | 0.797 (0.533, 0.931) | 67.44% | 0.030 | 0.002 | 0.866 |
|  | Other | 2 (81, 91) | 0.923 (0.149, 0.999) | 88.40% | 0.249 | 0.003 |  |
|  | EUCAST | 1 (23, 27) | 0.852 (0.665, 0.943) | 0.00% | 0.001 | >0.999 |  |
| Quality score | Low Quality | 2 (63, 66) | 0.946 (0.624, 0.995) | 61.43% | 0.017 | 0.107 | 0.472 |
|  | Moderate Quality | 7 (135, 158) | 0.770 (0.446, 0.933) | 76.39% | 0.097 | <0.001 |  |
|  | High Quality | 3 (20, 26) | 0.723 (0.505, 0.870) | 1.13% | 0.045 | 0.364 |  |
| AST Method | DM | 5 (159, 169) | 0.958 (0.718, 0.995) | 79.58% | 0.005 | <0.001 | 0.109 |
|  | DD | 6 (58, 80) | 0.672 (0.404, 0.861) | 66.64% | 0.204 | 0.010 |  |
|  | AS | 1 (1, 1) | 0.750 (0.109, 0.987) | 0.00% | 0.501 | >0.999 |  |
| Country | Spain | 2 (49, 49) | 0.973 (0.832, 0.996) | 0.00% | <0.001 | 0.395 | 0.065 |
|  | Serbia | 2 (30, 38) | 0.776 (0.478, 0.929) | 62.48% | 0.067 | 0.103 |  |
|  | Saudi Arabia | 2 (18, 36) | 0.357 (0.042, 0.875) | 81.15% | 0.648 | 0.021 |  |
|  | Italy | 1 (64, 64) | 0.992 (0.889, 1.000) | 0.00% | <0.001 | >0.999 |  |
|  | China | 1 (29, 29) | 0.983 (0.783, 0.999) | 0.00% | 0.004 | >0.999 |  |
|  | European country | 1 (23, 27) | 0.852 (0.665, 0.943) | 0.00% | 0.001 | >0.999 |  |
|  | Malawi | 1 (1, 1) | 0.750 (0.109, 0.987) | 0.00% | 0.501 | >0.999 |  |
|  | India | 1 (0, 1) | 0.250 (0.013, 0.891) | 0.00% | 0.501 | >0.999 |  |
|  | Ethiopia | 1 (4, 5) | 0.800 (0.309, 0.973) | 0.00% | 0.215 | >0.999 |  |
| Continent | Europe | 6 (166, 178) | 0.902 (0.753, 0.965) | 62.27% | <0.001 | 0.021 | 0.144 |
|  | Asia | 4 (47, 66) | 0.575 (0.140, 0.919) | 77.03% | 0.779 | 0.005 |  |
|  | Africa | 2 (5, 6) | 0.785 (0.374, 0.957) | 0.00% | 0.161 | 0.884 |  |
| **aztreonam** | | | | | | | |
| Subgroup | Proportion | K (n, N) | Proportion (LCI, HCI) | I^2^ | P1 | P2 | P3 |
| Overall |  | 32 (2120, 2314) | 0.911 (0.878, 0.936) | 73.33% | <0.001 | <0.001 | NA |
| Year | 1958-2010 | 21 (1850, 2035) | 0.906 (0.866, 0.935) | 80.34% | <0.001 | <0.001 | 0.406 |
|  | 2011-2023 | 9 (250, 257) | 0.940 (0.883, 0.970) | 10.80% | <0.001 | 0.345 |  |
| Guideline | Other | 11 (1141, 1247) | 0.915 (0.848, 0.954) | 85.75% | <0.001 | <0.001 | 0.957 |
|  | CLSI | 21 (979, 1067) | 0.908 (0.867, 0.937) | 56.49% | <0.001 | <0.001 |  |
| Quality score | Low Quality | 3 (78, 85) | 0.916 (0.835, 0.960) | 0.00% | <0.001 | 0.879 | 0.292 |
|  | Moderate Quality | 20 (1141, 1265) | 0.893 (0.840, 0.929) | 72.88% | <0.001 | <0.001 |  |
|  | High Quality | 9 (901, 964) | 0.942 (0.889, 0.970) | 77.43% | <0.001 | <0.001 |  |
| AST Method | DD | 6 (409, 459) | 0.883 (0.850, 0.910) | 0.00% | <0.001 | 0.450 | 0.555 |
|  | DM | 21 (1417, 1553) | 0.911 (0.867, 0.941) | 77.44% | <0.001 | <0.001 |  |
|  | AS | 3 (274, 279) | 0.925 (0.558, 0.992) | 63.39% | 0.031 | 0.065 |  |
|  | DD, DM | 1 (4, 6) | 0.667 (0.268, 0.916) | 0.00% | 0.423 | >0.999 |  |
|  | DD, AS | 1 (16, 17) | 0.941 (0.680, 0.992) | 0.00% | 0.007 | >0.999 |  |
| Country | Saudi Arabia | 3 (293, 330) | 0.909 (0.816, 0.957) | 60.36% | <0.001 | 0.080 | 0.336 |
|  | Spain | 4 (146, 158) | 0.920 (0.864, 0.954) | 0.00% | <0.001 | 0.714 |  |
|  | Korea | 2 (39, 41) | 0.939 (0.778, 0.986) | 5.88% | <0.001 | 0.303 |  |
|  | United States | 4 (175, 190) | 0.894 (0.689, 0.970) | 78.74% | 0.002 | 0.003 |  |
|  | Mexico | 1 (105, 119) | 0.882 (0.811, 0.929) | 0.00% | <0.001 | >0.999 |  |
|  | Italy | 3 (629, 648) | 0.973 (0.937, 0.988) | 55.10% | <0.001 | 0.108 |  |
|  | Japan | 1 (65, 66) | 0.985 (0.900, 0.998) | 0.00% | <0.001 | >0.999 |  |
|  | Somalia | 1 (15, 17) | 0.882 (0.632, 0.970) | 0.00% | 0.007 | >0.999 |  |
|  | Iran | 1 (1, 1) | 0.750 (0.109, 0.987) | 0.00% | 0.501 | >0.999 |  |
|  | China | 4 (373, 418) | 0.899 (0.730, 0.967) | 59.15% | <0.001 | 0.062 |  |
|  | Belgium | 2 (88, 121) | 0.739 (0.577, 0.855) | 64.89% | 0.005 | 0.091 |  |
|  | United Kingdom | 1 (4, 4) | 0.900 (0.326, 0.994) | 0.00% | 0.140 | >0.999 |  |
|  | Malawi | 1 (1, 1) | 0.750 (0.109, 0.987) | 0.00% | 0.501 | >0.999 |  |
|  | Denmark | 1 (118, 124) | 0.952 (0.896, 0.978) | 0.00% | <0.001 | >0.999 |  |
|  | Taiwan | 2 (62, 70) | 0.905 (0.464, 0.991) | 80.20% | 0.065 | 0.025 |  |
|  | India | 1 (6, 6) | 0.929 (0.423, 0.996) | 0.00% | 0.081 | >0.999 |  |
| Continent | Asia | 14 (839, 932) | 0.902 (0.855, 0.935) | 49.93% | <0.001 | 0.017 | 0.808 |
|  | Europe | 11 (985, 1055) | 0.927 (0.855, 0.965) | 86.45% | <0.001 | <0.001 |  |
|  | North America | 5 (280, 309) | 0.888 (0.770, 0.949) | 71.88% | <0.001 | 0.007 |  |
|  | Africa | 2 (16, 18) | 0.865 (0.626, 0.961) | 0.00% | 0.007 | 0.610 |  |
| **Piperacillin/Tazobactam** | | | | | | | |
| Subgroup | Proportion | K (n, N) | Proportion (LCI, HCI) | I^2^ | P1 | P2 | P3 |
| Overall |  | 74 (3888, 6032) | 0.657 (0.603, 0.706) | 91.43% | <0.001 | <0.001 | NA |
| Year | 2011-2023 | 22 (504, 718) | 0.713 (0.605, 0.801) | 81.37% | <0.001 | <0.001 | 0.177 |
|  | 1958-2010 | 50 (3369, 5298) | 0.631 (0.567, 0.690) | 93.29% | <0.001 | <0.001 |  |
| Guideline | CLSI | 42 (1565, 2695) | 0.615 (0.535, 0.689) | 88.84% | 0.005 | <0.001 | 0.267 |
|  | Other | 30 (2297, 3302) | 0.695 (0.617, 0.763) | 93.88% | <0.001 | <0.001 |  |
|  | EUCAST | 2 (26, 35) | 0.733 (0.560, 0.856) | 0.00% | 0.010 | 0.347 |  |
| Quality score | Low Quality | 7 (113, 208) | 0.785 (0.401, 0.952) | 91.53% | 0.135 | <0.001 | 0.328 |
|  | Moderate Quality | 41 (2110, 3216) | 0.623 (0.552, 0.689) | 89.89% | <0.001 | <0.001 |  |
|  | High Quality | 26 (1665, 2608) | 0.687 (0.597, 0.765) | 93.47% | <0.001 | <0.001 |  |
| AST Method | DM | 41 (2683, 4338) | 0.634 (0.559, 0.702) | 93.57% | <0.001 | <0.001 | 0.601 |
|  | DD | 29 (931, 1326) | 0.694 (0.630, 0.751) | 75.73% | <0.001 | <0.001 |  |
|  | AS | 3 (257, 338) | 0.601 (0.055, 0.975) | 97.74% | 0.806 | <0.001 |  |
|  | DD, AS | 1 (17, 30) | 0.567 (0.388, 0.729) | 0.00% | 0.467 | >0.999 |  |
| Country | Saudi Arabia | 3 (161, 257) | 0.656 (0.441, 0.822) | 68.05% | 0.151 | 0.044 | <0.001 |
|  | Canada | 4 (224, 358) | 0.678 (0.459, 0.839) | 92.86% | 0.109 | <0.001 |  |
|  | Serbia | 2 (35, 38) | 0.908 (0.735, 0.973) | 9.90% | <0.001 | 0.292 |  |
|  | Tunis | 1 (7, 8) | 0.875 (0.463, 0.983) | 0.00% | 0.069 | >0.999 |  |
|  | Sweden | 1 (24, 28) | 0.857 (0.676, 0.945) | 0.00% | <0.001 | >0.999 |  |
|  | Korea | 3 (25, 209) | 0.195 (0.039, 0.591) | 86.12% | 0.120 | <0.001 |  |
|  | Taiwan | 9 (495, 636) | 0.756 (0.650, 0.837) | 84.21% | <0.001 | <0.001 |  |
|  | Multi-Country | 5 (599, 952) | 0.684 (0.550, 0.793) | 79.94% | 0.008 | <0.001 |  |
|  | United States | 5 (567, 883) | 0.620 (0.404, 0.797) | 95.95% | 0.274 | <0.001 |  |
|  | Turkey | 4 (204, 281) | 0.570 (0.171, 0.895) | 95.81% | 0.765 | <0.001 |  |
|  | Italy | 9 (815, 1086) | 0.666 (0.517, 0.788) | 94.18% | 0.030 | <0.001 |  |
|  | China | 4 (68, 178) | 0.365 (0.158, 0.638) | 90.37% | 0.333 | <0.001 |  |
|  | Somalia | 1 (17, 17) | 0.972 (0.678, 0.998) | 0.00% | 0.013 | >0.999 |  |
|  | Spain | 4 (139, 185) | 0.753 (0.598, 0.862) | 70.74% | 0.002 | 0.017 |  |
|  | Belgium | 2 (83, 121) | 0.676 (0.492, 0.817) | 73.82% | 0.060 | 0.051 |  |
|  | United Kingdom | 2 (41, 44) | 0.922 (0.798, 0.972) | 0.00% | <0.001 | 0.845 |  |
|  | European country | 2 (101, 219) | 0.554 (0.287, 0.793) | 85.19% | 0.705 | 0.009 |  |
|  | Malaysia | 1 (75, 84) | 0.893 (0.807, 0.943) | 0.00% | <0.001 | >0.999 |  |
|  | India | 3 (4, 9) | 0.467 (0.173, 0.786) | 7.44% | 0.857 | 0.339 |  |
|  | Malawi | 1 (1, 1) | 0.750 (0.109, 0.987) | 0.00% | 0.501 | >0.999 |  |
|  | France | 2 (24, 25) | 0.937 (0.741, 0.987) | 0.00% | 0.001 | 0.614 |  |
|  | Egypt | 1 (15, 32) | 0.469 (0.306, 0.639) | 0.00% | 0.724 | >0.999 |  |
|  | Thailand | 1 (43, 64) | 0.672 (0.549, 0.775) | 0.00% | 0.007 | >0.999 |  |
|  | Denmark | 1 (0, 124) | 0.004 (0.000, 0.061) | 0.00% | <0.001 | >0.999 |  |
|  | Israel | 1 (7, 10) | 0.700 (0.376, 0.900) | 0.00% | 0.220 | >0.999 |  |
|  | England | 1 (80, 100) | 0.800 (0.710, 0.867) | 0.00% | <0.001 | >0.999 |  |
|  | Latin America | 1 (34, 83) | 0.410 (0.309, 0.518) | 0.00% | 0.102 | >0.999 |  |
| Continent | Asia | 26 (936, 1527) | 0.605 (0.487, 0.712) | 92.07% | 0.081 | <0.001 | 0.582 |
|  | North America | 9 (791, 1241) | 0.648 (0.508, 0.767) | 94.33% | 0.038 | <0.001 |  |
|  | Europe | 30 (1546, 2251) | 0.705 (0.617, 0.780) | 91.29% | <0.001 | <0.001 |  |
|  | Africa | 4 (40, 58) | 0.793 (0.396, 0.957) | 67.39% | 0.136 | 0.027 |  |
|  | Multi Continent | 4 (541, 872) | 0.660 (0.465, 0.812) | 82.25% | 0.105 | <0.001 |  |
|  | South America | 1 (34, 83) | 0.410 (0.309, 0.518) | 0.00% | 0.102 | >0.999 |  |
| **Piperacillin** | | | | | | | |
| Subgroup | Proportion | K (n, N) | Proportion (LCI, HCI) | I^2^ | P1 | P2 | P3 |
| Overall |  | 40 (1925, 2560) | 0.722 (0.643, 0.789) | 89.23% | <0.001 | <0.001 | NA |
| Year | 1958-2010 | 29 (1670, 2212) | 0.705 (0.607, 0.787) | 91.20% | <0.001 | <0.001 | 0.708 |
|  | 2011-2023 | 9 (220, 310) | 0.726 (0.570, 0.841) | 75.91% | 0.006 | <0.001 |  |
| Guideline | CLSI | 18 (384, 634) | 0.704 (0.538, 0.829) | 86.69% | 0.017 | <0.001 | 0.899 |
|  | Other | 19 (1407, 1748) | 0.728 (0.620, 0.814) | 91.81% | <0.001 | <0.001 |  |
|  | EUCAST | 1 (7, 8) | 0.875 (0.463, 0.983) | 0.00% | 0.069 | >0.999 |  |
|  | CLSI-EUCAST | 2 (127, 170) | 0.747 (0.676, 0.807) | 0.00% | <0.001 | 0.860 |  |
| Quality score | Low Quality | 5 (82, 117) | 0.703 (0.485, 0.856) | 69.74% | 0.067 | 0.010 | 0.789 |
|  | Moderate Quality | 29 (1312, 1737) | 0.712 (0.626, 0.786) | 88.10% | <0.001 | <0.001 |  |
|  | High Quality | 6 (531, 706) | 0.759 (0.470, 0.918) | 92.35% | 0.076 | <0.001 |  |
| AST Method | DD | 12 (450, 575) | 0.793 (0.673, 0.877) | 71.70% | <0.001 | <0.001 | 0.535 |
|  | DM | 21 (1071, 1482) | 0.673 (0.544, 0.780) | 92.13% | 0.009 | <0.001 |  |
|  | AS | 6 (399, 497) | 0.738 (0.498, 0.889) | 92.46% | 0.051 | <0.001 |  |
|  | DD, DM | 1 (5, 6) | 0.833 (0.369, 0.977) | 0.00% | 0.142 | >0.999 |  |
| Country | India | 4 (13, 17) | 0.695 (0.266, 0.934) | 46.49% | 0.380 | 0.132 | <0.001 |
|  | Saudi Arabia | 2 (65, 71) | 0.915 (0.824, 0.962) | 0.00% | <0.001 | 0.971 |  |
|  | Spain | 4 (100, 142) | 0.715 (0.534, 0.846) | 69.80% | 0.021 | 0.019 |  |
|  | Tunis | 1 (7, 8) | 0.875 (0.463, 0.983) | 0.00% | 0.069 | >0.999 |  |
|  | Sweden | 2 (24, 49) | 0.465 (0.166, 0.792) | 82.81% | 0.854 | 0.016 |  |
|  | United States | 4 (105, 172) | 0.515 (0.287, 0.737) | 86.37% | 0.904 | <0.001 |  |
|  | Mexico | 1 (105, 119) | 0.882 (0.811, 0.929) | 0.00% | <0.001 | >0.999 |  |
|  | Japan | 2 (68, 76) | 0.833 (0.336, 0.980) | 87.48% | 0.169 | 0.005 |  |
|  | Turkey | 2 (207, 245) | 0.777 (0.359, 0.956) | 95.55% | 0.181 | <0.001 |  |
|  | Italy | 4 (591, 641) | 0.905 (0.839, 0.945) | 69.33% | <0.001 | 0.021 |  |
|  | Taiwan | 3 (130, 198) | 0.542 (0.242, 0.814) | 92.12% | 0.801 | <0.001 |  |
|  | Somalia | 1 (17, 17) | 0.972 (0.678, 0.998) | 0.00% | 0.013 | >0.999 |  |
|  | Iran | 1 (1, 1) | 0.750 (0.109, 0.987) | 0.00% | 0.501 | >0.999 |  |
|  | China | 4 (315, 435) | 0.673 (0.431, 0.849) | 90.69% | 0.157 | <0.001 |  |
|  | Belgium | 2 (65, 121) | 0.536 (0.443, 0.627) | 6.86% | 0.444 | 0.300 |  |
|  | France | 1 (88, 100) | 0.880 (0.800, 0.931) | 0.00% | <0.001 | >0.999 |  |
|  | Germany | 1 (23, 24) | 0.958 (0.756, 0.994) | 0.00% | 0.002 | >0.999 |  |
|  | Denmark | 1 (1, 124) | 0.008 (0.001, 0.055) | 0.00% | <0.001 | >0.999 |  |
| Continent | Asia | 16 (592, 798) | 0.725 (0.605, 0.819) | 82.79% | <0.001 | <0.001 | 0.386 |
|  | Europe | 17 (1099, 1446) | 0.728 (0.590, 0.832) | 92.63% | 0.002 | <0.001 |  |
|  | Africa | 2 (24, 25) | 0.926 (0.699, 0.985) | 0.00% | 0.003 | 0.368 |  |
|  | North America | 5 (210, 291) | 0.613 (0.367, 0.812) | 91.19% | 0.370 | <0.001 |  |
| **Cefepime** | | | | | | | |
| Subgroup | Proportion | K (n, N) | Proportion (LCI, HCI) | I^2^ | P1 | P2 | P3 |
| Overall |  | 60 (2579, 3934) | 0.709 (0.644, 0.767) | 91.47% | <0.001 | <0.001 | NA |
| Year | 2011-2023 | 17 (256, 528) | 0.608 (0.427, 0.763) | 87.11% | 0.241 | <0.001 | 0.096 |
|  | 1958-2010 | 42 (2320, 3402) | 0.737 (0.668, 0.796) | 92.32% | <0.001 | <0.001 |  |
| Guideline | CLSI | 39 (1268, 2210) | 0.693 (0.607, 0.768) | 89.62% | <0.001 | <0.001 | 0.771 |
|  | Other | 18 (1219, 1593) | 0.738 (0.633, 0.822) | 92.38% | <0.001 | <0.001 |  |
|  | EUCAST | 3 (92, 131) | 0.702 (0.618, 0.774) | 0.00% | <0.001 | 0.749 |  |
| Quality score | Low Quality | 5 (72, 132) | 0.652 (0.302, 0.891) | 85.04% | 0.400 | <0.001 | 0.101 |
|  | Moderate Quality | 30 (1187, 2016) | 0.643 (0.541, 0.734) | 90.89% | 0.007 | <0.001 |  |
|  | High Quality | 25 (1320, 1786) | 0.777 (0.693, 0.844) | 91.21% | <0.001 | <0.001 |  |
| AST Method | DM | 31 (1575, 2635) | 0.682 (0.596, 0.758) | 92.53% | <0.001 | <0.001 | 0.159 |
|  | DD | 19 (612, 740) | 0.801 (0.743, 0.848) | 55.71% | <0.001 | 0.002 |  |
|  | DD, DM | 4 (41, 94) | 0.522 (0.145, 0.875) | 86.78% | 0.925 | <0.001 |  |
|  | AS | 4 (258, 334) | 0.441 (0.053, 0.917) | 96.47% | 0.860 | <0.001 |  |
|  | DD, AS | 1 (22, 29) | 0.759 (0.573, 0.880) | 0.00% | 0.008 | >0.999 |  |
| Country | Saudi Arabia | 2 (32, 35) | 0.913 (0.763, 0.972) | 0.00% | <0.001 | 0.753 | 0.029 |
|  | Canada | 2 (111, 139) | 0.797 (0.722, 0.856) | 0.00% | <0.001 | 0.375 |  |
|  | Serbia | 2 (25, 38) | 0.655 (0.491, 0.789) | 0.00% | 0.063 | 0.393 |  |
|  | Korea | 2 (37, 41) | 0.902 (0.766, 0.963) | 0.00% | <0.001 | 0.772 |  |
|  | Taiwan | 11 (548, 707) | 0.755 (0.596, 0.865) | 90.39% | 0.003 | <0.001 |  |
|  | Mexico | 1 (25, 119) | 0.210 (0.146, 0.292) | 0.00% | <0.001 | >0.999 |  |
|  | Multi-Country | 5 (471, 925) | 0.574 (0.421, 0.714) | 82.14% | 0.346 | <0.001 |  |
|  | France | 2 (80, 115) | 0.696 (0.606, 0.773) | 0.00% | <0.001 | 0.978 |  |
|  | United States | 5 (120, 264) | 0.757 (0.416, 0.931) | 90.54% | 0.131 | <0.001 |  |
|  | Turkey | 6 (192, 316) | 0.607 (0.551, 0.659) | 0.00% | <0.001 | 0.547 |  |
|  | Italy | 3 (532, 610) | 0.871 (0.837, 0.899) | 17.83% | <0.001 | 0.296 |  |
|  | China | 5 (119, 188) | 0.671 (0.336, 0.891) | 92.08% | 0.316 | <0.001 |  |
|  | Spain | 4 (74, 108) | 0.680 (0.374, 0.884) | 77.47% | 0.244 | 0.004 |  |
|  | Belgium | 2 (42, 121) | 0.348 (0.268, 0.437) | 0.00% | 0.001 | 0.517 |  |
|  | United Kingdom | 1 (3, 4) | 0.750 (0.238, 0.966) | 0.00% | 0.341 | >0.999 |  |
|  | Japan | 1 (52, 54) | 0.963 (0.864, 0.991) | 0.00% | <0.001 | >0.999 |  |
|  | European country | 1 (20, 27) | 0.741 (0.547, 0.871) | 0.00% | 0.017 | >0.999 |  |
|  | India | 3 (11, 13) | 0.761 (0.187, 0.978) | 58.43% | 0.388 | 0.090 |  |
|  | England | 2 (85, 110) | 0.742 (0.560, 0.867) | 43.58% | 0.011 | 0.183 |  |
| Continent | Asia | 24 (799, 1038) | 0.782 (0.672, 0.862) | 87.61% | <0.001 | <0.001 | 0.180 |
|  | North America | 8 (256, 522) | 0.690 (0.452, 0.857) | 94.32% | 0.115 | <0.001 |  |
|  | Europe | 23 (1053, 1449) | 0.676 (0.583, 0.757) | 87.41% | <0.001 | <0.001 |  |
|  | Multi Continent | 5 (471, 925) | 0.574 (0.421, 0.714) | 82.14% | 0.346 | <0.001 |  |
| **Moxifloxacin** | | | | | | | |
| Subgroup | Proportion | K (n, N) | Proportion (LCI, HCI) | I^2^ | P1 | P2 | P3 |
| Overall |  | 29 (249, 2409) | 0.132 (0.093, 0.184) | 84.03% | <0.001 | <0.001 | NA |
| Year | 1958-2010 | 10 (75, 977) | 0.128 (0.067, 0.231) | 74.14% | <0.001 | <0.001 | 0.921 |
|  | 2011-2023 | 17 (156, 1359) | 0.123 (0.078, 0.187) | 84.77% | <0.001 | <0.001 |  |
| Guideline | CLSI | 21 (162, 1292) | 0.147 (0.098, 0.213) | 80.22% | <0.001 | <0.001 | 0.505 |
|  | CLSI-EUCAST | 1 (48, 763) | 0.063 (0.048, 0.083) | 0.00% | <0.001 | >0.999 |  |
|  | Other | 6 (32, 227) | 0.139 (0.075, 0.246) | 64.35% | <0.001 | 0.015 |  |
|  | EUCAST | 1 (7, 127) | 0.055 (0.027, 0.111) | 0.00% | <0.001 | >0.999 |  |
| Quality score | Low Quality | 2 (3, 7) | 0.467 (0.015, 0.981) | 73.74% | 0.949 | 0.051 | 0.003 |
|  | Moderate Quality | 21 (146, 2013) | 0.097 (0.064, 0.144) | 79.52% | <0.001 | <0.001 |  |
|  | High Quality | 6 (100, 389) | 0.260 (0.215, 0.310) | 11.38% | <0.001 | 0.343 |  |
| AST Method | DD | 7 (15, 424) | 0.161 (0.030, 0.543) | 86.21% | 0.076 | <0.001 | 0.292 |
|  | DM | 14 (187, 1580) | 0.143 (0.091, 0.217) | 88.24% | <0.001 | <0.001 |  |
|  | AS | 5 (11, 210) | 0.063 (0.032, 0.119) | 21.22% | <0.001 | 0.279 |  |
|  | DD, DM | 3 (36, 195) | 0.187 (0.138, 0.248) | 0.00% | <0.001 | 0.483 |  |
| Country | Taiwan | 4 (6, 36) | 0.252 (0.043, 0.714) | 62.95% | 0.286 | 0.044 | 0.636 |
|  | India | 1 (0, 4) | 0.100 (0.006, 0.674) | 0.00% | 0.140 | >0.999 |  |
|  | Iran | 2 (30, 525) | 0.051 (0.004, 0.429) | 96.55% | 0.030 | <0.001 |  |
|  | Saudi Arabia | 1 (4, 9) | 0.444 (0.177, 0.749) | 0.00% | 0.739 | >0.999 |  |
|  | Multi-Country | 1 (48, 763) | 0.063 (0.048, 0.083) | 0.00% | <0.001 | >0.999 |  |
|  | United States | 6 (32, 222) | 0.165 (0.100, 0.259) | 51.11% | <0.001 | 0.069 |  |
|  | Spain | 3 (15, 131) | 0.104 (0.024, 0.354) | 83.70% | 0.007 | 0.002 |  |
|  | Germany | 4 (34, 252) | 0.085 (0.021, 0.290) | 87.44% | 0.002 | <0.001 |  |
|  | Hungary | 2 (14, 157) | 0.116 (0.025, 0.400) | 87.63% | 0.014 | 0.004 |  |
|  | United Kingdom | 1 (3, 40) | 0.075 (0.024, 0.208) | 0.00% | <0.001 | >0.999 |  |
|  | Korea | 1 (1, 16) | 0.063 (0.009, 0.335) | 0.00% | 0.009 | >0.999 |  |
|  | China | 3 (62, 254) | 0.246 (0.174, 0.337) | 56.53% | <0.001 | 0.100 |  |
| Continent | Asia | 12 (103, 844) | 0.171 (0.096, 0.286) | 82.99% | <0.001 | <0.001 | 0.434 |
|  | Multi Continent | 1 (48, 763) | 0.063 (0.048, 0.083) | 0.00% | <0.001 | >0.999 |  |
|  | North America | 6 (32, 222) | 0.165 (0.100, 0.259) | 51.11% | <0.001 | 0.069 |  |
|  | Europe | 10 (66, 580) | 0.099 (0.052, 0.182) | 82.23% | <0.001 | <0.001 |  |
| **Doxycycline** | | | | | | | |
| Subgroup | Proportion | K (n, N) | Proportion (LCI, HCI) | I^2^ | P1 | P2 | P3 |
| Overall |  | 11 (53, 1099) | 0.071 (0.030, 0.158) | 86.80% | <0.001 | <0.001 | NA |
| Year | 1958-2010 | 6 (23, 433) | 0.082 (0.026, 0.231) | 78.90% | <0.001 | <0.001 | 0.987 |
|  | 2011-2023 | 4 (29, 616) | 0.075 (0.015, 0.303) | 93.65% | 0.003 | <0.001 |  |
| Guideline | Other | 5 (21, 247) | 0.071 (0.026, 0.180) | 70.54% | <0.001 | 0.009 | 0.820 |
|  | CLSI | 6 (32, 852) | 0.073 (0.017, 0.262) | 91.93% | <0.001 | <0.001 |  |
| Quality score | Low Quality | 1 (14, 66) | 0.212 (0.130, 0.327) | 0.00% | <0.001 | >0.999 | 0.557 |
|  | Moderate Quality | 9 (27, 961) | 0.049 (0.015, 0.154) | 87.37% | <0.001 | <0.001 |  |
|  | High Quality | 1 (12, 72) | 0.167 (0.097, 0.271) | 0.00% | <0.001 | >0.999 |  |
| AST Method | DM | 9 (48, 1029) | 0.060 (0.023, 0.147) | 87.84% | <0.001 | <0.001 | 0.207 |
|  | DD | 1 (2, 64) | 0.031 (0.008, 0.117) | 0.00% | <0.001 | >0.999 |  |
|  | DD, DM | 1 (3, 6) | 0.500 (0.168, 0.832) | 0.00% | >0.999 | >0.999 |  |
| Country | France | 1 (14, 66) | 0.212 (0.130, 0.327) | 0.00% | <0.001 | >0.999 | 0.700 |
|  | Multi-Country | 1 (0, 230) | 0.002 (0.000, 0.034) | 0.00% | <0.001 | >0.999 |  |
|  | Italy | 1 (1, 50) | 0.020 (0.003, 0.129) | 0.00% | <0.001 | >0.999 |  |
|  | China | 3 (20, 528) | 0.109 (0.012, 0.558) | 94.50% | 0.078 | <0.001 |  |
|  | Spain | 2 (3, 98) | 0.038 (0.007, 0.180) | 37.47% | <0.001 | 0.206 |  |
|  | Switzerland | 1 (3, 33) | 0.091 (0.030, 0.247) | 0.00% | <0.001 | >0.999 |  |
|  | Hungary | 1 (10, 30) | 0.333 (0.190, 0.516) | 0.00% | 0.074 | >0.999 |  |
|  | Thailand | 1 (2, 64) | 0.031 (0.008, 0.117) | 0.00% | <0.001 | >0.999 |  |
| Continent | Europe | 6 (31, 277) | 0.103 (0.043, 0.226) | 75.90% | <0.001 | <0.001 | 0.169 |
|  | Multi Continent | 1 (0, 230) | 0.002 (0.000, 0.034) | 0.00% | <0.001 | >0.999 |  |
|  | Asia | 4 (22, 592) | 0.081 (0.014, 0.355) | 92.21% | 0.009 | <0.001 |  |
| **Ticarcillin** | | | | | | | |
| Subgroup | Proportion | K (n, N) | Proportion (LCI, HCI) | I^2^ | P1 | P2 | P3 |
| Overall |  | 15 (1260, 1646) | 0.816 (0.737, 0.876) | 86.06% | <0.001 | <0.001 | NA |
| Year | 1958-2010 | 12 (1184, 1560) | 0.805 (0.714, 0.872) | 88.21% | <0.001 | <0.001 | 0.816 |
|  | 2011-2023 | 2 (45, 55) | 0.817 (0.692, 0.899) | 0.00% | <0.001 | 0.655 |  |
| Guideline | Other | 7 (612, 701) | 0.889 (0.803, 0.941) | 83.04% | <0.001 | <0.001 | 0.022 |
|  | CLSI | 7 (641, 937) | 0.694 (0.592, 0.781) | 63.69% | <0.001 | 0.011 |  |
|  | EUCAST | 1 (7, 8) | 0.875 (0.463, 0.983) | 0.00% | 0.069 | >0.999 |  |
| Quality score | Low Quality | 4 (97, 120) | 0.803 (0.421, 0.958) | 83.58% | 0.110 | <0.001 | 0.345 |
|  | Moderate Quality | 9 (881, 1214) | 0.786 (0.705, 0.850) | 77.97% | <0.001 | <0.001 |  |
|  | High Quality | 2 (282, 312) | 0.903 (0.864, 0.931) | 0.00% | <0.001 | 0.906 |  |
| AST Method | DM | 9 (1105, 1452) | 0.827 (0.718, 0.899) | 91.26% | <0.001 | <0.001 | 0.985 |
|  | DD | 5 (117, 147) | 0.803 (0.674, 0.889) | 26.22% | <0.001 | 0.247 |  |
|  | AS | 1 (38, 47) | 0.809 (0.671, 0.897) | 0.00% | <0.001 | >0.999 |  |
| Country | France | 2 (138, 166) | 0.897 (0.449, 0.989) | 90.31% | 0.073 | 0.001 | 0.843 |
|  | India | 2 (7, 9) | 0.722 (0.206, 0.963) | 44.66% | 0.417 | 0.179 |  |
|  | Spain | 3 (115, 140) | 0.860 (0.528, 0.971) | 88.87% | 0.037 | <0.001 |  |
|  | Tunis | 1 (7, 8) | 0.875 (0.463, 0.983) | 0.00% | 0.069 | >0.999 |  |
|  | Multi-Country | 1 (519, 763) | 0.680 (0.646, 0.712) | 0.00% | <0.001 | >0.999 |  |
|  | Italy | 2 (305, 338) | 0.902 (0.866, 0.930) | 0.00% | <0.001 | 0.987 |  |
|  | Australia | 1 (25, 45) | 0.556 (0.410, 0.692) | 0.00% | 0.457 | >0.999 |  |
|  | United States | 1 (75, 99) | 0.758 (0.664, 0.832) | 0.00% | <0.001 | >0.999 |  |
|  | Canada | 1 (31, 31) | 0.984 (0.794, 0.999) | 0.00% | 0.004 | >0.999 |  |
|  | China | 1 (38, 47) | 0.809 (0.671, 0.897) | 0.00% | <0.001 | >0.999 |  |
| Continent | Europe | 7 (558, 644) | 0.874 (0.761, 0.938) | 87.42% | <0.001 | <0.001 | 0.554 |
|  | Asia | 3 (45, 56) | 0.780 (0.591, 0.897) | 15.97% | 0.006 | 0.304 |  |
|  | Africa | 1 (7, 8) | 0.875 (0.463, 0.983) | 0.00% | 0.069 | >0.999 |  |
|  | Multi Continent | 1 (519, 763) | 0.680 (0.646, 0.712) | 0.00% | <0.001 | >0.999 |  |
|  | Australia | 1 (25, 45) | 0.556 (0.410, 0.692) | 0.00% | 0.457 | >0.999 |  |
|  | North America | 2 (106, 130) | 0.910 (0.364, 0.994) | 76.87% | 0.115 | 0.038 |  |
| **Ceftazidime/Avibactam** | | | | | | | |
| Subgroup | Proportion | K (n, N) | Proportion (LCI, HCI) | I^2^ | P1 | P2 | P3 |
| Overall |  | 8 (329, 1180) | 0.406 (0.241, 0.596) | 91.66% | 0.331 | <0.001 | NA |
| Guideline | CLSI-EUCAST | 1 (0, 7) | 0.063 (0.004, 0.539) | 0.00% | 0.064 | >0.999 | <0.001 |
|  | CLSI | 4 (190, 609) | 0.536 (0.285, 0.770) | 91.25% | 0.791 | <0.001 |  |
|  | Other | 2 (138, 226) | 0.610 (0.540, 0.675) | 11.91% | 0.002 | 0.287 |  |
|  | EUCAST | 1 (1, 338) | 0.003 (0.000, 0.021) | 0.00% | <0.001 | >0.999 |  |
| Quality score | Low Quality | 1 (0, 7) | 0.063 (0.004, 0.539) | 0.00% | 0.064 | >0.999 | 0.120 |
|  | Moderate Quality | 6 (230, 1043) | 0.350 (0.178, 0.574) | 92.62% | 0.185 | <0.001 |  |
|  | High Quality | 1 (99, 130) | 0.762 (0.681, 0.827) | 0.00% | <0.001 | >0.999 |  |
| Country | Italy | 1 (0, 7) | 0.063 (0.004, 0.539) | 0.00% | 0.064 | >0.999 | <0.001 |
|  | United States | 2 (130, 171) | 0.760 (0.691, 0.818) | 0.00% | <0.001 | 0.943 |  |
|  | Canada | 1 (60, 100) | 0.600 (0.501, 0.691) | 0.00% | 0.047 | >0.999 |  |
|  | Spain | 1 (79, 123) | 0.642 (0.554, 0.722) | 0.00% | 0.002 | >0.999 |  |
|  | France | 1 (59, 103) | 0.573 (0.476, 0.665) | 0.00% | 0.141 | >0.999 |  |
|  | Multi-Country | 2 (1, 676) | 0.002 (0.000, 0.012) | 0.00% | <0.001 | 0.687 |  |
| Continent | Europe | 3 (138, 233) | 0.580 (0.439, 0.710) | 65.29% | 0.264 | 0.056 | <0.001 |
|  | North America | 3 (190, 271) | 0.705 (0.581, 0.805) | 73.69% | 0.002 | 0.022 |  |
|  | Multi Continent | 2 (1, 676) | 0.002 (0.000, 0.012) | 0.00% | <0.001 | 0.687 |  |
| **Cefiderocol** | | | | | | | |
| Subgroup | Proportion | K (n, N) | Proportion (LCI, HCI) | I^2^ | P1 | P2 | P3 |
| Overall |  | 7 (818, 1224) | 0.047 (0.001, 0.785) | 96.58% | 0.171 | <0.001 | NA |
| Guideline | CLSI | 4 (1, 179) | 0.014 (0.004, 0.048) | 0.00% | <0.001 | 0.976 | 0.487 |
|  | EUCAST | 3 (817, 1045) | 0.189 (0.000, 0.999) | 98.21% | 0.724 | <0.001 |  |
| Quality score | Moderate Quality | 6 (818, 1204) | 0.052 (0.000, 0.874) | 97.03% | 0.240 | <0.001 | 0.902 |
|  | High Quality | 1 (0, 20) | 0.024 (0.001, 0.287) | 0.00% | 0.009 | >0.999 |  |
| Country | United States | 2 (0, 87) | 0.011 (0.002, 0.076) | 0.00% | <0.001 | 0.882 | <0.001 |
|  | Multi-Country | 1 (816, 819) | 0.996 (0.989, 0.999) | 0.00% | <0.001 | >0.999 |  |
|  | Spain | 1 (1, 123) | 0.008 (0.001, 0.055) | 0.00% | <0.001 | >0.999 |  |
|  | France | 1 (0, 103) | 0.005 (0.000, 0.072) | 0.00% | <0.001 | >0.999 |  |
|  | China | 1 (1, 72) | 0.014 (0.002, 0.092) | 0.00% | <0.001 | >0.999 |  |
|  | Andalusia | 1 (0, 20) | 0.024 (0.001, 0.287) | 0.00% | 0.009 | >0.999 |  |
| Continent | North America | 2 (0, 87) | 0.011 (0.002, 0.076) | 0.00% | <0.001 | 0.882 | 0.877 |
|  | Europe | 4 (817, 1065) | 0.119 (0.000, 0.989) | 97.62% | 0.547 | <0.001 |  |
|  | Asia | 1 (1, 72) | 0.014 (0.002, 0.092) | 0.00% | <0.001 | >0.999 |  |
| **Tobramycin** | | | | | | | |
| Subgroup | Proportion | K (n, N) | Proportion (LCI, HCI) | I^2^ | P1 | P2 | P3 |
| Overall |  | 37 (1871, 2520) | 0.748 (0.699, 0.791) | 81.45% | <0.001 | <0.001 | NA |
| Year | 1958-2010 | 28 (1676, 2236) | 0.765 (0.718, 0.806) | 79.53% | <0.001 | <0.001 | 0.364 |
|  | 2011-2023 | 6 (160, 218) | 0.703 (0.391, 0.897) | 89.38% | 0.195 | <0.001 |  |
| Guideline | Other | 22 (1387, 1906) | 0.726 (0.661, 0.782) | 84.80% | <0.001 | <0.001 | 0.222 |
|  | CLSI | 15 (484, 614) | 0.785 (0.703, 0.849) | 72.25% | <0.001 | <0.001 |  |
| Quality score | Low Quality | 4 (74, 105) | 0.660 (0.403, 0.848) | 77.83% | 0.218 | 0.004 | 0.628 |
|  | Moderate Quality | 22 (777, 1044) | 0.748 (0.685, 0.802) | 71.80% | <0.001 | <0.001 |  |
|  | High Quality | 11 (1020, 1371) | 0.763 (0.673, 0.835) | 90.47% | <0.001 | <0.001 |  |
| AST Method | DM | 22 (1402, 1849) | 0.783 (0.723, 0.833) | 85.06% | <0.001 | <0.001 | 0.013 |
|  | DD | 11 (432, 589) | 0.732 (0.667, 0.788) | 54.83% | <0.001 | 0.014 |  |
|  | AS | 3 (27, 67) | 0.387 (0.118, 0.749) | 69.90% | 0.562 | 0.036 |  |
|  | DD, DM, AS | 1 (10, 15) | 0.667 (0.406, 0.854) | 0.00% | 0.206 | >0.999 |  |
| Country | Canada | 2 (118, 150) | 0.779 (0.683, 0.852) | 26.48% | <0.001 | 0.244 | <0.001 |
|  | India | 1 (1, 4) | 0.250 (0.034, 0.762) | 0.00% | 0.341 | >0.999 |  |
|  | Spain | 5 (130, 160) | 0.798 (0.668, 0.886) | 56.87% | <0.001 | 0.055 |  |
|  | Sweden | 1 (14, 28) | 0.500 (0.323, 0.677) | 0.00% | >0.999 | >0.999 |  |
|  | Mexico | 1 (90, 119) | 0.756 (0.671, 0.825) | 0.00% | <0.001 | >0.999 |  |
|  | Japan | 1 (9, 10) | 0.900 (0.533, 0.986) | 0.00% | 0.037 | >0.999 |  |
|  | United States | 6 (451, 566) | 0.765 (0.615, 0.869) | 84.79% | 0.001 | <0.001 |  |
|  | Italy | 7 (520, 729) | 0.725 (0.678, 0.768) | 37.72% | <0.001 | 0.141 |  |
|  | Australia | 1 (36, 45) | 0.800 (0.658, 0.893) | 0.00% | <0.001 | >0.999 |  |
|  | China | 2 (31, 57) | 0.733 (0.141, 0.979) | 74.82% | 0.482 | 0.046 |  |
|  | Taiwan | 1 (47, 50) | 0.940 (0.830, 0.981) | 0.00% | <0.001 | >0.999 |  |
|  | Korea | 1 (2, 16) | 0.125 (0.031, 0.386) | 0.00% | 0.010 | >0.999 |  |
|  | Germany | 2 (100, 114) | 0.891 (0.725, 0.962) | 38.42% | <0.001 | 0.203 |  |
|  | Multi-Country | 2 (100, 116) | 0.861 (0.785, 0.913) | 0.00% | <0.001 | 0.548 |  |
|  | European country | 1 (102, 192) | 0.531 (0.461, 0.601) | 0.00% | 0.387 | >0.999 |  |
|  | Latin America | 1 (68, 83) | 0.819 (0.722, 0.888) | 0.00% | <0.001 | >0.999 |  |
|  | Brazil | 1 (23, 46) | 0.500 (0.359, 0.641) | 0.00% | >0.999 | >0.999 |  |
|  | Turkey | 1 (29, 35) | 0.829 (0.667, 0.921) | 0.00% | <0.001 | >0.999 |  |
| Continent | North America | 9 (659, 835) | 0.763 (0.680, 0.830) | 76.91% | <0.001 | <0.001 | 0.918 |
|  | Asia | 7 (160, 217) | 0.709 (0.398, 0.900) | 88.51% | 0.181 | <0.001 |  |
|  | Europe | 17 (895, 1258) | 0.745 (0.683, 0.799) | 78.00% | <0.001 | <0.001 |  |
|  | Australia | 1 (36, 45) | 0.800 (0.658, 0.893) | 0.00% | <0.001 | >0.999 |  |
|  | Multi Continent | 1 (30, 36) | 0.833 (0.675, 0.923) | 0.00% | <0.001 | >0.999 |  |
|  | South America | 2 (91, 129) | 0.681 (0.327, 0.904) | 92.63% | 0.316 | <0.001 |  |
| **Cefoperazone** | | | | | | | |
| Subgroup | Proportion | K (n, N) | Proportion (LCI, HCI) | I^2^ | P1 | P2 | P3 |
| Overall |  | 13 (456, 873) | 0.595 (0.409, 0.757) | 93.78% | 0.316 | <0.001 | NA |
| Year | 1958-2010 | 10 (310, 680) | 0.536 (0.346, 0.716) | 92.22% | 0.718 | <0.001 | 0.738 |
|  | 2011-2023 | 2 (30, 63) | 0.682 (0.122, 0.971) | 73.23% | 0.585 | 0.053 |  |
| Guideline | Other | 6 (396, 715) | 0.722 (0.432, 0.899) | 97.11% | 0.128 | <0.001 | 0.163 |
|  | CLSI | 7 (60, 158) | 0.383 (0.248, 0.539) | 57.90% | 0.140 | 0.027 |  |
| Quality score | Low Quality | 1 (116, 130) | 0.892 (0.826, 0.935) | 0.00% | <0.001 | >0.999 | 0.131 |
|  | Moderate Quality | 8 (247, 589) | 0.487 (0.290, 0.689) | 91.49% | 0.907 | <0.001 |  |
|  | High Quality | 4 (93, 154) | 0.642 (0.409, 0.823) | 74.01% | 0.231 | 0.009 |  |
| AST Method | DM | 7 (236, 377) | 0.524 (0.302, 0.738) | 92.56% | 0.838 | <0.001 | 0.558 |
|  | DD, AS | 1 (24, 57) | 0.421 (0.301, 0.552) | 0.00% | 0.235 | >0.999 |  |
|  | DD | 4 (193, 433) | 0.844 (0.228, 0.990) | 95.86% | 0.255 | <0.001 |  |
|  | DD, DM | 1 (3, 6) | 0.500 (0.168, 0.832) | 0.00% | >0.999 | >0.999 |  |
| Country | United States | 5 (240, 304) | 0.699 (0.297, 0.927) | 95.00% | 0.332 | <0.001 | 0.059 |
|  | China | 6 (205, 558) | 0.421 (0.277, 0.580) | 88.87% | 0.328 | <0.001 |  |
|  | India | 2 (11, 11) | 0.923 (0.609, 0.989) | 0.00% | 0.017 | 0.936 |  |
| Continent | North America | 5 (240, 304) | 0.699 (0.297, 0.927) | 95.00% | 0.332 | <0.001 | 0.234 |
|  | Asia | 8 (216, 569) | 0.476 (0.322, 0.635) | 86.85% | 0.775 | <0.001 |  |
| **Doripenem** | | | | | | | |
| Subgroup | Proportion | K (n, N) | Proportion (LCI, HCI) | I^2^ | P1 | P2 | P3 |
| Overall |  | 3 (315, 321) | 0.979 (0.932, 0.994) | 26.44% | <0.001 | 0.257 | NA |
| **Cefotaxime** | | | | | | | |
| Subgroup | Proportion | K (n, N) | Proportion (LCI, HCI) | I^2^ | P1 | P2 | P3 |
| Overall |  | 26 (1237, 1493) | 0.850 (0.774, 0.904) | 87.67% | <0.001 | <0.001 | NA |
| Year | 1958-2010 | 15 (971, 1135) | 0.868 (0.769, 0.929) | 91.10% | <0.001 | <0.001 | 0.269 |
|  | 2011-2023 | 9 (217, 308) | 0.759 (0.584, 0.876) | 69.79% | 0.005 | <0.001 |  |
| Guideline | Other | 11 (747, 836) | 0.859 (0.707, 0.939) | 91.35% | <0.001 | <0.001 | 0.971 |
|  | CLSI | 13 (462, 622) | 0.825 (0.732, 0.890) | 75.03% | <0.001 | <0.001 |  |
|  | EUCAST | 2 (28, 35) | 0.802 (0.503, 0.942) | 27.35% | 0.048 | 0.241 |  |
| Quality score | Low Quality | 5 (101, 121) | 0.902 (0.538, 0.986) | 86.64% | 0.036 | <0.001 | 0.854 |
|  | Moderate Quality | 16 (699, 825) | 0.837 (0.726, 0.909) | 86.28% | <0.001 | <0.001 |  |
|  | High Quality | 5 (437, 547) | 0.867 (0.683, 0.952) | 92.75% | <0.001 | <0.001 |  |
| AST Method | DD | 7 (168, 182) | 0.899 (0.787, 0.956) | 48.57% | <0.001 | 0.070 | 0.407 |
|  | DM | 16 (804, 1017) | 0.842 (0.755, 0.903) | 87.19% | <0.001 | <0.001 |  |
|  | AS | 3 (265, 294) | 0.694 (0.078, 0.984) | 94.80% | 0.626 | <0.001 |  |
| Country | Saudi Arabia | 2 (41, 44) | 0.929 (0.801, 0.977) | 0.00% | <0.001 | 0.574 | 0.695 |
|  | Spain | 4 (122, 147) | 0.863 (0.662, 0.953) | 74.75% | 0.002 | 0.008 |  |
|  | Tunis | 1 (8, 8) | 0.944 (0.495, 0.997) | 0.00% | 0.052 | >0.999 |  |
|  | Sweden | 1 (11, 28) | 0.393 (0.233, 0.580) | 0.00% | 0.261 | >0.999 |  |
|  | Korea | 2 (11, 24) | 0.619 (0.024, 0.991) | 86.33% | 0.821 | 0.007 |  |
|  | Mexico | 1 (72, 119) | 0.605 (0.515, 0.689) | 0.00% | 0.023 | >0.999 |  |
|  | Italy | 1 (261, 277) | 0.942 (0.908, 0.964) | 0.00% | <0.001 | >0.999 |  |
|  | China | 1 (26, 29) | 0.897 (0.724, 0.966) | 0.00% | <0.001 | >0.999 |  |
|  | Taiwan | 1 (25, 28) | 0.893 (0.716, 0.965) | 0.00% | <0.001 | >0.999 |  |
|  | United States | 4 (298, 372) | 0.895 (0.624, 0.978) | 91.67% | 0.010 | <0.001 |  |
|  | United Kingdom | 2 (40, 44) | 0.900 (0.773, 0.960) | 0.00% | <0.001 | >0.999 |  |
|  | European country | 1 (20, 27) | 0.741 (0.547, 0.871) | 0.00% | 0.017 | >0.999 |  |
|  | Malawi | 1 (1, 1) | 0.750 (0.109, 0.987) | 0.00% | 0.501 | >0.999 |  |
|  | Iran | 2 (60, 94) | 0.637 (0.535, 0.727) | 0.00% | 0.009 | 0.740 |  |
|  | Brazil | 1 (45, 46) | 0.978 (0.861, 0.997) | 0.00% | <0.001 | >0.999 |  |
|  | Turkey | 1 (196, 205) | 0.956 (0.918, 0.977) | 0.00% | <0.001 | >0.999 |  |
| continent | Asia | 8 (163, 219) | 0.803 (0.592, 0.919) | 79.62% | 0.008 | <0.001 | 0.643 |
|  | Europe | 10 (650, 728) | 0.864 (0.727, 0.938) | 89.25% | <0.001 | <0.001 |  |
|  | Africa | 2 (9, 9) | 0.887 (0.484, 0.985) | 0.00% | 0.057 | 0.428 |  |
|  | North America | 5 (370, 491) | 0.840 (0.675, 0.930) | 90.65% | <0.001 | <0.001 |  |
|  | South America | 1 (45, 46) | 0.978 (0.861, 0.997) | 0.00% | <0.001 | >0.999 |  |
| **aztreonam/CA** | | | | | | | |
| Subgroup | Proportion | K (n, N) | Proportion (LCI, HCI) | I^2^ | P1 | P2 | P3 |
| Overall |  | 4 (172, 219) | 0.796 (0.602, 0.910) | 86.04% | 0.005 | <0.001 | NA |
| Country | Spain | 2 (62, 98) | 0.633 (0.533, 0.722) | 0.00% | 0.009 | 0.911 | <0.001 |
|  | Belgium | 2 (110, 121) | 0.909 (0.842, 0.949) | 0.00% | <0.001 | 0.681 |  |
| **Ceftolozane/Tazobactam** | | | | | | | |
| Subgroup | Proportion | K (n, N) | Proportion (LCI, HCI) | I^2^ | P1 | P2 | P3 |
| Overall |  | 8 (377, 573) | 0.605 (0.492, 0.708) | 77.48% | 0.068 | <0.001 | NA |
| Year | 2011-2023 | 5 (349, 514) | 0.679 (0.610, 0.741) | 53.75% | <0.001 | 0.071 | 0.014 |
|  | 1958-2010 | 1 (14, 34) | 0.412 (0.261, 0.581) | 0.00% | 0.306 | >0.999 |  |
| Guideline | CLSI | 5 (207, 313) | 0.562 (0.352, 0.753) | 81.33% | 0.569 | <0.001 | 0.317 |
|  | Other | 2 (156, 226) | 0.690 (0.627, 0.747) | 0.00% | <0.001 | 0.751 |  |
|  | EUCAST | 1 (14, 34) | 0.412 (0.261, 0.581) | 0.00% | 0.306 | >0.999 |  |
| Quality score | Moderate Quality | 6 (363, 548) | 0.636 (0.543, 0.720) | 72.77% | 0.005 | 0.003 | 0.258 |
|  | High Quality | 2 (14, 25) | 0.647 (0.015, 0.996) | 88.98% | 0.805 | 0.003 |  |
| AST Method | DD | 1 (1, 9) | 0.111 (0.015, 0.500) | 0.00% | 0.050 | >0.999 | 0.025 |
|  | DM | 7 (376, 564) | 0.633 (0.531, 0.725) | 75.34% | 0.011 | <0.001 |  |
| Country | Saudi Arabia | 1 (1, 9) | 0.111 (0.015, 0.500) | 0.00% | 0.050 | >0.999 | 0.513 |
|  | Canada | 1 (73, 100) | 0.730 (0.635, 0.808) | 0.00% | <0.001 | >0.999 |  |
|  | Spain | 1 (86, 123) | 0.699 (0.613, 0.774) | 0.00% | <0.001 | >0.999 |  |
|  | Ireland | 1 (14, 34) | 0.412 (0.261, 0.581) | 0.00% | 0.306 | >0.999 |  |
|  | Taiwan | 1 (119, 179) | 0.665 (0.593, 0.730) | 0.00% | <0.001 | >0.999 |  |
|  | France | 2 (82, 115) | 0.828 (0.331, 0.979) | 65.09% | 0.176 | 0.091 |  |
|  | United States | 1 (2, 13) | 0.154 (0.039, 0.451) | 0.00% | 0.027 | >0.999 |  |
| Continent | Asia | 2 (120, 188) | 0.378 (0.040, 0.899) | 84.95% | 0.716 | 0.010 | 0.590 |
|  | North America | 2 (75, 113) | 0.437 (0.052, 0.916) | 91.19% | 0.850 | <0.001 |  |
|  | Europe | 4 (182, 272) | 0.644 (0.488, 0.775) | 76.41% | 0.071 | 0.005 |  |
| **Norfloxacin** | | | | | | | |
| Subgroup | Proportion | K (n, N) | Proportion (LCI, HCI) | I^2^ | P1 | P2 | P3 |
| Overall |  | 9 (217, 402) | 0.666 (0.473, 0.815) | 85.96% | 0.090 | <0.001 | NA |
| year | 1958-2010 | 4 (110, 189) | 0.652 (0.356, 0.864) | 80.95% | 0.312 | 0.001 | 0.256 |
|  | 2011-2023 | 1 (37, 114) | 0.325 (0.245, 0.416) | 0.00% | <0.001 | >0.999 |  |
| Guideline | Other | 5 (80, 126) | 0.756 (0.385, 0.939) | 88.04% | 0.166 | <0.001 | 0.696 |
|  | CLSI | 4 (137, 276) | 0.596 (0.346, 0.804) | 86.94% | 0.458 | <0.001 |  |
| Quality score | Low Quality | 2 (61, 62) | 0.976 (0.890, 0.995) | 0.00% | <0.001 | >0.999 | 0.004 |
|  | Moderate Quality | 6 (119, 226) | 0.600 (0.375, 0.790) | 81.46% | 0.386 | <0.001 |  |
|  | High Quality | 1 (37, 114) | 0.325 (0.245, 0.416) | 0.00% | <0.001 | >0.999 |  |
| AST Method | DM | 8 (216, 401) | 0.663 (0.464, 0.817) | 87.57% | 0.107 | <0.001 | 0.828 |
|  | DD | 1 (1, 1) | 0.750 (0.109, 0.987) | 0.00% | 0.501 | >0.999 |  |
| Country | Italy | 1 (20, 20) | 0.976 (0.713, 0.999) | 0.00% | 0.009 | >0.999 | <0.001 |
|  | Spain | 2 (72, 75) | 0.956 (0.871, 0.986) | 0.00% | <0.001 | 0.436 |  |
|  | Saudi Arabia | 2 (20, 63) | 0.319 (0.216, 0.444) | 0.00% | 0.005 | 0.436 |  |
|  | Mexico | 1 (58, 119) | 0.487 (0.399, 0.577) | 0.00% | 0.783 | >0.999 |  |
|  | United States | 1 (9, 10) | 0.900 (0.533, 0.986) | 0.00% | 0.037 | >0.999 |  |
|  | Iran | 1 (1, 1) | 0.750 (0.109, 0.987) | 0.00% | 0.501 | >0.999 |  |
|  | China | 1 (37, 114) | 0.325 (0.245, 0.416) | 0.00% | <0.001 | >0.999 |  |
| Continent | Europe | 3 (92, 95) | 0.960 (0.890, 0.986) | 0.00% | <0.001 | 0.678 | <0.001 |
|  | Asia | 4 (58, 178) | 0.327 (0.262, 0.399) | 0.00% | <0.001 | 0.600 |  |
|  | North America | 2 (67, 129) | 0.697 (0.211, 0.952) | 77.34% | 0.448 | 0.036 |  |
| **Trovafloxacin** | | | | | | | |
| Subgroup | Proportion | K (n, N) | Proportion (LCI, HCI) | I^2^ | P1 | P2 | P3 |
| Overall |  | 9 (91, 1021) | 0.095 (0.060, 0.148) | 74.59% | <0.001 | <0.001 | NA |
| Quality score | Low Quality | 1 (8, 99) | 0.081 (0.041, 0.153) | 0.00% | <0.001 | >0.999 | 0.285 |
|  | Moderate Quality | 3 (13, 80) | 0.130 (0.027, 0.442) | 73.38% | 0.025 | 0.023 |  |
|  | High Quality | 5 (70, 842) | 0.081 (0.051, 0.128) | 68.70% | <0.001 | 0.012 |  |
| Country | United States | 3 (47, 487) | 0.097 (0.074, 0.127) | 0.00% | <0.001 | 0.839 | <0.001 |
|  | Saudi Arabia | 1 (0, 27) | 0.018 (0.001, 0.230) | 0.00% | 0.005 | >0.999 |  |
|  | Spain | 1 (11, 33) | 0.333 (0.195, 0.508) | 0.00% | 0.061 | >0.999 |  |
|  | Multi-Country | 1 (10, 80) | 0.125 (0.069, 0.217) | 0.00% | <0.001 | >0.999 |  |
|  | Canada | 1 (14, 119) | 0.118 (0.071, 0.189) | 0.00% | <0.001 | >0.999 |  |
|  | European country | 1 (4, 192) | 0.021 (0.008, 0.054) | 0.00% | <0.001 | >0.999 |  |
|  | Latin America | 1 (5, 83) | 0.060 (0.025, 0.137) | 0.00% | <0.001 | >0.999 |  |
| Continent | North America | 4 (61, 606) | 0.101 (0.080, 0.128) | 0.00% | <0.001 | 0.848 | 0.951 |
|  | Asia | 2 (10, 107) | 0.075 (0.013, 0.330) | 49.39% | 0.006 | 0.160 |  |
|  | Europe | 2 (15, 225) | 0.095 (0.005, 0.699) | 96.07% | 0.153 | <0.001 |  |
|  | South America | 1 (5, 83) | 0.060 (0.025, 0.137) | 0.00% | <0.001 | >0.999 |  |
| **Cefuroxime** | | | | | | | |
| Subgroup | Proportion | K (n, N) | Proportion (LCI, HCI) | I^2^ | P1 | P2 | P3 |
| Overall |  | 5 (180, 187) | 0.950 (0.903, 0.975) | 0.00% | <0.001 | 0.533 | NA |
| **Gatifloxacin** | | | | | | | |
| Subgroup | Proportion | K (n, N) | Proportion (LCI, HCI) | I^2^ | P1 | P2 | P3 |
| Overall |  | 11 (194, 2710) | 0.093 (0.059, 0.145) | 88.15% | <0.001 | <0.001 | NA |
| Year | 2011-2023 | 2 (27, 193) | 0.124 (0.057, 0.249) | 53.04% | <0.001 | 0.144 | 0.660 |
|  | 1958-2010 | 9 (167, 2517) | 0.089 (0.052, 0.146) | 88.99% | <0.001 | <0.001 |  |
| Guideline | CLSI | 3 (27, 246) | 0.082 (0.026, 0.232) | 67.80% | <0.001 | 0.045 | 0.857 |
|  | Other | 8 (167, 2464) | 0.095 (0.056, 0.157) | 90.03% | <0.001 | <0.001 |  |
| Quality score | Low Quality | 1 (3, 43) | 0.070 (0.023, 0.195) | 0.00% | <0.001 | >0.999 | 0.358 |
|  | Moderate Quality | 4 (128, 1772) | 0.142 (0.058, 0.307) | 95.27% | <0.001 | <0.001 |  |
|  | High Quality | 6 (63, 895) | 0.071 (0.040, 0.123) | 76.01% | <0.001 | <0.001 |  |
| AST Method | DD, DM | 2 (27, 193) | 0.124 (0.057, 0.249) | 53.04% | <0.001 | 0.144 | 0.660 |
|  | DM | 9 (167, 2517) | 0.089 (0.052, 0.146) | 88.99% | <0.001 | <0.001 |  |
| Country | Taiwan | 1 (3, 43) | 0.070 (0.023, 0.195) | 0.00% | <0.001 | >0.999 | 0.371 |
|  | Multi-Country | 3 (84, 1621) | 0.063 (0.025, 0.147) | 78.95% | <0.001 | 0.009 |  |
|  | Iran | 1 (24, 150) | 0.160 (0.110, 0.228) | 0.00% | <0.001 | >0.999 |  |
|  | United States | 3 (56, 502) | 0.153 (0.064, 0.325) | 90.52% | <0.001 | <0.001 |  |
|  | Canada | 1 (18, 119) | 0.151 (0.097, 0.227) | 0.00% | <0.001 | >0.999 |  |
|  | European country | 1 (4, 192) | 0.021 (0.008, 0.054) | 0.00% | <0.001 | >0.999 |  |
|  | Latin America | 1 (5, 83) | 0.060 (0.025, 0.137) | 0.00% | <0.001 | >0.999 |  |
| Continent | Asia | 3 (37, 273) | 0.136 (0.096, 0.190) | 13.87% | <0.001 | 0.313 | 0.024 |
|  | Multi Continent | 2 (74, 1541) | 0.038 (0.011, 0.121) | 31.50% | <0.001 | 0.227 |  |
|  | North America | 4 (74, 621) | 0.151 (0.082, 0.262) | 86.07% | <0.001 | <0.001 |  |
|  | Europe | 1 (4, 192) | 0.021 (0.008, 0.054) | 0.00% | <0.001 | >0.999 |  |
|  | South America | 1 (5, 83) | 0.060 (0.025, 0.137) | 0.00% | <0.001 | >0.999 |  |
| **Cefperazone/Sulbactam** | | | | | | | |
| Subgroup | Proportion | K (n, N) | Proportion (LCI, HCI) | I^2^ | P1 | P2 | P3 |
| Overall |  | 13 (595, 2553) | 0.428 (0.269, 0.603) | 94.24% | 0.422 | <0.001 | NA |
| Year | 2011-2023 | 9 (424, 2070) | 0.399 (0.246, 0.574) | 87.09% | 0.255 | <0.001 | 0.166 |
|  | 1958-2010 | 3 (51, 353) | 0.166 (0.091, 0.284) | 40.37% | <0.001 | 0.187 |  |
| Guideline | Other | 2 (165, 453) | 0.580 (0.020, 0.989) | 99.28% | 0.881 | <0.001 | 0.563 |
|  | CLSI | 11 (430, 2100) | 0.377 (0.243, 0.532) | 84.48% | 0.119 | <0.001 |  |
| Quality score | Low Quality | 2 (469, 2006) | 0.620 (0.033, 0.988) | 99.29% | 0.804 | <0.001 | 0.768 |
|  | Moderate Quality | 7 (75, 404) | 0.342 (0.157, 0.593) | 82.30% | 0.213 | <0.001 |  |
|  | High Quality | 4 (51, 143) | 0.434 (0.221, 0.675) | 79.05% | 0.602 | 0.002 |  |
| AST Method | DM | 6 (176, 306) | 0.479 (0.196, 0.776) | 94.32% | 0.901 | <0.001 | 0.730 |
|  | AS | 1 (349, 1876) | 0.186 (0.169, 0.204) | 0.00% | <0.001 | >0.999 |  |
|  | DD | 5 (66, 349) | 0.557 (0.124, 0.918) | 88.95% | 0.837 | <0.001 |  |
|  | DD, AS | 1 (4, 22) | 0.182 (0.070, 0.396) | 0.00% | 0.007 | >0.999 |  |
| Country | United States | 1 (120, 130) | 0.923 (0.863, 0.958) | 0.00% | <0.001 | >0.999 | <0.001 |
|  | China | 7 (449, 2387) | 0.229 (0.160, 0.315) | 83.13% | <0.001 | <0.001 |  |
|  | Somalia | 1 (15, 17) | 0.882 (0.632, 0.970) | 0.00% | 0.007 | >0.999 |  |
|  | India | 2 (5, 8) | 0.546 (0.050, 0.965) | 65.25% | 0.907 | 0.090 |  |
|  | Turkey | 2 (6, 11) | 0.536 (0.266, 0.786) | 0.00% | 0.808 | 0.530 |  |
| Continent | North America | 1 (120, 130) | 0.923 (0.863, 0.958) | 0.00% | <0.001 | >0.999 | <0.001 |
|  | Asia | 9 (454, 2395) | 0.247 (0.173, 0.340) | 81.55% | <0.001 | <0.001 |  |
|  | Africa | 1 (15, 17) | 0.882 (0.632, 0.970) | 0.00% | 0.007 | >0.999 |  |
|  | Europe | 2 (6, 11) | 0.536 (0.266, 0.786) | 0.00% | 0.808 | 0.530 |  |
| **Ceftizoxime** | | | | | | | |
| Subgroup | Proportion | K (n, N) | Proportion (LCI, HCI) | I^2^ | P1 | P2 | P3 |
| Overall |  | 3 (142, 168) | 0.897 (0.419, 0.991) | 74.89% | 0.088 | 0.019 | NA |
| Quality score | Moderate Quality | 2 (75, 75) | 0.958 (0.334, 0.999) | 69.31% | 0.108 | 0.071 | 0.469 |
|  | High Quality | 1 (67, 93) | 0.720 (0.621, 0.802) | 0.00% | <0.001 | >0.999 |  |
| AST Method | DM | 2 (141, 167) | 0.939 (0.229, 0.999) | 87.45% | 0.175 | 0.005 | 0.661 |
|  | DD | 1 (1, 1) | 0.750 (0.109, 0.987) | 0.00% | 0.501 | >0.999 |  |
| Country | United States | 1 (74, 74) | 0.993 (0.902, 1.000) | 0.00% | <0.001 | >0.999 | 0.005 |
|  | Iran | 2 (68, 94) | 0.721 (0.623, 0.802) | 0.00% | <0.001 | 0.927 |  |
| Continent | North America | 1 (74, 74) | 0.993 (0.902, 1.000) | 0.00% | <0.001 | >0.999 | 0.005 |
|  | Asia | 2 (68, 94) | 0.721 (0.623, 0.802) | 0.00% | <0.001 | 0.927 |  |
| **Cefazolin** | | | | | | | |
| Subgroup | Proportion | K (n, N) | Proportion (LCI, HCI) | I^2^ | P1 | P2 | P3 |
| Overall |  | 5 (44, 54) | 0.831 (0.479, 0.964) | 65.24% | 0.062 | 0.021 | NA |
| Year | 1958-2010 | 2 (15, 16) | 0.894 (0.603, 0.979) | 0.00% | 0.015 | 0.429 | 0.560 |
|  | 2011-2023 | 3 (29, 38) | 0.786 (0.227, 0.979) | 72.54% | 0.313 | 0.026 |  |
| Guideline | Other | 2 (17, 26) | 0.753 (0.112, 0.987) | 78.37% | 0.491 | 0.032 | 0.436 |
|  | CLSI | 3 (27, 28) | 0.892 (0.648, 0.974) | 0.00% | 0.006 | 0.382 |  |
| AST Method | DM | 1 (10, 10) | 0.955 (0.552, 0.997) | 0.00% | 0.035 | >0.999 | 0.012 |
|  | AS | 2 (8, 17) | 0.467 (0.254, 0.692) | 0.00% | 0.781 | 0.430 |  |
|  | DD, DM | 1 (5, 6) | 0.833 (0.369, 0.977) | 0.00% | 0.142 | >0.999 |  |
|  | DD, AS | 1 (21, 21) | 0.977 (0.723, 0.999) | 0.00% | 0.009 | >0.999 |  |
| Country | Japan | 1 (10, 10) | 0.955 (0.552, 0.997) | 0.00% | 0.035 | >0.999 | 0.178 |
|  | Iran | 1 (1, 1) | 0.750 (0.109, 0.987) | 0.00% | 0.501 | >0.999 |  |
|  | Korea | 1 (7, 16) | 0.438 (0.225, 0.676) | 0.00% | 0.618 | >0.999 |  |
|  | China | 2 (26, 27) | 0.923 (0.602, 0.990) | 29.88% | 0.019 | 0.232 |  |
| **Sparfloxacin** | | | | | | | |
| Subgroup | Proportion | K (n, N) | Proportion (LCI, HCI) | I^2^ | P1 | P2 | P3 |
| Overall |  | 3 (87, 926) | 0.165 (0.056, 0.395) | 95.43% | 0.008 | <0.001 | NA |
| Guideline | Other | 2 (39, 163) | 0.241 (0.181, 0.312) | 0.00% | <0.001 | 0.338 | <0.001 |
|  | CLSI | 1 (48, 763) | 0.063 (0.048, 0.083) | 0.00% | <0.001 | >0.999 |  |
| Quality score | Low Quality | 1 (29, 130) | 0.223 (0.160, 0.303) | 0.00% | <0.001 | >0.999 | 0.731 |
|  | Moderate Quality | 2 (58, 796) | 0.142 (0.026, 0.508) | 95.25% | 0.054 | <0.001 |  |
| **Carbenicillin** | | | | | | | |
| Subgroup | Proportion | K (n, N) | Proportion (LCI, HCI) | I^2^ | P1 | P2 | P3 |
| Overall |  | 3 (139, 187) | 0.760 (0.413, 0.935) | 90.39% | 0.134 | <0.001 | NA |
| Guideline | CLSI | 1 (18, 42) | 0.429 (0.289, 0.580) | 0.00% | 0.356 | >0.999 | 0.304 |
|  | Other | 2 (121, 145) | 0.927 (0.325, 0.997) | 81.96% | 0.128 | 0.019 |  |
| AST Method | DM | 2 (64, 88) | 0.872 (0.058, 0.999) | 90.88% | 0.423 | <0.001 | 0.847 |
|  | DD | 1 (75, 99) | 0.758 (0.664, 0.832) | 0.00% | <0.001 | >0.999 |  |
| **Netilmicin** | | | | | | | |
| Subgroup | Proportion | K (n, N) | Proportion (LCI, HCI) | I^2^ | P1 | P2 | P3 |
| Overall |  | 6 (302, 424) | 0.527 (0.289, 0.753) | 91.62% | 0.832 | <0.001 | NA |
| Year | 1958-2010 | 4 (281, 377) | 0.574 (0.280, 0.824) | 93.29% | 0.637 | <0.001 | 0.502 |
|  | 2011-2023 | 1 (0, 1) | 0.250 (0.013, 0.891) | 0.00% | 0.501 | >0.999 |  |
| Guideline | Other | 3 (291, 387) | 0.657 (0.388, 0.852) | 94.26% | 0.250 | <0.001 | 0.104 |
|  | CLSI | 3 (11, 37) | 0.308 (0.184, 0.468) | 0.00% | 0.020 | 0.966 |  |
| Quality score | Moderate Quality | 4 (270, 343) | 0.658 (0.395, 0.851) | 84.29% | 0.235 | <0.001 | 0.110 |
|  | High Quality | 2 (32, 81) | 0.392 (0.263, 0.537) | 40.00% | 0.143 | 0.197 |  |
| AST Method | AS | 1 (231, 277) | 0.834 (0.785, 0.873) | 0.00% | <0.001 | >0.999 | <0.001 |
|  | DM | 2 (60, 110) | 0.538 (0.389, 0.681) | 60.01% | 0.621 | 0.114 |  |
|  | DD | 3 (11, 37) | 0.308 (0.184, 0.468) | 0.00% | 0.020 | 0.966 |  |
| Country | Italy | 2 (270, 341) | 0.740 (0.475, 0.899) | 93.29% | 0.073 | <0.001 | 0.179 |
|  | Turkey | 2 (11, 36) | 0.311 (0.184, 0.475) | 0.00% | 0.025 | 0.849 |  |
|  | India | 1 (0, 1) | 0.250 (0.013, 0.891) | 0.00% | 0.501 | >0.999 |  |
|  | Brazil | 1 (21, 46) | 0.457 (0.320, 0.600) | 0.00% | 0.556 | >0.999 |  |
| Continent | Europe | 4 (281, 377) | 0.574 (0.280, 0.824) | 93.29% | 0.637 | <0.001 | 0.771 |
|  | Asia | 1 (0, 1) | 0.250 (0.013, 0.891) | 0.00% | 0.501 | >0.999 |  |
|  | South America | 1 (21, 46) | 0.457 (0.320, 0.600) | 0.00% | 0.556 | >0.999 |  |
| **Ampicillin/Sulbactam** | | | | | | | |
| Subgroup | Proportion | K (n, N) | Proportion (LCI, HCI) | I^2^ | P1 | P2 | P3 |
| Overall |  | 4 (85, 98) | 0.876 (0.599, 0.971) | 50.41% | 0.014 | 0.109 | NA |
| **Cefoxitin** | | | | | | | |
| Subgroup | Proportion | K (n, N) | Proportion (LCI, HCI) | I^2^ | P1 | P2 | P3 |
| Overall |  | 3 (134, 138) | 0.960 (0.907, 0.983) | 0.00% | <0.001 | 0.529 | NA |

K: Number of reports, n: Number of resistant isolates, N: Number of total isolates, LCI: 95% Lower Confidence Interval, HCI: 95% Higher Confidence Interval, P1: P-value of difference from zero resistance rate, P2: P-value of heterogeneity between reports, P3: P-value of difference between groups
